# Insight into Role of Selection in the Evolution of Polyglutamine Tracts in Humans

**DOI:** 10.1371/journal.pone.0041167

**Published:** 2012-07-25

**Authors:** Hongwei Li, Jing Liu, Keliang Wu, Yuan Chen

**Affiliations:** 1 College of Veterinary Medicine, China Agricultural University, Beijing, China; 2 College of Animal Science and Technology, China Agricultural University, Beijing, China; 3 College of Biological Sciences, China Agricultural University, Beijing, China; Institut Jacques Monod, France

## Abstract

Glutamine tandem repeats are common in eukaryotic proteins. Although some studies have proposed that replication slippage plays an important role in shaping these repeats, the role of natural selection in glutamine tandem repeat evolution is somewhat unclear. In this study, we identified all of the glutamine tandem repeats containing four or more glutamines in human proteins and then estimated the nonsynonymous (*d_N_*) and synonymous (*d_S_*) substitution rates for the regions flanking the glutamine tandem repeats and the proteins containing them. The results indicated that most of the proteins containing polyglutamine (polyQ) tracts of four or more glutamines have undergone purifying selection, and that the purifying selection for the regions flanking the repeats is weaker. Additionally, we observed that the conserved repeats were under stronger selection constraints than the nonconserved repeats. Interestingly, we found that there was a higher level of purifying selection for the regions flanking the polyQ tracts encoded by pure CAG codons compared with those encoded by mixed codons. Based on our findings, we propose that selection has played a more important role than was previously speculated in constraining the expansion of polyQ tracts encoded by pure codons.

## Introduction

Tandem repeats of single amino acids are very common in eukaryotic proteins [1], [2]. Most of the single amino acid repeats (AARs) are embedded in intrinsically unstructured regions (IURs) that do not form compact structures under normal solvation conditions [3]–[5]. Using X-ray crystallography, Kim et al. recently reported that the secondary structure of a poly17Q region in the huntingtin protein adopts multiple conformations [6]. Numerous studies have reported that variation in the repeat size is associated with morphological evolution or changes in animals [7], [8] and certain neurological, developmental, and neuromuscular disorders in humans [9], [10].

Polyglutamine (polyQ) tracts are the most intensively studied type of AAR because several neurodegenerative disorders result from the expansion of the polyQ tracts in human proteins [9], [11], [12]. Functionally, polyQ tracts may play a role in the activation of transcription; therefore, their expansion may disrupt the transcription of the gene [13]. The most typical example is the mutant huntingtin protein, which contains expanded polyQ tracts that disrupts specific Sp1-mediated transcription [13], [14]. This expansion leads to early deleterious consequences in the brain in the form of Huntington’s disease [14]. Moreover, polyQ tracts may stabilize protein-protein interactions [15] and function as polar zippers [16].

However, the mechanism that drives the production of amino acid tandem repeats remains unclear. One hypothesis is that the selection is unrelated to the amino acid repeats and that the different rates are merely the consequence of different protein functions and CG contents [17]. Yet, studies on the correlation between the expansion of amino acid repeats and the rate of nonsynonymous substitution support a role for selection in their expansion [5], [18], [19]. Additionally, Mularoni et al. [20] found that selection has played an important role in the evolution of amino acid repeats by increasing the repeat retention rate and modulating the repeat size.

In an analysis of the role of selection in coding CAG repeats in humans, Hancock et al. [19] proposed that slippage produces polyQ tracts and that selection and point mutations modulate their expansion. Additionally, comparative genomic studies of polyQ tracts also showed that slippage plays an important role in shaping these tracts in humans [21], [22]. Nevertheless, the role of natural selection in glutamine tandem repeat evolution is still not well understood, particularly for the repeats encoded by pure CAG codons.

Knowledge of the patterns of nucleotide substitution is important for an understanding of the dynamics of molecular evolution because mutation and selection have various effects on the synonymous *(d_S_)* and nonsynonymous *(d_N_)* substitution rates [23], [24], and the estimation of these rates provides insight into the role of selection in the evolution of polyQ tracts. In this study, we identified all of the polyQ tracts of four or more glutamine residues in the human genome using phylogenetic analysis by maximum likelihood (PAML) [25] to estimate the *d_N_*, *d_S_* and *d_N_*/*d_S_* ratios for the regions flanking the polyQ tracts and the proteins containing these tracts. The results showed that selection has played a more important role than was previously speculated in constraining the expansion of the polyQ tracts encoded by pure codons in humans.

## Materials and Methods

### Repeat Identification

Protein sequences containing polyQ tracts of four or more glutamines were downloaded from the Ensembl database (http://www.ensembl.org/human, Version 60). Only the longest protein product of each gene was used in the analysis. We developed an in-house PERL script to locate repeats in the protein sequences and mapped the protein sequences to the DNA sequences to identify their corresponding codons. We performed an alignment using the tblastn program of BLAST to locate the codons, and manually checked for proteins that did not perfectly match their corresponding cDNA sequences.

### dN/dS Calculation

Human orthologous genes in the mouse and rat genomes were downloaded from the Ensembl database, and only the longest protein/transcript of each gene was used in the analysis. The orthologous protein alignment generated by MAFFT (version 5) was transformed into a corresponding coding sequence alignment by an in-house PERL script. In our study, all of the d_N_/d_S_ calculations were performed using human-mouse-rat orthologs (Ortholog group alignment in Information S1). The average *d_N_*/*d_S_* ratios were calculated using the maximum likelihood-based program yn00 in the PAML software package [25], based on the CDS alignments. This program implements the method of Yang and Nielsen [26] to estimate *d_N_* and *d_S_* in pairwise comparisons of proteins-coding DNA sequences. Unless otherwise noted, the d_N_ and d_S_ for the regions flanking the polyQ tracts were calculated for regions of an arbitrary length of 33 codons upstream and downstream of the repeats [19], [27]. It must be noted that other polyQ tracts were excluded from estimates of the *d_N_* and *d_S_* for a region flanking a single polyQ tract. A *d_N_*/*d_S_* ratio of less than one implies purifying (stabilizing) selection. All statistical tests were performed using SPSS (Statistical Package for the Social Sciences) Statistics 17.0.

## Results

### The General Organization of PolyQ Tracts in Humans

In several disease-linked proteins, the typical repeat length of the polyQ tracts is four or longer [9]. Therefore, we searched the Ensembl database for polyQ tracts. A total of 618 polyQ tracts with 4 or more residues were identified in 454 different human proteins, including nine known neurological disease-linked proteins (Information S2).

**Figure 1:**
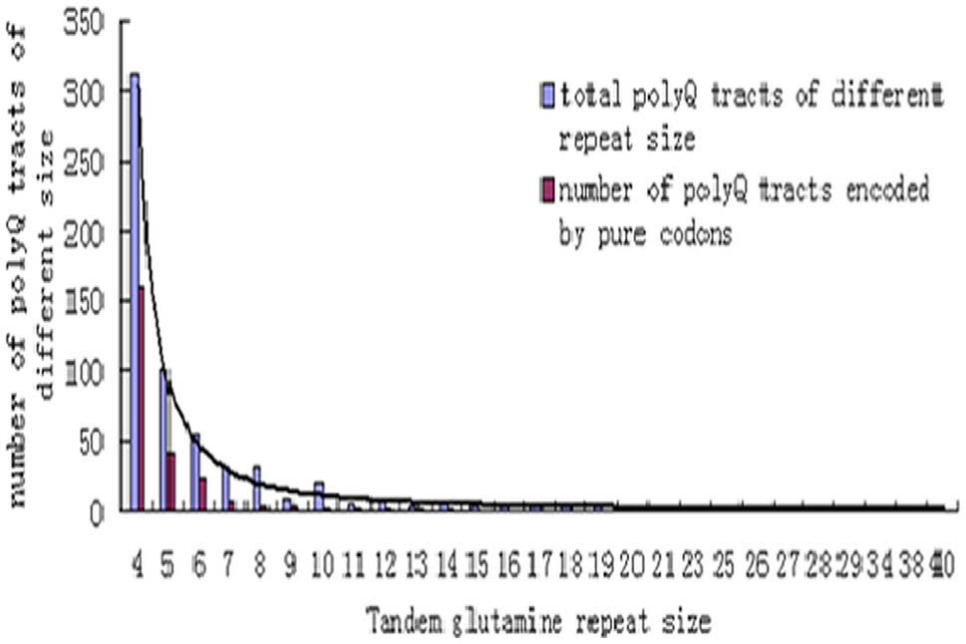
The general organization of the polyglutamine tracts in human proteins. We identified 312, 101, 126, and 89 glutamine repeats of 4, 5, 6–9, and 10–40 residues, respectively (blue column). The numbers of glutamine repeats encoded by pure CAG codons in each size bin are 160, 41, 39, and 8, respectively (red column).

Several general organizational trends were apparent across all of the tracts. First, we observed that the number of polyglutamine tracts decreased dramatically with an increase in the size of the repeats. The observed distribution roughly fits a power law curve (Figure 1). The largest human polyQ tract contains 40 tandem glutamines, whereas the largest tracts in mice and rats are 46 and 94, respectively. Secondly, at the genomic level, only a single tract of 4 glutamines was encoded by pure CAA codons, whereas the number of polyQ tracts encoded by pure CAG codons dropped sharply when the length of the polyQ tracts increased (Figure 1). Additionally, we found that the polyQ tracts were not well conserved evolutionarily. A total of 98 polyQ tracts in human proteins were absent in the mouse and rat orthologs, and only 141 polyQ tracts, accounting for 23% of the total polyQ tracts in human proteins, were fully conserved in the orthologous proteins in mice and rats.

A previous study showed that conserved polyQ tracts were encoded by mixed codons in humans [28]. Conversely, we found that a portion of the glutamine tandem repeats in human proteins that were 80–100% conserved in mice and rats were encoded by pure CAG codons. Additionally, nine neurological disorder-associated polyQ tracts [9] showed extreme variability between humans and rodents.

### Different Evolutionary Rates for the Regions Flanking the Repeats and the Proteins Containing the Repeats

**Figure 2:**
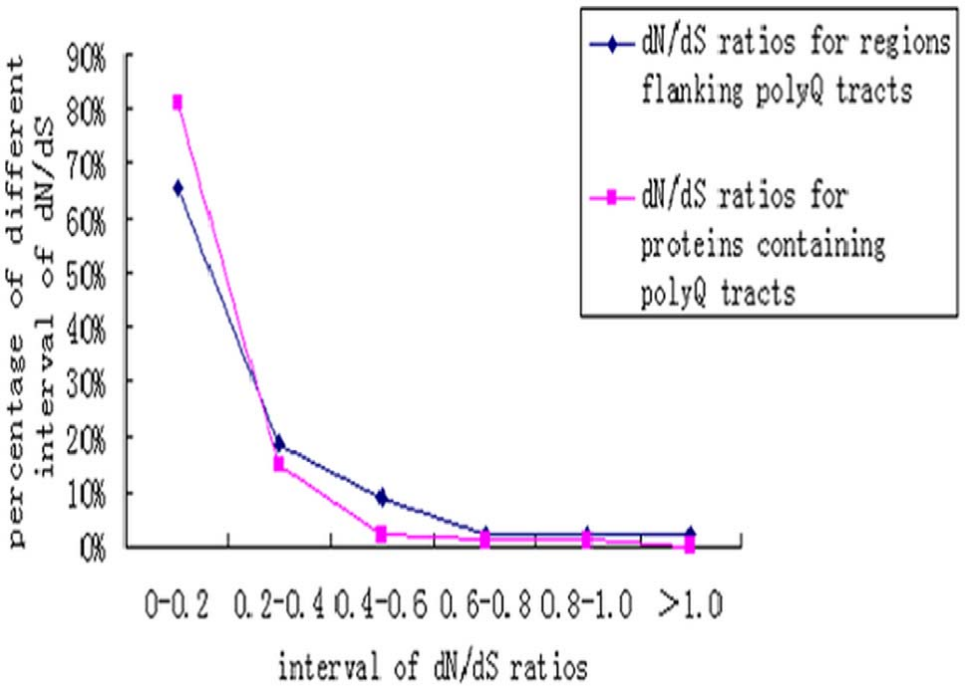
Distribution of the d_N_/*d_S_* ratios of proteins containing polyQ tracts and regions flanking the tracts. The *d_N_*/*d_S_* ratios for more than 80% of the proteins containing polyQ tracts and more than 60% of the regions flanking the tracts are under 0.2.

Several authors have reported that there are weaker selection constraints for regions flanking repeats than the remainder of the protein containing them [18], [19]. Thus, we investigated whether there was a difference in the evolutionary rates between the regions flanking the tracts of four or more glutamines and the proteins containing them in humans. In this case, the *d_N_* or *d_S_* for the entire protein included the repeat, and the *d_N_* or *d_S_* for a region flanking the repeat excluded the repeat. The *d_N_*/*d_S_* ratio was taken as an indicator of the strength of purifying selection. We calculated the *d_N_*/*d_S_* ratios for 392 proteins containing polyQ tracts and 480 regions flanking the tracts (Information S3). The average *d_N_*/*d_S_* ratio of all of the proteins tested was 0.139. Most of the *d_N_*/*d_S_* ratios were less than 0.90, but one protein, C9orf144B, had a *d_N_*/*d_S_* ratio greater than 1.0 (Figure 2, pink line). These results indicated that most proteins containing polyQ tracts had undergone purifying selection.

As shown in Figure 2 (blue line), the *d_N_*/*d_S_* ratios for 98% of the regions flanking the tracts were less than 1.0, which indicates that most of the regions had also undergone purifying selection. However, the average *d_N_*/*d_S_* ratio for the regions flanking the tracts was significantly higher than that for the proteins containing the tracts (P<0.001, two-tailed Mann-Whitney *U*-test), presumably reflecting a weaker purifying selection for the regions flanking the repeats.

**Figure 3:**
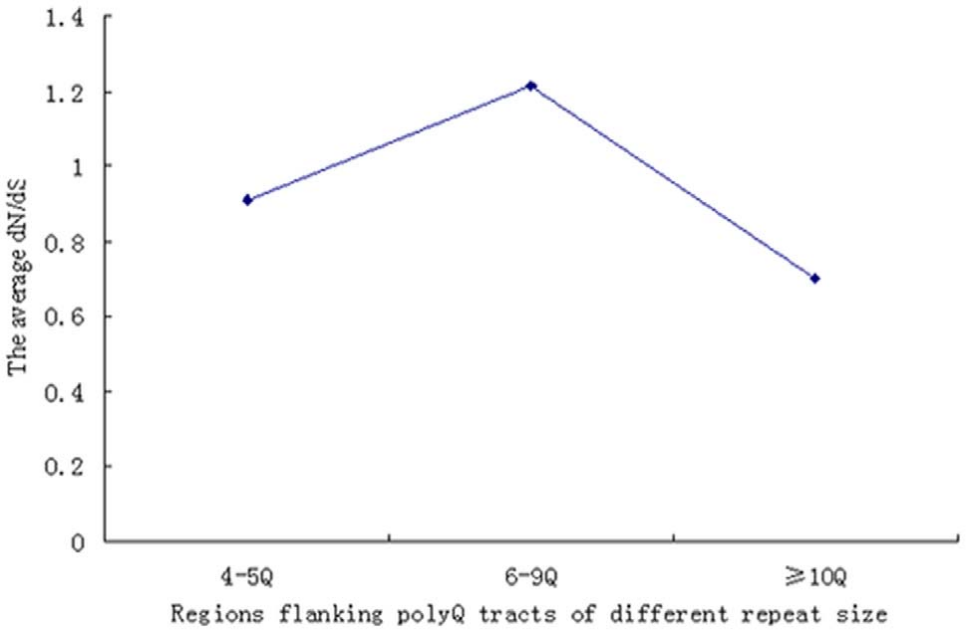
The average *d_N_*/*d_S_* ratio for the regions flanking polyQ tracts of different repeat sizes. The tracts were divided into three different groups. The first group of tracts included tracts of 4 or 5 glutamines, the second group consisted of tracts of 6–9 glutamines, and the third group comprised tracts of 10 or more glutamines.

To understand the relationship between the *d_N_*/*d_S_* ratio and polyQ tract length, we compared the *d_N_*/*d_S_* ratios for the regions flanking the polyQ tracts of different repeat sizes. The tracts were divided into three different groups. The first group of tracts included tracts of 4 or 5 glutamines, the second group consisted of tracts of 6–9 glutamines, and the third group comprised tracts of 10 or more glutamines. The average *d_N_*/*d_S_* ratio for the regions flanking the first group of tracts was lower than that for the regions near the second group and higher than that for the regions near the third group, but the average *d_N_*/*d_S_* ratio for first and second, third groups exhibited no significant difference (Figure 3). These results indicated that there is no positive correlation between the *d_N_*/*d_S_* ratios of the regions flanking the polyQ tracts and the tract length.

Additionally, we found that the average *d_N_*/*d_S_* ratio for the C-terminal regions flanking the polyQ tracts is significantly higher than that for the N-terminal flanking regions (P = 0.001, two-tailed Mann-Whitney U-test) (Information S4), reflecting greater selection constraints on the N-terminal flanking regions.

### Highly Variable Versus Highly Conserved Repeats

The analysis of the repeat size variability between humans and rodents had shown a low degree of conservation of the repeats in orthologous proteins. Therefore, we addressed whether there was any association between repeat conservation and the evolutionary rate. We investigated the difference in the evolutionary rates between two groups of proteins containing either highly conserved or highly variable polyQ tracts by estimating the *d_N_* and *d_S_* ratios for these two groups of proteins.

The highly conserved (HC) group included human proteins containing glutamine tandem repeats that were 80–100% conserved in their mouse and rat orthologs. In contrast, the highly variable (HV) group comprised human proteins containing glutamine tandem repeats that were 0–40% conserved in their orthologous mouse and rat proteins. We estimated the *d_N_*/*d_S_* ratios for the HC and HV proteins and found that the average *d_N_*/*d_S_* for the HC group was significantly lower than that for the HV group (Table 1) (P<0.001, two-tailed Mann-Whitney *U*-test). In the HV group, the average *d_N_*/*d_S_* ratio for the regions flanking the repeats was significantly higher than that in the HC group (Table 2) (P<0.001, two-tailed Mann-Whitney *U*-test). These results reflected a weaker purifying selection for the regions near the repeats in the HV group. Additionally, eight proteins encoded by genes that are associated with neurodegenerative diseases in humans were included in the HV group.

**Table 1:** Mean *dN/dS* ratios for the protein groups.

Group	Number	*d_N_/d_S_*
All proteins containing polyQ tracts of four or more glutamines	392*	0.139±0.007
Proteins containing highly conserved poly-Q tracts	137	0.093±0.008
Proteins containing highly variable poly-Q tracts	211	0.176±0.012
Proteins containing polyQ tracts encoded by pure codons	142	0.108±0.009
Proteins containing polyQ tracts encoded by mixed codons	250	0.156±0.010

* 454 proteins contain poly-Q tracts of four or more glutamines in humans. However, this number excludes 41 human proteins without assigned homologs in either mouse or rat, which served as the references for estimating the *dN/dS* ratios. Additionally, the *dN/dS* ratios for 21 human proteins were not available by PAML estimation. The *dN/dS* ratios were estimated for the entire protein, including the repeat.

**Table 2:** Mean *dN/dS* ratios for region groups.

Group	Number	*d_N_/d_S_*
All regions flanking polyQ tracts of four or more glutamines	480*	0.943±0.165
Regions flanking highly conserved polyQ tracts	142	0.456±0.128
Regions flanking highly variable polyQ tracts	271	1.107±0.194
Regions flanking polyQ tracts encoded by pure codons	195	0.803±0.191
Regions flanking polyQ tracts encoded by mixed codons	285	1.035±0.246

* 454 proteins contain poly-Q tracts of four or more glutamines in humans. However, this number excludes 41 human proteins without assigned homologs in either mouse or rat, which served as the references for estimating the *dN/dS* ratios. Additionally, the *dN/dS* ratios for some regions flanking the poly-Q tracts were not available by PAML estimation. The *dN/dS* ratios were calculated for a region including 33 amino acids on either side of the repeat and excluding the repeat.

### Lower Average *d_N_*/*d_S_* Ratios were Observed for the PolyQ Tracts Encoded by Pure CAG Codons than for the PolyQ Tracts Encoded by Mixed Codons

To understand the role of natural selection in the evolution of polyQ tracts, it is crucial to investigate whether a difference in selection constraints exists between the polyQ tracts encoded by pure CAG codons and those encoded by mixed codons; tandem CAG repeats are prone to slippage, whereas interrupted structures are less likely to undergo slippage [21]. By estimating the *d_N_* and *d_S_*, we found that the average *d_N_*/*d_S_* ratio for the proteins containing polyQ tracts encoded by pure CAG codons was significantly lower than that for the proteins containing polyQ tracts encoded by mixed codons (Table 1) (P<0.001, two-tailed Mann-Whitney *U*-test). This result indicated a stronger purifying selection for the proteins containing polyQ tracts encoded by pure CAG codons.

Surprisingly, these results showed that the average *d_N_*/*d_S_* ratio for the regions flanking polyQ tracts encoded by mixed codons was significantly higher than that for the regions flanking polyQ tracts encoded by pure CAG codons (Information S5,Table 2) (P = 0.011, two-tailed Mann-Whitney *U*-test). This result indicated there was a higher level of purifying selection for the regions flanking polyQ tracts encoded by pure CAG codons compared with those encoded by mixed codons.

**Figure 4:**
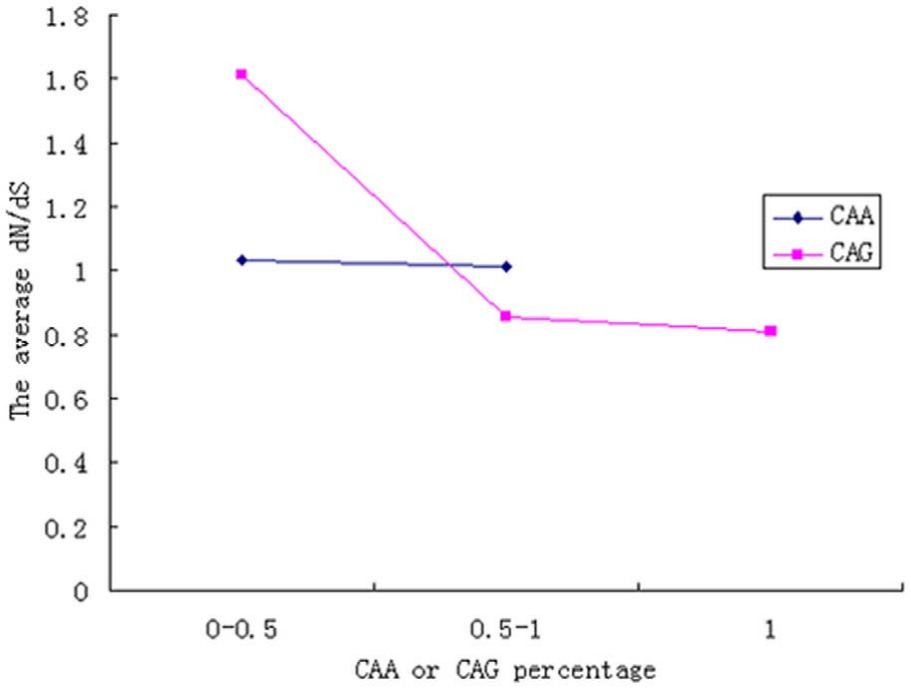
The average *d_N_*/*d_S_* ratio for the regions flanking polyQ tracts with different percentages of CAG or CAA in the codons. The labels 0–0.5, 0.5–1 and 1 indicate less than or equal to 50%, more than 50% but less than 100%, and 100% of CAG or CAA codons, respectively. Only one tract of 4 glutamines was encoded by pure CAA codons, so only two groups were encoded by more than 50% and by less than or equal to 50% CAA.

To further investigate whether the *d_N_*/*d_S_* ratio for the regions flanking polyQ tracts is correlated with the percentage of CAG codons, the tracts were divided into three different groups based on their percentage of CAG codons. The first group of tracts consisted of pure CAG codons, the second group consisted of more than 50% CAG codons, and the third group was consisted of less than or equal to 50% CAG codons. The results indicated that the average *d_N_*/*d_S_* ratio for the regions flanking the first and second groups was significantly lower than that for the regions near the third group; however, the average *d_N_*/*d_S_* ratio for first and second groups exhibited no significant difference (Figure 4). Additionally, the conserved polyQ tracts contained some members of the first group and a large proportion of the second group but no representatives from the third group.

**Figure 5:**
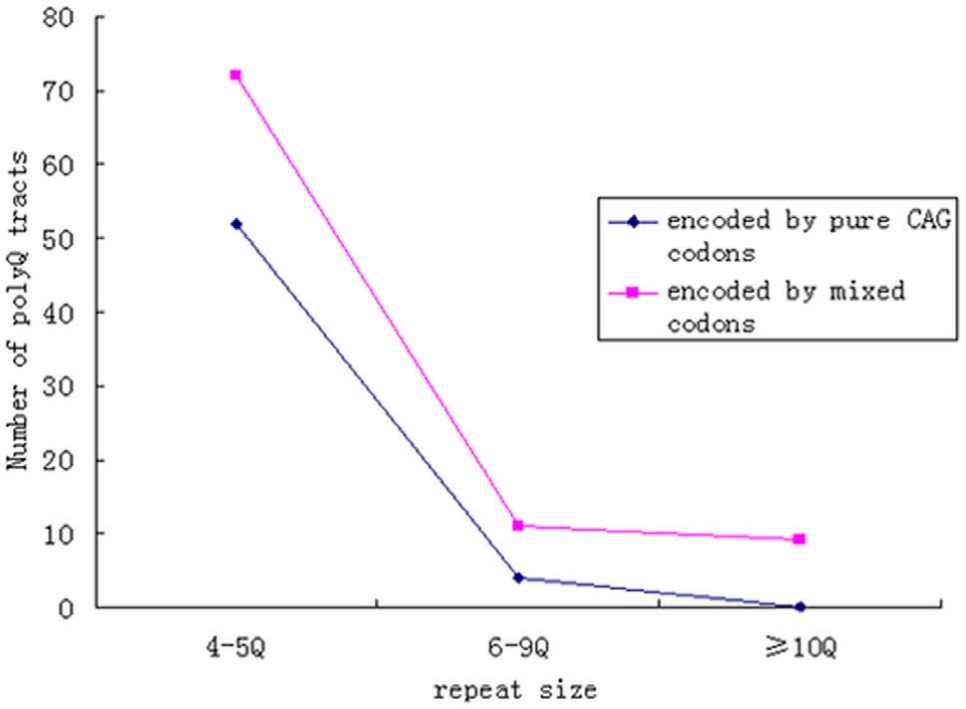
Highly conserved polyQ tracts encoded by pure or mixed codons. A large number of 4–5Q tracts encoded by pure codons are highly conserved, but conserved polyQ tracts of 10 or more glutamines are all encoded by mixed codons.

To exclude the possibility that CAA richness is associated with a high *d_N_*/*d_S_* ratio, we investigated the relationship between the percentage of CAA codons and the *d_N_*/*d_S_* ratio. The result showed that there was no significant difference between the average *d_N_*/*d_S_* ratio for the regions flanking the tracts encoded by more than 50% CAA codons and that for the tracts encoded by less than or equal to 50% CAA codons (Figure 4). This finding excluded the possibility that CAA richness is the factor that is associated with the high *d_N_*/*d_S_* ratio for the regions flanking the tracts in the third group.

## Discussion

In this study, we found that most of the human proteins containing polyQ tracts of four or more glutamines have undergone purifying selection (Figure 2). The fact that the *d_N_*/*d_S_* ratio for the regions flanking the tracts is higher than that for the proteins containing them suggests that the regions flanking the tracts evolve more quickly than the remainder of the protein containing them, consistent with the findings of previous studies [5], [19], [27]. The weak purifying selection observed for the regions near the repeats may imply the action of slippage in the repeats.

Further analysis reveals that there is a weaker purifying selection for the regions near the nonconserved polyQ tracts than for the regions flanking the conserved tracts. In other words, the regions flanking the conserved repeats are under stronger selection constraints than the regions flanking the nonconserved repeats, which is consistent with some previous studies [18], [19]. These data suggest that the nonconserved tracts lie in regions that experience lower purifying selection.

Interestingly, we found dramatic differences between the polyQ tracts encoded by pure CAG and mixed codons. Our results showed stronger selection constraints for the proteins containing the polyQ tracts encoded by pure CAG codons. Furthermore, our analysis indicated that the average *d_N_*/*d_S_* ratio for the regions flanking the polyQ tracts encoded by mixed codons is significantly higher than that for the regions flanking the polyQ tracts encoded by pure CAG codons, suggesting that the regions near impure CAG codons evolve faster than the regions near pure CAG codons. However, a previous study showed that conserved polyglutamine tracts usually are encoded by mixed codons, whereas rapidly evolving tracts are encoded primarily by pure CAG codons [28]. A comparative analysis showed that proteins containing conserved repeats had lower evolutionary rates than did proteins with nonconserved repeats [18], [19].

The discrepancy between the previous and present studies could be due to the different data sets used. We identified all of the polyQ tracts of four or more glutamines in humans and then estimated the *d_N_*/*d_S_* ratios for the regions flanking the polyQ tracts and the proteins containing them to investigate the molecular evolution of the tracts. In previous studies, however, short tracts of 4 or 5 glutamines were excluded from the analyses [18], [19], [28]. We found that the normal repeat length of the polyQ tracts in several disease-linked proteins is four or longer [9], indicating that tracts of 4 or 5 repeats in length should be included in a comprehensive analysis. However, the polyQ tracts of 4 or 5 residues contain a large amount of the conserved tracts and tracts encoded by pure codons, which would result in overlap with regard to the relative proportion of each. In other words, a large number of the 4–5Q tracts encoded by pure codons are conserved (Figure 5), which may result in a bias between the previous and present analyses.

Additionally, a previous study found that polyQ tracts are associated with specific sequence biases [29]. To exclude the possibility that other amino acid repeats in the regions flanking the polyQ tracts introduced biases in our analysis, we identified all of the amino acid repeats of 4 residues or longer in the regions flanking the pure CAG- and mixed codon-encoded polyQ tracts and then analyzed their *d_N_*/*d_S_* ratios (Information S6). We found 23 and 41 amino acid repeats of 4 residues or longer in these regions, respectively, and determined that most of the repeats are located in the C-terminal regions flanking the polyQ tracts. Our analysis indicated that the average *d_N_*/*d_S_* ratio for the regions flanking the polyQ tracts encoded by mixed codons is also significantly higher than that for the regions flanking the polyQ tracts encoded by pure CAG codons (P = 0.009, two-tailed Mann-Whitney U-test) when the regions containing other amino acid repeats were excluded. This result indicated that the presence of other amino acid repeats in the regions flanking the polyQ tracts do not negatively affect our analysis.

Polyglutamine tracts are the most intensively studied type of amino acid tracts because expansions of these tracts in several human proteins can cause neurological disorders [9], [11], [12]. The largest class of neurological diseases results from the expansion of CAG repeats within exons. We found that there is a stronger purifying selection for the regions flanking the pure repeats and the protein containing them. We also observed that most of the longer polyQ tracts are encoded by mixed codons (Figure 1). Thus, our results suggest that selection has played a more important role than was previously speculated in constraining the expansion of the tracts encoded by pure CAG codons. This finding could help explain why only a few proteins containing polyQ tracts encoded by pure CAG codons massively expand to result in human disorders.

In conclusion, by estimating the synonymous and nonsynonymous rates of evolution for the regions flanking the tracts and the proteins containing them, our observations support the hypothesis that selection is involved in the evolution of polyQ tracts by analyzing the divergent levels of purifying selection experienced by different groups of repeats.

## Supporting Information

Information S1 **Ortholog Group Alignment in Phylip format.**
(DOC)

Information S2 **A total 618 ployQ tracts of four or more glutamines in huamn proteins.**
(XLS)

Information S3 ***d_N_*** **and** ***d_S_*** **rates for all regions flanking ployQ tracts and for all proteins containing them.**
(XLS)

Information S4 ***d_N_*** **and** ***d_S_*** **rates for polyQ tracts’ N and C-terminal flanking regions.**
(XLS)

Information S5 ***d_N_*** **and** ***d_S_*** **rates for regions flanking polyQ tracts encoded by pure CAG and mixed codons.**
(XLS)

Information S6 ***d_N_*** **and** ***d_S_*** **rates for regions in which there are other amino acid repeats flanking polyQ tracts encoded by pure CAG and mixed codons.**
(XLS)
