# Supplementary material for: Insight into Role of Selection in the Evolution of Polyglutamine Tracts in Humans
Source: PLoS One. 2012 Jul 25;7(7):e41167. doi: 10.1371/journal.pone.0041167 (PMC3405088; doi:10.1371/journal.pone.0041167)
Supplement: Information S1 — Ortholog Group Alignment in Phylip format. (DOC) [file pone.0041167.s001.doc]

>Ortholog Group 100, Repeat 1

3 198

H00343948 GCGTCACGCA TAGGGTTGCG CATGCAGCTC ATGCGGGAGC AGGCGCAGCA

M00024786 GCGTCACGCA TCGGGCTGCG CATGCAGCTC ATGCGGGAGC AGGCCCAGCA

R00037517 GCGTCGCGCA TCGGGCTGCG CATGCAGCTC ATGCGGGAGC AGGCGCAGCA

GGAGGAGCAG CGGGAGCGCA TGCAGCAACA GGCTGTCATG CATTACATGC

GGAGGAGCAG CGAGAGCGCA TGCAGCAGCA GGCTGTCATG CATTATATGC

GGAGGAGCAG CGAGAGCGCA TGCAGCAGCA GGCCGTCATG CATTACATGC

TCGGAGGGCC GCCCACCCCG GCCATCAATA CCCCCGTCCA CTTCCAGTCG

TGGGTGGGCC CCCCACCCCA GCCATCAACA CCCCTGTCCA CTTCCAGTCG

TGGGAGGCCC TCCCACCCCA GCCATCAACA CCCCAGTTCA CTTCCAGTCG

CCACCACCTG TGCCTGGGGA GGTGTTGAAG GTGCAGTCCT ACCTGGAG

CCCCCGCCTG TGCCCGGGGA GGTGCTGAAG GTGCAGTCCT ACCTGGAG

CCACCACCAG TGCCTGGGGA GGTGCTGAAG GTGCAGTCCT ACCTGGAG

>Ortholog Group 101, Repeat 1

3 198

M00124317 ATTCCCATCT TTAGTCCAAT GATGCCTTAC GGCACAGGAC TTACTCCACA

R00002038 ATTCCCATCT TCAGTCCAAT GATGCCTTAC GGCACAGGGC TTACTCCACA

H00230354 ATCCCTATCT TTAGTCCAAT GATGCCTTAT GGCACTGGAC TGACCCCACA

GCCTATTCAG AACACCAACA GTCTCTCTAT TTTGGAAGAG CAACAAAGAG

GCCTGTTCAG AACACCAATA GTTTATCTAT TTTGGAAGAG CAACAAAGAG

GCCTATTCAG AACACCAATA GTCTGTCTAT TTTGGAAGAG CAACAAAGGG

CAACTGCAGC AGCCTCAGTA CAGCAATCAA CATCTCAGCA ACCCACACAG

CAACTGCAGC AGCCTCAGTA CAGCAATCAA CATCTCAGCA GCCCACACAG

CAGTGGCAGC TGCAGCCGTT CAGCAGTCAA CGTCCCAGCA GGCAACACAG

GGTGCCTCAG GCCAGACCCC ACAACTCTTC CATTCTCAAA CTCTGACC

GGTGCCTCAG GCCAGACCCC ACAACTCTTC CATTCTCAAA CTCTAACC

GGAACCTCAG GCCAGGCACC ACAGCTCTTC CACTCACAGA CTCTCACA

>Ortholog Group 102, Repeat 1

3 198

M00064839 TTGGATGATT ACATGAAATG TTTTAAGACT CCAGTTGTAA AGAATGACTT

R00047739 TTGGATGATT ACATGAAATG TTTTAAGACT TCAGTTGTAA AGAATGAATT

H00358813 TTGGATGATT ACATGAGCTG TTTTAGAACT CCAGTTGTAA AGAATGACTT

TCCACCTGCC TGTCCATCAT CAACACCTTA CAGCCAGCTT GCCCGCCTCC

CCCACCTGCC TGTCCATCAT CAACACCTTA CAGCCAACTT GCCCGCCACC

TCCACCTGCT TGTCAGTTGT CAACACCTTA TGGCCAACCT GCCTGTTTCC

AGCAGGGACT CAGCACTCCT CTTCAAAGCT TGCAGATTTC AGGTTCTTCA

AGCAAGGGCT CAGCACTCCT CTTCAAAACT TACAGATTTC AGGATCCTCA

ATCAAATACT TGCCACTCCA CTTCAAAATT TACAGGTTTT AGCATCTTCT

TCAATAAATG AATGCATTTC AGTTAACGGA AGAATTTATT CCATATTA

TCAATGAGTG AATGCATTTC AGTTAACGGA AGAATTTATT CCATATTA

TCAGCAAATG AATGCATTTC GGTTAAAGGA AGAATTTATT CCATATTA

>Ortholog Group 103, Repeat 1

3 198

R00020780 CAAGAGTTCA CCGCAGCGCG GGACGGTTCA GCACTTGAGA AGGAAGGAGT

M00037788 CAAGAGTTCA CAGCAACTCG GGACAGTTCA GCACTTGAAA AGGAAGGAAT

H00264935 GAAGAATTCA CAGCAGCTCA GGACAGCTCT GCGATGGTGG GTGAAGATGT

TGGGTACCTG TCTCTGGAGA ACAAGGCTCT GCAAAGCCGC CTTACGGAGT

TGGTTACCTA TCTCTTGAAA ATAAGACTCT GCAAAGCCGC CTTGCTGAGT

CGGCTCCCTG GCTCTGGAGA GTAAGTCCCT GCAAAGCCGC CTTGCTGAGC

ATGCTGCTGC GGTGACCAAG GTGACGACAG AGCTCAACAA CACAAAGAGG

ATACTGCTAC AGTGACCAAG ATGACAGCAG AGCTCAATAA TACAAAGAGG

ACGCCCGGGA GATGAGCGAG GTGACGGCGG AGCTGCACCA CACACACAAG

GAGCTGGACA CATTGAGACA ACATTTGGAC AACTCATTGG AAGAGAAT

GAGCTGGACA CATTGAGACA ACATTTGGAC AAATCATTGG AAGAGAAT

GAGCTGGATG ATTTGAGACA ACATTTAGAT AAATCTTTGG AAGAGAAC

>Ortholog Group 104, Repeat 1

3 198

H00231524 GCAGCCTGCC TCCACTGTGA AAAGACTTTG CAGCAGGATG ATTGTAGAGT

R00016647 ACAGCCTGTC TCCATTGT-- -AAGACTTTG CAGCAGGATG ATTGCAGAGT

M00022225 ACAGCCTGTC TCCACTGTGA AAAAACTCTG CAGCAGGATG ATTGCAGAGT

TGTCTTGGCA AAACAGGAAA TTACAAGGTT ACTGGAAACA TTGCAGAAAT

TGTCTTGGCA AAACAAGAAA TCACAAGATT ATTAGAAACA CTGCAGAAAT

TGTCTTGGCA AAACAAGAAA TCACAAGATT ACTAGAAACA CTGCAGAAAT

TTACAGAAGT TGCAGATCAC ATTCAGTTGG ATGCCAGCAT CCCTGTCACT

TTACAGAAGT TGCAGATCAT ATTCAGTTGG ATGCCAGTAT TCCAGTCACT

TTACAGAGGT TGCAGATCAT ATTCAGTTGG ATGCCAGTAT TCCAGTCACT

TTTACAAAGG ATAATCGAGT TCACATTGGA CCAAAAATGG AAATTCGG

TTTACAAAGG ACAACAGAGT TCATATTGGA CCCAAAATGG AAATCCGA

TTTACAAAGG ACAATAGAGT TCATATTGGA CCCAAAATGG AAATCCGA

>Ortholog Group 105, Repeat 1

3 207

H00231656 AAATCAGAGC TGGCTGCCAA TCTGGGGCTC ACTGAACGGC AGGTGAAGAT

M00025521 AAGTCCGAGC TGGCTGCTAA CCTGGGGCTC ACAGAGCGGC AGGTAAAGAT

R00025141 AAGTCAGAGC TGGCTGCTAA CTTGGGTCTC ACAGAGCGGC AGGTAAAGAT

CTGGTTCCAA AACCGGCGGG CAAAGGAGCG CAAAGTGAAC AAGAAGAAAC

CTGGTTCCAG AACCGCCGGG CCAAGGAGCG CAAAGTAAAC AAGAAGAAAC

CTGGTTCCAG AACCGTCGGG CCAAGGAGCG TAAAGTAAAC AAGAAGAAAC

CCCCACAGCC G--------- CCGATGGCCC ACGACATCAC GGCCACCCCA

CCCTGCCTCC CACACAGCTG CCCCTGCCCC TGGATGGCAC TCCCACACCA

CCATGCCTCC CACACAGTTG CCCCTGCCCC TGGATGGCAC CCCCACACCA

GCCGGGCCAT CCCTGGGGGG CCTGTGTCCC AGCAACACCA GCCTCCTGGC

TCAGGGCCAC CCCTAGGAAG TCTATGCCCT ACTAATGCTG GCCTTCTGGG

TCGGGGCCAC CCCTGGGGAG TCTATGCCCC ACCAATGCTG GTCTTCTGGG

CACCTCC

CACCCCC

CACCCCC

>Ortholog Group 106, Repeat 1

3 198

H00424765 ACTGCTGTCA CTGACCCACG GTACCGTGCC CGCGGCAGCC CGCACTTCCA

R00058797 GCGGCTGTCA ATGACCCAAG ATACCGTCCG AGAAGCAGCC CACACTTCCA

M00121113 GCGGCTGTCA CTGACCCAAG ATACCGACCC CGAAGCAGCC CACACTTCCA

GCATGCTGAA GTCAGGATCC TGCAGGCCCA GGTGCCTCCT GTGTTCCTCT

GCATGCTGAA GTCAGGATCC TGCAGGCACA GGTACCACCG GTTTTCCTCT

GCATGCCGAA GTCAGGATCC TGCAGGCCCA GGTACCACCG GTGTTCCTCT

ACCAGTACCT GCAGCAATCT CAGGAGCACC CCCCTCCCCC ACATCCAGCT

ACCAGTACCT GCAGCAACCC CAGGAGCACT CCCCACCCCT CCATCCAGCC

ACCAGTACCT GCCACAGCCC CAGGAGCACT CTCCACCCCT CCACCCGGCA

GCTCTCGGCC ATGGCCCCCT GAGCTCCCTC AGTCCACCTG CTGTGGAG

GCTCTAGGCC ATGGTCCCCC AAGTTCCTTC AGTCCACCTG CCCTGGAG

GCTCTGGGCC ATGGACCCCC AAGCTCCTTT GGTCCACCTG CAGTGGAG

>Ortholog Group 107, Repeat 1

3 177

M00108954 CAGCAGCAGC AGCAACAGCA GCAACAGCAG ---------- ----------

H00420736 TACAGCAAGA AGACGGAAAT CCAAAGGCAG ACAGTACGGG CTCCCTTCGC

R00013271 CAGCAGCAGC AGCAACAGCA GCAGCAGCAG ---------- ----------

---------- ---------- -----CAGCA ACAACAGCAG CAGCAGCAAG

CAAACTCTTC ATTTTCTCTG CACTTCAGGT GGCAAGACAG CTCCTTCTTG

---------- ---------- -----CAGCA GCAGCAGCAG CAACAGCAGG

TTAGTGGATT AAAGTCTCCC AAGAGGAATG ACAAGCAACC AGCTCTTCAG

TTAGTGGATT AAAATCTCCC AAGAGGAATG ACAAACAACC AGCTCTTCAG

TTAGTGGATT AAAGTCTCCG AAGAGGAATG ACAAGCAACC AGCTCTTCAG

GTTCCCGTGT CAGTGGCTAT GATGACA

GTTCCCGTGT CAGTGGCTAT GATGACA

GTTCCCGTGT CAGTGGCTAT GATGACA

>Ortholog Group 107, Repeat 2

3 84

M00108954 CAGGTTATCA CTCCTCAACA AATGCAGCAG ATCCTCCAGC AGCAGGTGCT

H00420736 CAAGTTATCA CTCCCCAGCA AATGCAGCAG ATCCTCCAGC AACAAGTGCT

R00013271 CAGGTTATCA CTCCTCAACA AATGCAGCAG ATCCTCCAGC AGCAAGTGCT

GAGCCCCCAG CAGCTCCAGG TTCTCCTCGC CCTC

GAGCCCTCAG CAGCTCCAGG TTCTCCTCGC CCTC

GAGCCCACAG CAGCTCCAGG TTCTCCTCGC CCTC

>Ortholog Group 107, Repeat 3

3 111

M00108954 ATGCTTCTTC AAGAATTTTA TAAAAAACAA CAGGAACAGT TGCAGCTTCA

H00420736 ATGCTTCTTC AAGAGTTTTA TAAAAAACAA CAGGAACAGT TGCAGCTTCA

R00013271 ATGCTTCTTC AAGAATTTTA CAAGAAACAA CAGGAACAGT TGCAGCTTCA

ACTTCTCCAA CAGCAACATG CTGGAAAACA GCCGAAAGAG ------CAGC

ACTTTTACAA CAACAACATG CTGGAAAACA GCCTAAAGAG ------CAAC

ACTCCTCCAA CAGCAACATG CTGGAAAACA GCCGAAAGAG CAGCAGCAGC

AGCAGGTGGC T

AGCAGGTGGC T

AGCAGGTGGC T

>Ortholog Group 108, Repeat 1

3 198

R00011668 TACCCAGACA TCTTCATGCG CGAGGAGGTG GCTCTCAAGA TCAACCTGCC

H00282549 TACCCTGACA TCTTCATGCG GGAGGAGGTG GCGCTCAAGA TCAACCTGCC

M00006071 TACCCAGACA TCTTCATGCG CGAGGAGGTG GCACTCAAGA TCAACCTGCC

CGAGTCCAGA GTCCAGGTTT GGTTCAAGAA CCGCCGAGCC AAGTGCCGCA

GGAGTCTAGA GTCCAGGTCT GGTTCAAGAA CCGCCGCGCC AAATGCCGCA

AGAGTCCAGA GTCCAGGTTT GGTTCAAGAA CCGCCGCGCC AAGTGCCGCA

GCGGGAATGG AACGAAAAGC CGGCCGGTCA AGAAGAAGTC GTCTCCGGTG

GCGGGAGCGG AACCAAGAGC CGCCCAGCCA AGAAGAAGTC CTCTCCAGTG

GCGGGAATGG AACGAAAACC CGGCCGGTCA AGAAGAAGTC GTCTCCAGTA

CGCGAGAGCT CGGGTTCCGA GAGCAGCGGC CAGTTCACGC CGCCCGCC

CGGGAGAGCT CGGGCTCCGA AAGCAGTGGC CAATTCACGC CGCCAGCT

CGCGAGAGCT CGGGTTCAGA GAGCAGCGGC CAGTTCACGC CGCCCGCC

>Ortholog Group 109, Repeat 1

3 132

H00386456 GCTCCTCCCA CTCCACAGCA GACGCCTTCT ACTCAGGCCC AGGGTCTGCC

M00086948 GCTCCACCCA CCCCACAGCA GACACCTGCT ACCCAGACAC AAGGTTTGCC

R00044665 GCTCCACCCA CCCCACAGCA GACGCCTGCT CCCCAGACCC AGGGTCTGCC

CGCTCAGGCC CAGGCCACAC CCCAGCACCA GCAGCAACTC TTCCTCAAG-

CACCCAGGCC CAGGCCACTC CCCAGCACCA GCAGCAACAC CTCCTCAAG-

CACCCAGGCC CAGGCTACTC CTCAGCACCA GCAGCAGCTC CTCCTCAAGC

---------- ----CCACCG CCAGCACAGC AG

---------- ----CAGCCT ACAGCACCAC CA

AGCAGCAGCA GCAGCAGCCA ACAGCACCAC CA

>Ortholog Group 109, Repeat 2

3 93

H00386456 CCGGCAGGCA CGTTTTAC-- ---------- ------GCCC AGACTCAGCA

M00086948 CCTGCAGGCA CCTTTTACCA GCAGCAGCAG CAACAGGCTC AGACTCAGCA

R00044665 CCATCAGGCA CCTTTTAC-- -CAGCAGCAG CAGCCCGCTC AGACTCAGCA

GTTTCAGGCA GTACATCCAG CAACCCAGAA ACCAGCAATT GCT

GTTTCAGGCA GTACATCCAG CAGCCCAGCA ACCAGTCACT GCT

GTTTCAGGCA GTGCATCCAG CAGCCCAGCA ATCAGTCACT GCT

>Ortholog Group 109, Repeat 3

3 156

H00386456 CAGTTCCCTG TGGTGTCCCA AGGAGGCTCT CAACAGCAGC TAATGCAGAA

M00086948 CAGTTCCCTG TGGGGTCCCA GGGAGGTGCT CAGCAACAGC TGATGCAGAA

R00044665 CAGTTCCCTG TGGTGTCCCA GGGAGGCTCT CAGCAACAGC TGATGCAGAA

TTTCTACCTG GCCACAGCCC TGCATCAACA ACAGCTGATG ACTCAGCAGG

CTTCTAC--- ---------- -----CAGCA GCAGCTGATG GCTCAGCAGG

CTTCTAC--- ---------- -----CAGCA ACAGCTGATG GCTCAGCAGG

CTGCCTTGCA GCAAAAGCCC ACTATGGCAG CAGGACAGCA GCCCCAGCCA

CTGCCCTGCA GCAGAAG--- ACTGCTGTGG TAGTACCACA GTCTCAGGCA

CCGCCCTGCA GCAGAAG--- ACTGCTGTGG TAGTACCACA GCCTCAGGCA

CAGCCA

CAGCCC

CAGCCA

>Ortholog Group 10, Repeat 1

3 207

R00022939 ---------- ---------- ---------- ---------- ----------

H00356116 CACCAGCAGC ACCACCACCA CCACCATGCC CACCACCACC ACCACCATGC

M00090398 CATCAGCAGC ACCACCACCA CCATGCCCAC CACCACCACC ACCACCATGC

---------- ---------- ---------- ---------- ----------

C--------- CACCACCTCC ACCACCACCA CGCACTACAG CAGCAGCTAA

CCACCACCTC CACCACCTCC ACCACCACCA CGCACTACAG CAGCAGCTAA

---------- ---------- ---------- ---------- ----------

ACCAGTTCCA TCCCATTTCC AACAACAACA GCTTGGGCGG CGCGGGCGGC

ACCAGTTCCA TCCCACTGCC ---AACAACA GCCTGGGCGG TGCGGGCGGC

---------- ---------- ---------- ---------- ----------

GGCGCGCCTC AGCCCGGCCC CGACATGGAG CAGCCGCAAC ATGGAGGCGC

GGCGCGCCTC AGCCCGGCCC GGACATGGAG CAGCCGCAAC ATGGAGGCGC

-------

CAAGGAC

CAAGGAC

>Ortholog Group 110, Repeat 1

3 207

R00017064 CACACGAGCC AG-------- ---------- ---------- --------GG

H00234389 CACACCAGCC AGAAAATCCA CCGCGCCCTC AACACGGAGC CACCAGAGGG

M00048576 CACACGAGCC AGAAGATCCA CCGAGCCCTC AACACAGGGC CACCAGAGGG

G---CAACAG GAGAGGGCAG AGCACGAGCG CAGCGGCCCC ---------G

GTCGAAGGAG GAGACGGCAG AGGCGGAGCC CAGCGGCCCC GAGGTGGAGG

G---CAACAG GAGAGGGCAG AGCAGGAGTG CAGCGGCCCC ---------G

AGCTGCCTGC CACCGATGGT GCAGGGCGCT GGAGGCGGGT GCGCCGGGCT

ACCAGCCAAC GGCTCCGGAG GGC------T GGAAACGGGC GCGCCGGGCC

AGCAACCTGC AGCCGACGGT GCGGGGCGCT GGAGGCGGGT GCGCCGGGCC

---GTGGAAC GGGAGCGACG CGTGCGTTTC CTGCTGGAAC CTGGG-----

---GTGGACA AGGAGCGCCG CGTGCGCTTC CTGCTGGAGC CCGCCGTGGT

GTGGTGGAAC GGGAACGGCG CGTGCGTTTC CTGCTGGAAC CTGGG-----

-------

TGTGGCA

-------

>Ortholog Group 111, Repeat 1

3 198

H00365007 ATTCAAATCA GTAAGGCTTA CGAGATTCTT TCAAATGAAG AAAAGAGATC

M00048714 ATTCAGATCA GCAAGGCATA CGAGATCCTG TCCAATGAGG AAAAGAGGAC

R00017247 ATTCAGATCA GCAAGGCTTA TGAGATTCTG TCCAATGAGG AGAAGAGGAC

AAATTATGAT CAATATGGAG ACGCTGGAGA GAACCAGGGC TACCAGAAGC

AAACTACGAC CACTATGGTG ATGCCGGTGA GAACCAAGGC TACCAGAAGC

AAACTATGAC CACTACGGTG ATGCGGGTGA GAACCAAGGC TACCAG---C

GAGAGTATCG CTTCCGCCAT TTCCATGAAA ATTTTTATTT TGATGAATCC

GCGAACACCG CTTCCGCCAT TTCCACGAGA ACTTCTATTT TGATGAGTCC

GCGAGTACCG CTTCCGCCAT TTCCACGAGA ACTTCTATTT TGATGAATCC

TTTTTTCACT TCCCTTTTAA TTCTGAACGG CGGGACTCAA TTGACGAA

TTTTTCCACT TCCCCTTCAA TGCGGAGCGG CGGGACTCGG GTGACGAG

TTTTTTCACT TCCCTTTCAA TTCCGAGCGG CGGGACTCAA TCGATGAG

>Ortholog Group 112, Repeat 1

3 132

H00349748 CCGCCGCCCG GCATGGGCCT CAATCAGAAT CGCGGCCCCA TGGGTCCTGG

R00018049 ---------- ---------- ---------- ---------- ----------

M00030623 CCGCCGCCGG GCATGGGCCT CAACCAGAAC CGCGGCCCCA TGGGCCCGGG

CCCGGGCCAG AGCGGCCCTA AGCCTCCGAT CCCGCCACCG CCTCCACACC

---------- ---------- ---------- ---------- ----------

CCCTGGC--- ---GGCCCGA AGCCGCCGCT CCCGCCTCCA CCTCCTCACC

CACCACCGCA GCAGCCACCG CCGCAGCAGC CG

---------- ---------- ---------- --

CGCCGCCGCA GCAGCCTCCG CCGCAGCAGC CG

>Ortholog Group 112, Repeat 2

3 135

H00349748 CCGCAT---C AGCCGCCGCC GCATCCACAG CCGCATCCGC CGCCACCGCC

R00018049 ---------- ---------- ---------- ------CCCC CGCCTCCGCC

M00030623 CCGCACCAGC AGCCGCCGCC GCACCAGCCG CCCCATCCCC CGCCTCCGCC

GCAGGACTCT TCCAAGCCCG TCGTTGCTCA GGGACCCGGC CCCGCTCCCG

GCAGGAA--- TCCAAGCCCG TCGTCCCGCA AGGCCCCGGC TCGGCGCCCG

GCAGGAA--- TCCAAGCCCG TCGTCCCCCA AGGCCCCGGC TCGGCGCCGG

GAGTAGGCAG CGCACCACCA GCCTCCAGCT CGGCC

GAGTGAGCAC TGCGCCGCCT CCGGGGGTCT CGGCC

GGGTGAGCAG TGCGCCTCCG CCGGCGGTCT CGGCT

>Ortholog Group 113, Repeat 1

3 198

M00041241 CCTCAACAGT CACAGCCACC TTCCCAGCCA CCCCTTACAT CTTTACCAGC

R00048900 CCTCAACAAT CACAGCCACC TTCCCAGCCA CCCCTTACAT CTTTACCAGC

H00425133 CCTCAACAAT CACAGCCACC TTCCCAGCAA CCCCTTACAT CTTTACCAGC

TCAGCCAACA GCACAGTCTA CAAGCCAATT GCAGGTTCAA GCTCTAGCTT

TCAGCCAACA GCACAGTCTA CAAGCCAGTT GCAGGTTCAA GCTCTAGCTT

TCAGCCAACA GCACAGTCTA CAAGCCAGCT GCAGGTTCAA GCTCTAACTT

CCCCTACAAA AGTCATACCA GCTTTGGGGA AAAGCCCGCC TCACCACTCT

CCCCTACAAA AGTCATACCA GCCTTGGGGA AAAGCCCGCC TCACCACTCT

CCCCTACAAA AGCTGTGCCG GCTTTGGGGA AAAGCCCGCC TCACCACTCT

GGATTCCAGC AGTATCAACA GGCAGATGCC TCCAAACAGC TGTGGAAT

GGATTCCAGC AGTATCAACA GGCAGATGCC TCCAAACAGC TGTGGAAT

GGATTCCAGC AGTATCAACA GGCAGATGCC TCCAAACAGC TGTGGAAT

>Ortholog Group 114, Repeat 1

3 207

H00375928 CCTCGGCCGG CTGTATTATC TGGCTATTTC AAACAGTTTC AGAAGTCTTT

R00039138 ---------- ---------- ---------- ---------- ----------

M00028016 CCTCGACCT- ---------- -GGCTATTTC AAACAGTTTC AGAAATCATT

ACCTCCACGA TTCCAGCGGC AGCAGGAACA GATGAAACAG CAGCAGTGG-

---------- ---------- ---------- ---------- ----------

GCCACCACGA TTTCAGCGTC AGCAGGAGCA GATGAAACAG CAACAGTGGC

--------GG TGTACTTCCA CAGACTGTTC CTTCACAACC GTCCAGTAGT

---------- ---------- ---------- ---------- ----------

AGCAGCAGGG TGTGCTTCCA CAGACTGTGC CCTCACAGCC GTCTAATGGT

ACTGTCCCTC CTCCACCACA CAGACCTCTT TATCAGCCTA TGCAGCCTCA

---------- ---------- ---------- ---------- ----------

TCAGTCCCTC CTCCACCACA CAGACCCCTT TACCAGCCAA TGCAACCCCA

TCCTCAG

-------

CCCTCAA

>Ortholog Group 114, Repeat 2

3 198

H00375928 CCAAATATTT GTAAAGTGAA ACCTCAGCAG TTACAGACAA GCAGCCTGCC

R00039138 ---------- ---------- ---------- ---------- ----------

M00028016 CCGAATATTT GTAAAGTAAA ACCCCAACAA TTACAGACCA GCAGCCTACC

TTCTGCAAGT CATTTTTCAC AGTTAAGCTG TATGCCTTCC CTTATTGCCA

---------- ---------- ---------- ---------- ----------

TTCTGCAAGT CACTTTTCAC AGTTGAGCTG TATGCCTTCC CTCATTGCAA

ATCCGCAAGT TTATGTGTCT CAGTCTGCAG CAGCTCAAAT CCCAGCCTTC

---------- ---------- ---------- ---------- ----------

GCCCGCAGGT TTATGTGTCT CAGTCTGCAG CAGCTCAAAT CCCAGCCTTC

TATATGGACA CAAGTCATTT ATTCAATACC CAACATGCAC GATTGGCT

---------- ---------- ---------- ---------- --------

TATATGGATA CAAGTCATTT ATTCAATACG CAGCATGCGC GATTGGCT

>Ortholog Group 115, Repeat 1

3 198

M00010319 GTGGCATTAA GGGGCAATGA AAACGAAAGA GAGATGGCCC CGCAGTCTGT

R00004905 GTGGCATTAA GGGGCAATGA AAACGAAAGA GAGATGGCCC CGCAGTCTGT

H00400694 GTGGCATTAA GGGGCAATGA AAATGAAAGA GAGATGGCCC CGCAGTCTGT

GAGTCCCCGA GAAAGTTACA GAGAAAACAA ACGCAAGCAG AAGCTGCCCA

GAGTCCCCGA GAAAGTTACA GAGAAAACAA ACGCAAGCAG AAGCTGCCCA

GAGTCCCCGA GAAAGTTACA GAGAAAACAA ACGCAAGCAA AAGCTTCCCA

GTTTCCAGCA GCTGGTTTCA GCCCGAAAAG AACAGAAGCG AGAGGAGCGC

GTTTCCAGCA GCTGGTTTCA GCCCGAAAAG AACAGAAGCG AGAGGAGCGC

GTTTCCAGCA GCTGGTTTCA GCCCGAAAAG AACAGAAGCG AGAGGAGCGC

CGACAGCTGA AACAGCAGCT GGAAGACATG CAGAAGCAGC TGCGCCAG

CGACAGCTGA AACAGCAGCT GGAAGACATG CAGAAGCAGC TGCGCCAG

CGACAGCTGA AACAGCAGCT GGAGGACATG CAGAAACAGC TGCGCCAG

>Ortholog Group 116, Repeat 1

3 102

R00029706 AGGGTACAAA ACACCATTCC CCAC------ ---------- ----------

M00044653 ---------- ---------- ---------- ---------- ----------

H00394875 CGGATGCCAG AGACAGTGCC CCAAGAGGAG ATGCCAGGGC CGCCACTGAA

---------- ---------- ---------- ---------- ----------

---------- ---------- ---------- ---------- ----------

TTCAGAGTCT GGGGAGGAGG CTCCCACAGG CCGGGACAAG AAGCGGAAGC

--

--

CT

>Ortholog Group 117, Repeat 1

3 198

R00030918 TGGCAGCTGC CCCCTCCTCC TCCCCAGAAT GGCTTCATGA ATGGCACCAT

H00353734 TGGCAGCTGC CCCCTCCTGC TACCCAAAAT ACCTTTGGGA ATAGCACTCT

M00112847 TGGCAGCTCC CCCCTCCTAC TCCCCAGAAT GGCTTTGTGA ATAGCACCAT

CCCTGTGGGG GCCGGGGAGC CGCTGCCCCA TAGGATAACT TGCCTGGCAG

TGCCCTGGGG CCTGGGGAAT CTTTGCCCCA CAGGTTAAGC TGTCTGGGGG

CCCTGTGGGG CCTGGGGAGC CACTGCCCCA TAGGATAACC TGTCTGGCGG

AAGCCCAAAT AGCAATGAGC GCTGTGAATA TGGGCCAGCC CCCGCTACCC

AACCCCCACT TGCCATGGGC ACTGTGAGCC TGGGCCAGCT CCCCCTGCCC

AAGCCCAAAT AGCAGTGAGT GCTGTGAATC TGGGCCAGCC CCCCCTACCC

CCGACCCCTC ATGTTTTCAC AGCTGGCACC AACACCGCTA TCCTGCCC

CCCATCCCTC ATGTGTTCTC AGCTGGCACT GGCTCTGCCA TCCTGCCT

CCAACTCCCC ATATTTTCAC AGCTGGCTCC AACACTGCTA TCCTGCCC

>Ortholog Group 118, Repeat 1

3 198

R00006691 CAGCTACAGC AGATCCTACA GCAGTACCAG CAAGTCATCC AGCACTCGCC

M00021670 CAGTTACAGC AGATCCTGCA GCAGTACCAG CAAGTTATCC AGCACTCACC

H00324463 CAGTTACAGC AGATTCTACA ACAGTATCAG CAGATTATAC AGCCCCCACC

ACATATACAG ACCATGTCTC TCGATGTGCA GCTGCGACAC TATGAGATGT

ACACATACAG GTA------- ---------- ---------- ----------

ACATATACAG ACCATGTCTG TAGATATGCA GCTGCGGCAT TATGAGATGT

TTCAACGTCT CTATCAGGAG TGGGAGCGAG AGTTCCAGCT GTGGGAGGAG

---------- ---------- ---------- ---------- ----------

TTCAACATCT TTACCAAGAA TGGGAGCGAG AGTTTCAGCT ATGGGAGGAA

CAGCTCCATT CCTACCCCCA TAAAGATCAG CTTGAGGAGT ATGAGAAG

---------- ---------- ---------- ---------- --------

CAACTCCATT CCTATCCTCA TAAAGATCAG CTTCAGGAGT ATGAGAAG

>Ortholog Group 119, Repeat 1

3 201

R00019968 AGTAGCAGTG GCTCGACACA CTGTGCATTT ACAGCCGAGC AGTACCAGCA

M00028100 AGTAGCAGTG GCTCAGCACA CTGTGCATTC ACAGCCGAAC AGTACCAGCA

H00263062 AGTAGCAGTG GTTCAGCACA CTTTGCATTT ACAGCCGAAC AATACCAGCA

GCACCAGCAG CAACTGGCAC TCATGCAGCA GCAGCAGCTT GCACAAACT-

GCACCAGCAG CAGCTGGCAC TCATGCAGCA GCAGCAGCTT GCGCAGACTC

ACATCAACAG CAACTGGCAC TCATGCAGAA ACAGCAGCTT GCACAAATT-

--GCAAATAG CAGTTCCTCC ACCGCCGCTC CA-------- ----------

AGGCAAATAG TAGTTCCTCT GCCGCCGCGC AA-------- ----------

--GCAAATAG TAATTCCTCC ACCAACACAT CACAGAACCT TGCATCTAAC

---------- ---------- ---------- ---------- ----------

---------- ---------- ---------- ---------- ----------

CAGCAGAAAA GTGGCTTTCG CCTGAATATA CAGGGTTTAG AAAGAACACT

-

-

A

>Ortholog Group 11, Repeat 1

3 201

R00037001 CCCGAGGGCA CTTTCTGGGC CAACAACCAT GGCCGCAACT ACACTGTCCT

H00055335 CCTGAGGGCA CTTTCTGGGC CAACAACCAC GGCCGCAACT ACACAGTCCT

M00122903 CCCGAGGGCA CTTTCTGGGC CAACAACCAT GGCCGCAACT ACACTGTCCT

GCTCCGGATC GCACCCGCTC CCACACCCAC TGATGCTGAA GGGCTGCCCC

GCTCCGGATC GCACCCGCTC CCACACCCAC TGATGCCGAA GGGCTGCCCC

GCTCCGGATC GCACCCGCTC CCACACCCAC TGATGCCGAA GGGCTGCCCC

TGCAGCAGCT GGAGCCACAG CCTGAGTGCC AGGGTCCTGT GGAGGCTGAG

TGCCGCAGCT GGAGCCACAG CCCGAGTGCC AGGGTCCCGT GGAGGCTGAG

TGCAGCAGCT GGAGCCACAG CCTGAGTGCC AGGGTCCTGT GGAGGCTGAG

GCCAGGCAGC TGAAGAGCTG CATGAAGCCG GTGAGGCGCA GGACAAGCCT

GCCAGGCAGC TGAAGAGCTG CATGAAGCCG GTGAGGCGCA GG---CCTGC

GCCAGGCAGC TGAAGAGCTG CATGAAGCCG GTGAGGCGCA GG---CCTTT

C

C

T

>Ortholog Group 120, Repeat 1

3 198

M00036507 AAATCTGAAG AGGTATATTG TTTGCAGAAA GAGCTAAAGA TAAAGACTCA

H00359299 AAATCTGAAG AGGTATATTG TTTACAGAAA GAGCTAAAGA TAAAAAATCA

R00035046 AAATCTGAAG AGGTATATTG TTTGCAGAAA GAGCTAAAGA TAAAGACTCA

TAATCTTGAA GAGACGAGTG AACAAAATGC CATCCTACAG CACACTCTTA

CAGTCTTCAA GAGACTTCTG AGCAAAACGT TATTCTACAG CATACTCTTA

TAACCTTGAA GAGACGAGTG AACAAAATGC TATTCTGCAG CATACGCTTA

TGTTACAGCA AGAGACCATG AGAAATGGAG AGCTAGAAGA TACTCAGAGT

TGTTACAACA AGAGACAATT AGAAATGGAG AGCTAGAAGA TACTCAAACT

TGTTACAGCA GGAGACCCTC AGAAGTGGAG AGCTAGAAGA TATCCAGAGT

AAACTTGAAA AACAGGTATC AAAGCAAGAA CAAGAGCTTC AGAAACAA

AAACTTGAAA AACAGGTGTC AAAACTGGAA CAAGAACTTC AAAAACAA

AAACTGGAAA AACAGGTATC AAAGCAAGAA CAGGAGCTGC AGAAACAA

>Ortholog Group 121, Repeat 1

3 198

R00017848 GTTGCCATCT TCACATTGAT GGTGGAGATA AACAAGAAAG GGAAAGCCCT

H00340507 GTTGCTATAT TTACACTGAT GGTAGAAATA AATAAAAAAG GAAAAGCTCT

M00031859 GTTGCCATCT TCACATTGAT GGTGGAGATA AACAAAAAAG GGAAAGCTCT

GCTGCACCAG CTTGAGAGTC TTGCAAAAGA CCATCGAATG AAACTTATGG

ACTGCATCAG TTAGAGAGCC TTGCAAAGGA CCATCGCATG AAACTTATGG

GCTGCACCAG CTTGAGAGTC TTGCAAAGGA CCATCGAATG AAACTCATGG

AAGTGGCTGG GCTTTCTAAG CAGTTAGAGC ATGTCATGCA TTTTTCTAAA

AAGTGGCTGG ACTCTCTAAA CAATTGGAGC ATGTCATGCA TTTTTCTAAA

AAGTGGCTGG GCTTTCTAAG CAGTTAGAGC ACGTCATGCA TTTTTCTAAA

TGGGCTGTTT CCAGTGGCAG CAGCACAGCC TTGCTGTACA GCAAGCGG

TGGGCAGTTT CCAGTGGCAG CAGTACAGCA TTACTTTATA GCAAACGA

TGGGCTGTTT CCAGTGGCAG CAGCACAGCC TTGCTGTACA GCAAGCGG

>Ortholog Group 122, Repeat 1

3 204

M00021606 TGTGAAAATA GTCCACAGAC ATCAAGTCCA GATCTCTCTT CAGAAGAGCT

H00369996 TCGCAAGATC TTCCAAAGAC ATCATGTGTA ACTCCTGCTT CAGAACAGCC

R00007505 TGTGAAGATA GTCCGCAGAC ATCAAGCACA GATCTTTCTT CAGAAGAGCT

GCGGAGGAGA CGAGAAGCCT ACTTTGAAAA G--------- ---CAACAGG

GAAGAAAATA AAAGAAGACT ATTTTGAAAA GCATCAGCAG GAACAGAAGT

GCGGAAGAGG AGAGAAGCCT ACTTTGAAAA G--------- ---CAACAGG

TAGACCGACC TGGACCCCTT TCATATCCAC GTGAAAGACC GACCACAAGT

CAGATCTGCC GGGCCACAGT TCATACCTAC ACGAAAGGCC AACAACAAGT

CAGACCGACC TGGATACCTT TCATACCCAT GTGAAAGACC CACCACAAGT

TCAGGAGGAC GTAGGAGCGA CCAAGGAGGC GACGCTGTGA GTGAAGAGGA

TCGAGAGCAA TTGAGAGTGA TCTCAGTGAT GACATC---- --AGTGAAGG

TCAGGAGGAC TCCGGAGCAA CCAAGCAGGC AATGCTATGA GTGAAGAGGA

CATG

CACA

CGTG

>Ortholog Group 123, Repeat 1

3 267

H00361066 GGAGATTATA ATACTTCTTT ACCCAGACCT GCACTGGGTG GCTCTATTCC

R00007768 GGAGACTACA GCGCAGCTTT ACCAAGACCC GCCTTGGGGA GCTCCGGGCC

M00085416 GCCGATTACA GTGCCACTTT ACCCAGACCT GCCATGGGGG GCTCTGTGCC

CACATTGCCT CTTCGGTCTA ATAGCATACC AGGTGCGAGA CCAGTATTG-

TACCTTGCCA CTTCGTTCTA ATAGACTGCC AGGCGCAAGA CCAACGTTGC

TACCTTGCCA CTTCGTTCTA ATCGACTGCC AGGTGCAAGA CCATCGTTGC

---------- ---------- ---------- ---------- -------ATG

AGCCTCAGCC GCAGCCGCAG CAGCAACAGC AGCAGCAACA GCAGCAAATG

AGCAACAGCA GCAGCAACAG CAGCAACAGC AACAACAACA GCAGCAACAG

---------- ---------- -CTTCAAATG AGGCCTGGTG AAATCCCCAT

CTTCAAATGA TCTTGATTGA CCTGGAGCTC AGAGCTGGTG AGGTTCCCAT

CAGCAGCAAC AGCAGCAGAT GCTTCAAATG AGAACTGGTG AGATTCCCAT

GGGAATGGGG GCTAATCCCT ATGGCCAAGC AGCAGCATCT AACCAACTGG

GGGAATGGGC GTCAGTCCCT ATAGCCCAGC AGTGCCATCT AACCAACCCG

GGGAATGGGA GTCAATCCCT ATAGCCCAGC AGTGCCGTCT AACCAACCAG

GTTCCTGGCC CGATGGC

GATCGTGGCC AGAGGGC

GTTCCTGGCC AGAGGGC

>Ortholog Group 123, Repeat 2

3 198

H00361066 AGCTCCCAGC AGGGTTTTCT TAATGCTCAA ATGGTCGCCC AACGCAGCAG

R00007768 ------TCCC AGGCTTTCTT TAATGCCCAA ATGGCAGCCC AGCAGAAACG

M00085416 ------CAGC AGGCTTTCTT TAATGCCCAA ATGGCTGCCC AGCAGAAACG

AGAGCTGCTA AGTCATCACT TCCGACAACA GAGGGTGGCT ATGATGATGA

AGAGCTGATG AACCATCACC TGCAGCAGCA GAGGATGGCG ATGATGATGC

AGAGCTGATG AGCCATCACC TGCAGCAGCA GAGGATGGCG ATGATGATGC

CCCAGGCCTT CAGCCCACCT CCTAATGTGA CTGCTTCCCC CAGCATGGAT

CTCAGGCCTT CAGCCCACCT CCCAACGTCA CCGCTTCCCC CAGCATGGAC

CTCAGGCCTT CAGCCCACCT CCCAACGTCA CCGCCTCCCC CAGCATGGAC

GGGCTTTTGG CAGGACCCAC AATGCCACAA GCTCCTCCGC AACAGTTT

GGGGTCTTGG CAGGCTCAGC AATGCCACAA GCCCCTCCAC AACAGTTT

GGGGTTTTGG CAGGTTCAGC AATGCCGCAA GCCCCTCCAC AACAGTTT

>Ortholog Group 124, Repeat 1

3 198

H00362238 GAAAATGACA AGATGAGACT TGAGAAAGAT TTATCATTCA AAGACACTCA

R00006415 GAAAATGACA AGATAAGACT TGAAAAGGAC TTGGCATTCA AGGAAAATCA

M00047655 GAAAATGACA AGATAAGACT TGAAAAGGAC TTGGCATTCA AAGAAAATCA

ATTAAAAGAG TACGAAGAAC TCTTGGCATC AGTGAGAGCA AATAATCACG

AATAAAAGAG TATGAAGAAC TCTTGGCATC AGTGAGAGCA AATAATCGCG

AATGAAAGAG TATGAAGAAC TCTTGGCATC AGTGAGAGCA AATAATCGCG

GACTTCAAGA CTCAAGTTCA AAATGCCAGG CATTGGAAGA AAACAATCTC

GACTTCAAGA CTCAAGCTCA AAATGTCAGA CATTGGAAGA AAATAACCTT

GACTTCAAGA CTCAAGCGCA AAATGTCAGT CATTGGAAGA AAATAACCTT

TCTCTTCGAC ATACACTATC AGACATGGAA TACAGACTAA AAGAACTG

TCTCTTCGAC ATACACTGTC GGACATGGAA TACAGACTAA AAGAACTT

TCTCTTCGAC ATACACTGTC AGACTTGGAA TACAGACTGA AAGAACTT

>Ortholog Group 125, Repeat 1

3 150

H00362013 CTGGCTCAGG TGCCAGTGCA GGGAGGACAG CCACTGCAGG CCCCACAGAT

M00077103 CTGGCCCAGA TGTCCATGCA AGGAGGACAG CCACTGCAAG CCCCTCAGGT

R00061868 CTGGCCCAGA TGTCCGTGCA AGGCGGACAG CCACTGCAAG CCCCTCAGGT

GCTGTCACAG CACATGCAAC AGATGCAGCA GCACCAGTAT TAC------C

GCTGTCCGGC CATATGCAAC AATTGCAGCA GCACCAGTAT TACCCACAGC

GCTGTCCAGC CATATGCAGC CATTGCAGCA GCACCAGTAT TACCCACAGC

CACCGGCCGG GCAACAGCGT ATCTCCATGC AAGAAATA-- ----CAGACG

CGCCGGCCGG ACTGCAGCGG ATCTCTGTGC AGGAGATGCA GCAGCAGCAG

CGCCGGCCGG ACTGCAGCGG CTCTCCGTGC AGGAGATGCA GCAGCAGCAG

>Ortholog Group 125, Repeat 2

3 84

H00362013 CCGCAACAAA TTCGCCCATC ACAGCCACAG CCGCCGCCAC CGCAGCAGCT

M00077103 CCGCAGCAAA TTCGCCCCTC A--------- ---CCACCT- --CAGCAGCT

R00061868 ---CAGCAGA TTCGCCCCTC A--------- ---CCGCCT- --CAGCAGCT

ACAGCTGCAG CAGCGGCAGG GTTCAATGCA GATA

CCAGCTGCAG CAGCGGCAGA GTTCACTGCA GATA

CCAACTGCAG CAACGGCAGA GTTCACTGCA GATA

>Ortholog Group 125, Repeat 3

3 144

H00362013 CCTCAGTATT ATCAGCCCCA ACCCATGATG CAGCACTTGC AAGAGATGCA

M00077103 CCTCAGTATT ATCAGCCCCA ACCCATGATG CAACACTTGC AAGAGATGCA

R00061868 CCTCAGTATT ATCAGCCCCA ACCCATGATG CAACACTTGC AAGAGATGCA

CCTGCAGCCT CCTTCTTATC ACAGGGACCC TCACCAGTAT ACCCCAGAGC

CCTGCAGCCA CCCTCATACC ACAGGGACCC TCATCAGTAT ACCCCGGAGC

CCTGCAGCCC CCTTCGTACC ACAGGGACCC TCACCAGTAT ACCCCGGAGC

AGGCACACAC TGTCCAGCTG ATTCCCCTGG GCTCCATGTC CCAG

AGGCACACGC TGTCCAGCTG ATCCAGCTGG GCTCTATGCC CCAG

AGGCACATGC GGTCCAGCTG ATCCAGCTGG GCTCCATGCC CCAG

>Ortholog Group 126, Repeat 1

3 102

H00244769 GGGGCCACCA CTCCATCCCA GCGCTCCCAG CTGGAGGCCT ATTCCACTCT

R00023140 GGGGCCACCA CTCCATCACA GCGCTCCCAG CTGGAGGCAT ATTCCACCCT

M00089217 GGGGCCACCA CTCCATCACA GCGCTCCCAG CTGGAGGCTT ATTCCACCCT

GCTGGCCAAC ATGGGCAGTC TGAGCCAGAC GCCGGGACAC AAGGCTGAGC

GCTGGCCAAC ATGGGCAGTC TGAGCCAGGC ACCAGGACAC AAGGTTGAG-

GCTGGCCAAC ATGGGCAGTC TGAGCCAGGC ACCAGGACAT AAGGTTGAG-

AT

--

--

>Ortholog Group 126, Repeat 2

3 105

H00244769 CATCACCTCA GCAGGGCTCC GGGGCTCATC ACCCCGGGGT CC---CCCCC

R00023140 ---CACCTCG GCAGGGCTGC GGGATTAGTC AACCCGGGGT CC---CCTCC

M00089217 ---CACCTCA GCAGGGCTGC AGGATTAGTC AACCCGGGGT CCCCTCCTCC

ACCAGCCCAG CAGAACCAGT ACGTCCACAT TTCCAGTTCT CCGCAGAACA

ACCTACCCAG CAGAACCAGT ACATTCACAT TTCCAGCTCT CCACAGAGCT

ACCCACCCAG CAGAACCAGT ACATCCATAT TTCCAGCTCT CCACAGAGCT

CCGGC

CCGGG

CCGGG

>Ortholog Group 127, Repeat 1

3 222

H00352514 AACTTCTTTT GGGATCCGAG CACCAGCCGG CGCTTCAGCC CCCCCTCCAG

R00057733 ---------- --GATCCGAG CACCAGCCGG CGCTTCAGCC CCCCCTCCAG

M00123743 AGCTTCTTTT GGGATCCGAG CACCAGCCGG CGCTTCAGCC CCCCCTCCAG

CAGCCTGCAG CCCGGCAAAA TGAGCGACGT GAGCCCGGTG GTGGCTGCG-

CAGCCTGCAG CCCGGCAAGA TGAGCGACGT GAGCCCGGTA GTGGCTGCTC

CAGCCTGCAG CCCGGCAAGA TGAGCGACGT GAGCCCGGTG GTGGCTGCG-

--GAG----- ---------- ------GCGG CGGCGGCGGC TGCGGCGGCG

AGCAGCAGCA GCAGCAGCAG CAGGAGGCGG CCGCAGCAGC AGCAGCGGCA

--CAGCAGCA GCAGCAGCAG GAGGCGGCCG CAGCAGCAGC GGCGGCAGCG

GCGGCGGCTG CGGCGGCGGC AGCTGCAGTG CCCCGGTTGC GGCCGCCCCA

GCGGCGGCGG CAGCAGCGGC GGCAGCCGTG CCCCGGTTGA GGCCGCCGCA

GCGGCGGCAG CAGCGGCGGC GGCCGCAGTG CCCCGATTGA GGCCGCCGCA

CGACAACCGC ACCATGGTGG AG

CGACAACCGC ACCATGGTGG AG

CGACAACCGC ACCATGGTGG AG

>Ortholog Group 128, Repeat 1

3 198

H00313500 TGGATTGGCA ATCAGAGAAG AGTGACAGTG ACAGAAGTGC TCAGAGAGAC

M00097961 AGCTTGAGTA GTCAGCAAAG AATGACGGGA GCAAAGGTGT TCCAAGAGTC

R00028383 AGCTCAGGCA GTCAGCAAAG AATGACAGGA ACAAAAGTGT CCCAGGAGTC

AGCAAGACCT CAGTCCTCAG CCTTACACCC CCTACTCACC TTTGAGAGCG

GTCAGGCCCC CAGCCCTCAG CCGCACACTC TGGATATATT TTTGAGAGCA

ATCAGGACGT CAGTCCTCGG CCGCACACTC TGGATTTATC TTTGAGAGCA

TTGGTGGCTT TGAGGGGCCT GAACAAGACG AATTTGATAA AGTCCTGGCA

TTGGTGACTT TGAAGCACCT GATCAAGATG ACCTTGACAA GGCCCTGGCA

TTGGTGACTT CGAAACACTT GATCAAGATG AGTTTGACAA GGCCCTGGCG

AGCATGGAGT TGGAGGAGCC TGGCATGGAG CTGGAATGTG GAGTCAGC

AGCATGGAGT TCGAGGGAGC TGGCTTGGAG CTAGAA---- --GCCGAC

AGCATGGAGT TCGAGGGAGC TGGCTTGGAG CCAGAA---- --GTTAAC

>Ortholog Group 129, Repeat 1

3 198

H00245564 GACAGAGCAT GCCACACAAG CCAGCTCACC CCAGGGACAC CTCCACCCTC

R00027579 GACAGAGCTA GCCACACCAG TAAGCTCAAC CCAGGGACAC CTTTGCCCTC

M00103118 GACAGAGCTA GCCACACCAG TAAGCTCAAC CCAGGGACAC CTCTGCCCTC

TGCCCTTCAT GCATGTACCA CTGGGGAAGA AATCTTGGCT CAGTATTTAC

CACTCTCCAT GCGTGTGCCT CTGGAGAAGA AGTCTTGGCC CAGTACTTAC

TGCTCTCCAC GCATGCGCCT CTGGAGAAGA AGTCTTGGCC CAGTACTTAC

CTGGAGTCAT GAGTTCTTCC CATCTGCTGC TGACTCCCTG CAGGGTGGCT

CTAGGGTCTT GAGTTCGTCA TATCTGCTGC TGACACCCTG CAAAATGGCT

CTAGAGTCTT GAGTTCGTCA CATCTGCTGC TGACTCCCTG CAAAGTGGCT

CCTCCTTACC CCCACCTCTT CTCAAGCTGC AGTCCACCGG GTATGGTT

CCTCCGTACC CACACTTCTT CTCAAGCTTC AGTCAGAAAG GGTTGGCC

CCTCCATACC CACACTTCTT CTCAAGCTTC AGTCAGAAAG GGCTGGCC

>Ortholog Group 12, Repeat 1

3 198

M00047962 GCACCGCTGG AGAGCAGCTC CGGGGCGCGG GGATCCTTTA ACAAGTTCCA

R00000009 GCACCGCTGG AAAGCAACGC TGGGGCGCGG GGATCCTTTA ACAAGTTCCA

H00332744 ACACCCCTGG AGAGTGGAGC TGGGGCGCGG GGCTCCTTTA ACAAGTTCCA

GCCGCAGCCG CCCACCCCAC AGCCGCCGCC CGCTCCA--- ----------

GCCGCAGCCG CCCACCCCGC AGCCGCCGCC CGCTCCG--- ----------

GCCCCAGCCG TCGACCCCGC AGCCCCAGCC GCCGCCGCAG CCGCAGCCGC

-----CCCGC GCCGCCTGCG ------CATC TCTACTTGCA GAGGGGCGCC

-----CCCGC GCCGCCTGCG ------CATC TCTACTTGCA GAGGGGCGCC

CGCAGCCCCA GCCGCCCGCG CAACCGCATC TTTACTTGCA GCGAGGCGCC

TGCAAAACGC CCCCGGACGG TAGCCTCAAG CTCCAGGAGG GCAGCGGC

TGCAAAACGC CCCCGGACGG CAGCCTCAAG CTCCAGGAGG GCAGCGGC

TGCAAGACGC CCCCGGACGG CAGCCTCAAA CTCCAGGAAG GCAGCAGC

>Ortholog Group 130, Repeat 1

3 198

M00045918 GCTTCTCCGT GTCAGGAGCA CAAGCGAGGT GGCCTAAGCG AGCTAAAGGG

R00017742 GCTTCTCCGT GTCAGGAGCA CAAGCGAGGT GGCCTGAGCG AGCTGAAGGG

H00315955 GCCTCCCCGT GCCAGGAGCA CAAGCGAGGG GGCCTGGGAG AGCTGAAGGG

AGCACCTGCC TCTGCGCTGA GTCCTCCCGA GCCGGCGCCC TCGCCTGGGG

AACACCTGCC TCTGCGCTGA GTCCTCCGGA GCCGGCGCCC TCGCCTGGGG

GACGCCGGCT GCGGCGCTGA GCCCCCCAGA GCCGGCGCCC TCTCCCGGGG

CTGCAGCCCA CCTGCTGGGC CCACCTCACC ACCCAGGCCT GCCACCAGAG

CTGCAGCCCA CCTGCTGGTC CCACCTCACC ATCCTGGCCT GCCACCAGAG

CCGCGGCCCA CCTGCTGGGC CCGCCCCACC ACCCGGGCCT GCCGCCTGAG

GCCCACCTGA AGCCCGAGCA CCATTACGCC TTCAACCACC CCTTCTCT

GCCCACCTGA AGCCCGAGCA CCATTACGCC TTCAACCACC CCTTCTCT

GCCCACCTGA AGCCGGAACA CCACTACGCC TTCAACCACC CGTTCTCC

>Ortholog Group 131, Repeat 1

3 198

R00009140 GAGGCCTACC AGATCCCTGC CTCGTACCAG CCTGATGAGG AGGAGCGAGC

H00348632 GAGGCCTACC AGGTCCCCGC CTCATACCAG CCCGACGAGG AGGAGCGAGC

M00105473 GAGACCTACC AGATACCTGC TTCATACCAG CCTGACGAGG AAGAGCGAGC

ACGCCTGGCT GGTGAGGAGG AGGCGCTGCG CCAGTATGAG CAGCGGAAAG

GCGCCTGGCG GGCGAGGAGG AGGCGCTGCG TCAGTACCAG CAGCGGAAGG

ACGCCTGGCC GGCGAGGAGG AGGCGCTGCG CCAGTACCAG CAGCGGAAAG

AGGGGAACTA CCTGCAGCAC GTGCAGCTGG AGCAGAGGAG CCTGGTGCTG

AGGGGAACTA CCTGCAGCAC GTCCAGCTGG ACCAGAGGAG CCTGGTGCTG

AGGGGAACTA CCTGCAGCAC GTGCAGCTGG AGCAGAGGAG CCTGGTGCTG

AACACCGAAC CTGCTGAGTG CCCTGTGTGC TACTCAGTGC TGGCACCC

AACACGGAGC CCGCCGAGTG CCCCGTGTGC TACTCGGTGC TGGCGCCC

AACACCGAAC CTACTGAGTG CCCTGTGTGC TACTCAGTGC TGGCACCC

>Ortholog Group 132, Repeat 1

3 198

R00007888 GAGGCTCTGC AGAAGCGCCT GGAGGAGGTC ACCCGGGAGC TATGCCGGGC

H00246043 GAGGCCCTGC AGAAGCGCCT GGACGAGGTC AGCCGGGAGC TGTGCCACAC

M00016072 GAGGCTCTGC AGAAGCGCTT GGAGGAGGTC ACCCGGGAAC TCTGCCGGGC

ACAGACGAGC CATGCCAACC TCCGAGCGGA TGCTGAGAAG GCTCAGGAAG

GCAGAGCAGC CACGCCAGCC TCCGGGCGGA TGCCGAGAAG GCCCAGGAGA

ACAAACGAGC CATGCCAACC TCCGAGCGGA TGCTGAAAAG GCTCAGGAAG

TGGCAGAGTT GCACAGCAAA TTACAGTCCT CGGAGGTGGA GGTAAAAAGC

TGGCCGAGCT GCACAGCAAG TTACAGTCCT CCGAGGCGGA GGTGCGCAGC

TGGCAGAGTT GCACAGCAAA TTACAGTCAT CTGAGGTGGA GGTAAAAAGC

AAGTGTGAGG AGCTGAGTGA TCTCCATGGG CAGCTCAAGG AGGCCAGG

AAATGCGAGG AGCTGAGTGG CCTCCACGGG CAGCTCCAGG AGGCCAGG

AAGTGTGAAG AGCTGAGTAG TCTCCATGGG CAGCTCAAGG AGGCCAGG

>Ortholog Group 133, Repeat 1

3 108

M00116189 GGTGACCACT CTGCTCACCT GTCAAGGCAT CAGCAGCATC TTCTTTCGAG

R00040549 GGAGACCACT CTGCTCACCT GCCGAGGCAT CAGCAGCATC TTCTTCCGAA

H00429013 GGAGACCATT CAGCTCACCT GCCTAGGCCG CAGCAGCATT TCCTTCCTAA

TCAGAGTCAT CAGGGGGACC ACTACCGTCA CGCCCAGGCC AGCCTGACT-

TCAGAGTCAT CAGGGGGACC ACTACCGGCA CCCCCAGCCC AGCCTGACT-

TCAGGCTCAC CAGGGGGATC ATTACCGTCT CTCCCAACCT GGCCTGAGTC

--------

--------

ACCATCAT

>Ortholog Group 133, Repeat 2

3 108

M00116189 ---------G CGCAGCAGCA GCCAGGAGAA GCCTACTCAG CTATGCCTCG

R00040549 ---------G CCCAGCAGCA ACCAGGAGAA GCCTACTCAG CTATGCCTCG

H00429013 CATCACCACC CACAGCAGCA GCCAGGAGAA GCCTATTCAG CTATGCCTCG

GGCTCAG--- CAGTCTGCTT CTTATCAGCC CATGCCAGCT GACCCTTTTG

GGCTCAG--- CAGGCAGCTG CTTATCAGCC CATGCCAGCT GACCCTTTTG

GGCTCAGCCA TCCTCTGCTT CTTATCAGCC AGTGCCAGCA GACCCTTTTG

CTATGGTC

CCATCGTC

CCATTGTT

>Ortholog Group 134, Repeat 1

3 219

H00380019 GCAGCAGCGG CAGCAGTG-- ---------- GCCCAGGCCC AGGCTCAAGC

R00052839 GCAGCGGCAG CGGCAGTG-- ---------- GCCCAGGCTC AGGCCCAGGC

M00108046 GCAGCAGCAG CAGCGGTGGC CCAGGCCCAG GCCCAGGCTC AGGCCCAAGC

TCAAGCCCAG GCTCAGGCTC AGGCTCAAGC CCAGGCCCAG GCCCAGGCCT

TCAGGCTCAG GCCCAGGCCC AGGCTCAGGC CCAGGCCCAG GCCCAGGCTC

TCAAGCCCAG GCCCAAGCTC AAGCCCAGGC CCAAGCTCAA GCCCAGGCCC

CCCAGGCATC ACCA------ ---CCACCAC ACTTCCAGTC TCCTGGGGCA

AGGCTCAGGC CCCACCTCCA CAACCACCAC ACTTCCAGTC TCCTGGGGCA

AGGCCCAGGC CCCACCGCCA CAACCACCAC ACTTCCAGTC CCCTGGGGCA

GCCCCCCAGG GTGGGGGTGG TGGGGACAGC AATCCCAACC CTCCACCCCA

GCTCCCCAGG GAGGGGGTGG TGGGGACAGC AACCAGAACC CTCCACCCCA

GCTCCCCAGG GAGGGGGTGG TGGGGACAGC AACCCGAACC CTCCACCCCA

GTGTTCCTTT GACCTGACC

GTGTTCCTTT GACCTGACC

GTGTTCCTTT GACTTGACC

>Ortholog Group 135, Repeat 1

3 198

M00111134 ATGAGCACTT TAAGCAGCCA ATTAGATGCT GGCAGCAGGG ATGGGAGATC

H00386200 ATGAGCACTC TAAGCAGCCA ATTAGATGCT GGCAGCAGAG ATGGAAGATC

R00007759 ---------- ---------- ---------- ---------- ----------

AAGTGGTGAC ACCAGCTCTG AAGTAAGCAC AGTGGAGCTG CTGCATCTGG

AAGTGGTGAC ACCAGCTCTG AAGTAAGCAC AGTAGAACTG CTGCATCTGG

---------- ---------- ---------- ---------- ----------

CTCTCCAGGC AGCAAGACAA CTTTTGTTAC AGCAGCAAAC CAGTGGATTG

CTCTCCAGGC AGCAAGACAA CTTCTTTTAC AGCAGCAAAC AAGTGGATTG

---------- ---------- ---------- ---------- ----------

AAATCTCCTA AGAGCAGTGA GAAGCAGAGG CCACTGCAGG AATTGCTC

AAATCTCCTA AGAGCAGTGA TAAACAGAGA CCACTGCAGG AATTGCTT

---------- ---------- ---------- CCATGCAAG- --------

>Ortholog Group 135, Repeat 2

3 105

M00111134 TCGGTGGCCA TGATGACTCC CCAAGTGATC ACCCCTCAGC AGATGCAGCA

H00386200 TCAGTGGCCA TGATGACTCC CCAGGTGATC ACCCCTCAGC AAATGCAGCA

R00007759 TCGGTGGCCA TGATGACTCC CCAAGTGATC ACCCCTCAGC AGATGCAGCA

GATTCTTCAG CAGCAGGTCC TGTCTCCTCA GCAGCTCCAG GCCCTCCTCG

GATCCTTCAG CAACAAGTCC TGTCTCCTCA GCAGCTACAA GCCCTTCTCG

GATCCTTCAG CAGCAGGTCC TGTCTCCTCA GCAGCTCCAG GCCCTCCTCG

CTGTG

CTGTC

CTGTG

>Ortholog Group 135, Repeat 3

3 30

M00111134 ATGTTGCTAC AAGAGTTTTA CAAGAAACAG

H00386200 ATGCTGCTAC AAGAGTTTTA CAAGAAACAG

R00007759 ATGCTGCTAC AAGAGTTTTA CAAGAAACAG

>Ortholog Group 135, Repeat 4

3 36

M00111134 GAACAGTTAC ATCTTCAGCT TTTGCATCCT GGAAAG

H00386200 GAGCAGTTAC ATCTTCAGCT TTTGCATCCT GGAAAG

R00007759 GAGCAGTTAC ATCTTCAGCT TTTGCATCCT GGAAAG

>Ortholog Group 135, Repeat 5

3 39

M00111134 CAAGCAAAAG AGTTGGCGGC GCAGCAGCTC GTCTTCCAG

H00386200 CAAGCGAAAG AGTTGGCAGC CCAGCAGCTT GTCTTCCAG

R00007759 CAAGCAAAAG AGTTGGCCGC TCAACAGCTT GTCTTCCAG

>Ortholog Group 135, Repeat 6

3 126

M00111134 CAGCAGCTTC TCCAAATGCA GCAGCTACAT CTGCTCAGCC TTCAGCGCCA

H00386200 CAGCAGCTTC TCCAGATGCA ACAACTCCAT CTGCTCAGCC TTCAGCGTCA

R00007759 CAACAGCTTC TCCAGATGCA GCAACTACAT CTGCTCAGCC TTCAGTGTCA

GGGCCTCATC TCCATCCCAC CCGGCCAAGC AGCCCTTCCT GTCCAGTCGC

GGGACTCATC TCCATTCCAC CTGGCCAGGC AGCACTTCCT GTCCAATCGC

GGGCCTCATC TCCATACCAC CCGGCCAAGC AGCACTTCCC GTCCAGTCGC

TGCCTCAAGC TGGCTTAAGT CCTGCT

TGCCTCAAGC TGGCTTAAGT CCTGCT

TGCCTCAAGC TGGCTTAAGT CCTGCC

>Ortholog Group 136, Repeat 1

3 198

H00249499 GCGACTGGGA CGGGCGGCTC GTCGGAGCCC TCAGCTTGCA GCGACCACCC

R00059218 AAACCACAGT GC------AG TTCTGAACCA CTAAATTGGG AGATCCACNC

M00058490 GGGCCAGGGG CGGTAGGCTC GTCCGAGCCC TCAGCTTGCA GCGATCACCC

GATCCCAGGC TGTTCGCTGA AGGAGGAGGA GAAGCAGCAT TCGCAGCCGC

GAGCCCGGGC TGCCCGCTGA AGGAGGAGGA GAAGCAGCCG CCGCAGCCGC

GAGCCCGGGC TGCTCGCTGA AGGAGGAGGA GAAGCAGCCG CCGCAGCCGC

TTGACCCAAA CAACCCCGCC GCGAACTGGA TCCACGCTCG CTCCACCCGG

TTGACCCAAA CAACCCTGCA GCGAACTGGA TCCACGCTCG CTCCACCCGG

TTGACCCAAA CAACCCTGCA GCGAACTGGA TCCACGCTCG CTCCACCCGG

AAAAAGCGCT GTCCCTACAC CAAATACCAG ACGCTTGAGC TGGAGAAA

AAAAAGCGCT GTCCCTACAC CAAATACCAG ACGCTAGAGC TGGAGAAG

AAAAAGCGCT GTCCCTACAC CAAATACCAG ACGCTAGAGC TGGAGAAG

>Ortholog Group 137, Repeat 1

3 276

H00349856 GTGGCACAGA GCAACAGCAG T--------- ---------- -----GAGGA

R00013663 GTGGCTCAGA GCAACAACAG CAGCAGCAGC AGCAGTAGCA GCATCGAGGA

M00064892 ---------- ---------- ---------- ---------- ----------

AGAGGCCAGA GAGGCTGGGT CCCCTGCACA GGAGTTC--- AAGTATCAGA

GGAAGTCCGG GAGGCTGGGT CCCCTGCACA GGAGTTCAGC AAGTACCAGA

---------- ---------- ---------- ---------- ----------

AGTCCCTTCC TCCCCGATTC CAGCGC---- ---------- ----------

AGTCCCTTCC TCCCCGTTTC CAGCGCCAGC AACAGCAGCA GCAGCAGCAG

---------- ---------- ---------- ---------- ----------

---------- ---------- -------GAG CAGCTGTACA AGATGCAGCA

CAACAACAAC AGCAGCAGCA GCAGCAAGAG CAGCTGTACA AGATGCAACA

---------- ---------- ---------- ---------- --ATGCAGCA

CTGGCAGCCG GTGTACCCCC CGCCGTCCCA CCCCCAGCGC ACCTTTTACC

CTGGCAGCCG GTGTACCCCC CACCTTCTCA CCCCCAGCGT ACCTTCTACC

CTGGCAGCCG GTCTACCCCC CACCTTCTCA CCCCCAGCGC ACCTTCTACC

CACACCACCC CCAGATGTTG GGCTTC

CACACCACCC CCAGATGCTG GGCTTT

CACACCACCC CCAGATGCTG GGCTTT

>Ortholog Group 138, Repeat 1

3 198

M00034700 TCCCGAGAGC ACGTGGCTGT GGTTGTGGGT TCAGGCATTG GCTATGGAGC

H00377144 CCACAAGGGC ACGTGGCTGT GGCCGTGGGC TCAGGTGGCA GCTATGGAGC

R00012138 TCACAAGAGC ATGTGGCTGT GGTTGTGGGT TCAGGCATTG GCTATGGAGC

TGAAGTAGGG ---GAGGAAG AGGATGACAA GACATCACTT CTACAGGAAC

CGAGGATGAG GTGGAGGAGG AGAGTGACAA GGCCGCGCTC CTGCAGGAGC

CGAA---GGA ---GAGGAAG AGGATGACAA AACATCACTT CTACAGGATC

CCAGATTCTG GACATTTGAC TACTATCAGA GCTTTTTTGA TGTGGATACC

CGGGATTCTG GACCTTCAGC TACTATCAGA GCTTCTTTGA CGTGGACACC

CAAGATTCTG GACGTTTGAC TACTATCAGA GCTTTTTTGA TGTAGACACC

TCCCAGGTCT TGGACAGGAT CAAAGGCTCC CTGCTGCCCC ATCCTGGC

TCACAGGTCC TGGACCGGAT CAAAGGCTCA CTGCTGCCCC GGCCTGGC

TCCCAGGTCT TGGACAGGAT CAAAGGTTCC CTGCTGCCCC ATCCTGGC

>Ortholog Group 139, Repeat 1

3 198

M00028984 GAAGGACGGA TCCAGAGCAT GCGCCTTCTG GACCGTCTGA ACGTCTCAGG

R00018512 GAAGGACGAA TCCAGAGCAT GCGTCTTCTG GACCGTCTGA ACGTCTCAGG

H00364603 GAAAGCCGAA TTCAGAACAT CCACTTTGGG GACAGACTGA ATGCCTCAGC

GACAGTGGCT CCAGGGATGG TGGGCTGGCT GATTGGTGGC ATGAACTTCG

GACAGTGGCC CCAGGGATGG TGAGCTGGCT TATTAGTGGC ATGAACTTCG

ACAAGTGGCC CCAGGGCTGG TGGGCTGGCT AATCAGCGGC AGGAAACACG

AAATCAGCAT CAACATTACC AATGTGCAGC TGGACTGTGG TGGGATCCAG

AAATCAGTAT CAACATTACC AATGTGCAGC TGGACTGTGG CGGGATCCAG

AGAGCAGCAT CAACATCACC AACATTCAGC TGGACTGTGG TGGGATCCAG

ATGGCTTTCC CCAAAGAGTG GTTCTCTGCA AACATCACAC TTGAATTT

ATGGCTTTCC CCAAAGAGTG GTTCTCCGCA AACATCACAC TTGAATTT

ATATCATTCC ATAAGGAGTG GTTCTCGGCA AATATCTCAC TTGAATTT

>Ortholog Group 13, Repeat 1

3 198

R00055547 ---------- ---------- ---------- ---------- ----------

M00119120 GTGGAGCAGC TGGCTCACAG GAAGTGTTTC CAGCAAGCCA CAGAACACAG

H00350719 GTGGAACAGC AGATTCAAAA GAAGTATTCT CAGCAGGTAG TGGAATATGA

---------- ---------- ---------- ---------- ----------

CGAACTCACC TGCCTCCTGG AGAAGCTCCA GGACCTTAAG GTTTCTCTGC

TGAATTTACA ACCCTCATGA ATAAGGTACA GGACACTGAG ATTTCTCTGC

---------- ---------- ---------- ---------- ----------

GCCTAACGCT AAGTCTGAAC TCTCCAGGAC AACAGGCGGC GATCGTGGAC

ATCTACAGTT AAGGCTGAAG TCTCCAGAAG AACGGGCAGG GAACCAAAGC

---------- ---------- ---------- ---------- --------

ATGGTCACTC CAGCCGCTGA GCTCCAGGCA ATCAAGTGTG AGTTTTCT

ATGATTGCCT TGACCACTGA CCTCCAGGCT ACCAAGCATG GATTTTCT

>Ortholog Group 13, Repeat 2

3 198

R00055547 ---------- ---------- ---------- ---------- ----------

M00119120 TCAGAGCCC- -----CACGA CGTCGACCAG CCAGCTGGCC TTTCTGAGCT

H00350719 TCAGAGCCAG AGCATCAAGA AGCTCTCCAA CCAGTTAACC TTTCTGAATT

---------- ---------- ---------- ---------- ----------

TGACTCTGTC CTAACTGAAA GGCCACAGTT CAGCAGACAG AAGGATGCTA

GGAATCCATT GTAACTGAAA GGCCACAATT CAGCAGACAA AAAGATTTCG

---------- ---------- ---------- ---------- ----------

TTCTGGAGTT AAAACCATCA GAGCAGAAAG ATTTAATCAA GTTCACAGAA

TTCTGGAGTT AAAACCAATG GAACAGAAAG ATTTCATCAA ATTCATAGAA

---------- ---------- ---------- ---------- --------

CTGAATGCCA AGAAAACATG GCTGCAGGGT CACCAAGAGA ATGAAGAT

TTTAATGCTA AGAAAATGTG GCCCCAGTAT TGCCAACATG ATAACGAT

>Ortholog Group 140, Repeat 1

3 270

M00024717 GGCAGTATGG GTGGCCCTGT ACCCGGAAAC AACAGCAGCA GTTCCTTCTC

H00402935 GGCAGTGCAG GTGGCCCTGT ACCTGGAGGC AACAGCAGTA GCTCCTCCTC

R00014171 GGCAGTATGG GTGGCCCTGT GCCTGGGAGC AACAGCAGCA GCTCTTTCTC

TGCTGCCATC CCCACCAGGA CCTCCACAGA AGCCCCAAGG CATCATTTGC

TGTTGTAATT CCTACCAGGA CCTCAGCAGA GGCCCCAAGC CATCACTTG-

TGCTGCCGTC CCCTTCAGGA CCTCCGCAGA AGCCCCGAGG CATCATTCTC

TGCAGCAGCA ACAGCAGCAG CAGCACCAGC AGCAGCAGCA GCAGCAACCA

---------- ---------- ---------- ---------- ----------

AGCAGCAGCG GCAGCAGCAG CAGCAGCAGC AGCAGCAGCA GCAGCAG---

CAGCAGCAGC AGCAGCAACA CAGACTGATG AAATCTGAAA GCATGCCGGT

---------- ---------- -AGGCTGATG AAATCAGAAA GCATGCCTGT

---------- ---------- -CGGTTGATG AAGTCTGAAA GCATGCCGGT

ACAGTTGAAC AAAGGCGACG TAGTTACAGG AAGCGATGCT CAGGTTTCTG

GCAATTGAAC AAAGGCGATG TAGTTACAGG AAGCGATGCT CAGGTTTCTG

ACAGTTGAAC AAA------- ---------- ---------- ----------

TTCCTGTCCA GGCTCTAACT

TTCCTGTCCA GACTCTAACT

---------- ----------

>Ortholog Group 141, Repeat 1

3 204

M00103539 TGCGTCTGGT CGTTCCGCGG GCGCCAGGGG ACCGGCAAGC AGCAGCCTCA

R00027062 TGCGTCTGGT CGTTCAGCGG GCGCCAGGGG ACCCGCAAGC AGCATTCTCA

H00303282 TGCGTCTCGT CCTTCCGCGG GCGCCAGGGG GCCAGCAAGC AG------CA

GCCGGTGCCA ACGCCGCAGC CGCCTGAGTC CTCACCGCCG CCTCTGCCGC

GCCGGCGCCA ACGCCGCAGC CGCCTGAGTC CTCACCGCCG CCTCTGCTAC

GCCGGCGCCA CCGCCGCAGC CGCCCGAGTC CCCGCCGCCG CCACCGCTGC

CGCCGTGCTC TCAGCCCGGC ACTGCCGCCT CCCCGGCGGG TGCCCCGCTT

CGCCGTGCGC TCAGCCCGGC ACCGCCGCCT CCCCGGCGGG TGCCCCGCTT

CGCCGCCTGC GCAGCCCGGC CCCGCCGCGT CCCCGGCGGG CCCCCCGGCA

TCCTGCGGGC CTGGGGGCCG GCGTGCCGAG CCATGCCCCG GGCTGCCGGC

TCCTGTGGGC CTGGGGGCCG GCGCGCCGAG CCATGCTCCG GGCTGCCGGC

CCCCGCGGGC CCGGGGACCG GCGCGCCGAG CCATGCCCCG GGCTGCCGGC

GGTG

GGTG

GGCG

>Ortholog Group 142, Repeat 1

3 108

H00419704 ATGGAGCGGC TGCGAAACCT GCGTGACTTC CTGTTGGTCT ACAATCGGAT

M00102398 ---------C TGAGAAACTT GCGAGACTTC CTGTTGGTCT ACAATCGGAT

R00000351 CTGGGCGTGC TGAGAAACTT GCGAGACTTT CTGTTGGTCT ACAATCGGAT

GACAGAACTC TGCTTCCAGC GCTGTGTGCC CAGCTTGCAC CACCGAGCTC

GACAGAACTG TGTTTCCAGC GCTGTGTGCC CAGCCTGCAC CACCGAGCTC

GACAGAACTG TGCTTCCAGC TCTGTGTGCC CAGCCTGAAC CACCGAGCTC

TGGACGCT

TGGACGCT

TGGACGCT

>Ortholog Group 143, Repeat 1

3 198

M00073616 GTCCGCATGG TGAGACAATT CCATTTCCAT GGCTGGCCTG AGGTTGGCAT

R00021359 GTCCGGATGG TGAGACAATT CCATTTCCAT GGCTGGCCTG AGGTTGGAAT

H00254667 GTCCGAGTAG TGCGCCAGTT TCACTTCCAC GGCTGGCCTG AGATCGGGAT

CCCCGCTGAA GGCAAAGGCA TGATTGACCT GATTGCAGCA GTGCAGAAGA

CCCCACTGAG GGTAAAGGCA TGATTGACCT GATCGCGGCA GTGCAGAAGA

TCCCGCCGAG GGCAAAGGCA TGATTGACCT CATCGCAGCC GTGCAGAAGA

CGGGCAACCA CCCCATCACC GTGCACTGCA GCGCGGGAGC AGGGCGGACA

CAGGCAACCA CCCCATCACC GTGCACTGCA GTGCGGGAGC AGGGCGGACA

CAGGCAACCA CCCCATCACC GTGCACTGCA GTGCCGGAGC TGGGCGAACA

GGTACATTCA TAGCACTCAG TAACATTTTG GAACGAGTGA AAGCCGAG

GGTACATTCA TAGCACTCAG TAACATTTTG GAACGAGTGA AAGCCGAG

GGTACATTCA TAGCCCTCAG CAACATTTTG GAGCGAGTAA AAGCCGAG

>Ortholog Group 144, Repeat 1

3 198

H00351416 TTGGAGGAAC TACAGAAAGT AGAACGAGAG TTACAACTGA AAACTCAGCA

M00014421 CTGGAGGAGC TGCAGAAAGT AGAGCGAGAG CTACAACTGA AAACTCAGCA

R00032591 CTGGAGGAAC TGCAGAAAGT AGAGCGAGAG CTACAACTGA AAACTCAGCA

GCAGCTAAAA AAGCAGTATC TAGAGGTTAA AGCTCAAAGA ATTCAACTTT

GCAGCTTAAA AAACAGTATC TAGAAGTTAA AGCTCAACGA ATTCAGCTTT

GCAGCTTAAA AAACAGTATC TAGAAGTTAA AGCCCAGCGG ATTCAGCTGT

CTTGCCAACA CCTGGGATTA CTAACTCCTG TTGGAGTTGG AGAGCAGCTT

CTTGCCAGCA TCTGGGACTA TTTACTTCTG TTGGGGTTGG AGAGCAGCTT

CTTGCCAGCA TCTGGGACTC CTTACTTCTG TTGGGGTTGG AGAGCCGCTT

TCTGAGGGAG ACTATGCACG GTTACAGCAA GTGGATCCTG TTTTACTT

TCCGAGGGAG ACTATGCACG GTTACAGCAA GTGGATCCTG TTTTGCTT

CCTGAGGGAG ACTATGCACG GTTACAGCAA GTGGATCCTG TTTTGCTT

>Ortholog Group 145, Repeat 1

3 198

R00028825 AGTCGGCAGA TCCGGCAGTT CCACTTCCAC GGCTGGCCTG AGGTGGGCAT

M00076533 AGTCGGCAAA TCCGGCAGTT CCACTTCCAC GGCTGGCCTG AGGTGGGCAT

H00351559 AGCCGGCAGA TCCGGCAGTT CCACTTCCAT GGCTGGCCTG AAGTGGGCAT

CCCCAGCGAT GGGAAGGGTA TGATCAACAT CATTGCAGCA GTGCAGAAGT

CCCCAGCGAC GGCAAGGGCA TGATCAACAT CATTGCAGCA GTGCAGAAGT

CCCCAGTGAC GGAAAGGGCA TGATCAGCAT CATCGCCGCC GTGCAGAAGT

CAGGGAACCA TCCCATCACT GTGCACTGCA GTGCCGGGGC AGGACGGACA

CGGGGAACCA TCCCATCACT GTGCACTGCA GTGCCGGGGC AGGACGGACA

CAGGGAACCA CCCCATCACC GTGCACTGCA GCGCCGGGGC AGGAAGGACG

GGGACCTTTT GTGCCTTGAG CACAGTCCTG GAGCGCGTGA AAGCAGAA

GGAACCTTCT GTGCCTTGAG CACAGTCCTG GAACGTGTGA AAGCAGAA

GGGACCTTCT GTGCCCTGAG CACCGTCCTG GAGCGTGTGA AAGCAGAG

>Ortholog Group 146, Repeat 1

3 198

M00031640 CTTCCCACGT CAGATGTTTT CGCCGGTTGT CAGATCCCAT ATCCCAAACG

R00057821 ---------- ---------- ---------- ---------- ----------

H00370938 CTTCCTACAT CAGACGTTTT TGCCGGTTGT CAAATCCCTT ACCCAAAACG

AGAATTTTTA ACAGAAGAAG AGCCTGATGA GAAAGGAGAC AAAAAGACCG

---------- ---------- ---------- ---------- ----------

AGAATTTTTA ACGGAAGAAG AACCTGATGA CAAAGGAGAC AAAAAGAACG

GCAACAACCA CACTAACGGA ACTGGCCATC CGGGGAACCA GGACAGCGGC

---------- ---------- ---------- ---------- ----------

GCAATAACCA CACTAATGGA ACTGGCCACC CAGGGAATCA AGACAGCAGT

CACGCACAGG GGCCCCCCTT GAAAAAAGTG AGAGTTGTCC CTCCTACC

---------- ---------- ---------- ---------- --------

CACACACAGG GACCCCCGTT GAAGAAAGTG AGAGTTGTTC CTCCTACC

>Ortholog Group 147, Repeat 1

3 126

M00038369 ATGAAGAGGT GCAAATCGGA CGAGCTGGGC GAGGAGGATG GGGCTGGGAT

R00000036 ATGAAGAGGT GCAAATCGGA TGAGCTGGGC GAGGAGGATG GGGCTGGGAT

H00350718 ATGAAGAGGT GCAGATCGGA CGAGCTGGGC GAGGAGGATG GAGCTGGGCT

GGAAGACGCT GCTTGCCTTC TGCCAGGCGC GGACCTCCGG CATGGGGAGG

GGAAGACGCC GCTTGCCTTC TGCCAGGCGC GGACCTCCGG CATGGGGAGG

GGAAGATGCC GCTTCCCACC TGCCGGGCGC GGACCTCCGG CCTGGGGAGA

CCTCGAGTGC TAACTCCGCT GGCGGG

CCTCGAGTGC TAACTCCGCT GGCGGG

CCACGGGTGC TAACTCTGCT GGCGGG

>Ortholog Group 147, Repeat 2

3 198

M00038369 GAGGGCTCCA TGTTTGGCCA CGGCCTGAAG CACCTGTTTC ACAGCCGCCG

R00000036 GAGGGCTCCA TGTTTGGCCA CGGCCTGAAG CACCTGTTTC ACAGCCGCCG

H00350718 GAGGGCTCCA TGTTTGGCCA CGGTCTGAAG CACCTGTTCC ACAGCCGCCG

CAGGTCACGG GAGAGGGAGC ACCAGGCGTC TCAGGAGGCC CAGCAGCAGG

CAGGTCACGG GAGAGGGAGC ACCAGGCGTC TCAGGAGGCC CAGCAACAGG

TCGGTCTCGG GAAAGGGAGC ACCAGACGTC TCAGGATTCC CAGCAGCATG

GCCTATCCGA TCAGGACTCC CCAGATGAGA AGGAACGCTC CCCGGAGATG

GCCTATCGGA TCAGGACTCC CCAGATGAGA AGGAGCGCTC CCCGGAGATG

GTATGTCCGA CCATGACTCC CCAGATGAGA AGGAGCGCTC TCCGGAGATG

CACCGCGTCT CCTATGCTGT GTCCCTGCAC GACCTGCCTG CCCGACCT

CACCGCGTCT CCTATGCTGT GTCCCTGCAC GACCTGCCCG CGCGACCT

CATCGCGTCT CCTACGCCAT GTCCCTGCAC GACCTGCCCG CCCGGCCC

>Ortholog Group 147, Repeat 3

3 198

M00038369 AGCATGGAGG AGAAGGTGGC CTACCAGTCC TACGAGAGGG CCCGGGATAT

R00000036 AGCATGGAGG AGAAGGTGGC CTACCAGTCC TACGAGAGGG CCCGGGATAT

H00350718 AGCATGGAGG AGAAGGTGGC CTACCAGTCC TATGAGAGGG CACGGGACAT

CCAGGAGGCC GTGGAGTCCT GCCTGACCCG AGTCACCAAG CTGGAACTGG

TCAGGAGGCC GTGGAGTCTT GCCTGACCCG TGTCACCAAG CTGGAGCTGG

CCAGGAGGCC GTGGAGTCCT GCCTGACCCG GGTCACCAAG CTGGAGCTGG

TGGTGCAGTT AGAAGGTGTG GAGAACGCCA ACGCGCGCGC CCTGCTGGGC

TGGTGCAGTT AGAAGGTGTA GAGAACGCCA ACGCGCGCGC TCTGCTGGGC

TGGTACAGCT GGAGGGCGTG GAGAATGCCA ACGCGCGGGC GCTGCTGGGC

AAGTTCATCA ACGTGATCCT GGCACTCATG GCCGTGCTGC TGGTGTTT

AAGTTCATCA ACGTGATCCT GGCACTCATG GCAGTGCTGC TGGTGTTT

AAGTTCATCA ACGTGATCCT GGCGCTCATG GCCGTGCTGC TGGTGTTC

>Ortholog Group 148, Repeat 1

3 132

M00024123 ATGAACTTCC AGGCAGGCGG GGGCCAGAGT CCTAGCCTGG CGGCTCCGGG

H00380413 ATGAACTTCC AGGCGGGCGG GGGGCAGAGC CCGAGCCTGG CGGCTCCGGG

R00017827 ---------- ---------- ---------- ---------- ----------

GACCGGCGGC GGCGGCGGCG GCGGCGCGGG GGGCGGCGGG CAGTTCGGCG

GGGCGGCGGC GCTGCCGCGC AGCAGCTCGT CTGCGGCGGG CAGTTCGGCG

---------- NNNGGCGGCG GCGGCGCGGG GGGCGGCGGG CAGTTCGGCG

GCGCGGGGCC CGGAGCC--- GGGGGTGGCG GC

GCGCGGGGCC CGGGGCCGGG GGCGGCGGCG GC

GCGCGGGGCC CGGAGCC--- GGGGGTGGCG GC

>Ortholog Group 149, Repeat 1

3 198

H00256495 CCCGAACATC TCAAACTTAC AACTTTGGGT CACTTGGAAA AAGCAGTGGT

R00009564 CCCGAACATC TCAAACTTAC TACTTTGGGT CACTTGGAGA AAGCAGTGGT

M00032194 CCCGAACATC TCAAACTTAC TACTTTGGGT CACTTGGAAA AAGCAGTGGT

TCTTGAACTT ACCTTGAAGC ATGTGAAAGC ACTAACAAAC CTAATTGATA

TCTCGAGCTG ACGCTGAAGC ACGTGAAAGC ATTGACAAAC CTAATTGATA

TCTGGAGCTT ACGTTGAAGC ACGTGAAAGC ATTGACAAAT CTAATTGATA

AAATCATTGC CCTGCAGAGT GGTTTACAAG CTGGTGAGCT GTCAGGGAGA

AAATCATGGC CCTGCAGAGC GGTTTACAAG CTGGTGATCT GTCGGGAAGA

AAATCATTGC CCTGCAGAGC GGTTTACAAG CTGGTGATTT GTCGGGAAGA

AATGTCGAAA CAGGTCAAGA GATGTTCTGC TCAGGTTTCC AGACATGT

AATATTGAGG CAGGACAAGA AATGTTCTGC TCCGGTTTCC AGACCTGT

AATCTCGAGG CAGGGCAAGA AATGTTCTGC TCAGGTTTCC AGACTTGT

>Ortholog Group 14, Repeat 1

3 183

M00043874 AGCTCTCAGG AAAGAGCACC ATATGTGCAA AAAGCCAGAG ATAACAGGGC

R00010349 ---------- ---------- ---------- ---------- ----------

H00347325 AGCTCACAAG AAAGAGCACC ATATGTGCAA AAAGCCAGAG ATAACAGAGC

TGCTTTACGC ATAAATAAAG TTCAGATGTC AAACGATTCT ATGAAGAGGG

---------- ---------- ---------- ---------- ----------

TGCTTTACGC ATTAATAAAG TACAGATGTC AAATGATTCC ATGAAAAGGG

ACAGCATCGA TCCCAGCTCA CGCATCGATT CGGATCTTTT TAAAGATCCT

---------- ---------- ---------- ---------- ----------

ATAGCATTGA TCCCAGCTCT CGTATTGATT CGGAGCTTTT TAAAGATCCT

TTAAAGCAGA GAGAATCAGA GCATGAACAG GAA

---------- ---------- ---------- ---

TTAAAGCAAA GAGAATCAGA ACATGAACAG GAA

>Ortholog Group 14, Repeat 2

3 204

M00043874 AAGTTTAGAC AGCAAATGCG TCAGAAAAGT AAGCAACAAG CTAAAATTGA

R00010349 ---------- ---------- ---------- ---------- ----------

H00347325 AAATTTAGAC AGCAAATGCG TCAGAAAAGT AAGCAGCAAG CTAAAATTGA

AGCCACACAG AAGCTGGAAC AAGTGAAGAA TGAGCAGCAG CAGCAGCAGC

---------- ---------- ---------- ---------- ----------

AGCCACACAG AAACTTGAAC AGGTGAAAAA TGAG------ ----------

AGCAGCTTGC TTCTCAGCAC CTTCTGGTAG CACCTGGTTC AGATACTCCA

---------- ---------- ---------- ---------- ----------

-----TTTGG TTCTCAGCAT CTTCTGGTGC AGTCTGGTTC AGATACACCA

AGTAGTGGAG CACAGAGTCC CTTGACACCT CAGGCTGGCA ATGGGAATGT

---------- ---------- ---------- ---------- ----------

AGTAGTGGGA TACAGAGTCC CTTGACACCT CAGCCTGGCA ATGGAAATAT

GTCT

----

GTCT

>Ortholog Group 14, Repeat 3

3 198

M00043874 CCAACCTCAG GAGGAACTGA TACACAGAAC ACTGTAAACA TGTCTCAAGC

R00010349 ---------- ---------- ---------- ---------- ----------

H00347325 CCAACTTCAG GAGTAACTGA TACACAGAAT ACTGTAAATA TGGCCCAAGC

TGACACAGAG AAACTGAGAC AGCGGCAGAA ACTGCGTGAA ATCATTCTCA

---------- ---------- ---------- ---------- ----------

AGATACAGAG AAATTGAGAC AGCGGCAGAA GTTACGTGAA ATCATTCTCA

AGAAGATTGC TAGTCGCCAG GAGAAGGGGC CTCAGGATAC AGCAGTAGTA

---------- ---------- ---------- ---------- ----------

AGAAGATTGC AGGTCGACAG GAGAAGGGGT CACAGGACTC ACCCGCAGTG

CCTCACCCAG TGCCCCTTCC ACACTGGCAG CCAGAGAGCA TCAACCAG

---------- ---------- ---------- ---------- --------

CCTCATCCAG GGCCTCTTCA ACACTGGCAA CCAGAGAATG TTAACCAG

>Ortholog Group 14, Repeat 4

3 198

M00043874 ATTCCTCAAA CATTAGCCCA GCAGAACAGA GAGAGGCCCC TCCTTCTAGA

R00010349 ------CAGC AAATGCGTCA GAAAAGTAAG CAGCAAGCTA AAATTGAAGC

H00347325 ATTCCTCAAA CATTAGCACA GCAGAATAGA GAGAGGCCCC TTCTTCTAGA

GGAACAGCCT CTGCTTCTAC AAGATCTTTT GGATCAAGAG AGGCAGGAGA

CACACAGAAG CTGGAACAAG TGAAGAATGA ACAGCAGCAG CAGCAGCAGC

AGAACAGCCT CTACTTCTAC AGGATCTTTT GGATCAAGAA AGGCAAGAAA

GACAAATGCA AGCCATGATT CGTCAGCGGT CAGAACCATT CTTCCCTAAC

AACAACAACA ACAACTTGCT TCTCAGCACC TT-------- ----------

GACAGATGCA AGCCATGATT CGTCAGCGAT CAGAACCGTT CTTCCCTAAT

ATTGATTTTG ATGCTATTAC AGATCCTATA ATGAAAGCGA AAATGGTA

CTAGATTTCG ATGCAATAAC AGATCCTATA ATGAAAGCGA AAATGGTA

ATTGATTTTG ATGCAATTAC AGATCCTATA ATGAAAGCCA AAATGGTG

>Ortholog Group 14, Repeat 5

3 207

M00043874 CATGTTACTG AGCAGCAGAG CATGGTTCAG AAACAGCTTG AGCAGATTCG

R00010349 CATGTTACTG AGCAGCAGAG CATGGTTCAG AAACAACTTG AGCAGATTCG

H00347325 CATGTTACTG AACAGCAAAG CATGGTTCAG AAACAGCTAG AACAGATTCG

GAAACAACAG AAAGAGCATG CTGAGCTGAT TGAAGATTAT CGGATCAAAC

TAAACAACAG AAAGAGCATG CTGAGCTGAT TGAAGATTAC CGGATCAAGC

TAAACAACAG AAAGAACATG CTGAATTGAT TGAAGATTAT CGGATCAAA-

AGCAGCAGTG TGCCCTAGCC CCTCCCATCC TCATGCCAGG GGTTCAGCCC

AGCAGCAGTG CGCCCTAGCC CCTCCCATCC TCATGCCAGG GGTTCAGCCC

--------TG TGCAATGGCC CCACCTACCA TGATGCCCAG TGTCCAGCCC

CAGCCACCTC TAGTTCCAGG TGCCACTTCA CTTACCATGA GCCAACCCAA

CAGCCACCTC TAGTTCCAGG TGCCAGCCCA CTCACCATGA GCCAACCCAA

CAGCCACCCC TAATTCCAGG TGCCACTCCA CCCACCATGA GCCAACCCAC

CTTTCCC

CTTCCCC

CTTTCCC

>Ortholog Group 150, Repeat 1

3 171

R00027738 ATGGCTAACA ACAGCCCCGC GCTGACCGGC AACTCGCAAC CGCAGCACCA

M00029480 ATGGCCAACA ACAGCCCCGC GCTGACCGGC AACTCGCAAC CGCAGCACCA

H00359042 ATGGCTAACA ACAGCCCCGC GCTGACAGGC AACTCGCAGC CGCAGCACCA

GGCGGCTGCG GCCGTGGTCT GT---GGCGG CGGCGGCGCC ACCAAGCCGG

GGCGGCCGCG GCCGTGACCT GTGGCGGCGG CGGCGGCGCC ACCAAGCCGG

GGCGGCTGCA GCTGCGGCTT GC---GGCGG CGGCGGCGCT ACCAAGCCGG

CGGTGTCGGG CAAGCAGGGC AATGTGCTGC CGCTGTGGGG CAATGAGAAG

CGGTGTCGGG CAAGCAGGGC AATGTGCTGC CGCTGTGGGG CAACGAGAAG

CGGTCTCCGG CAAGCAGGGC AATGTGCTCC CGCTCTGGGG CAACGAGAAG

ACCATGAACC TCAACCCCAT G

ACCATGAACC TCAACCCCAT G

ACCATGAACC TCAACCCCAT G

>Ortholog Group 151, Repeat 1

3 198

R00020729 ---------- ---------- ---------- ---------- ----------

H00308927 GCTGCCCACG ATGAGCAGAA GAAACTAGCT GCCTCTCAGA TTGAGAAACA

M00047567 GCTGCCCACG ATGAGCAGAA GAAGTTGGCT GCATCTCAGA TAGAGAAACA

---------- ---------- ---------- ---------- ----------

GCGTCAGCAA ATGGAGCTGG CCAAGCAGCA ACAAGAACAA ATTGCAAGAC

GCGTCAGCAG ATGGAGCTGG CCAAGCAGCA ACAGGAGCAG ATCGCGAGGC

---------- ---------- ---------- ---------- ----------

TTCTACAGCA ACAACACAAA ATCAATTTGC TCCAGCAACA GATCCAGGTT

TTCTGCAGCA ACAACACAAA ATCAACTTGC TTCAGCAACA GATCCAGGTT

---------- ---------- ---------- ---------- --------

CAAGGTCAGC TGCCGCCATT AATGATTCCC GTATTCCCTC CTGATCAA

CAAGGTCAGC TGCCGCCATT GATGATTCCC GTGTTCCCTC CTGACCAG

>Ortholog Group 152, Repeat 1

3 231

H00256972 CGTCAGGGAC CACCTTTGGG AGGACAGCAA TCTCAACCCT CTGCTGGTGA

R00048827 AATCAGAAAC CACCTCCACC AGGATTCCCA CCAAGACCAC CTGCT-----

M00073689 AATCAAAGGC CACCTCCTTC AGGGTTCCAA CCAAGACCGC CTGTT-----

TGGGAACCAG AATGATGGCC CTCAGCAGGG ACCACCCCAA CAAGGAGGCG

---------- ---AATGGGA GCCAGCAAGG CCCACCCCCA CAAGGAGGCG

---------- ---AATGGGA GCCAGCAAGG CCCACCACCA CCAGGAGGCG

GTCCACCACC TCCTCAGGGA AAGCCACAAG GACCACCCCA ACAGGGAGGC

GCCCACCCCC ACCAGGAGGC CCACAGCAGA AACCCCCTCA GCCTGGAAAC

GCCCACCACC ACCAGGAGTA CCACAGCCGA GACCCCCT-- ----------

CAT------C CCCCTCCTCC TCAAGGA--- ---------- ----------

CAACAAGGCC CACCCCCACC AGGAGGCCCA CAACAGAAAC CAACTCAGCC

---CAAGGCC CACCACCACC AGGAGGCCCA CAGCAGAGAC CC--------

----AGGCCA CAAGGACCAC CCCAACAGGG A

TGAAAAGCCC CAAGGCCCAC CCCCACCAGG A

-------CCT CAAGGCCCAC CACCACCAGG A

>Ortholog Group 153, Repeat 1

3 198

R00008550 GCACCAGCTT ACACTCAACT GCAGCCACAC CAACTCCTCC CACAGCCGTC

M00101690 GCACCAGCTT ATGCTCACCT GCAGTCACAC CAGCTCCTCC CACAGCCGCC

H00391440 GCACCAGCCT ATGCTCAGCT GCAGCCACAC CAGCTCCTCC CACAGCCATC

ATCAAAGCAT CCACAGCCCC AGTTTGTGGC CCAGCAGCAA CCACAGCCA-

AGCGAAGCAC CCACAGCCCC AGTTTGTGGC CCAACAGCAA CCACAGCCA-

CTCAAAGCAC CTGCAGCCCC AATTTGTGAT CCAGCAGCAG CCACAGCCAC

---------- ----CCACGG CCTACACCCC AAGTCCAGTC CCAACCCCAG

---------- ----CCACGA CCTGCACCCC AAGTCCAGTC CCAACCCCAG

CGCCGCCCCA GCAGTCACGG CCTGTGCTCC AAGCTGAGCC CCACCCCCAG

CTTGCCGCAG TCTCTCCAAG CCTGGCCCTA CAGCCCAGTC CAGAAGGC

CTTGCCTCAG TCTCCCCAAG CCTGGCCCTA CAGTCCAGTC CAGAAGAC

CTCGCCTCAG TCTCTCCAAG CGTGGCCCTC CAGCCCAGCT CAGAGGCC

>Ortholog Group 154, Repeat 1

3 108

R00002976 ACCAAATTAG TGACTGCTCC TGTAGCTTGT GGGGCCGTCA TGGTGCCAAG

H00370723 ACCAAATTAG TGACTGCTCC TGTAGCTTGT GGGGCAGTCA TGGTACCTAG

M00031148 ACCAAATTAG TGACTGCTCC TGTAGCTTGT GGGGCCGTCA TGGTACCAAG

TACCATGCTT ATGGGTCAGG TAGTGACTGC CTATCCTACC TTTGCCACAG

TACTATGCTT ATGGGCCAGG TGGTGACTGC ATATCCTACT TTTGCTACAT

TACCATGCTT ATGGGTCAGG TGGTGACTGC CTATCCTACC TTCGCCACAG

CACAGGCC

CACAGACA

CACAGACA

>Ortholog Group 154, Repeat 2

3 102

R00002976 TCAGTAACAA GTTCCCAGGA CCAGCCGCAT CCTTCAGTTC AGCAGCCA--

H00370723 TCAGTAACGA GCTCCCAGGA GCAGCAGCTC ACTTCAGTTC AGCAACCATC

M00031148 TCGGTAACAA GTTCCCAGGA ACAGCAGCTT CCTTCAGTTC AGCAGCCAGC

----GCTCAG CTGACCCAGC CACCACAACA GTTCTTACAG ACATCTCGGT

TCAGGCTCAG CTGACCCAGC CACCGCAACA ATTTTTACAG ACTTCTAGGT

TCAGGCCCAG CTGGGCCAGC CACCACAGCA GTTCTTACAG ACATCTAGGT

TG

TG

TG

>Ortholog Group 154, Repeat 3

3 144

R00002976 CATGGGAATC CTTCGACACA GCTCATCCTC TCCGCTGCCT TTCCACTACA

H00370723 CATGGGAATC CCTCAACTCA ACTCATTCTC TCTGCTGCAT TTCCTCTACA

M00031148 CACGGGAATC CTTCGACACA GCTCATCCTC TCTGCTGCCT TTCCACTACA

ACAGAGCACT TTCCCTCCTT CGCACCACCA GCAACAC--- ---CTCCATC

ACAGAGCACC TTCCCTCAGT CACATCACCA GCAACATCAG TCTCTCAGCC

ACAGAGCACT TTCCCTCCTT CGCACCACCA GCAACACCAG CCTCTTCCTC

GGCACAGGAC TGACAGCCTG ACTGACCCTT CCAAGGTCCA GCCA

GGCACAGGAC TGACAGCTTG CCCGACCCTT CCAAGGTTCA ACCA

GGCACAGGAC TGACAGCCTG ACTGACCCTT CCAAGGTCCA GCCA

>Ortholog Group 155, Repeat 1

3 198

M00110900 TTGAAAGCCA AGTACACCTT GCTACACGAT ACAGTGGTGA GCACGCAGGA

R00017374 ---------- ---------- ---------- ---------- ----------

H00285871 TTAAAAGCCA AGTATACCTT GCTGCATGAC GCCGTGATGA GCACACAAGA

GTCTGAGGTG CAGCTACTGG AGAACGCCAA GCGCTTCACG GAGCAGATCG

---------- ---------- ---------- ---------- ----------

GTCAGAGGTC CAACTGCTAC AGAATGCCAA ACGTTTCACT GAGCAAATAT

TCTGTCTGCA GCAGGCCGAA GACTTCCCCA ACGTCTTCAC CACTGAGGTC

---------- ---------- ---------- ---------- ----------

TTCACCTGCA GCAAGCTGAT AATTTTCCAG AAGCATTCTC CACGGAGGTC

TGCAAACTTC GGGAGCAGCT GCTCAAGTAC CAAAATGAAT ACACGGCA

---------- ---------- ---------- ---------- --------

TCCAAAATGA GAGAACAACT TCTCAAGTAT CAAAATGAAT ATAATGCA

>Ortholog Group 156, Repeat 1

3 243

H00257821 ATAGTAAAGC AAGAACAACC GGACAAGTTC CAAATACAGC CATTGCCACA

M00106924 ATAGTCAAAC AAGAGCAGCC GGACAAGTTC CAGATACAGC CATTGTCACA

R00046694 ATAGTCAAGC AAGAGCAGCC GGACAAGTTC CAGATACAGC CATTGTCACA

ATCTGAAAAC AAACTACAAA CAGCACAGCA GCAACCACTA ----------

GTCTGAAAAC AAACTACAAA CAGCACAGCA GCAGCCACTA CAGCCACTG-

GTCTGAAAAC AAACTACAAA CAGCACAGCA GCAGCCACTA CAGCCACTGC

---------- ---------- ---------- -----CAGCA ACTATACCAC

---------- ---------- CAGCAGCAGC AACCGCAGCA ACCGCAGCAG

AGCAGCAGCA ACAGCAACAG CAGCAACAGC AGCAGCAGCA GCAGCAGCAG

CACCACCACG CCCAGCAGTC AGCTGCAGCC TCTCCCAACC TGACTGCTTC

CAGCAGCACG CCCAGCAGTC AGCAGCAGCA CCGCCTAGTC TAACTGCATC

CAACAGCACG CCCAGCAGTC A---GCAGCC CCGCCTAGTC TGACTGCATC

ACAGAAGACT GTAACTACAG CTTCTATGAT TACCACAAAG ACA

ACAGAAGACT GTAACTACAG CTTCTATGAT TACCACAAAG ACA

ACAGAAGACT GTAACTACAG CTTCTATGAT TACCACAAAG ACA

>Ortholog Group 157, Repeat 1

3 198

R00052146 AAGTCCACAC TGTCAATGTC TCCACGGCAA CGGAAGAAGA TGACAAGGAC

H00257905 AAGTCCACTT TGGCAATGTC TCCACGGCAA CGGAAGAAGA TGACAAGGAT

M00023133 AAGTCCACCT TGTCAATGTC TCCACGGCAA CGGAAGAAGA TGACAAGGAC

TACACCCACC ATGAAAGAGC TCCAGACAAT GGTTGAACAT CACCTAGGGG

CACACCCACA ATGAAAGAGC TCCAGATGAT GGTTGAACAT CACCTGGGGG

CACACCCACC ATGAAAGAGC TCCAGACGAT GGTTGAACAT CACCTAGGGG

GGGAAGAACC TGAGGGAGCC ACTGAGAGCA CAGGGAACCA GGAGTCCTGC

GAGAGGAACC TGAGGGGGCC GCTGAGAGCA CAGAAACCCA GGAGTCCCGC

GGGAAGAACC TGAGGGAGCC ACTGAGAGCA CAGGGAACCA GGAGTCCTGC

CCACCTGGGA TCCCAGACAC AGGCTCAGCG TCAAGGCCAG ATACCTCG

CCACCTGGGA TCCCAGACAC AGAAGTGGAG TCAAGGCTGG GCACCTCT

CCACCTGGGA TCCCAGACAC AGGCTCGGCG TCAAGGCCAG ATACCCCC

>Ortholog Group 158, Repeat 1

3 198

M00097773 CCTGCAGATG GAATCAGACT CTTTAATGCA TTAAAAGGCC GGATGGTGCG

H00257915 CCTGCAGATG GAATCAGACT TTTTAATGCA TTAAAAGGCC GGATGGTGCG

R00037251 CCTGCAGATG GAATCCGGCT CTTCAACGCA TTGAAAGGCC GGATGGTGCG

GCCAAGGCTA ACCATTTATG TCTGCCAGGA GTCGTTGCAG TTGAGGGAGA

TCCAAGGTTA ACCATTTATG TTTGTCAGGA ATCACTGCAG TTGAGGGAGA

TCCAAGGCTG ACCATTTATG TCTGTCAGGA GTCGTTGCAG TTGAGGGAGA

AGCAGGAGGA TGGGGATTCA AACGGTACTT TCTTCGTGTA CCATGCTATC

AGCATGAGGA TGGAGACTCA AATGGTACTT TCTTCGTTTA CCATGCTATC

AGCAGGAGGA CGGAGATTCA AATGGTACTT TCTTTGTGTA CCATGCTATC

TATCTCGAGG AGCTGACAGC TGTCGAACTC ACAGAAAAAA TTGCTCAG

TATCTAGAAG AACTAACAGC TGTTGAATTG ACAGAAAAAA TTGCTCAG

TATCTAGAGG AGCTGACAGC TGTTGAATTA ACAGAAAAAA TTGCTCAG

>Ortholog Group 159, Repeat 1

3 198

H00258201 TACACCCCGC AGGCGGCCCG TGAAGTGCGC ATCATGCAGT TCTGCCACAC

M00014922 TACACCCCAC AGGCAGCAAG GGATGTACGC ATCATGCAGT TCTGCCACAC

R00021935 TACACCGCAC AGGCAGCAAG GGAAGTACGC ATCATGCAGT TCTGTCACAC

GCTGCGGGAA TTTGCGCTTG AGTATCGGAC TTGCCGGGAA CGAGTGCTAA

ACTGAGAGAG TTTGCCCTTG AGTATCGGAC TTGTCGGGAA CGGGTACTGA

GCTCAGAGAG TTTGCACTTG AGTATCGGAC TTGTCGGGAG CGGGTACTGA

AGCAGGCCAC ATACCGTGAG CGCAACAAGA CCCGGGGACG CATGATCACC

AGCGGGCTAC ATACCGTGAG CGCAACAAGA CCCGTGGTCG CATGATTACC

AACGGGCTAC ATACCGCGAA CGCAACAAGA CCCGGGGTCG CATGATTACT

GAGACAGAGA AGTTCTCAGG TGTGGCTGGG GAAGCCCCCA GCAACCCC

GAGACAGAGA AGTTCTCAGG TGTGGCTGGG GAGGCCCCCA ATAACCTG

GAGACAGAGA AGTTCTCGGG TGTGGCTGGG GAGGTCCCCA GTAACCTG

>Ortholog Group 15, Repeat 1

3 198

H00354370 GACTCTTCAG GAGCTACTGT AGGCCTTTTT GACTACAATT CCCAGCAGCA

M00106752 GATTCTTCAG GAGCTACTGT AGGCCTTTTT GACTACAATT CCCAACAGCA

R00008359 GATTCTTCAG GAGCTACTGT AGGCCTTTTT GACTACAATT CCCAACAGCA

GCTCTTTCAG AGGACTAATG CACTAACAGT TCAACAGTTA ACTGCAGCTT

GCTCTTTCAG AGGACTAGTG CACTAACAGT TCAGCAGTTA ACTGCAGCTT

GCTCTTTCAG AGGACTAATG CACTAACAGT TCAGCAGTTA ACTGCTGCTT

ATGCATTAGC AGCAGCTCAG CAGCCACATA TAGCTGGTGT ATTCTCAGCA

ATGCATTAGC AGCAGCTCAG CAGCCACATA TAGCTGGTGT ATTCTCAGCA

ATGCATTAGC AGCAGCTCAG CAGCCGCATA TAGCTGGTGT ATTCTCAGCA

GGCCTTGCTC CAGCTGCATT TGTGCCAAAT CCATACATTA TTAGTGCT

GGCCTTGCTC CAGCTGCATT TGTGCCAAAT CCATATATTA TTAGTGCT

GGCCTTGCTC CAGCTGCATT TGTGCCAAAT CCATATATTA TTAGTGCT

>Ortholog Group 15, Repeat 2

3 207

H00354370 GCAGCACAA- --GCTGCAGC AGCAGCAGCA GCTGGAGGAA CTGCAAGTAG

M00106752 GCAGCACAAG CTGCAGCAGC AGCAGCAGCA GCTGGAGGAA CTGCAAATAG

R00008359 GCAGCACAAG CTGCAGCAGC AGCAGCAGCA GCTGGAGGAA CTGCAAATAG

CCTTACAGGC AGCACAAATG GTCTGTTTCG GCCAATTGGC ACTCAGCCAC

TCTTACAGGC AGCACAAATG GTCTGTTTCG GCCAATTGGC ACTCAGCCAC

TCTTACAGGC AGCACAAACG GTCTGTTTCG GCCGATTGGC ACTCAGCCAC

CA------CC AAGCACTAAT CTGCAATCTA ATTCATTTTA TGGAAGCAGT

CACAGCAGCC AAGCACTAAC CTGCAGTCTA ATTCATTTTA TGGGAGCAGC

CG---CAGCC AAGCACTAAC CTGCAGTCTA ATTCGTTTTA TGGGAGCAGC

TCTTTGACTA ATAGCTCCCA GAGTAGTTCT TTATTTTCTC ATGGACCTGG

TCTTTGACTA ACAGCTCCCA GAGCAGCTCT TTATTCTCTC ATGGACCTGG

TCTTTGACTA ACAGCTCCCA GAGCAGCTCT TTATTTTCTC ACGGACCTGG

TCAACCT

CCAACCT

CCAACCT

>Ortholog Group 160, Repeat 1

3 198

M00035445 GGGTTTCTTC CTCAGCCAAC TCCTCTGTCT GTTAAAAAAC TGAGGTGTAA

H00393662 GGATTTCTTC CTCAGCCAGT TCCTCTTTCT GTTAAAAAAC TGAGGTGTAA

R00033308 GGGTTCCTTC CTCAGCCAAC TCCCCTATCT GTTAAAAAAC TGAGGTGTAA

CCAGGATTAC GCGGGCTGGA ACAGACCTAG AGTGCCCCTT TCCTCCCATC

CCAGGATTAC ACTGGCTGGA ATAAACCAAG AGTGCCCCTT TCCTCTCACC

CCAGGATTAC ACAGGCTGGA ACAAACCTAG AGCGCCCCTT TCCTCTCACC

TGCAGGGATT CTCCAACTTG GGAAATACAT GCTATATGAA TGCTATCTTA

TGCAGGGCTT CTCCAATTTG GGAAATACCT GCTATATGAA TGCTATTCTA

TGCAGGGATT CTCCAATTTG GGAAATACAT GCTATATGAA TGCTATCTTA

CAATCTCTGT TTTCACTCCA GTCATTTGCA AATGATTTGC TTAAACAA

CAATCTCTAT TTTCACTCCA GTCATTTGCA AATGACTTGC TTAAACAA

CAATCTCTGT TTTCACTCCA GTCATTTGCA AATGATTTAC TTAAACAA

>Ortholog Group 161, Repeat 1

3 204

M00123765 GCGGCCGCCC TGAGGAGCCA CCGGCCGCAG ACTACGCTGC GAGCCGCCGC

H00367923 GTGGCCACCC TTCGGAACCA CCGGCCTCGG ACGGCCCAGC GGGCTGCTGC

R00060229 GCGGCCGCCC TGAGGAGCCA CCGGTCGCAG ACTACAGTGC GAGCCACGGC

CCAGGTTCTG GGAAATTCTG GCTTGTTTAA CAAGCATGGA CTACAAGTG-

TCAGGTTCTG GGAAGTTCTG GATTGTTTAA TAACCATGGA CTCCAAGTA-

CCAGGCTCTG GGAAGTTCTG GATTGTTTAA CAAGCATGGA TTCCAAATGC

-----AGGAC CCTTTCGCTG CATGAATACT TGAGTATGGA GTTATTGCAG

-----AGGAA TCTCTCACTA CATGAATACA TGAGTATGGA ATTATTGCAA

AGCAGAGGAG TCTCTCACTG CATGAATACT TGAGTATGGA ATTACTGCAA

GAAGCGGGTG TCTCTGTTCC CAAAGGCTTC GTGGCAAAGT CATCCGATGA

GAAGCTGGTG TCTCCGTTCC CAAAGGATAT GTGGCAAAGT CACCAGATGA

GAAGCGGGCG TCTCTGTCCC CAAAGGCTTC GTTGCAAAGT CATCAGATGA

AGCT

AGCT

AGCT

>Ortholog Group 162, Repeat 1

3 201

M00001536 CGCTACAAGA TGAAGCGCCA GGCTAAGGAC AAGGCGGCGC AGCAACAACT

H00346879 CGCTACAAAA TGAAGCGCCA GGCCAAGGAC AAGGCGGCGC AGCAGCAACT

R00011453 CGCTACAAGA TGAAGCGCCA GGCGAAGGAC AAGGCGGCGC AGCAGCAACT

GCAGCAGGAC AGCGGCGGCG GCGGAGGCGG CGGTGGCGGT GCGGGATGCC

GCAGCAGGAC AGC---GGCG GCGGCGGGGG CGGCGGGGGC ACCGGGTGCC

GCAGCAGGAC AGCGGCGGCG GCGGAGGCGG CGGCGGCGGC GCGGGTTGCC

CGGCTCAGCA GCAGTCGCCG CGCCGGGTGG CCGTGCCGGT CCTAGTCAAA

CGGCTCAGCA GCAGTCGCCG CGACGCGTGG CGGTGCCGGT CCTGGTGAAA

CGGCTCAGCA GCAGTCGCCG CGCCGGGTGG CCGTGCCGGT CCTGGTGAAA

GACGGCAAAC CCTGCCAGGC GGGCGCCCCT GCCCCGGGAG CCGCAAGCCT

GACGGCAAAC CGTGCCAGGC GGGTGCCCCC GCGCCGGGCG CCGCCAGCCT

GACGGCAAAC CCTGCCAAGC GGGCGCCCCT GCACCGGGAG CCGCCAGCCT

G

A

G

>Ortholog Group 163, Repeat 1

3 198

M00018521 CACTTAAAGA CACATGGGCA GAGCCAAAGT ATCAACTGTA ACACGTGCAA

R00052564 CACTTAAAGA CACATGGGCA GAGCCAAAGT ATCAACTGTA ACACGTGTAA

H00258963 CACTTAAAGA CTCATGGGCA GAGCCAAAGT ATCAACTGTA ATACATGTAA

ACAAGGCATC AGCAAAACGT GCATGAGTGA GGAGACCAGC AATCAGAAGC

ACAAGGCATC AGCAAAACTT GCATGAGTGA GGAGACCAGC AATCAGAAGC

ACAAGGCATC AGTAAAACAT GCATGAGTGA AGAGACCAGT AACCAAAAGC

ATGTGACAAG CTGGCCAGGG AAGCAGGTAG AGACACTGAG ACTGTGGGAA

ATGTGACGAG CTGGCCAGGG AAGCAGGTAG AGACACTGAG ACTATGGGAA

ATGTGACAAG CTGGCCAGGG AAGCAAGTAG AAACACTGAG ACTGTGGGAA

GAAGCTGTCA AAGCAAGAAA GAAAGAAGCT GCCAACCTGT GCCAAACC

GAAGCTGTCA AAGCGAGAAA GAAAGAAGCT GCCAACCTGT GCCAAACC

GAAGCTGTTA AAGCAAGGAA GAAAGAAGCT GCTAACCTGT GCCAAACC

>Ortholog Group 164, Repeat 1

3 99

M00040505 CCGCTCCGTC CCACCCCGGT GGTGGCGCCC TCAGAAGTCC CGGCTGGCTT

H00355870 CCGCTTCGAC CCACCCCAGT GGTGGCGCCC TCCGAAGTCC CGGCTGGCTT

R00003155 CCGCTCCGTC CCACCCCGGT GGTGGCGCCC TCAGAAGTCC CGGCTGGATT

CCCTCAGCGG CTGTCTCCGC TCTCTGCCGC CTTCCACCAG CATCTCCCG

CCCGCAGCGG CTGTCTCCGC TCTCAGCCGC CTACCACCAC CATCACCCG

CCCTCAGCGG CTGTCTCCGC TCTCTGCCGC CTACCACCAG CATCTCCCG

>Ortholog Group 164, Repeat 2

3 99

M00040505 CCCCCGCCTC CACCCCGGGC GGTCTCCCTG CAGCCACCGA CTTCGGGGAC

H00355870 CCTCCGCCTC CGCCCCGGGC TGGCGCCCTG CAGCCCCCGG CCTCGGGGAC

R00003155 CCCCCGCCTC CACCCCGGGC GGGCTCCCTG CAGCCGCCGA CTTCGGGGAC

GCGGGTGGTC ------CCCC ACCATAGCGG CTCCGCCCCT GCCCCCTCC

GCGAGTGGTT CCGAACCCCC ACCACAGTGG CTCTGCCCCG GCCCCCTCC

GCGGGTGGTC ------CCCC ACCATAGCGG CTCCGCCCCT GCCCCCTCC

>Ortholog Group 165, Repeat 1

3 198

M00043616 CGAGAGACAT TGGCAGCAGA GACAGGCCTC AGCGTGCGTG TGGTCCAGGT

H00362573 CGAGAGACAC TGGCAGCTGA GACGGGCCTC AGTGTGCGCG TGGTCCAGGT

R00022953 CGAGAGACAC TAGCCGCAGA GACAGGCCTC AGTGTGCGTG TGGTCCAGGT

CTGGTTTCAG AACCAAAGAG CAAAGATGAA GAAGCTGGCC CGGAGACACG

CTGGTTTCAG AACCAAAGAG CAAAGATGAA GAAGCTGGCG CGGCGGCACG

CTGGTTTCAG AACCAAAGAG CAAAGATGAA GAAGCTGGCC CGGAGGCACG

AGCAGCAGAA CTCCCAGCGG CTGGGCCAAG AGGTTCTGTC AAGCCGCATG

AGCAGCAGAA CTCCCAGCGG CTGGGCCAGG AGGTCCTGTC CAGCCGCATG

AGCAGCAGAA CTCCCAGCGG CTGGGCCAAG AGGTTCTGTC AAGCCGCATG

GAGGGCATGA TGGCCTCCTA CACGCCGCTG GCCCCTCCGC AGCAGCAG

GAGGGCATGA TGGCTTCCTA CACGCCGCTG GCCCCACCAC AGCAGCAG

GAGGGCATGA TGGCCTCCTA CACGCCGCTG GCCCCTCCGC AGCAGCAG

>Ortholog Group 166, Repeat 1

3 198

M00097885 ---ATGCCCC TCCACGTGAG CCTCGCTAAC GGCAACCGCG ACCTGGACTA

H00367207 ACGATGCCCC TCAACGTTAG CTTCACCAAC AGGAACTATG ACCTCGACTA

R00059721 ---ATGCCCC TCCACGTGAG CCTCGCCAAC GGCAACCGCG ACCTGGACTA

TGACTCGGTG CAGCCATACT TCATGTGCGA CGATGAGGAG GAGGACGTGC

CGACTCGGTG CAGCCGTATT TCTACTGCGA CGAGGAGGAG AACTTCTACA

CGACTCGGTG CAGCCATACT TCATGTGCGA CGATGAGGAG GAGGACGTGC

CG------CA GCCGCCAGCG CCCAGCGAGG ACATCTGGAA GAAATTCGAG

GCGAGCTGCA GCCCCCGGCG CCCAGCGAGG ATATCTGGAA GAAATTCGAG

CG------CA GCCGCCAGCG CCCAGCGAGG ACATCTGGAA GAAATTCGAG

CTGCTGCCCA CGCCGCGCCC GTCCCCGGGC CACGCCGGGC TCTACTCG

CTGCTGCCCA CCCCGCCCCT GTCCCCTAGC CGCCGCTCCG GGCTCTGC

CTGCTGCCCA CGCCGCGCCC GTCCCCGGGC CACCCGGGCC TCTACTCG

>Ortholog Group 167, Repeat 1

3 198

M00074490 TTGAAGCTAG AGCGCTTGGA ACAGAGGAGA AAAGCAGAGA AAGACTCAGA

H00260210 TTGAAACTCG AACGTTTAGA GCAAAGGAGG AAAGCAGAAA AGGACTCAGA

R00025232 TTGAAGCTGG AGCGCTTGGA ACAGAGGAGA AAAGCAGAGA AAGACTCAGA

GCGAGATGAG CTATATGCCC AGTGGGGGAA AGGGCTTGCC CAGAGCCGGA

GAGAGATGAG CTGTATGCCC AGTGGGGAAA AGGGCTTGCC CAGAGCCGGA

GCGAGATGAG CTGTATGCTC AGTGGGGGAA AGGGCTTGCC CAGAGCCGGA

ATGTAGAAGA TGCAATGAAG GAGATGCAGA AGCCTCTGGC CCGCTACATA

ATGTGGAGGA TGCAATGAAA GAGATGCAAA AGCCTCTGGC CCGCTATATT

ATGTAGAAGA CGCAATGAAG GAGATGCAGA AACCTCTGGC TCGCTACATA

GATGATGAAG ATCTGGATCG GATGCTGAGA GAACAAGAAA GAGAAGGG

GATGACGAAG ATCTGGATAG GATGCTAAGA GAACAGGAAA GAGAGGGG

GATGATGAAG ATCTAGATCG GATGCTGAGA GAGCAAGAAA GAGAAGGG

>Ortholog Group 168, Repeat 1

3 198

H00282441 ATGAACCAGA GAATCAGTCA GAGTGCTCCA GTGAAACAGC CACCACCCCT

R00034369 ATGAACCAGA GGATCACTCA GAGTGCTCCA GTGAAGCAGC CCCCACCCTT

M00069554 ATGAACCAGA GGATCACTCA GAGTGCTCCA GTGAAGCAGC CCCCACCCTT

GGCTCCCCAG AGCCCACAGG GAGGCGTCAT GGGTGGCAGC AACTCCAACA

GGCTCCCCAG AGCCCACAGG GAGGCGTCCT GGGTGGAGGC AGCTCAAACA

GGCTCCCCAG AGCCCACAGG GAGGCGTCCT GGGTGGAGGC AGTTCCAACA

TGCGACTGCA GCAACTGCAG ATGGAGAAGG AGAGGCTGCG GCTGAAACAG

TACAGCTGCA GCAGCTACAG ATGGAGAAGG AGAGGCTGCG ATTGAAACAG

TACAGCTGCA GCAGTTACAG ATGGAGAAGG AGAGACTGCG GTTGAAACAA

CAAGAACTGC TTCGGCAGGC AATGCGGAAT ATCAATCCCA GCACAGCA

CAGGAGTTAT TTCGGCAGGC AATACGGAAT ATCAATCCCA GCACAGCA

CAGGAATTAT TTCGGCAGGC AATACGGAAT ATCAATCCCA GCACAGCA

>Ortholog Group 169, Repeat 1

3 198

R00019984 ATGGAACGCG AACGCTTGGA AAGGGAACGC ATTCGTATTG AACAGGAACG

M00049112 ATGGAACGCG AACGCTTGGA AAGGGAACGC ATTCGTATTG AACAGGAGCG

H00369887 ATGGAACGCG AACGCTTGGA AAGGGAACGC ATTCGTATTG AACAGGAACG

GCGTAGGGAA GCTGAACGGA TTGCTCGAGA GAGAGAGGAA CTCAGAAGGC

GCGCAGGGAA GCTGAAAGGA TTGCTCGGGA GAGAGAGGAG CTCAGAAGGC

TCGTAAGGAA GCTGAACGGA TTGCTCGAGA AAGAGAGGAA CTCAGAAGGC

TTCGCTATGA ACAGGAAAAA AGGAATTCTT TGAAACGCCC ACGTGATGTA

TTCGCTATGA ACAAGAAAAA AGGAATTCTT TGAAACGCCC ACGTGACGTA

TTCGTTATGA ACAAGAAAAA AGGAATTCCT TGAAACGCCC ACGTGATGTA

GACCATAGGC GAGATGATCC TTACTGGAGC GAGAATAAAA AGTTATCT

GACCATAGGC GAGATGACCC TTACTGGAGC GAGAATAAAA AGTTATCT

GATCATAGGC GAGATGATCC TTACTGGAGC GAGAATAAAA AGTTGTCT

>Ortholog Group 16, Repeat 1

3 198

R00060825 ACTCAGAGTG CTACTTACAG TACCACAGCA GTTACTTACT CCGGTACATC

M00118911 ACTCAGAGTG CTACTTACAG TACCACAGCA GTTACTTATT CTGGTACATC

H00265069 ACTCAGAGTG CTACTTACAG TACCACAGCA GTTACATATT CTGGTACGTC

TTATTCAGGT TATGAAGCAG CCGTATATTC AGCTGCGTCT TCCTACTACA

TTATTCAGGT TATGAAGCAG CCGTATATTC AGCTGCGTCT TCCTACTACA

TTATTCAGGT TATGAAGCAG CAGTGTATTC AGCTGCATCT TCCTACTATA

AGCAAGCAGC AGCAGCAGCT GCTGCTGCAG CAGCAACAGC TGCCTGGACA

AGCAAGCAGC AGCAGCAGCT GCTGCTGCAG CAGCAACAGC TGCCTGGACA

AGCAGGCAGC AGCAGCAGCT GCTGCTGCTG CTGCAACAGC TGCCTGGACA

GGGACCACCT TTACTAAAAA AACACCATTC CAAAATAAAC AACTGAAA

GGGACCACCT TTACTAAAAA AACACCATTC CAAAATAAAC AACTGAAA

GGGACCACCT TTACTAAAAA AGCACCATTC CAAAATAAAC AACTGAAA

>Ortholog Group 170, Repeat 1

3 198

H00260600 TCCTTCTTCA TCACGCTGCC CCTCAGCCTC AGCCCCATCG TGATCATCTC

R00005389 TCCTTCTTCA TAACGCTGCC ACTCAGCCTC AGCCCCATCG TGATCATCTC

M00115406 TCCTTCTTCA TAACGCTGCC CCTCAGCCTC AGCCCCATCG TGATCATCTC

CGTGGTCTCC TGTGTGGTGC ACACGTTGGT CCTGGGGGTC ACCGTGGCCG

CGTGGTCTCC TGTGTTGTGC ATACGCTTGT CTTGGGGGTC ACGGTGGCCG

TGTGGTCTCC TGTGTTGTGC ACACGCTTGT GTTGGGGGTC ACTGTGGCCG

AGGAGCTCAA GGGGATGCAG CTGCTGCGGG AGATCCTGGC CAACGTCTTC

ACGAGCTAGA AGGGATGCAG CTGCTGAGGG AGATCCTGGC TAACGTCTTC

ATGAGCTGGA AGGGATGCAG CTGCTGAGGG AGATCCTGGC CAACGTCTTC

CTCTACCTGT GCGCCATCGC TGTGGGCATC ATGTCCTACT ACATGGCT

CTCTACCTGT GCGCCATCAT CGTGGGCATC ATGTCCTACT ACATGGCA

CTCTACCTGT GTGCTATCAT CGTGGGCATC ATGTCCTACT ACATGGCA

>Ortholog Group 171, Repeat 1

3 93

M00005504 ATGAACTCCG TCCGAGCCGC CAACCGGAGA CCCAGGCGAG TGTCGCGGCC

H00384823 ATGAACTCCG TCCGAGCCGC CAACCGGAGA CCCAGGCGAG TGTCGCGGCC

R00021998 ---------- ---------- ---------- ---------- ----------

GCGCCCGGTG CCCCCGCAGC AGCCGCCGCC GCAGCCGCCT CAG

GCGCCCGGTG CCCCCGCAGC AGCCGCCGCC GCAGCCGCCC CAG

---------- ---------- ---------- ---------- ---

>Ortholog Group 171, Repeat 2

3 132

M00005504 CAGCCGCCGC CGCAGCCGCC CCAGCAGCCG CCGCCCCCGC CGCCGCCCCC

H00384823 CAGCCGCCCC AGCAGCAGCC TCCGCCGCCG CCGCCTCCGC CGCCGCCACC

R00021998 ---------- ---------- ---------- ---------- ----------

GCCGCCTCCG CCGCCGCCTC AGGATCGGAA CAACGCCGGC GAGAGG---G

GCCGCCTCCG CCGCTGCCTC AGGAGCGGAA CAACGTCGGC GAGCGGGATG

---------- ---------- ---------- ---------- ---------G

ATGATGTTCC TGCAGATATG GTTGCAGAAG AA

ATGATGTGCC TGCAGATATG GTTGCAGAAG AA

ATGATGTTCC TGCAGATATG GTTGCAGAAG AA

>Ortholog Group 172, Repeat 1

3 198

H00313199 AAGGAAGAAG AACCAGTGAA GAAGATAATG GAAAAGAAAT ACCACAATGT

M00019128 AAGGAAGAGG AGCCAGTGAA GAAGATAATG GAAAAGAAAT ACCACAATGT

R00046491 AAGGAAGAGG AACCAGTGAA GAAGATAATG GAGAAGAAAT ACCACAATGT

TGGTCTTAGT AAATGTGAAA TAAAAGTAGC CATGTCGAAG GAACAATATT

TGGTCTTAGT AAATGTGAAA TAAAAGTAGC CATGTCAAAG GAACAGTATT

TGGTCTTAGT AAATGTGAAA TAAAAGTAGC CATGTCGAAG GAGCAGTATT

GGGGATCTAG AGGAGGATTT GCAGGAAGAG CTCGTGGAAG AGGTGGTGGC

GGGGATCTAG AGGAGGGTTT GCAGGCAGAG CTCGCGGAAG AGGTGGAGGC

GGGGATCTAG AGGAGGGTTT GCAGGAAGAG CTCGCGGAAG AGGCGGTGGC

CCCAGTCAAA ACTGGAACCA GGGATATAGT AACTATTGGA ATCAAGGC

CCCAGTCAAA ACTGGAACCA GGGATATAGT AACTATTGGA ATCAAGGC

CCCAGTCAAA ACTGGAACCA GGGATATAGT AACTATTGGA ACCAAGGC

>Ortholog Group 173, Repeat 1

3 198

M00092157 CCCAAGTGGA TCCGGAGGCC TGTGGGCGCT TCCTTCTCGT TTGGAGGGAA

R00003072 CCCAAGTGGA TCCGGAGACC TGTGGGCGCC TCCTTCTCAT TCGGAGGGAA

H00421633 CCCAAGTGGA TTCGAAGGCC TGTTGGTGCT TCTTTTTCAT TTGGAGGCAA

GTTGGTGACT TTTGAGAGCG TTGCAGTGCC GCTGCAGCAG GGAGCTGAG-

GTTGGTGACT TTCGAGAACG TTACAGGGCA GCCTCAGCAG GGAGCTGAG-

ACTGGTTACG TTTGAGAATG TCAGAATGCC TTCTCATCAG GGAGCTGAGC

--CCTGTGTT CATCAGTCAG GTGGTGACGG AGAAGGACTT TCTCAACCGG

--CCCGTGTT CATCAGTCAG GTGGTGACAG AAAAGGACTT CCTCAGCCGC

ACCATGTGTT CATTAGTCAG GTTGTAACAG AAAAGGAGTT CCTCAGCCGA

TCGGCGCAGC TGCAGCATGC TGTGCAGTCT CAGGGCTTCA TCGGTTAC

TCAGAGCAGC TACAGCACGT CGTGCAGTCT CAGGGCTTTA TCAGTTAC

TCAGACCAAC TTCAGCAGGC TGTGCAGTCA CAAGGATTTA TCAATTAT

>Ortholog Group 174, Repeat 1

3 198

H00378431 AGTGGGCCGA AATGTCAACT AAGAGCCCAG GAACAGCAAA GTTTGATACA

M00119609 ATAGGTCCAA ACTGTCAGCT CAAAGTGGAA GAACAGCAGC AGCTGGCACA

R00035433 ATAGGGCCAA ACTGTCAGCT CAAAGCGGAA GAACGGCAGC AGTTAGTGCA

CACCAACCAG GCTGAAAGTC ATACAGCTGT TGGCAGAGGA GTAGCTGAGG

CAGTAACCAG GCTGAAAGTC ACACCGCTGT TGACCAAGGA AGAGCT---G

CAGTAACCAG GCTGAAAGTC ACACAGCTGT TGACCAAGGA ACAGCC---G

GCTGTGGTGA CCCAGAAGTG ATGCAAAAAA TGACTGATCA GGTGAACTAC

ACTGTGGTGA CCCAGCCATG ATTCAAAAAC TGGCAGAGCA GCTGAGCCAA

ACAGTGGTGA CCCAGCCATG ATTCATAAAA TGGCAGAGCA ACTGAGCCAG

CAGGCAATGA AACTGACTCT TCTGCAGAAG AAGATTGACA ATATTTCT

CAGGAGAGGA AGCTGTCGCT TCTGCAGAAG AAGGTTGACA ATGCTTCT

CAGGAGAGGA AGCTGATGCT TCTGCAGAAG AAGGTTGACA ATGTTTCT

>Ortholog Group 175, Repeat 1

3 135

H00347046 ATGGAGCGGG CCGGCCCCAG CTTCGGGCAG CAGCGACCCC AGCAGCAGAA

R00019637 ATGTTGCCCT TTGGAGACAA A--------- ---------- ----------

M00069011 ATGGAACGAG CGGGCCCCAA C--------- ---------T CCGTGCGGTC

GCAGCAGCAG AGGGATCAGG ACTCGGTCGA AGCATGGCTG GACGATCACT

---------- ---------- ---------- ---------- ----------

GCAGCAGCAG CGGGACCCGG ACTGGGTGGA AGCGTGGCTG GATGATCACC

GGGACTTTAC CTTCTCATAC TTTGTTAGAA AAGCC

---------- ---------- ---------- -----

GGGACTTTAC CTTCTCTTAC TTTATTAGAA AGGCC

>Ortholog Group 176, Repeat 1

3 204

H00406103 GGTTTTGGAT TTGGTACTGG TTTTGGCACA ACAACGGGAA CTAGTACTGG

M00046540 GGTTTTGGCT TTGGTACTGG TTTTGGCACA ACGACGGGAA CTGGCACTGG

R00003070 GGTTTTGGCT TTGGCACTGG TTTTGGCACA TCGACGGGTA CTGGCACTGG

TTTAGGTACT GGTTTGGGAA CTGGACTGGG ATTTGGAGGA TTTAATACA-

TTTAGGCACT GGCTTGGGAA CCGGACTTGG ATTTGGAGGA TTTAATACCC

TTTAGGCACT GGCTTGGGAA CCGGACTTGG ATTCGGAGGA TTTAACACCC

-----ACTAC AACATTAGGT GGTCTCTTCA GTCAGCCTAC ACAAGCTCCT

AGCAGCAGAC TTCTTTAGGT GGTCTCTTCA GTCAGCCGAC CCAGGCTCCT

AGCAGCAGAC TTCTTTAGGC GGTCTCTTCA GTCAGCCTGC ACAGGCCCCT

ACCCAGTCCA ACCAGCTGAT AAATACTGCG AGTGCTCTTT CTGCTCCAAC

GCACAGTCTA CCCAGCTCAT CAACACTGCC AGCGCACTTT CTGCTCCAAC

GCGCAGTCCA ACCAACTCAT CAACACTGCC AGCGCTCTTT CTGCTCCAAC

GCTG

GCTA

GCTA

>Ortholog Group 176, Repeat 2

3 198

H00406103 CGATTTAAGG CAGTAGGTTA TAGTTGCATG CCCAGTAATA AAGATGAAGA

M00046540 CGGTTTAAGG CTGTAGGCTA TAGTTGTATG CCCAATAATA AAGATGAAGA

R00003070 CGATTTAAGG CTGTCGGTTA CAGTTGTATG CCCAATAACA AAGATGAAGA

TGGGCTAGTG GTTTTAGTTT TCAACAAAAA AGAAACAGAG ATTCGAAGCT

TGGACTAGTA GTTTTAATTT TCAACAAAAA AGAAACAGAT ATTAGAAGCT

TGGACTAGTG GTTCTAATTT TCAACAAAAA AGAAACAGAT ATTAGAAGCT

TGGTAGAATC ATTGCATAAA GTTTTGGGAG GAAACCAGAC CCTTACTGTA

TGGTAGAATC ATTGCATAAA GTTTTGGGAG GAAACCAGAC CCTTACGGTC

TGGTAGAATC ATTGCATAAA GTTCTGGGAG GAAACCAGAC CCTCACTGTC

AATGTAGAGG GCACTAAAAC ATTGCCAGAT GATCAGACAG AAGTTGTT

AATGTGGAGG GCATTAAGAC GTTGCCGGAT GATCAGACGG AAGTTGTC

AATGTGGAGG GCATTAAGAC ACTGCCCGAC GATCAGACAG AAGTTGTC

>Ortholog Group 177, Repeat 1

3 132

H00334836 CCTCCTCAGC AGCCGCCACA GCAGCATAGA GTACTCCAGC AACTACAGCA

R00057591 CCCACTCAGC AGCCACCACA GCAGCATAGA GTTCTCCAGC AATTACAGCA

M00037970 CCCCCTCAGC AACCCCCACA GCAGCATAGA GTCCTCCAGC AGTTGCAGCA

GGGAGATTGG AGATTACAGC AACTCCATTT ACAGCATCGT CATCCTCACC

GGGAGATTGG AGATTACAGC AGCTTCATTT G---CATCGT CATCCC----

GGGAGACTGG AGATTACAGC AACTTCATTT G---CATCGT CATCCCCAC-

ACCACCACCA CCACCACCAC CACCTACTTC AA

---------- ---------- ---CTACTTC AA

---------- ---------- ---CTACTTC AA

>Ortholog Group 177, Repeat 2

3 132

H00334836 GATGCTTATA TGCAGCAGTA TCAACATGCA ACAATGCTTC AACAACAATT

R00057591 AACGCTTATT TGCAGCAGTA TCAACATGCA ATGATACTTC AACAACAGTT

M00037970 AATGCTTACT TGCAGCAGTA TCAGCATGCA ATGATACTTC AGCAACAGTT

TTTAATGCAT TCGGTATATC AACCACAACC TTCTGCATCA CAGTATCCTA

TTTAATGCAC TCAGTGTATC AGCCACAACC TCTTGCATCT CAGTATCCGA

TCTAATGCAC TCAGTGTATC AGCCACAACC GCCTGCATCC CAGTATCCTG

CAATGATGCC GCAGTATCAG CAGGCTTTCT TT

CCATGATGCA GCAGTATCAG CAGGCTCTCT TG

CCATGATGCA GCAGTATCAG CAGGCTTTCT TG

>Ortholog Group 178, Repeat 1

3 198

R00005674 CCGCAGCCTC CGCAGCCCTT CCTGCCGCCT GCAGCCTGCT TCTTTGCGAC

M00020243 CCGCAGCCCC CGCAGCCCTT CCTGCCTCCC GCAGCCTGCT TCTTTGCGAC

H00266744 CCGCAGCCCC AGCAGCCCTT CCTGCCGCCC GCAGCCTGTT TCTTTGCCAC

CGCGGCGGCC GCGGCGGCGG CGGCAGCGGC AGCGGCAGCT CAGAGCGCGG

C---GCGGCG GCGGCGGCAG CGGCGGCGGC CGCGGCAGCT CAGAGCGCGG

GGCCGCAGCC GCGGCGGCCG CAGCCGCCGC AGCGGCAGCG CAGAGCGCGG

CGCCGCAGCT GAGCCCGGTG GCGGACGGCC AGCCCTCAGG GGGCGGTCAC

CGCCGCAGCT GAGCCCGGTG GCCGACAGCC AGCCCTCAGG GGGCGGTCAC

CGCCGCAGCT GAGACCGGCG GCCGACGGCC AGCCCTCAGG GGGCGGTCAC

AAGTCAGCGG CCAAGCAGGT CAAGCGCCAG CGCTCGTCCT CTCCCGAA

AAGTCAGCGG CCAAGCAGGT CAAGCGCCAG CGCTCGTCCT CTCCGGAA

AAGTCAGCGC CCAAGCAAGT CAAGCGACAG CGCTCGTCTT CGCCCGAA

>Ortholog Group 179, Repeat 1

3 198

R00017204 GCCAAGAAGT GGCAGAATGC ACCCAGAGTT ACTACTGGAC CCACGCCTTT

M00038716 GCCAAGAAGT GGCAGAATGC ACCCAGAGTT ACTACTGGAC CCACTCCTTT

H00371155 GCCAAGAAGT GGCAGAACAC ACCCAGAGTT ACTACTGGAC CCACTCCTTT

CAGCACCATG CCAAACGCAG CAGCCGTTGC CATGGCTGCA ACACTTACAC

CAGCACCATG CCAAACGCAG CAGCCGTTGC CATGGCTGCA ACACTTACAC

CAGCACCATG CCAAACGCAG CAGCCGTTGC CATGGCTGCA ACACTTACAC

CTGCTACAGG GCCACAGCCG TCTCTGGGAG TTAGTTTTGG AACGCCATTC

CTGCTACAGG GCCACAGCCG TCTCTGGGAG TTAGTTTTGG AACGCCATTC

CTGCTACAGG GCCACAGCCA TCTCTGGGAG TTAGTTTTGG AACGCCATTC

GGCTCAGGTA TTGGCACTGG CTTGCAATCA AGTGGCTTAG GTTCTTCA

GGCTCAGGTA TTGGCACTGG CTTGCAATCA AGTGGCTTAG GTTCTTCA

GGCTCAGGTA TTGGCACTGG CTTGCAATCA AGTGGCTTAG GTTCTTCA

>Ortholog Group 17, Repeat 1

3 201

M00099549 CTGGACCTGG CCGAGGCCCA ACAGCAGATC CAACATTTGG AGGCACAGGT

R00011115 CTGGACCTGG CTGAAGCCCA ACAGCAGATC CAACATTTAG AGGCACAGGT

H00364691 CTGGAGCTGG CAGAGGCGCA GAGGCAGATC CAGCAGCTGG AGGCGCAGGT

GGATGTAGCC CTGGAGGGGA ACCACAATCC GGTCCAGCCA GAG------G

GGTGGAAGTC CTGGAGCGGA ACCACAGTCC TGTCCAGGTG GAG------G

G---GTGGTG CTGGAGCAGA GCCACAGCCC GGCCCAGCTG GAGGTGGATG

CGCTGGAACT GCAACAGGAG GTTGAACGCC TGCGCAGTGC CCAGGTACAG

CACTGGAGCT GCAACAGGAG GTTGAGCGCC TGCGCAGTGC CCAGGTACGG

CGCTGGAGCT GCAGCAGGAG GTGGAGCGGC TGCGCAGCGC CCAGGCGCAG

ACGGAGCGCA CACTGGAGGC ACGGGAGCGG GCCCACCGCC AGAGGGTGTC

ACAGAGCGCA CACTGGAGGC ACGGGAGCGG GCCCACCGCC AGCGCGTGTC

ACTGAGCGCA CCCTGGAGGC TCGGGAGCGG GCCCACCGCC AGAGGGTGCG

A

G

T

>Ortholog Group 180, Repeat 1

3 150

M00016279 ATGGAGGACA GTTTTCTGGA ATCGTTCGGG AGGCTGAGCC TC------CA

H00369473 ATGGAGGACA GTTTCCTTCA ATCTTTTGGG AGGCTGAGCC TCCAGCCCCG

R00058165 ATGGAGGACA GTTTTTTGGA ATCGTTCGGG AGGCTGAGCC TC------CA

GCAGCCGCCT CCTCGGCCGC CGCCCGCTCG GGGGCCACCT CCGCGCCGCC

GCAGCGGCCG CCCCGGCCGC CCCCG---CG GGGGACACCT CCTCGCCGCC

GCAACCGCCT CCTCGGCCGC TGCCCGCGCG GGGGCCACCT CCGCGCCGCC

ACAGCTTTAG GAAACACCTC TACCTCCTGC GCGGCCTCCC GGGCTCGGGA

ACAGCTTTAG GAAACACCTC TACCTCCTGC GAGGCCTCCC GGGCTCCGGG

ACAGCTTTAG AAAACACCTC TACCTCCTGC GCGGCCTCCC GGGCTCGGGA

>Ortholog Group 181, Repeat 1

3 201

M00070592 TCACCTTTCC AAAGAGGGAT CCCCCACTCA CAGACTTTCT CCAGCATCCG

R00047181 TCACCCTTCC AAAGAGGCAT CCCCCACTCA CAGACTTTTA AACCTTTGAG

H00374110 TCCCCTTTCC AAAGAGGAAT TCCCCATTCA CAGACTTTCT CCAGCATTCG

GGATTGCAGG AGGAGCCCCA GTACCCAGTA TTTCCCCTCA ---AATAACT

GTTTTGGAAG GAATCCCCTG AGAGCAAGGG TTTTCCCTCT AGGAACCGCT

GGAGTGTAGG AGGAGCCCCA GTTCCCAGTA TTTTCCTTCA ---AATAATT

TCTATTACCC ACCTCAAGCC CAGACTGCAG ACCAGCAACC AAATAGGACC

GCGAGTATCC CGTGTCATTG GGAACGGGAG AGAAACAAAA CAATGTATCA

ACTATTATTC ACCTCAAGCC CAAACTCCAG ATCAGCAACC AAATAGGACC

AATGGAGATA AGCTACGAAG AAGCATGCCT AATCTGGCCC GGATGCCGAG

TCAGTAGATA AACTACGAAG AAGTATGCCT AATCTGGCCC GGATGCCAAG

AATGGAGATA AGCTCCGAAG AAGTATGCCT AACCTAGCCC GGATGCCAAG

C

C

T

>Ortholog Group 182, Repeat 1

3 198

R00011026 AGGCCTGGCG TGCCAACTCA GGCTCCTATT AATGCACAGA TGCTGGCCCA

H00267974 AGGCCTGGAG TACCAACACA GGCACCTATT AATGCACAGA TGCTGGCCCA

M00006037 AGGCCTGGAG TGCCCACTCA GGCTCCTATT AATGCACAGA TGCTGGCCCA

GAGGCAGAGG GAAATCCTTA ACCAGCATCT CCGGCAGAGA CAGATGCATG

GAGACAGAGG GAAATCCTGA ACCAGCATCT TCGACAGAGA CAAATGCATG

GAGGCAGAGG GAAATCCTCA ACCAACATCT TCGGCAGAGA CAGATG---G

TGCAGCAGCG AACTTTGATG ATGAGAGGAC AAGGGTTGAA TATGACCCCA

TTCAGCAACG AACTTTGATG ATGAGAGGAC AAGGGTTGAA TATGACACCA

TGCAGCAGCG GACTTTGATG ATGAGAGGAC AGGGCTTGAA TGTGACCCCA

AGCATGGTGG CTCCCACTGG TCTACCAGCA GCCATGAGCA ACCCCCGG

AGCATGGTGG CTCCTAGTGG TATGCCAGCA ACTATGAGCA ACCCTCGG

AGCATGGTGG CTCCCGCTGG CCTACCAGCA GCCATGAGCA ATCCCCGG

>Ortholog Group 183, Repeat 1

3 198

R00047900 GCCGGCGCCA AAGTCGCCTC AGGGCCAGTA CCAGCAACAG GACGCTGGGT

M00035345 GCTGGCGCCA AGGTCGCCTC AGGGCCAGTA CCAGCAACCG CGCGCTGGGT

H00399851 GCCGGCGCCA CCGTCGGGCC AGGGCCGGTT CCAGCTCCGG GGCGCTGGGT

CTCCAGCTCC GTCCTCGACC CCGTCCCCAG CGACGGGCAG ----------

CTCCAGCTCC GTCCTCGACC CCGTCCCCAG CGACGGGCGG ----------

CTCCAGCTCC GTCCCCGCGT CCGACCCCAG CGACGGGCAG CGGCGGCGGC

--CCGCAGTC GCAAATGCCC TCCTCAGAGA ATGGGCAGCT GCGGCTCAAC

--CCGCCGTC GCAAATGCCC TCCTCCGAGA ATGGGCAGCT GCGGCTCAAC

CTCAGCAGCC GCAAGTGCTA TCCTCGGAGG GCGGGCAGCT GCGGCACAAC

CCTCTGCACA TCCAGATGCT GTCGAGAGGC CTGCACGAGC AAATCTTC

CCTCTGCTCA TCCAGATGTT GTCGAGAGGC CTGCACGAGC AGATCTTC

CCATTGGACA TCCAGATGCT CTCGAGAGGG CTGCACGAGC AAATCTTC

>Ortholog Group 184, Repeat 1

3 171

R00019408 CTGCCTCCTC TGGGGAATCC CATCAGTGCC AACATCTCTT CCCCCTCAGA

H00268489 ATGCCACCCC TGGGGAATCC TATTGGTGCC AACATTGCTT CCCCTTCAGA

M00044612 ATGCCGCCTC TGGGGAACCC CATCAGTGCC AACATCGCTT CCCCTTCAGA

GCCCAAGGAG ACCAACCGGA AGAAGTTAGC GGATATGATT GCATCCAGGG

GCCCAAAGAG GCCAATCGGA AGAAACTGGC AGATATGATT GCATCCAGGG

GCCCAAAGAG GCCAACCGGA AGAAGTTAGC GGATATGATT GCGTCCAGGG

CCCAGACACT TGCCCAGGCA CAGGCTCAAG TCCAGGCACA CCTGCAGCAG

CACAAACGCT GGCCCAGGCC CAGGCTCAAG TTCAAGCTCA CCTGCAGCAG

CCCAGACACT CGCCCAGGCC CAGGCGCAAG TCCAGGCACA CCTGCAGCAG

GAGCTGCAGC AGCAGGCCGC C

GAGCTGCAGC AACAGGCTGC C

GAGCTGCAGC AACAGGCCGC C

>Ortholog Group 184, Repeat 2

3 156

R00019408 ATCCAGTCTC AGCTGTTCAA CCCCACGCTC CTTCCTCACT TTCCCATGAC

H00268489 ATCCAGTCTC AGCTGTTTAA CCCCACCCTC CTTCCTCACT TCCCCATGAC

M00044612 ATCCAGTCTC AGCTGTTCAA CCCCACGCTC CTCCCACACT TCCCCATGAC

CACCGAGACC TTGCTCCAAC TGCATCTACT CTTCCCCTTT TACATCCCCA

AACTGAGACC CTGCTGCAAC TACACCTCCT CTTCCCTTTC TACATCCCCA

CACAGAGACC TTGCTCCAGC TGCACCTCCT CTTCCCCTTT TACATCCCCA

GTGCAGAGTT CCAGCTCAAC CCTGAGGTGA GCTTGCCTGT GACCAGCGGG

GTGCTGAGTT CCAGCTTAAC CCCGAGGTGA GCTTGCCAGT GACCAGTGGG

GCGCTGAGTT CCAGCTCAAC CCTGAGGTGA GCCTGCCTGT GACCAGCGGG

GCACTG

GCACTG

GCACTG

>Ortholog Group 184, Repeat 3

3 186

R00019408 CTGACGGGGT CAGGCCCAGG CCTGCTGGAA GACCTGAAGG CTCAGATCCA

H00268489 CTGACTGGGA CAGGCCCAGG CCTGCTGGAA GATCTGAAGG CTCAGGTTCA

M00044612 CTCACGGGGT CAGGCCCAGG CCTGCTGGAA GATCTGAAGG CTCAGGTCCA

GATCCCACAG CAGAGCCACC CACAGATACT GCAGCAGAGT CAGCTCTCTC

GGTCCCACAG CAGAGCCATC AGCAGATCTT G---CCGAAC CAACTCTCTA

GATCCCACAG CAGAGCCACC AGCAGATCCT GCAGCAGAGT CAGCTCTCTC

TTTCCCAGAG TCACTCAGCT CTCCTTCAGC CAAGCCAGCA CCCGGAAAAG

TAGCCCAGAG TCACTCTGCC CTCCTTCAGC CAAGCCAGCA CCCCGAAAAG

TTTCCCAGAG TCATTCAGCC CTCCTGCAGC CAAGCCAACA CCCGGAAAAG

AAAAACAAAG TGGTCATCAA AGAAAAGGAT AAGGAA

AAGAACAAAT TGGTCATCAA AGAAAAGGAA AAAGAA

AAAAACAAAG TGGTCATCAA GGAAAAGGAT AAGGAA

>Ortholog Group 184, Repeat 4

3 132

R00019408 CTCCCCACAT CTGGATTACC AAATAAACCG TCCTCAGCCT CACTGAGCTC

H00268489 CTCCCCACTT CTGGATTACC AAATAAACCG TCCTCAGCGT CGCTGAGCTC

M00044612 CTCCCCACAT CTGGATTACC AAATAAACCG TCCTCAGCCT CGCTGAGTTC

CCCGACCCCA GCACAAGCCA CCATGGCAAT GGCCCCTCAG CAACCCCCAC

CCCAACCCCA GCACAAGCCA CGATGGCGAT GGGCCCTCAG CAACCCCCCC

CCCGACCCCA GCACAAGCCA CCATGGCAAT GGCCCCTCAG CCACCCCCAC

CACAGGTG-- -CAGCCGCCG CCGCCGCCAG CA

CACAGGTGCA GCAGCCTCCC CCGCCGCCAG CA

CACCGGTGCA GCAGCCTCCG CCGCCGCCAG CA

>Ortholog Group 184, Repeat 5

3 132

R00019408 GCCCAGCAGA TACCCACGCC ACAGCTTCCC CTACGGAAGG ACAAAGATGG

H00268489 GCCCAGCCGC CACCCACACC ACAGCTCCCA CTGCGCAAGG ACAAAGACAG

M00044612 GCCCAACAGA TCCCTGCACC ACAGCTGACC CCGCGTAAGG ACAAAGATGG

TGAGAAAGGA AAGGAGAAGG AGAAGGCACA CAAAGGGAAA GGGGAACCCC

TGAGAAAGTA AAGGAGAAGG AAAAGGCACA CAAAGGGAAA GGGGAACCCC

TGAGAAAGGA AAGGAGAAGG AAAAGGCACA CAAAGGGAAG GGGGAGCCCC

CACCTGTCCC CAAGAAGGAG AAAGGAGAGG CC

TGCCTGTCCC CAAGAAGGAG AAAGGAGAGG CC

TCCCCGTCCC CAAGAAGGAG AAAGGAGAGG CC

>Ortholog Group 184, Repeat 6

3 102

R00019408 CCCTACAGCC CTGCCCTGTC GCAGGCCCTG ATGGGGTTGT CTCCGGGCTC

H00268489 CCCTACAGCC CTGCACTGTC GCAGGCCCTG ATGGGGCTGT CCCCAGGCTC

M00044612 CCCTACAGCC CTGCCCTATC GCAGGCCCTG ATGGGCTTGT CTCCAGGCTC

ACTACTGCAG CAGTACCAGC AATACCAGCA GAGTCTGCAG GAGGCCATTC

CCTACTGCAG CAGTACCAGC AATACCAGCA GAGTCTGCAG GAGGCAATTC

CCTACTGCAG CAGTACCAGC AATACCAGCA GAGTCTGCAG GAGGCGATTC

AG

GG

AG

>Ortholog Group 184, Repeat 7

3 6

R00019408 CAACAG

H00268489 CTAAAA

M00044612 CAACAG

>Ortholog Group 184, Repeat 8

3 102

R00019408 CAGCCCAAAG CAAGCCAAAC CCCAGTCCCC CAGGGGCCTG CTTCCCCAGA

H00268489 GTGCCCAAAG CAAGCCAAAC CCCAGTCCCC CCCGGGGCTC CTTCCCCAGA

M00044612 CAGCCCAAAG CAAGCCAAAC CCCAGTCCCC CAGGGGCCTG CTTCCCCAGA

CAAAGACCCT GCCAAAGAAT CCCCCAAACC AGAAGAGCAG AAAAACGTCC

CAAAGACCCT GCCAAAGAAT CCCCCAAACC AGAAGAACAG AAAAACACCC

CAAAGACCCT GCCAAAGAAT CCCCCAAACC AGAAGAGCAG AAAAACGTCC

CC

CC

CC

>Ortholog Group 185, Repeat 1

3 183

H00268711 AGCCCGGAGG TCCACCAGGA CCTGAACGCC CTCAAAAGCA AGTTCCAGGA

R00004455 AGCCCGGATC TCCACCAGGA CCTGAACGCC CTCAAAACAA AGTTCCAGGA

M00080641 AGCCCGGATC TCCACCAGGA CCTGAACGCC CTCAAAACCA AGTTCCAGGA

GATGCGCAAG CTCATCAGCA CCATGCCCGG CATCCACCTG AGCCCCGAAC

GATGCGGAAG CTCATAGGCA CCATGCCTGG CATCCATGTG AGCCCTGAGC

GCTGCGGAAG CTCATCGGCA CCATGCCCGG CATCCACGTG AGCCCCGAGC

TGCAGAGCCT CCGGGAGCAA GTCAGGACCA AGAATGAGCT TCTGCAAAAG

TACACAGCCT CCGAGAGCAA GTCAGGACCA AGAATGAGCT TCTGCAGAAG

TCCACAGCCT CCGAGAGCAA GTGAGGACCA AGAACGAGCT GCTGCAGAAG

TACAAGAGCC TCTGCATGTT CGAAATCCCC AAG

TACAAGAGCC TCTGCATGTT TGAGATCCCC AAG

TACAAGAGCC TCTGCATGTT TGAGATCCCC AAG

>Ortholog Group 186, Repeat 1

3 201

R00054489 GTCCAGTATA CCTTTCCCAG TACCCGGCAC CAGCAGGAAT TTGCAGTTCC

H00379198 GTCCAGTATA CATTTCCCAA CACCCGCCAC CAGCAGGAGT TCGCAGTCCC

M00018645 GTCCAGTATA CCTTTCCCAG TACCCGACAC CAGCAGGAAT TTGCAGTTCC

TGACTATCGC TCTTCTCACA TCGAAGTTAG TCAGGCATCG CAGCTCTTAC

TGATTATCGT TCCTCTCATC TTGAAGTGAG TCAGGCATCA CAGCTTTTG-

TGACTACCGC TCTTCTCATA TTGAAGTTAG CCAGGCATCA CAGCTTTTAC

AGCTTCGAAG GCGACCTTCC TTGCTTTCAG AATTTCACCC AGGTTCTGAC

--CTTCGAAG GCGACCTTCC TTGCTTTCAG AATTTCACCC AGGTTCTGAC

AGCTTCGAAG ACGACCTTCC TTGCTTTCAG AATTTCACCC GGGTTCTGAC

AGGCCTCAAG AGAGAAGAAC TGGATATGAA CAGTTTCACT CAGGACCCTC

AGGCCTCAAG AAAGGAGAAC TAGTTATGAA CCGTTTCATC CAGGCCCATC

AGGCCTCAAG AAAGGAGAAC TGGATATGAA CAGTTTCACT CAGGACCCTC

A

C

A

>Ortholog Group 187, Repeat 1

3 111

H00401727 CCAAAGGTGA AGAAGCTTAA ATATCACCAG TACATTCCCC CAGACCAGAA

M00104335 CCAAAGGTGA AGAAGCTCAA ATACCATCAG TACATCCCCC CAGACCAGAA

R00054372 CCAAAGGTGA AGAAGCTCAA ATACCACCAG TACATCCCCC CGGACCAGAA

GGCAGAGAAG TCCCCTCCAC CTATGGACTC AGCCTACGCT CGGCTGCTCC

GGCAGAGAAG TCTCCCCCAC CCATGGACTC TGCCTATGCC CGGCTGCTCC

GGCAGAGAAA TCCCCTCCCC CCATGGACTC TGCCTATGCT CGGCTGCTTC

TGTTCCTGCA G

TATTCCTGCA G

TATTCCTGCA G

>Ortholog Group 187, Repeat 2

3 150

H00401727 CAAATCCTCA GC-------- ---------- ---------- ----------

M00104335 CAGATCCTCA GCCAGCAGCA GCAACAGCAG CAGCAACAGC AGCAGCAGCA

R00054372 CAGATCCTCA GC---CAGCA GCAACAGCAG CAGCAACAGC AGCAACAGCA

-CACCGATTC AGCTACCTAG GGATGCACCA AGCTCAGCTT AAGGAACCAA

ACAGCGGTTC AGCTACCCTG GGATGCACCA AACACACCTC AAAGAACCAA

GCAACGGTTC AGCTACCCTG GGATGCACCA AGCACACCTC AAAGAGCCAA

ATGAACAGAT GGTCAGAAAT CCAAACTCTT CTTCAACGCC ACTGAGCAAT

ATGAACAGAT GGCCAGAAAT CCGAATCCTT CTTCAACACC ACTGAGCAAT

ATGAACAGAT GACCAGAAAT CCGAATTCTT CCTCAACACC ACTGAACAAT

>Ortholog Group 188, Repeat 1

3 198

R00038436 GAGGAGTCCT CACCACATTA CTGCGAAGGA GGAAACTCGC AGTACGGCCA

H00414516 GAGGATTCCT CACAACATTA CTACGAAGGA GGAAATTCAC AGTATGGCCA

M00046320 GAGGATTCCT CACAACATTA CTACGAAGGA GGAAACTCCC AGTATGGCCA

GCAGCAGGAC GCTTACCAAG GACCACCTCC ACAGCAAGGA TACCCACCCT

ACAGCAAGAT GCATACCAGG GACCACCTCC ACAACAGGGA TATCCACCCT

ACAGCAAGAC GCTTACCAGG GACCACCTCC ACAGCAAGGA TACCCACCCT

ACCCGGGGCA GCAGGGCTAC CCAGGACAGC AGCAGGGCTA CGGTCCTACC

ACCCAGGGCA GCAAGGTTAC CCAGGACAGC AGCAGGGCTA CGGTCCTTCA

ACCCGGGACA GCAGGGATAC CCAGGGCAGC AGCAGAGCTA TGGTCCTTCG

CAGGGCGGTC CAGGGCCTCA GTATCCTAAT TATCCTCAGG GTCAAGGT

CAGGGTGGTC CAGGTCCTCA GTATCCTAAC TACCCACAGG GACAAGGT

CAGGGCGGTC CAGGTCCTCA GTATCCTAAT TATCCTCAGG GTCAAGGT

>Ortholog Group 189, Repeat 1

3 198

R00020661 CTGAAGGCTT TTGCATTAAA AAATGCAGAT TTTTCTTCCT ACTTACTCTC

H00269197 TTAAAAGCAT TCGCGCTAAA AAGTGCAGAT TTCTCTTCCT ATTTGCTTTC

M00112793 CTGAAGGCAT TTGCATTAAA AAATGCAGAT TTTTCTTCCT ACTTGCTCTC

TGAGCCCCAA AAGCCTTTTA CCCAATTAGC TGCTCAGAAA CTTCCGGTAC

TGAGCCACAA AAGCCTTTTA CCCAATTAGC TGCTCAGAAA ATGCAGGTGC

TGAGCCCCAA AAGCCTTTTA CCCAATTAGC TGCTCAAAAA CTACCAGTAC

TCTGTGGAAG TTACCCGACC ATACACTTTG GGAGCACAAA TTTCAAAAGG

TCTGTGGAAA TTATCCAACA ATACACTTTG GTAGCACGAG TTTCAAAAGG

TCTGTGGAAG TTACCCGACG ATACACTTTG GGAGCACAAA TTTCAAAAGG

GCAGCGTCTG CCATTGAAAA GTCCATTGGG ATTCTGGGAA GTGGCTCC

GCAGCATCTG CAATTGAAAA GTCCATTGGG ATTTTGGGAA GTGGCTCC

GCAGCATCTG CCATTGAAAA GTCCATTGGG ATTCTGGGAA GTGGCTCC

>Ortholog Group 18, Repeat 1

3 198

H00313059 GTATATCCAC CCTCAAGTGT GGCACAGGGG CAGAGCCAGG GTCAGCCATC

M00063001 GTGTATCCAC CCTCAAGTAT GGCACAGGGG CAGAACCAGG GCCAGCCA--

R00041493 GTGTATCCAC CCTCAAGTAT GGCACAGGGG CAGAACCAGG GCCAGCCA--

CTCAAGTAGC TTAACAGGGG TTTCATCTTC CCAACCCATA CAACATCCTG

-TCAAGTAGT TTGGCTGGGG TTCTATCTTC ACAACCTATC CAACATCCTG

-TCAAGTAGT TTAGCAGGGG TTCTATCTTC ACAACCCGTC CAACATCCTG

GAATACAGCA GACAGCCCCT CCTCAACAGA CAGTGCAGTA TTCACTTTCA

GAATACAGCC AACTGTTCCT TCTCAACAGG CAGTACAGTA TTCACTTCCA

GAATACAGCC GACTGTACCT CCTCAACAGG CAGTACAGTA TTCACTTCCA

CAGACATCAA CCTCCAGTGA GGCCACTACT GCACAGCCAG TGAGTCAG

CAAGCAGCAT CTTCCAGTGA AGGTACTACT GCACAGCCAG TGAGTCAG

CAGGCAGCGT CTTCCAGTGA AGGC---ACT GTCCAGCCAG TGAGTCAG

>Ortholog Group 190, Repeat 1

3 99

M00072091 GAGCCAAGCT CCATCATGAA TGTGCCCGGT GAGTCCACAC TGCGCAGAGA

H00347427 GAGCCAAGCT CCATCATGAA CGTGCCTGGA GAGTCGACTC TACGCCGGGA

R00048832 GAGCCAAGTT CCATCATGAA TGTGCCTGGG GAGTCCACAC TCCGCCGAGA

ATTCCTCAGA CTCCAGCAGG AGAATAAGAG CAACTCTGAG GCTTTAAAG

GTTTCTCCGG CTCCAGCAGG AAAATAAGAG CAACTCAGAG GCTTTAAAA

ATTCCTCAGA CTCCAACAGG AGAATAAGAG CAACTCAGAG GCTTTAAAG

>Ortholog Group 190, Repeat 2

3 99

M00072091 CGGGACCCGG AGGCACACAT CAAACACCTG CTGCACCAGC GGCAGCGTCG

H00347427 CGAGACCCCG AGGCACACAT CAAACACCTG CTGCACCAGC GGCAGCGGCG

R00048832 CGGGACCCTG AGGCACACAT CAAACACCTG CTGCACCAGC GGCAGCGTCG

CATAGAGGAG CAGAAGGAGG AGCGGCGACG TGTGGAGGAG CAACAGCGG

CATAGAGGAG CAGAAGGAGG AGCGGCGCCG CGTGGAGGAG CAACAGCGG

CATAGAGGAG CAGAAGGAGG AGCGGCGACG TGTAGAGGAG CAACAGCGG

>Ortholog Group 190, Repeat 3

3 102

M00072091 CGGGAACAGG AATACAAGCG GAAGCAGCTG GAGGAGCAGC GGCAGTCAGA

H00347427 CGCGAGCAGG AATACAAGCG GAAGCAGCTG GAGGAGCAGC GGCAGTCAGA

R00048832 CGGGAACAGG AATACAAGCG GAAGCAGCTG GAGGAGCAGC GGCAGTCGGA

GCGGCTGCAG AGACAGCTGC AGCAGGAGCA CGCCTACCTC AAGTCCCTGC

ACGTCTCCAG AGGCAGCTGC AGCAGGAGCA TGCCTACCTC AAGTCCCTGC

GCGGCTCCAG AGACAGCTGC AGCAGGAGCA TGCCTACCTC AAGTCCCTGC

TC

TT

AG

>Ortholog Group 190, Repeat 4

3 108

M00072091 AAGCAGCAGA TCCTGCCTGG AGACAGGAAG CCCCTGTATC ATTACGGTCG

H00347427 AAA------C TCCTGCCTGG GGACAGGAAG CCCCTGTACC ATTATGGTCG

R00048832 CAGCAGCAGA TCCTACCTGG AGACAGGAAA CCCCTGTATC ATTATGGTCG

GGGCATTAAT CCTGCTGACA AGCCAGCATG GGCCCGCGAG GTGGAAGAGA

GGGCATGAAT CCCGCTGACA AACCAGCCTG GGCCCGAGAG GTAGAAGAGA

GGGCATTAAT CCTGCTGACA AGCCAGCATG GGCCCGAGAG GTGGAAGAGA

GAGCACGG

GAACAAGG

GAGCTCGG

>Ortholog Group 191, Repeat 1

3 198

R00059641 CGGCGCGGCC GCCGCCAGCT CCCCCAGACC CCCTGCACCC CGCGGCCGCT

H00353362 CGGCGGGGCC GCCGCCAGCT CCCCCAGACC CCCTCCACCC CCCGGCCACA

M00112436 CGGCGCGGCC GCCGCCAGCT CCCCCAGACC CCCTGCACCC CGCGGCCGCT

TGTGTCCTAC TCGCCCGCTC CGCGCAGGCC TGCGGCG--- ----------

CGTGTCCTAT TCCCCTGTGA TCCGTAAGGC CGGCGGCTCG GGGCCCCCGG

TGTGTCCTAC TCGCCCGCTC CGCGCAGGCC TGCGGCG--- ----------

---------- ---------- ---------- --------CG CAAGATGGCG

CGGTGGCCAG GCCGGGCCGG GCGGCCACCA GCGGCCCTCG GAGGTACCCA

---------- ---------- ---------- --------CG CAGGATGGCG

GGTCCCCCAG CGCCCCCT-- ---------- ---------- --------

GGCCCCACGG CCGAGCCTCT GGCCGGAGAT CGGCCGCCCA CGGGGGGC

GGTCCCGCAG CGCCCCCT-- ---------- ---------- --------

>Ortholog Group 192, Repeat 1

3 213

H00269724 TGCTCCGGGT CAGCTACAAG GGGAGCATCT CGTACCGCAA CGCGGCGCGC

M00092853 GCGCCCGCCG CGCCCCCGGC CGCGGCGCCC CCAGCCGGCC CGCGCCGTGC

R00007303 ---------- ---------- ---------- ---------- ----------

GTCCAGCCGC CCCGGCGCGG AGCCACCGCC GGCC------ ---------C

A--------- ---------- --CCGCCGCC CGCCGTCGCC GCCCGGGAGC

---------- ---------- ---------- ---------- ----------

CGCCGGCGCC GCCACCGCCG CCGCCGCAGC CACAGCCGCC GCCGGAGGGG

CGCCGGCGCC GCCACCGCCA CCGCCGCAGC CACAGCCGCC GCCGGAGGGG

---------- ---------- ---------- ---------- ----------

GGCGCGGTGC GGGCCGGCGG CGCGGCGCGG CCCGTGAGCC TGCGGGAAGT

GGCGCGGCGC GGGCGGGCGG CCCGGCGCGG CCCGTGAGCC TGCGGGAAGT

---------C GGGCGGGCGG CCCGGCGCGG CCCGTGAGCC TGCGGGAAGT

CGTGCGCTAC CTC

CGTGCGCTAC CTC

CGTGCGCTAC CTC

>Ortholog Group 193, Repeat 1

3 108

M00113791 CCACTCACCT GGCGAGCAGT GCCCCAGACA GATGTGCTGC AGCCGCCACA

R00041592 CCACTCACCT GGCGAGCTGT GCCCCAGACA GATGTACTGC AGCCGCCACA

H00269844 CCGCTCACGT GGCGGGCAGT GCCCCAGACT GACGTCTTGC CACCCTCGCA

GGCCCCTGCA GCCCCGCAGC AGGCGGTGCA ACCCCAGGTA CAGAATGAGA

GGCCCCTGCA GCCCCGCAGC AGGCGGTGCA ACCCCAGGTC CAGAATGAGA

GCCGCAGGCA CCCCCACAGC AGGCGGCCCA GCCCCAGGTG CAGGCGGAGA

TGTACAGC

TGTATAGC

TGTACAGC

>Ortholog Group 194, Repeat 1

3 198

R00018167 GAGAGGATGG CGGCGAGTCT CTCGGAACTG AGTGGCAGTG TGGCCCAGAC

H00349016 GAGAGGATGG AGGCCGGTCT CTCTGAGCTG AGTGGCAGCG TGGCCCAGAC

M00099506 GAGAGGATGG CAGCGAGTCT CTCGGAACTG AGTGGCACCG TGGCCCAGAC

AGTGACTCAG GTACAGACAA CACTGGCCTC TGTCCAGGAG CTACTGAGAA

AGTGACTCAG TTACAGACGA CCCTCGCCTC CGTCCAGGAG CTGCTGATTA

AGTGACTCAG GTGCAGACAA CACTGGCCTC TGTCCAAGAG CTACTGAGAA

AGGTCCAGGA GCTAGCCCAT GAGCTGGCCG CTGCCAAGGC TACCACATCA

AGATCCAGGA GCTTGCCCAC GAGCTGGCCG CTGCCAAGGC CACCACATCC

AGGTCCAGGA GCTAGCTCAC GAGCTGGCCA CTGCCAAGGC CACCACATCA

ACCAACTGGA TCCTGGAGTC CCAGAATATC AATGAGCTCA AGTCAGAA

ACCAACTGGA TCCTGGAGTC CCAGAATATC AACGAACTCA AGTCCGAA

ACCAACTGGA TCCTGGAGTC CCAGAATATT AATGAGCTCA AGTCGGAA

>Ortholog Group 195, Repeat 1

3 198

H00359474 CTGGTAGCAG ACGCGACCCA GCATCATCAC CACCTCCACC ACAGCCAGCA

M00084005 CTTGTGACAG ACGGACCCCA GCATCACCAT CACCTCCACC ACAGCCAACA

R00002887 CTTGTGACAG ACGGACCCCA GCATCACCAT CACCTCCACC ACGGCCAACA

GCCGCCGCCG CCGGCCGCGG CCCCGACGCA AAGTTTGCAG CCTTTGCCCC

GCCGCCGCCA CCATCCGCGG TCCCCGCGCA AAGTTTGCAG CCTTCGCCCC

GCCACCGCCA CCGTCCGCGC CCCCCGCGCA AAGTTTGCAG CCTTCTCCCC

CGCTGCCGCC ACAGCAGCCG CCGCCGCCGC CCCCCCAGCA GCTGGGCTCG

CGCCGCAGCC GCAGTCA--- ---------G CAGCCCAGCA GCTGGGCTCG

CGCCGCAGCC GCAGTCT--- ---------G CAGCCCAGCA ACTGGGCTCG

GCCGCCTCGG CCCCCAGGAC TTCCACGTCT TCTTTTTTAA TTAAGGAC

GCCGCCGCGG CCCCCAGGAC TTCCACCTCT TCCTTTTTAA TTAAGGAC

GCCGCCGCGG CCCCCAGGAC TTCCACCTCT TCCTTTTTAA TTAAGGAC

>Ortholog Group 196, Repeat 1

3 198

H00376461 GCGCTGGGCA TGTCCCGAGA TGCTGTCAAG TTCGGCCGCA TGTCCAAGAA

M00029795 GCTCTGGGCA TGTCCCGAGA TGCTGTCAAG TTTGGCCGAA TGTCCAAGAA

R00028292 GCTCTGGGCA TGTCCAGAGA TGCTGTCAAG TTCGGCCGAA TGTCCAAGAA

GCAGAGGGAC AGCCTGCATG CAGAAGTGCA GAAACAGCTG CAGCAGCGGG

GCAGAGGGAC AGTCTACATG CAGAAGTGCA GAAACAACTG CAA------G

GCAGAGGGAC AGTCTGCATG CAGAGGTGCA GAAACAACTG CAA------G

AACCAGTGGT CAAGACCCCT CCAGCAGGGG CCCAAGGAGC AGATACCCTC

AACAAGTGGC CAAGACTCCT CCAGCTGGGA GCCGCGGAGC AGACACACTT

AACAAGTAGC CAAGACTCCT CCAGCTGGGA GCCATGGAGC AGACACACTC

ACCTACACCT TGGGGCTCCC AGACGGGCAG CTGCCCCTGG GCTCCTCG

ACATACACTT TAGGGCTCTC AGATGGGCAG CTACCACTGG GCGCCTCA

ACATACACTT TAGGGGTCCC AGATGGGCAG CTACCACTGG GCGCCTCA

>Ortholog Group 197, Repeat 1

3 132

H00354669 AGTCATTCCC TGGGCAAGGG GCCTGGAGCA GAAGGTGGGA GTGGCTCCCC

M00102805 AGTCATTCCC TAGGCAAAAG CCATGGAGCA GAAGGTGGGG GTGGCTCTCC

R00053218 AGTCATTCCC TAGGCAAAAG CCATGGAGCA GAAGGTGGGA GTGGCTCTCC

AGAAACGCAG CTGCAGGTAG ACCAGGACTA CCTGATTGCT CTGTCCCTG-

AGAGAAGCAG CTGCAAGTGG ACCAGGACTA CCTTATTGCC TTATCCCTGC

AGAGAAGCAA CTGCAGGTAG ACCAGGACTA CCTTATTGCC TTGTCCCTGC

--CCACGAGG CCCGCTGGGG CTTACCGACT TG

AGCCACAAGG CACGTTGGGC CTCAGTGACC TG

AGCCACAGGG CATGCTGGGT CTCAGTGACC TG

>Ortholog Group 197, Repeat 2

3 129

H00354669 CTGGCCCAGC AGCTTCAGCA AGAGGAGTAT GCAGCGCAGC CAGTGCGGAT

M00102805 CTGGCCCAAC AACTTCAACA AGAAGAGTAT GCAGTTCAAC CTGTG-----

R00053218 CTGGCCCAGC AACTTCAACA AGAAGAGTAC GCAGTTCAAC CTGTG-----

GCGGACGCGG GTCCTGTCAC TGCAGGGGAG AGGAGCCACA TCTGGACGCC

-CGAACAAGG GCTCCTTCGC CCCAGGGGAG AGGAGCCACA TCTGGACGTC

-CGGACCAGG GCCCCTTCAT CCCCGGGTAG AGGAGCCACA TCTGGACGCC

CAGCCGGGGA GCGTCGGCAG AGGCCGAAG

CAGCTGGAGA GCGGCGGCAG AGGTCAAAG

CAGCTGGGGA GCGGAGGCAG AGGTCGAAG

>Ortholog Group 198, Repeat 1

3 234

R00060764 GAACCACGGC CTACATTGTC TAACACAATC CAGAGGTCAC AGCTAGGTCC

M00099810 GAACCACGGC CTACACTGTC CAACACCATC CCAAGGTCAC AGCTAGGTCC

H00351407 GAACCACGGC CTACACTCTC CAACACAATC CAGAGGCCAC AACTAGGTCC

GACAACTAAT TTATCCCTAG AGATGGGTAC AGGGCAGCTG GCATCCAGGC

GACAGCCAAT TTATCCCTAG AGATGGGTAC AGGGCAGCTG CCATCCAGG-

CACAGCTAAT TTACCCCTGG AGATGGGCTC AGGACAGCTG GCACCCAGG-

AGCAGCAGCA GCAGCAGCAG CAGCAGCAGC AGACAGAATT GGATATGGTA

---------- ---------- ---------C AGACAGAACT GGATATGGTA

---------- ---------- ---------- --ACAGAATT GGACATGGTA

CCAGGAAGAG ATGGGCTGGC CAGCTATAGT CATTCCCAGG TTTCTGTCCA

CCAGGAAGAG ATGGGCTGGC CAGCTATAAT CATTCCCAGG TTTCTGTCCA

CCAGGAAGAG ATGGACTGGC CAGCTACAAT CATTCCCAGG TG---GTTCA

GCCCGTGGCA ACTGCAGGAT CAGAACACAG CAAG

GCCTGTGGCA AGTGCAGGAT CAGAACACAG CAAG

GCCTGTGACA ACCACAGGAC CAGAACACAG CAAG

>Ortholog Group 199, Repeat 1

3 105

R00005715 TCAGTGAGAG ATGCCAATGG CATCGTCTGG AAGACGGGTC CCAGAACTAC

H00355961 TCTGTGAAAG ATGCCAATGG TATAGTATGG AAGACTGGTC CCAGAACTAC

M00036277 TCGGTGAGAG ATGCCAATGG CATCGTCTGG AAGACTGGCC CCAGAACTAC

CATGTTTGTC AAATCCCTGG AAGACCCCTA TTCCCAGCAG ATTCGCCTAG

CATATTTGTA AAATCCCTGG AAGACCCTTA TTCCCAGCAA ATTCGCTTAG

TATGTTTGTC AAATCCCTGG AAGACCCCTA CTCCCAGCAG ATTCGCCTAG

CCCAG

CCCAG

CCCAG

>Ortholog Group 199, Repeat 2

3 24

R00005715 CCTTTGCGCG GTGCCTACAC CCGG

H00355961 CCATTACGCA ATGCCTACAC ACGG

M00036277 CCTTTGCGCA GTGCCTACAC CCGG

>Ortholog Group 19, Repeat 1

3 198

R00026721 GCCGGTGTGT CTGGTCCGGG TTCTGCTGGT GGTCCTGGTC CCCAGCAACA

H00314343 GCTGGTGTAT CGGGTCCTAG TTCGGCTGGC GGCCCGGGTC CCCAGCAGCA

M00003536 TCTGGTGTGT CAGGCCCGGG TTCTGCCGGG GGTCCGGGTC CCCAGCAACA

GCCGCAA--- CCAACCCAAC TGGTGGGTGC TGCCCAGAGC GGGCTCCTGG

GCCGCAACCG CCAGCACAAC TGGTGGGCCC TGCCCAGAGC GGCCTCCTGG

GCCACAA--- CCGACCCAGT TGGTGGGTTC TGCCCAGAGC GGGCTCCTGG

ACTTCGATCC TGTGCAGCGC TACAAGATGC TCATCCCGCA ATTGAAGGAG

ACTTCGATCC TGTGCAGCGT TATAAGATGC TCATCCCGCA GCTGAAGGAG

ACTTCGATCC TGTGCAGCGC TACAAGATGC TCATCCCGCA ACTGAAGGAG

AGTCTCCAGA CTTTGATGAA AGTTGCTGCC CAGAACCTGA TTCAGAAC

AGTCTACAGA CCTTGATGAA GGTTGCGGCC CAAAACTTGA TTCAGAAC

AGTCTCCAGA CTTTGATGAA GGTTGCAGCC CAGAATCTGA TTCAGAAC

>Ortholog Group 1, Repeat 1

3 165

H00345702 ATGGAGCAGT ATACAGCAAA CAGCAATAGT TCGACAGAGC AGATTGTTGT

M00043909 ATGGAGCAGT ATACGACAAA CAGCAATAGT TCCACAGAGC AGATCGTGGT

R00017157 ATGGAGCAGT ATACAGCAAA CAGCAATAGT TCCACAGAGC AGATCGTGGT

CCAGGCAGGA CAGATTGGTG GTGTCACTGC TGTGCAGTTG CAGACTGAGG

GCAGGCCGGC CAGATTGGTG GTGTCACTGC TGTCCAGCTG CAGACTGAGG

GCAGGCTGGC CAGATTGGTG GTGTCACTGC TGTCCAGCTG CAGACTGAGG

CCCAGGTGGC ATCCGCCTCA GGCCAGCAAG TCCAGACCCT CCAGGTAGTC

CCCAGGTGGC ATCCGCCTCA GGCCAGCAAG TCCAGACCCT CCAGGTAGTC

CCCAGGTGGC ATCCGCCTCA GGCCAGCAAG TCCAGACCCT CCAGGTAGTT

CAAGGGCAGC CATTA

CAGGGGCAGC CGTTA

CAGGGGCAGC CATTA

>Ortholog Group 200, Repeat 1

3 132

R00045726 ATGAACCCGA GCAATGGAGA TGGATTTCTA GAGCCTCAGT CCCCCCAGAG

M00035477 ATGAACCCGA GCAATGGAGA TGGATTTCTA GAGCCTCAGT CCCCCCAGAG

H00399288 ATGAACCCGA GCAATGGAGA TGGATTTCTA GAGCCTCAGT CCCCCCAGAG

ACTCTTGGCC GTGATCCTGT GGTTTCAACT GGCGCTGTGC TTTGGCCCTG

ACTCTTGGCC GTGATCCTGT GGTTTCAACT GGCGCTGTGC TTTGGCCCTG

ACTCTTGGCC GTGATCCTGT GGTTTCAGCT GGCGCTGTGC TTCGGCCCTG

CGCAGCTCAC CGGTGGGTTC GATGACCTCA AC

CACAGCTCAC GGGTGGGTTC GATGACCTCA AC

CACAGCTCAC GGGCGGGTTC GATGACCTTC AA

>Ortholog Group 201, Repeat 1

3 135

M00112508 GGTGTCAAAG TTCCCGGCGA GCATCGGAGG AAGGAGAATG GGGTTAACAG

R00004330 GGTGTAAAAG TTCCTGGTGA GCATCGGAGG AAGGAGAATG GAGTTAACAG

H00375751 GGTGTAAAAG TTCCTGGTGA ATATCGAAGA AAGGAGAACG GTGTTAATAG

TCCTAGGCTG GACCTGACGC TTGCTGAGCT CCAGGAGATG GCATCTCGCA

TCCTAGGCTG GACCTGACGC TTGCTGAACT CCAGGAAATG GCATCTCGCA

TCCTAGGATG GATCTGACTC TTGCTGAACT TCAGGAAATG GCATCTCGCA

TCGAGGCCCA GCAACAAATG CTGGCTACTA AGGAG

TCGAGGCCCA GCAACAAATG CTGGCTACTA AGGAG

TTGAAGCCCA GCAACAATTG CTGGCAACTA AGGAA

>Ortholog Group 201, Repeat 2

3 135

M00112508 CAACGCTTAA AGTTTTTAAA ACAGCAAGAT CAACGTGCTG CTGAACAGGA

R00004330 CAACGCTTAA AGTTTTTAAA ACAGCAAGAT CAACGTGCTG CTGAACAGGA

H00375751 CAGCGCTTAA AGTTTTTGAA ACAACAAGAT CAGCGAGTTG CTGAGCAGGA

GAAACTTAAG AGGCTTAGAG AAATAGCTGA AAGTCAGGAA GCTAAGCTTA

GAAACTTAAG AGGCTCAGAG AAATCGCTGA AAGTCAGGAA GCCAAGCTCA

GAAACTTAAA AGGCTAAAAG AAATAGCTGA GAATCAGGAA GCTAAGCTAA

AGAAAGTGAG AGCGCTAAAG GGCCATGTGG AGCAA

AGAAAGTGAG GGCGCTGAAG GGCCATGTGG AGCAG

AAAAAGTGAG AGCACTTAAA GGCCACGTGG AACAG

>Ortholog Group 202, Repeat 1

3 123

M00000811 ATGGACACTT CTGGGCACTT CCATGACTCG GGGGTGGGGG ACCTGGATGA

H00271915 ATGGACACTT CTGGGCACTT CCATGACTCG GGGGTGGGGG ACTTGGATGA

R00028117 ATGGACACTT CTGGGCACTT CCATGACTCG GGGGTGGGGG ATCTGGATGA

AGACCCCAAG TGTCCCTGTC CATCTTCTGG GGACGAGCCA CCACCGCCAG

AGACCCCAAG TGCCCCTGTC CATCCTCTGG GGATGAGCCA CCACCGCCAG

AGACCCCAAG TGTCCCTGTC CATCTTCTGG GGACGAGCCA CCACCGTCAG

CGCCACCAGC AGTCCCCCAG CAG

CGCCACCAGC AGCCCCCCAG CAG

CGCCACCAGC AGTCCCCCAG CAG

>Ortholog Group 202, Repeat 2

3 135

M00000811 CCGGGACCCT TGCTGCAGCC TCAGCCTCCG CAGCCTCCAC TGCACCCCCT

H00271915 CTGGGACCCT CGCTGCAGCC TCAGCCTCCG CAGCTTCCAC CGCATCCCCT

R00028117 CCGGGACCCT TGCTGCAGCC TCAGCCTCCG CAGCTTCCAC TGCACCCCCT

GCCTCAGCTT GCCCAACTCC AGAGCCAGCT TGTCCATCCT GGTCTGTTGC

GTCTCAGCTC GCCCAACTCC AGAGCCAGCC CGTCCACCCT GGCCTGCTGC

GCCTCAGCTT GCCCAACTCC AGAGCCAGCT TGTCCATCCT GGTCTGTTGC

ACTCTTCTCC CACGGCGTTC AGGGCCCCCA CTTCA

ACTCCTCTCC CACCGCTTTC AGGGCCCCCC CTTCG

ACTCTTCTCC CACGGCTTTC AGGGCTCCCA ATTCA

>Ortholog Group 202, Repeat 3

3 198

M00000811 GAAGATCTAG AAAAGCAGAT TGGCAGCCTG GAGTCCAAGC TGGAGCACCT

H00271915 GAAGACCTGG AGAAGCAGAT TGGCAGCCTG GAGTCGAAGC TGGAGCATCT

R00028117 GAAGACCTGG AAAAGCAGAT TGGCAGCCTG GAATCCAAGC TGGAGCACCT

CACAGCCAGT TTCAATTCCC TGCCCCTGCT CATCGCAGAT ACCCTGCGCC

CACCGCCAGC TTCAACTCCC TGCCGCTGCT CATCGCCGAC ACCCTGCGCC

CACAGCCAGC TTCAATTCCC TGCCCCTGCT CATCGCAGAC ACCCTGCGCC

TGCTCACTGC CTTCGTGGAG GCCCGGGGCA TCAGTGTGGC CGTGGGAACT

TCCTGTCTGC CATCATCGAG GCCCGGGGTG TCAGCGTGGC AGTGGGCACC

TGCTCACTGC CTTCGTGGAG GCCCGGGGCA TCAGTGTGGC TGTGGGAACT

AGCCACGCCC CTCCCTCTGA CAGCCCTATC GGGATCAGCT CCACCTCT

ACCCACACCC CAATCTCCGA TAGCCCCATT GGGGTCAGCT CCACCTCC

AGCCACGCCC CTCCCTCTGA CAGCCCTATC GGGATCAGCT CCACCTCT

>Ortholog Group 203, Repeat 1

3 198

M00035417 ---------- ---------- ---------- GGGCCAGCAG GATACCTGAG

H00408891 AGACCCAGAG AGGTGTGCAG TGGCATGGAA GGGCCAGCGG GGTATCTGCG

R00027700 ---------- ---------- ---------- GGGCCAGCGG GATACCTTCG

ACGTGCCAGT GTGGCCCAGC TGACCCAGGA GCTGGGCACT GCCTTCTTCC

GCGGGCCAGT GTGGCCCAAC TGACCCAGGA GCTGGGCACT GCCTTCTTCC

ACGTGCGAGT GTGGCTCAAC TGACCCAGGA GCTGGGCACT GCCTTCTTCC

TGCCCGCAGC TATGGCGGAC ACCTTCCTGG AACACCTCTG CCTTCTGGAT

TGCCAGCTGC TATGGCAGAC ACCTTCCTGG AACACCTCTG CCTACTGGAC

TGCCCGCAGC TATGGCGGAC ACCTTCCTGG AACACCTCTG CCTTCTGGAT

ATCGACTCAG AGCCTGTGGC CGCTAGGAGC ACCAGCATCA TTGCCACC

ATTGACTCCG AGCCCGTGGC TGCTCGCAGT ACCAGCATCA TTGCCACC

ATCGACTCAC AGCCTGTGGC TGCTCGTAGC ACCAGCATCA TTGCCACC

>Ortholog Group 204, Repeat 1

3 117

R00030216 CAGCTTCTGG TCATGATCCA TCAGCTCTCC GCCCTTCGGG ACCAGCTCCT

H00356174 CAGCTTCTGG TCATGATTCA CCAGCTGTCC ACCCTGCGGG ACCAGCTCCT

M00092130 CAGCTTTTGG TCATGATCCA TCAGCTCTCT GCCCTTCGGG ACCAGCTCCT

TACGGCCCAC TCTGAGCAGA AGAACATGGC TGCCATGCTG TTTGAGAAAA

GACAGCCCAC TCGGAGCAGA AGAACATGGC TGCCATGCTG TTTGAGAAGA

TACGGCCCAC TCTGAGCAGA AGAACATGGC CGCCATGCTG TTTGAGAAGA

TGGAACTGGC CAGGCAG

TGGAGCTTGC CCGGCAG

TGGAACTGGC CAGGCAG

>Ortholog Group 204, Repeat 2

3 117

R00030216 CAGGAGCAGA TCGCCAAGCT GATTCAGCAG CAGCACAAGA TCAACCTCCT

H00356174 CAGGAGCAGA TTGCAAAGCT GATTCAGCAG CAGCATAAGA TCAACCTCCT

M00092130 CAGGAGCAGA TCGCCAAGCT GATTCAACAG CAGCACAAGA TCAACCTCCT

CCAGCAGCAG ATCCAGCAGG TTAACATGCC TTATGTCATG ATCCCGGCCT

TCAGCAGCAG ATCCAGCAGG TTAACATGCC TTATGTCATG ATCCCAGCCT

GCAGCAGCAG ATCCAGCAGG TTAACATGCC TTACGTCATG ATCCCAGCCT

TTCCCTCAAG TCACCAG

TCCCCCCAAG CCACCAA

TCCCCCCAAG CCACCAG

>Ortholog Group 205, Repeat 1

3 102

R00015827 TCTCCTCAGT CAAGCTGCCC ATCCCCCACC ATTCCAGCCA GTAAAGTCAT

M00036720 TCTCCCCCGT CAGGCTGCCC ATCACCCACC ATTCCAGCAA GTAAAGTCAT

H00381897 TCCCCGCAGT CAGGCTGCCC ATCACCCACC ATTCCAGCAG GTAAAGTCAT

TTCCCCATCA CAGAAGCACA GCAAAAAGGC ATTAAAGCAG GCCCTGAAGA

TTCTCCATCA CAGAAGCACA GCAAAAAGGC ATTAAAACAG GCCTTGAAGA

TTCTCCATCA CAGAAGCACA GCAAGAAGGC ACTAAAGCAG GCGCTAAAGA

AG

AG

AG

>Ortholog Group 205, Repeat 2

3 114

R00015827 AAGCAGCAGC AGCAGTGCAG GCCAAGCATG TCCATC---T CCAATCAGCA

M00036720 AAG------- -----TGCAG ACCAAGCATG TCCATC---T CCAATCAACA

H00381897 AAG------- -----TGCAG GCCAAGCATA TCCATCTCCT CCAACCAGCA

CCTCTCTCTC AAGACTGTCA AAACAGCCAG TGACTCTGTA CCTGCCAAAC

CCTCTCTCTC AAGACTGTCA AAGCAGCCAG TGACTCTGTA CCTGCCAAAC

TCTCTCACTA AAGACTGTCA AAGCAGCCAG TGACTCTGTA CCTGCCAAAC

CTGGT----- ----

CTGGA----- ----

CTGCAACATG GGAA

>Ortholog Group 206, Repeat 1

3 198

H00304108 TGCACCCTGC TTCTCGGCCT GGCCGTGGTG CTGCTGAAAG CGCGGCTGGT

M00062702 TGCGCCCTGC TCCTCGGCCT GGCCGCTGTG CTGCTGAAGG CGCGGCTGGT

R00006504 TGCGCCCTGC TCCTCGGCCT GGCCGCGGTG CTGCTGAAGG CGCGGCTCGT

CCCCGCGGCC GCCAGAGCGG AACTCAGCCG CTCCGACCTC AGCCTCATCA

CCCCGCGGCC GCTAGAGCCG AACTCAGTCG CTCCGACCTC AGCCTCATCA

CCCCGCGGCC GCTAGAGCCG AACTCAGCCG CTCCGACCTC AGCCTCATCA

AGCAGCTGGA GGAGGCTGAG GAGGAGAGGA CAGAGGTGCC TGGGGCAACC

AGCAGCGGGA GGAGGCAGAG GAGGGGAGGC CGGAGGTGCC TGGGGCATCC

GGCAGCGGGA GGAGGCGGAG GAGGAGCGGA TGGAGGTGGC TGGGGCATCC

TCCACCTTGA CGGTTCCAGT GTCTGTATTT ATGTTGAAAG TCCAGGTG

TCTACTTTGG TGGCTCCAGT GTCCGTATTT ATGCTGAAAG TCCAGGTC

TTTACTTTGG TGGTTCCAGT GTCTGTATTT ATGCTGAAAG TCCAAGTA

>Ortholog Group 207, Repeat 1

3 156

M00107403 CAGTACACGC CTGTGCCTCC GACAGCTGTT TCTATTGAAG GTGTTGTTGC

H00373277 CAGTACACGC CTGTGCCTCC GACAGCTGTT TCTATTGAAG GTGTTGTTGC

R00041726 CAGTACACGC CTGTGCCTCC GACAGCTGTT TCTATTGAAG GTGTTGTTGC

TGATACCTCT CCCCAGACGG TGGCCCCCTC ATCCCAGGAC AGCAGTGGTT

TGATACCTCT CCCCAGACAG TGGCACCTTC ATCCCAGGAC ACCAGTGGTA

TGATACCTCT CCCCAGACGG TGGCCCCCTC ATCCCAGGAC AGCAGTGGTT

TAGCAGTGGA CACACCCAGT GAACACGCAC CGGCATACTC CTTCCAACAG

TAGCAGTGGA CACATCCAAC GAACATGCAC CTGCATATTC TTACCAACAG

TAGCAGTGGA CACACCCAGT GAACACGCGC CGGCATATTC ATTCCAACAG

TCCAAA

TCTAAA

TCCAAC

>Ortholog Group 208, Repeat 1

3 201

R00006554 AAGGATGGGC TCTTCCGGGT GGACAAGGGT GCTAGCAACA ACCCCGAATT

M00026269 AAGGATGGGC TCTTCCGGGT GGACAAGGGT GCTGGCAACA ACCCAGAATT

H00273317 AAGGATGGGC TCTTCCGAGT GGACAAGGGT GCAGGCAACA ACCCCGAGTT

TGAGGAAACT CGAAGGGTGT TCGCGACCAA GATGGCCAAA ATCCACCTCC

TGAGGAAACT CGAAGGGTGT TCGCGACCAA GATGGCCAAA ATCCACCTCC

TGAGGAAACT CGCAGGGTGT TCGCCACCAA GATGGCCAAA ATCCACCTC-

AGCTCCTACA GGAGGAGGCC CTACCTAGGG CAGGCAGAAG CCCCATCAAC

AGCTCCTACA GGAGGAGGCC CTACCTAGGG CAGGCAGAAG CCCGGTCAAC

--CTCCTGCA GGAGGAGACT CTGCCCAGGG GGAGTAGAGG CCCTGTCAAT

GGTGGGAACC GTCAGGGT-- ---------- ---------G TGAGCAGCAA

GGTGGGAACC GTCAGGGT-- ---------- ---------G CGAGCGGCAA

GGAGGGGGCC GCCTGGGCCC ACAGGCCCGT TGGGAAGTTG TGGGCAGCAA

G

G

G

>Ortholog Group 209, Repeat 1

3 231

M00042269 TTGTCCAACA TGCTGCAGAG ACGCTCAGGC GCCATGCTGC AGCCACCCTC

R00054649 TTGTCCAACA TGCTGCAGAG ACGCTCAGGC GCCATGCTGC AGCCACCCTC

H00417235 CTGTCAAACA TGCTACAGCG GCGCTCAGGC GCCATGATGC AGCCGCCTTC

CCTTCATGCA GTCACGTCTC AGCAACAGCT GCTACAGATG AAGCTTCTG-

CCTTCATGCA GTCACGTCTC AGCAGCAGCT GCTACAGATG AAGCTTTTGC

TCTTCATGCA ATCACATCGC AGCAGCAGTT GATACAGATG AAGCTTCTG-

-----CAGCA GCAACAGCAG CAGCAGCAGC AGCGGCTTCT CAGGCAGGCC

AGCAGCAGCA GCAGCAACAG CAGCAGCAGC AGCGGCTTCT CAGGCAGGCC

---------- ---------- ---------- --CGACTTCT CAGGCAAGCC

CAGACTCGAC CTTTCCAACA GGGCCAGCCA GGGGACCAGG CTGCTCTCTT

CAGACTCGAC CTTTCCAACA GGGCCAGCCA GGGGACCAGG CTGCTCTCTT

CAGACTCGGC CTTTCCAACA GGGCCAGCCG GGGGACCAGG CTGCTCTCTT

TACTGCACAA GCACGGCCCT CCCCTCAGCT C

TACTGCACAA GCACGGCCCT CCCCTCAGCT C

TGCTGCGCAA GCACGGCCCT CCCCTCAGCT C

>Ortholog Group 209, Repeat 2

3 186

M00042269 CAGCCCCAGC AGCCCTCACA GACCCAGAGT CAGGCCCTTG GTCTCCAAGC

R00054649 ---------- ---------- ---------- ---------- ----------

H00417235 CAGCCCCAGC AGTCCTCGCA GTCCCAGAGT CAGACCCTTG GTCTCCAAGC

AATGCAGCCC CAGCAGCCTT TGTTCCCCAG GCAAGGCTTG CAGCAGACCA

---------- ---------- ---------- ---------- ----------

AATGCAGCCC CAGCAGCCCT TGTTTCCCAG GCAAGGCTTG CAGCAGACCA

CAGCCGCCCT GGTGCGACAG CTCCAAAAGC AGCTTTCTAG TAACCAGCCA

---------- ---------- ---------- ---------- ----------

CGGCCGCCTT GGTGCGGCAG CTCCAGAAGC AGCTTTCCAG CAACCAGCCA

CAGCAAGGAG TGACTCCCTG TGCACACCCT TCACAC

---------- ---------- ---------- ------

CAGCAAGGAG TGACTCCGTA TGGGCATCCT TCACAC

>Ortholog Group 20, Repeat 1

3 198

R00060564 TCCAAGTCCT CCAGCACCGT GACCTCCACC AGTGGCCACT CTTCAGGGAG

H00263551 TCCAAGTCCT CCAGCAACGT GACCTCCACC AGCGGTCACT CTTCAGGGAG

M00124133 TCCAAGTCCT CCAGCACCGT GACCTCCACC AGTGGACACT CTTCAGGGAG

CTCGTCAGGA GCCATCGCCT ACCGTCAACA GCGGCCAGGC CCCCACTTCC

CTCATCTGGA GCCATCACCT ACCGGCAGCA GCGGCCGGGC CCCCACTTCC

CTCTTCAGGA GCCATCGCCT ACCGTCAGCA GCGGCCAGGC CCCCACTTCC

CCCTCAATCT CAGCCAGGCT CAGCAGCACA TGGCTGCAGA CCGCACTGGG

CACTCAATCT CAGCCAGGCT CAGCAGCACA TCACCACGGA CCGCACTGGG

CCCTCAATCT CAGCCAGGCC CAGCAGCACA TGGCTGCGGA CCGCACCGGG

AGTCACCGTC GGCAGCAGGC CTACATCACT CCTACCATGG CTCAGGCT

AGCCACCGAA GGCAGCAGGC CTACATCACT CCCACCATGG CCCAGGCT

AGTCACCGTC GACAGCAGGC CTACATCACT CCGACCATGG CGCAAGCT

>Ortholog Group 210, Repeat 1

3 198

H00410708 CATGATACTG GAGAATGTCG GCTAGAAAAA CTCAGCAGCA ACATCACTGT

M00110477 CATGATACTG GGGAATGTCG CCTAGAAAAG CTCAGCAGCA ACATCACTGT

R00003208 CATGATACTG GGGAATGTCG CCTAGAAAAA CTCAGCAGCA ACATCACTGT

AAAAAAAACA AGAGTTGAAG GAAGCAGTAA AATTCAGTAT CGTAAAGAAA

GAAAAAAACA AGAGTGGAAG GGAGTAGCAG AATCCAGTAC AGACTAGAAA

GAAAAAAACA AGAGGAGAAG GAAGTAGCAA AATCCAGTGC AGACTAGAAA

TGTGGAATTC AGCCAGGACT CCCAATCTTG TAAAACATTC TCCATCTGAA

TGTGGAATCT GCCTAGGACT TCCAATCTTG TACAGCATTC TCCATCAGAA

TGTGGAATCC ACCCAGGACA TCCAACCTTG TACAGCATTC TCCATCAGAA

GATAAGATGT CCCCAGCATC TCCAATAGAT GATATCGAAA GAGAACTG

GAGAAGATGT CTCCAACGTC TCTAATGGAT GATATTGAAA GAGAACTG

GATAAGCTGT CCCCAACGTC TCTAATGGAT GATATTGAAA GAGAGCTG

>Ortholog Group 211, Repeat 1

3 198

M00001081 ATTCACAGCA GCGTCTCTCG AGTGGGCAAA GCCATTGACA GGAACTTCGA

R00036701 ATTCACAGCA GCGTCTCCCG AGTGGGCAAA GCCATTGACC GGAACTTTGA

H00320623 ATTCACAGCA GTGTATCCCG AGTGGGCAAA GCCATTGACA GGAACTTCGA

CTCTGAGATC TGCGGCGTGG TCTCGGATGC TGTGTGGGAC TCCCGTGAGA

CTCTGAGATC TGCGGCGTGG TCTCAGATGC TGTGTGGGAC TCCCGTGAGA

CTCTGAGATC TGTGGTGTTG TGTCAGATGC GGTGTGGGAC GCGCGGGAAA

TCCTGCAGAT GGCCATCGTA GAGCACCTGT ACCAGCAAGG CATGCTCAGT

TCCTGCAGAT GGCCATTGTC GAGCACCTGT ACCAGCAGGG CATGCTCAGC

TCCTGCAGAT GGCCATCGTG GAACACCTGT ATCAGCAGGG CATGCTCAGC

GTAGCGGAGG AGCTGTGCCA GGAATCGACA TTGAATGTGG ACCTGGAC

GTAGCTGAGG AGCTGTGCCA GGAATCAACA TTGAATGTGG ACCTGGAC

GTGGCCGAGG AGCTGTGCCA GGAATCAACG CTGAATGTGG ACTTGGAT

>Ortholog Group 212, Repeat 1

3 198

M00115753 GGGAATGCTG AGGCTGGACC TCCAGACCCA GATGAATCTG CTGTCCTTTT

R00001084 GGGAATGCAG AGGCTGGACC TCCAGACCCG GATGAATCTT CTGTCCTTTT

H00274853 GGGACTGTAG AGGCTGGACC TCCAGACCCG GATGAGTCTG CGGTCCTTCT

GGAGGCTATT GGACCAGTAC ATCAGAATCG ATTCATCCAA CAGGAGCGAC

GGAGGCTATT GGGCCAGTAC ATCAGAACCG ATTCATCCAA CAGGAGCGAC

GGAGGCCATC GGGCCAGTGC ACCAGAACCG ATTCATCCGG CAGGAGCGGC

AGAGAAACGA GGTGCTTGGT GATAGGAAGG CTGGGCCTCT GGAGGTTCTA

AGCGAAACGA GGTGCTTGGT GATAGGAAGG CTGGGCCTCT GGAGGCTCTA

GGAGTGAAGA GCTGCTAGCA GAGAGAAAGC CTGGGCCTCT GGAGGCCCGG

GAAAGGAGAT CAAGTCCTGG TAATTTAAGA GATCAGAGTC CTAAGGGA

GAACGGAGAT CAAGTCCTGG TAATTTAAGA GATCAGAGCC CTAAGGGA

GAGCGGAGAC CCAGCCCTGG GGAGATGCGG GATCAGAGCC CCAAGGGA

>Ortholog Group 213, Repeat 1

3 198

M00124205 AAACAATATG AAGCATATGT TCAAGCTTTG GAGGGAAAGT ACACAGATCT

R00025936 AAACAATATG AAGCATATGT TCAAGCTTTG GAGGGAAAGT ACACAGACCT

H00351141 AAACAATATG AAGCATATGT ACAAGCTTTG GAGGGCAAGT ACACAGATCT

TAATTCAAAC GATGTGACTG GTTTAAGGGA ATCTGAAGAA AAACTAAAGG

TAATTCAAAC GATGTGACTG GCTTAAGGGA ATCTGAAGAA AAACTAAAGG

TAACTCTAAT GATGTAACTG GCCTAAGAGA GTCTGAAGAA AAACTAAAGG

AGTCTGCACG CAGGGAGAAC ATTCTTGTCA TGCGGCTAGC AACCAAAGAG

AATCTGCACG CAGGGAGAAC ATCCTTGTCA TGCGGCTAGC AACCAAGGAA

AGTCTGCACG CAGGGAAAAC ATCCTTGTAA TGCGACTAGC AACCAAGGAA

CAGGAGATGC AAGAGTGCAC CACTCAAATC CAGTACCTCA AGCAAGTT

CAGGAGATGC AAGAGTGCAC CACTCAAATC CAGTACCTCA AGCAAGTT

CAAGAGATGC AAGAGTGTAC TACTCAAATC CAGTACCTCA AGCAAGTC

>Ortholog Group 213, Repeat 2

3 198

M00124205 TTCATCATTC AACTTGATGA AGAAGTAGAG GGTATGCAGA GCACCATTCT

R00025936 TTCATCATTC AACTTGACGA AGAAGTAGAG GGTATGCAGA GCACCATTCT

H00351141 TTCATCATCC AGCTTGATGA AGAAGTAGAG GGTATGCAGA GTACCATTCT

AGTTCTTCAG CAACAATTGA AGGAGACACG ACAGCAGTTG GCACAGTACT

AGTTCTTCAG CAACAGTTGA AGGAGACTCG ACAGCAGTTG GCACAGTACT

AGTTCTGCAG CAGCAGCTGA AGGAGACACG CCAGCAGTTG GCTCAGTACT

CTCAAGCTTC AGCTCCAAGT ACCAGCAGGA CTACATCTTC TGAACCTGTA

CTCAAGCTTC TGCTCCAAGT ACCAGCAGGA CTACATCTTC TGAACCTGTG

CTCAGGCCTC TGCCCCAAGT ACCAGCAGGA CTACAGCTTC TGAACCTGTA

GATCAGGCAG AGGTCACAAG CAAAGACTGC AGTCGTCTGG CAAATGGA

GATCAGGCAG AGGCCACAAG CAAAGACTGC AGTCGTCTGG CAAATGGA

GAACAGTCAG AGGCCACAAG TAAAGACTGC AGTCGTCTGA CAAACGGA

>Ortholog Group 214, Repeat 1

3 201

H00275732 CAGTTTCTCC AGCTGGTCAG CAGCCGCCAG CTCCCACAGT GCGCG---CT

M00031727 CACTTTCTGC AGCTGGTTGG CAGCCGCCAG CTCCCGCAGT GTACGACGCT

R00001912 CACTTTCTGC AGCTGGTTGG CAGCCGCCAG CTCCCGCAGT GTGCGACACT

CCGAGAAAAG GCAGCTCTGG GGGACCTGAC ACCGCCACCA CCGCCGCCGC

CCGGGAAAAG GCAGCTATGG GGGACCTGAC GCCGCCG--- ----------

CCGGGAAAAG GCAGCTATGG GGGACCTGAC GCCGCCG--- ----------

CACTCACGGC ATTCCTGCAG CAGCTCCAGG CGCTCAAACC CCCCAGAGGC

--CTCACTAC GTTCCTGCAG CAGCTCCAGG CTCTCAAAAC CCCCAGAGGT

--CTCACTAC GTTCCTGCAG CAGCTCCAGG CTCTCAAAAC CCCCAGAGGT

GGGGACCAGA ACCTGCTCCC GACGATGAGC CGGTCCTTGT CGGTGCCAGA

GGAGACCAGA ACCTGCTCCC GACGATGAGC CGGTCCTTGT CGGTGCCAGA

GGGGACCAGA ACCTGCTCCC GACAATGAGC CGGTCCTTGT CGGTGCCAGA

T

T

T

>Ortholog Group 214, Repeat 2

3 147

H00275732 CAACTCCAGC TGCAACATAA ATTCCAGGAG CGCAGAGAAG TGGAGCTCAG

M00031727 CAGCTCCAAC TGCAGCATAA ATTTCAAGAG CGCAGAGAAG TGGAGCTCAG

R00001912 CAACTCCAGC TGCAACACAA ATTTCAAGAA CGCAGAGAAG TGGAGCTCAG

GGCGAAGCGG GAGGAAGAGG AACGCAAGCG TCGAGAGGAG AAGCGCCGC-

GGCGAAGCGG GAGGAGGAGG AGCGCAAACG ACGGGAGGAG AAGCGGCGCC

GGTGAAGCGG GAGGAGGAGG AGCGCAAACG CCGGGAGGAG AAGCGTCGC-

-----GAGGA GCAGAAGCGG CGGCAGGAGG AGGAAGAGCT GTTTCGG

AACAGGAGGA GCAGAAGCGA CGGCAGGAAG AGGAGGAGCT CTTTCGG

--CAGGAGGA GCAGAAGCGG CGTCAGGAGG AGGAGGAGCT CTTTCGA

>Ortholog Group 214, Repeat 3

3 147

H00275732 CGCAAGCACG TGCGGCAGCA GGAGCTATTG CTGAAGTTGC TAGCG-----

M00031727 CGCAAGCAGG TACGGCAGCA GGAATTGTTG CTGAAGCTGC TAGCGACGAA

R00001912 CGCAAGCAGG TGCGGCAGCA GGAACTGTTG CTGAAGCTGC TAGCGACGAA

-GTCCCTGTG CCCCCCGCAC CCAGCTCCCC GCCCCCACTC TGGGCTGGCC

TGTCCCTGTG CCCCCTGCAC CCAGCTCCCC ACCCCCGCTC TGGGCTGGCC

TGTCCCTGTG CCCCCTGCAC CCAGCTCCCC ACCCCCACTC TGGGCTGGCC

TGGCCAAGCA GGGGCTGTCC ATGAAGACGC TCCTGGAGTT GCAGCTG

TAGCCAAGCA GGGCCTGTCC ATGAAGACTC TGCTGGAGCT GCAGATG

TAGCCAAGCA GGGCCTGTCC ATGAAGACTC TGCTGGAGCT GCAGATG

>Ortholog Group 214, Repeat 4

3 231

H00275732 GATTATATCC GTTCCTGCCT GGGGGACACG CTGGAAGCCA AAGAATTTGC

M00031727 GATTATATTC GTTCCTGCCT GGGGGACACG CTGGAAGCCA AAGAATTTGC

R00001912 GATTATATTC GTTCCTGCCT GGGGGACACG CTGGAAGCCA AAGAATTTGC

CAAACAATTC CTGGAGCGGA GGGCCAAGCA GAAAGCCAGC CAGCAGCGG-

CAAACAATTC CTGGAGCGGA GGGCCAAGCA GAAAGCCAGC CAACAGCGG-

CAAACAATTC CTGGAGCGGA GGGCCAAGCA GAAAGCCAGC CAACAGCGGC

---------- ---------- ---------- --GAGGCATG GCTGAGCAGC

--------CA GCAGCAGCAG CAGCAGCAGC AGGAGGCCTG GCTGAGCAGC

AGCAGCAACA GCAGCAGCAG CAGCAGCAGC AAGAGGCCTG GCTGAGCAGC

GCCTCGCTGC AGACGGCCTT CCAGGCCAAC CACAGCACCA AACTCGGCCC

ACCTCCCTAC AGACAGCCTT TCAGGCCAAC CACAGCACCA AACTGGGCCC

AGCTCCCTAC AGACAGCCTT TCAGGCCAAC CACAGCACCA AACTGGGCCC

CGGGGAGGGC AGCAAGGCCA AGAGGCGGGC A

TGGGGAGGGC AGCAAGGCCA AAAGGCGGGC A

TGGGGAGGGC AGCAAGGCCA AGAGGCGGGC G

>Ortholog Group 215, Repeat 1

3 204

H00308546 CCGCTCAGTC TCAATACTTG CACTGATGAG GGCCATGTAG TTCTTGCTTC

M00031740 CCGCTCAGTC TCAACACTTG CACTGACGAG GCCCATGTAG TGCTTGCCTC

R00001871 CCACTCAGTC TCAACACGTG CACTGATGAA GCCCATGTAG TGCTTGCTTC

GCCACTCAAG ACTGGTCGGA AGCGGCATAG ACACCGGGGA CAGCACCAC-

ACCACTCAAG ATTGGTCGCA AGCGCCATAG ACACCGGGGA CCGCACCACC

ACCACTCAAG ATTGGTCGCA AGCGCCATAG ACACCGGGGA CCGCACCAC-

--GCAGCCGG AGGGAGTGAG AGTCAC---C CCGTGCCGCC CACAGCCCCT

AGGCGTCTGG AGGGAATGAT AGCAACGCCG CCGTGCTGCC CACTGACCCT

--GCATCTGG AGGGAATGAT AGCCAA---G CTGTGCTGCC CACTGACCCT

CTCACCCCCT TACTCCACGG GGAGGGCGCC TCACAGCAGC CGCGGCACAG

CTCACGCCCT CACTCCATGG GGAGGGTGCT ACGCAGCAGC AGCAAAACAG

CTCACACCCT CAGTCCATGG GGAGGGTGCT ACG------C AGCAGCACAG

GGGC

AGGC

AGGC

>Ortholog Group 216, Repeat 1

3 237

R00062282 ATGCAGAGCA ATAAAACCTT TAACTTGAAG AAGCAGAATC ATACTCCAAG

H00362963 ATGCAGAGTA ATAAAACTTT TAACTTGGAG AAGCAAAACC ATACTCCAAG

M00033673 ATGCAGAGCA ATAAAGCCTT TAACTTGGAG AAGCAGAATC ATACTCCAAG

GAAGCACCAT AAGCATCACC ACCAGCAGCA CCATCAGCAG CAGCAGCACC

AAAGCATCAT CAACATCACC ACCAGCAGCA GCAC------ ----------

GAAGCATCAT CAGCATCACC ACCAGCAGCA CCAT------ ----------

ATCAGCAACA GCAGCAACAA CAGCAGCAAC AGCAGCAACA GCCACCCCCA

---------- ---------- ---------- -----CACCC GCCACCACCG

---------- ---------- ---------C AGCAGCAACA GCCACCCCCA

CCAATACCTG CAAATGGACA GCAGGCCAGC AGCCAGAATG AAGGCTTGAC

CCAATACCTG CAAATGGGCA ACAGGCCAGC AGCCAAAATG AAGGCTTGAC

CCAATACCTG CAAATGGCCA GCAGGCCAGC AGCCAGAATG AAGGCTTGAC

TATTGACCTG AAGAATTTTA GGAAACCAGG AGAGAAG

TATTGACCTG AAGAATTTTA GAAAACCAGG AGAGAAG

TATTGACCTG AAGAATTTTA GGAAACCAGG AGAGAAG

>Ortholog Group 217, Repeat 1

3 198

R00053782 ---------- ---------- ---------- ---------- ----------

M00055107 GCCCAGCGCC AGGAGCTGCT GGCCAAAGTG GAAGAGGTAG TGAGGAATTA

H00276185 GCCCAGCGCC AAGAGCTGCT GGCCAAGGTA GAAGAGGTGG TGAGGAACTA

---------- ---------- ---------- ---------- AACTTGGGA-

CACCTTCCTG CTGCGCGCCG CTGAGGAGTC CACAGCCCGG AACCTTAAC-

CACCTTCCTG CTGCGTGCAG CTGAGGAGTC CACAGCCCGT AACCTTAACG

---------- ---------- ---------- ---------- ----------

--CCACCGCT GCCAGCAGCC ACAGGGCACC ACCCACCAGA CTCCCCG---

TGGCAGCAGC TGCAGGGGCA GCCACAGAGC ATCCACCAGG CTCCCCAACT

---------- ---------- ---------- ---------- --------

TCATCGACTG TTATGAGCAC ATTCACACGC TCCTTAAAAA CCCTTATT

TCGGCGACTG TTATGAGCAC ATTCACCCAC TCCTTAAAAA CCCTTATT

>Ortholog Group 218, Repeat 1

3 138

M00022698 ATGGAGGAGC CAGCTGTGAA GCAGGGCTTC CTGCATCTTA CCTTTGGCAA

R00018725 ATGGGGGAGA TGGCCGTGAA GCAAGGCCTC CTGCATCTCA CCTTTGGCAA

H00276420 ATGGGAGACG GGGCAGTGAA ACAAGGCTTC TTGTATCTTA CGTTTGGAAA

GAAGTGGCGC CGGTTCGCAG CCGTGTTATA TGGAGAGTCT GGCTGTGCCC

GAAGTGGCGC CGGTTCACAG CCGTGTTATA TGGAGAGTCT GGCTGTGCCC

GAAATGGCGC CGCTTCGGCG CCTCACTGTA TGGAGGGTCG GACTGCGCCT

TAGCCAGACT AGAGCTCCAG GATGTCCCCG AGAAGACA

TAGCCAGACT AGAGCTCCAG GATGGCCCGG AGAAGACA

TGGCCCGGCT GGAGCTGCAG GAGGGCCCGG AGAAGCCT

>Ortholog Group 219, Repeat 1

3 198

H00277225 CAGAGTGAAA GCATTTCTTC CTCACTGAAT GAAGGTGTGG TGTCTTATGA

R00025799 ---------- ---------- ---------- ---------- ----------

M00095677 CAAAGCGAAA GCCTGTCTTC CTCGTTGAAT GAAGGAATGG TATCTTACGA

GAGCTCAAGC ATCAATGGTA GAAAGTCAGG AGTCATGTTG GATCCCTTGC

---------- ---------- ---------- ---------- ----------

GAGCTCCAGC ATCAATGGTA GAAAGTCGGG AGTCATGTTG GACCCCTTAC

CACCGCAGCC ACCACCACCG CCGCCGCCAC CACCACCATC ACAGCCACAG

---------- ---------- ---------- ---------- ----------

CACCACAGCC ACCTCCGCCA CTACCACCAC CACCACCACC ACCATCACAG

CCACTGCAGC AGCCACAGCC ACCACAGCTG CAGCCACCAC ATCAGGTG

---------- ---------- ---------- ---------- --------

CCACTGCCGC AGCCTCCACC TCCACCGCTG CAGTCACCAC ACCAGGTG

>Ortholog Group 21, Repeat 1

3 105

H00162391 CTCTATAAGT CCATGCTGGA GAAGTCCTCT TCCTCCTCTC AGCACGGCTT

M00003238 CTCTACAAGT CCATGCTGGA GCGG---TCC TCCTCCTCCC AGCACGGCTT

R00062121 CTCTACAAGT CCATGCTGGA GCGG---TCC TCCTCCTCCC AGCACGGCTT

TTCTTCTCTC CTGGGGGACA TCCCACCCTC GAACAACTAC TACATGTATC

TTCCTCACTC CTGGGAGACA TGCCACCTTC TAACAACTAC TACGTGTAC-

TTCCTCACTC CTTGGAGACA TGCCACCTTC CAACAACTAT TACATGTACC

CACCG

-----

AGCAG

>Ortholog Group 21, Repeat 2

3 57

H00162391 CCACCT---- ---------- ---------- ---------- --CCGCCACA

M00003238 ---------- ---------- ---------- ---------- --CAGCCACA

R00062121 CAGCAGCAGC AGCAGCAGCA GCAGCAGCAG CAGCAGCAGC AGCAGCCTCA

GCCACCT

GCCACCA

GCCCCCA

>Ortholog Group 21, Repeat 3

3 114

H00162391 CAGCAGTCCC AGCCAGCACC TGCCCAGGGC CCCTCAGCTG TAGGGGGTGC

M00003238 CAGCAACCAC AACCAGCACC CACTCAGGGC CCCTCAAATG TAGGGGGTGC

R00062121 CAGCAACCAC AACCGGCACC CACTCAGGGC CCCTCAAATG TAGGGGGTGC

TCCTCCACTG CACACCCCAA GCACAGATGG TTGTACCCCA CCAGGGGGAA

CCCTCCACTG CACACCCCAA GCCCTGATGG CTGTACTACA CCAGGGGGAA

CCCTCCACTG CACACCCCAA GCCCCGATGG CTGTACTACA CCAGGGGGAA

AGCAAGCTGG GGCG

AACAAGCGGG CGCT

AACAGGCAGG AGCT

>Ortholog Group 220, Repeat 1

3 9

M00108960 ATGTTCAAC

R00062979 ATGTTTAAC

H00377232 ATGTTCAGC

>Ortholog Group 220, Repeat 2

3 9

M00108960 TTGCAGCAG

R00062979 ------CAG

H00377232 CTC---CAG

>Ortholog Group 220, Repeat 3

3 6

M00108960 CAGCTC

R00062979 CAGCTC

H00377232 CAGTTA

>Ortholog Group 220, Repeat 5

3 99

M00108960 ATACTGCAGC TCCAACAGCT GCTGCAACAG TCCCCACCAC AGGCCTCCTT

R00062979 ATACTCCAGC TCCAACAGCT ACTGCAGCAG TCCCCACCAC AGGCCTCCTT

H00377232 TTACTGCAGC TCCAGCAGCT GCTCCAGCAG TCCCCACCAC AGGCCCCGTT

GTCCATTCCT GTCAGCCGGG GCCTCCCCCA GCAGTCATCC CCGCAACAG

GTCCATGCCT GTCAGCCGGG GCCTCCCCCA GCAGTCATCC CCGCAACAG

GCCCATGGCT GTCAGCCGGG GGCTCCCCCC GCAGCAGCCA CAGCAGCCG

>Ortholog Group 221, Repeat 1

3 201

H00372035 AAACAGCCAT CCAAGTCTTC GGTTCAGGGG CTACCCGCAG GACCAAACAG

R00014031 AAGCAGCCGG CCAAGTCTTC CACCCAGGGG CTGCTGGTGG GACCAAACAG

M00022531 AAGCAGCCAT CCAAGGCTTC CACCCAGGGG CTGCTGGTGG GACCAAACAG

TGACACT--- TCCCTGGATG CCAAAGTCCT GGGGAGCAAA GATGCCACCA

TGACACTTCT TCCCTGGATG CCAAAGTCCT GGGGAACAAA GATGCCTCCA

TGACACT--- TCCCTGGATG CCAAAGTCCT GGGGAGCAAA GATGCCTCCA

GGATGAGAGC CACCCCAAAG TTCGGACCTT ATCAGAAAGC CTTGAGGGAA

GGATGAGAGC CACCCCGAAG TTTGGACCTT ATCAAAAAGC GCTCAGGGAA

GGATGAGAGC CACCCCGAAG TTTGGACCTT ATCAAAAAGC TCTCAGGGAA

ATCAGATATT CCTTGTTGCC TTTTGCTAAT GAATCGGGCA CCTCTGCAGC

ATCCGATATT CCCTCCTGCC TTTTGCCAAT GAGTCAGGCA CGTCGGCAGC

ATCCGATATT CCCTCCTGCC TTTTGCCAAC GAGTCAGGCA CTTCGGCAGC

T

T

T

>Ortholog Group 222, Repeat 1

3 204

R00009832 TTGGACCCTG AGCCTCAACA CTTACCTTTG ACAGCGCTAT TTGGGAAACA

M00108397 TCGGACCCTG AGCCTCAGCA CTTATCTTTA ACAGCACTAT TTGGGAAACA

H00280665 TTAGACCCTG AACCCCAACA CTTATCCTTG ACAGCTCTGT TTGGGAAGCA

AGACAAAGCC CCTTGTCAGG AAACTGTGAA ACCATCCCGG ACCTTTGCCC

AGACAAAGCT CCCTGTCAGG AAACTGTAAA GCCCTCCCGG ACCTTTGCC-

GGACAAAGCT ACATGTCAGG AAACTGTGGA GCCTCCGCAG ACTCTC----

ACCACGAGAA GCTTCCAGTT CATCACGGGG TTGCATGCTC CCTGGCCTGT

--CACGAGAC GCGTCCAGTT CATCACGGGG TCGCATGCTC CCTGTCCTGT

--CACGAGAA GCTTCCAATT AGGCAGGGGG TTGTACGCTC CCTGTCCTAT

GAGGATCCCA GGAAGCTCTC ACTCCCAGTG GAGAAGCAGC TATGCCCAGC

GAGGAGCCCA GGAAGCTCTC ACTCCCGGTG GAGAAGCAGC TCTGCCCGGC

GAGGAACCCA GAAGACACTC ACCCCCCATT GAGAAGCAGC TCTGTCCAGC

CATT

CATT

CATT

>Ortholog Group 223, Repeat 1

3 198

H00361467 GGGCAGCTGC TGCGGGAGCG AAACCTGCTG CAGCAGTCAT GGGAGGACAT

M00087879 GGGAAGCTGC GGAGGGAGCG CAATCTGCTG CAGCAGTCCT GGGAGGACAT

R00007828 GGGAAGCTGC GGAGGGAGCG GAACCTGCTG CAGCAGTCCT GGGAAGACAT

GAAGCGGCTC CACGAGGAGG ACCAGAAGGA GATCGGTGAC CTCCGTGCCG

GAAGCGGCTC CGGGAAGAGG ACCAGAAGGA GATAGGTGAC CTCCGGGCCG

GAAGCGGCTC AGGGAGGAGG ACCAGAAGGA GATAGGTGAC CTCCGGGCCG

TGTTGAAGCA CAACGGGTCA TCCGAGATTC TCAACAAACT GTATGACACG

TGCTGAAGCA CAATGGCTCA TCAGAGATCC TCAACAAGCT GTACGACACG

TACTGAAGCA CAATGGCTCA TCAGAGATCC TCAATAAGCT GTACGACACG

GCCATGGACA AGTTGGAGGT GGTCAAGAAG GACTATGACG CCCTTCGG

GCCATGGACA AGCTGGAGGT AGTCAAGAAG GACTACGATG CCCTGCGG

GCCATGGACA AGCTGGAGGT AGTCAAGAAG GACTACGATG CTCTGCGG

>Ortholog Group 224, Repeat 1

3 198

H00385450 GAAGACCCTG TCTATGGTAT CTACTATGTA GACCACATCA ACAGGAAGAC

R00033083 ---------- ---------- ---------- ---------- ----------

M00086730 GAAGACCCTG TCTACGGTGT CTACTATGTA GACCACATCA ACAGGAAGAC

ACAATATGAG AACCCGGTTC TAGAAGCCAA ACGGAAGAAG CAGCTTGAGA

---------- ---------- ---------- ---------- ----------

GCAATATGAA AACCCAGTCC TAGAAGCCAA ACGGAAGAAA CAGCTTGAAC

CAGAAGAATG GACAGAAGAT CACTCAGCCC TTGTGCCTCC TGTTATTCCA

---------- ---------- ---------- ---------- ----------

CAGAAGAGTG GACAGAGGAT CATGCATCTG TTGTGCCTCC TGTTGCTCCT

AACCACCCTC CAAGCAATCC AGAGCCAGCC AGAGAAGTTC CACTTCAG

---------- ---------- ---------- ---------- --------

TCCCATCCCC CGAGCAATCC GGAGCCAGCC AGGGAAACTC CACTTCAG

>Ortholog Group 225, Repeat 1

3 198

H00299466 CAGTTCTCGC AGGGCGCGCG CGCGGCAGGC GGCTCGGGAG CAGGTGGAGG

M00025457 CAGTTCTCAC AGGGTGCACG TGCGGGCGGC ACCACAGGCG CTGGTGGCAG

R00037246 CAGTTCTCAC AGGGTGCGCG TGCAGGCGGC ACCACAGGCG CTGGTGGCAG

CGTGGCAGCT GCAGCCGTGC CCCTGATCCT GGAACAGCTC ATGGCCCTGA

CGTGGGCGCG GTGGCCATCC CCATGATCCT GGAGCAGCTG GTGGCGCTGA

CGTGGGTGCT GTGGCCATCC CCATGATCCT AGAGCAGCTG GTGGCACTGA

TCCACCAGCT GCAGCTCATC GAGCAGATCC GCAGCCAGGT GGCCCTCATG

TCCACCAACT TCAGCTCATC GAGCAGATCC GCAGCCAGGT GGCCCTGATG

TCCACCAGCT TCAGCTCATC GAGCAGATCC GCAGCCAGGT GGCCCTAATG

CAGCGCCCGC CGCCGCGGCC CTCACTCAGC CCCGCGGCCG CCCCGAGC

AGCCGG---C AGCCTGGGCC TCCATTGAAG CCCTCAGCC- -----AGT

AGCCGG---C AGCCTGGGCC TCCGCTGAAG CCCTCAGCC- -----AGT

>Ortholog Group 226, Repeat 1

3 198

M00034031 GCCTGCAGGC TCCAGAAATG CCTCCAAGCC GGCATGAACT TAGGAGCTCG

R00045942 GCCTGCAGGC TCCAGAAATG CCTTCAAGCT GGCATGAACT TAGGAGCTCG

H00421481 GCTTGCAGAC TTCAGAAATG TCTTCAAGCT GGAATGAATT TAGGAGCACG

AAAGTCAAAG AAGCTGGGGA AGTTAAAAGG CCTTCACGAG GAGCAGCCA-

AAAGTCAAAG AAGCTGGGGA AGTTAAAAGG CCTCCACGAG GAGCAACCA-

AAAGTCAAAG AAGTTGGGAA AGTTAAAAGG GATTCACGAG GAGCAGCCAC

-----CCCCC ACCGCCACCA CCCCAGAGCC CAGAAGAGGG GACCACATAC

-----CCCCC ACCACCGCCA CCCCAGAGTC CAGAAGAGGG GACCACATAC

CCCCACCCCC ACCCCCACCC CCGCAAAGCC CAGAGGAAGG GACAACGTAC

ATTGCTCCTA CCAAGGAGCC ATCAGTGAAC TCTGCGCTGG TCCCGCAG

ATCGCTCCGA CCAAGGAGCC ATCGGTGAAC TCTGCGCTGG TCCCGCAG

ATCGCTCCTG CAAAAGAACC CTCGGTCAAC ACAGCACTGG TTCCTCAG

>Ortholog Group 227, Repeat 1

3 198

M00050820 TCGGGGGGCA GCGGCACGGC GCTGGTCACC AAGGAGAGCA AATTTCGGGA

H00282388 TCGGGGGGCG GCGGCACAGC CCTGCTCAAC AAGGAGAACA AATTCCGGGA

R00050683 ---------- ---------- ---------- ---------- ----------

CCGCTCGTTC AGCGAGAACG GGGAGCGCAG CCAGCACCTC CTGCACCTGA

CCGCTCGTTT AGCGAGAACG GCGATCGCAG CCAGCACCTC CTGCACCTGA

---------- ---------- ---------- ---------- ---------A

AGGGGGGCAG CGGCTCCCAG ATCAACTCCA CGCGCTACAA GACGGAGCTG

AGGGGGGCGG CGGCTCCCAG ATCAACTCCA CGCGCTACAA GACCGAGCTG

AGGGGGGCAG CGGCTCCCAG ATCAATCCCA CA---TACAA GACCGAGTTG

TGCCGGCCCT TCGAGGAGAG CGGCACGTGC AAGTACGGCG AGAAGTGC

TGCCGGCCCT TCGAGGAGAG CGGCACGTGC AAGTACGGCG AAAAGTGC

TTCTGGCCCT TG-------- ---------- AAGGTACAGG AGAAGTGG

>Ortholog Group 227, Repeat 2

3 198

M00050820 GCCTTCGCTT TCGGCCCGGA GCTGAGCAGC CTCATCACGC CGCTCGCCAT

H00282388 GCCTTCGCCT TCGGTCCGGA GCTCAGCAGC CTCATCACGC CGCTCGCCAT

R00050683 ACCTTCACCT TTGGCCTGGA GCTGAGCTGC CTAATCACGC CACTTGCCAA

CCAGACCCAC AACTTCGCCG CCGCGGCCGC CGCCGCCTAC TACCGCAACC

CCAGACCCAC AACTTTGCCG CCGTGGCCGC CGCCGCCTAC TACCGCAGTG

CAAGACCCAC AGCTTCGCCG CC---GCAGT TGCCGCCTAC TACCGCAGCC

CCGCGCCACC CCCGGCACAG CCCCCCGCGG CCCCC----- ----------

GCCTGGCGCC CCCCGCGCAG CCGCCGGCGC CGCCCAGCGC GACCCTCCCC

CCGCGCCGCC CCCTGCACAG CCCGCCATGA CCCCC----- ----------

---------- --GCGCCGCC CTCCCCGCCC TTCGGCTTCC AGCTGCCG

GCCGGGGCCG CCGCACCTCC CTCGCCGCCC TTCAGCTTCC AGCTGCCG

---------- --ACACCAGC TTCTCGGCCC TTCAGCTTCC AGCTGCTG

>Ortholog Group 228, Repeat 1

3 213

H00339764 CCCAAGAGAC TTCACAGCGC TGATATAAGT CCCCAAGACG AACAAGGCGG

R00002746 CCCAAGAGAC TTCACAGCAC TGATGCCAAT CCCCAAAGCG AGCAAGGGGG

M00108583 CCCAAGAGAC TTCACAGCAC TGATGCGAAT CCCCAAGACG AGCAAGGGGG

CGGCGTGGGC ACCAAGGACC ACGGGGGCGG CGGCGGCGGT ----------

CGGCGTGGGC ACCAAGGACC AAGGCGGCGG TGGTGGTAAA GAGCAGCAGC

CGGCTTGGGC ACTAAGGACC ACGGCGGCGG CGGCGGTGGT ------AAAG

-----GGCAA AGACGAGGCA CCTTTAGGCC CGGGTCCCAA GTTTTGCAAA

AGCAGCAACA GCAGGAGACG CCATTGATCC CGGGCCCCAA GTTCTGCAAA

AGCAACAGCA GCAGGAGGCG CCGTTGATCC CGGGCCCCAA GTTCTGCAAA

GCCGGCCCCC TCCACCACTA CCCATCCCCC TCCCCGGAAA GCAGCAACCC

GCCGGCCCCA TACACCACTA CCCCGCGTCG TCCCCGGAGG CGAGCAACCC

GCCGGCCCCA TACACCACTA CCCCGCGTCA TCCCCGGAGG CGAGCAACCC

ATCCGCTGCC GCC

GCCGGGTGCC GCG

GCCGGGCTCC GCG

>Ortholog Group 229, Repeat 1

3 198

H00422040 GATGAAGAGC CAGTAAAAAA ATTGTTAGAA AGCAGATACC ATCAAATTGG

R00003106 GACGAAGAGC CAGTAAAGAA ACTGTTAGAA AGCAGATACC ATCAGATAGG

M00121005 GACGAAGAGC CAGTAAAGAA ACTGTTAGAA AGCAGATACC ATCAAATAGG

TTCTGGGAAG TGTGAAATCA AAGTTGCACA ACCCAAAGAG GTATATAGGA

CTCTGGGAAG TGTGAAATCA AAGTTGCACA ACCCAAAGAG GTGTACAGGA

TTCTGGGAAG TGCGAAATCA AAGTTGCGCA ACCCAAAGAG GTGTACAGGA

AAGGTGGAAG AGGTGCTGCA GCTGGTGGAC GAGGTGGTAC GAGGGGTCGT

AAGGTGGAAG AGGGGCTGCG GCTGGTGGAA GAGGTGGTGC TAGGGGACGC

AAGGAGGCAG AGGGGCTGCA GCCGGCGGAA GAGGAGGTGC TAGGGGGCGT

GGCCGAGGTC AGGGCCAAAA CTGGAACCAA GGATTTAATA ACTATTAT

GGAAGAGGTC AGGGCCAAAA CTGGAACCAA GGATTTAATA ACTATTAT

GGAAGAGGTC AGGGCCAAAA CTGGAACCAA GGATTTAATA ACTATTAT

>Ortholog Group 22, Repeat 1

3 204

M00021606 GATCTCTCTT CAGAAGAGCT GCGGAGGAGA CGAGAAGCCT ACTTTGAAAA

H00387442 AATCTTACTT CAGAAGAGCT TCGGAAGAGA CGAGAAGCCT ACTTTGAAAA

R00007505 GATCTTTCTT CAGAAGAGCT GCGGAAGAGG AGAGAAGCCT ACTTTGAAAA

G--------- ---------- ---------- ---------- ---------G

ACTTCGTCTC AAAAAAAAAG AGAAAGAAAA CTTCATACAG CAGCAAAAGG

G--------- ---------- ---------- ---------- ---------G

TAGACCGACC TGGACCCCTT TCATATCCAC GTGAAAGACC GACCACAAGT

GGGACCTATC AGGACAGAGT TCACATCCAT GTGAAAGGCC AGCCACCAGT

CAGACCGACC TGGATACCTT TCATACCCAT GTGAAAGACC CACCACAAGT

TCAGGAGGAC GTAGGAGCGA CCAAGGAGGC GACGCTGTGA GTGAAGAGGA

TCAGGAGCAC TTGGGAGTGA TCTA---GGT AAGGCCTGCT CA---CCATT

TCAGGAGGAC TCCGGAGCAA CCAAGCAGGC AATGCTATGA GTGAAGAGGA

CATG

CATC

CGTG

>Ortholog Group 230, Repeat 1

3 216

H00282753 GCGGCGACCA CTCCGCCTCT GCCGTCCCAC CTGACCGCAG AGGAGACCCC

M00037255 GTGGCCACCA CGCCACCTCT GCCACCCCAC CTTAGCGCAG AAGAGACGCC

R00019319 GTGGCCACCA CACCACCTCT GCCACCCCAT CTGACCGCAG AAGAGACCCC

CCTCTTCCTG GCCGAACCAG CCCTCCCCAA GGGCTTGCCC CCTCCTCTC-

CTTGTTCCTG GCTGATTCTG TCATCCCCAA GGGCTTGCCT CCTCCTCTCC

CCTGTTCCTG GCTGATTCCG TCATCCCCAA GGGCTTGCCT CCTCCTCTCC

--CCCCCTCC ACAGCAG--- ---------- --AAATCGCT GATGGACCAG

CGCCGCCCCC TCAGCCGCCT CCGCAGCAGC CCAAATCCCT GATGGACCAG

CGCCGCCCCC TCAGCAGCCC CCGCAGCAGC CCAAGTCCCT GATGGACCAG

CTCCAGGGAG TGGTCAGCAA CTTCAGTACC GCGATCCCGG ATTTTCACGC

CTGCAAGGCG TGGTCACCAA CTTTGGCTCT GGGATTCCAG ACTTCCACGC

CTGCAAGGCG TAGTCACCAA CTTCGGTTCG GGGATTCCAG ATTTCCATGC

GGTGCTGGCA GGCCCC

GGTGCTGGCA GGCCCG

GGTGCTGGCA GGCCCG

>Ortholog Group 231, Repeat 1

3 201

R00018359 ---------- ---------- ---------- ---------- ----------

M00048158 CAAAAGGAGA TCCTGTCGGT GCTGGGGCTC CCGCACAGGC CGCGGCCCCT

H00283147 CAGAAGGAGA TCTTGTCGGT GCTGGGGCTC CCGCACCGGC CCCGGCCCCT

---------- ---------- ---------- ---------- ----------

GCACGGTCTC CAGCAGCCTC AGCCCCCGGT GCTCCCGCCA CAGCAGCAGC

GCACGGCCTC CAACAGCCGC AGCCCCCGGC GCTC---CGG CAGCAGGAGG

---------- ---------- ---------- ---------- ----------

AGACGGCCCG CGAGGAGCCC CCTCCAGGGC GGCTGAAGTC CGCTCCACTC

AGCTGCCTCG CGGAGAGCCC CCTCCCGGGC GACTGAAGTC CGCGCCCCTC

---------- ---------- ---------- ---------- ----------

TTCATGCTGG ATCTCTACAA CGCCCTGTCC AATGACGACG AAGAGGATGG

TTCATGCTGG ATCTGTACAA CGCCCTGTCC GCCGACAACG ACGAGGACGG

-

G

G

>Ortholog Group 232, Repeat 1

3 201

M00047571 CAGGGCTTCC AGGAGGGGGA AGACGAGCTC GGCGACGAGG AGGAGGGCGC

R00046783 CAGGGCTTCC AGGAGGGGGA GGACGAGCTC GGCGACGAAG AGGAGGGCGC

H00283179 CAGGGTTTCC AGGAAGGGGA AGATGAGCTC GGGGACGAAG AGGAAGGCGC

GGGCGACGAG AACGGTCACG GGGAGCAGCA GTCCCAACCG CCGGCAGCGG

GGGCGACGAG AACGGCCACG GGGAGCAGCA GTCCCAACCG CCGGCGGCGG

GGGCGACGAG AACGGGCACG GGGAGCAGCA GCCTCAACCG CCGGCGACG-

CGCCTTCCCA GCAGCGTGGT GCCGGCAAGG AGGCCGCGGG CAAGAGCAGC

CGGCTTCTCA GCAGCGCGGG CCTGGCAAGG AGGCCGCGGG GAAAAGCAGC

--CCCCAACA GCAGCGCGGG GCCGCCAAGG AGGCCGCGGG GAAGAGCAGC

GGCCCCACCT CGCTCTTCGC GGTGACGGTG GCGCCGCCAG GGGCGAGGCA

GGCCCCACCT CGCTCTTCGC GGTGACGGTG GCGCCGCCAG GGGCGAGGCA

GGCCCCACCT CGCTGTTCGC GGTGACGGTG GCGCCGCCCG GGGCGAGGCA

G

G

G

>Ortholog Group 233, Repeat 1

3 198

M00024627 AACAAGGTTC AAGCAAAACC ACCAAAAGTC GACGGAGCCG AGTTTTGGAA

R00019358 AACAAAGTTC AAGCAAAACC ACCAAAAGTT GATGGAGCTG AGTTTTGGAA

H00284049 AACAAAGTTC AAGCAAAACC ACCGAAAGTT GATGGAGCTG AGTTTTGGAA

ATCTAGCCCC AGTATTCTGG CTGTCCAGAG ATCTGCAATG CTTAGGAAG-

ATCTAACCCC AGTATTCTGG CTGTTCAGAG ATCTGCAATG CTTAGGAAG-

ATCTAGTCCT AGTATTCTGG CCGTTCAGAG ATCTGCAATC CTCAAGAAGC

--CCAGCTTC ATCTAATAGT GGATCCGAAG AAGACTCGTC CAGCAGTGAA

--CCAGCTTC ATCTAATAGT GGATCCGAAG AGGATTCCTC TAGCAGTGAA

ATCAAGCCTC ATCTAATAGC GGATCAGAAG AGGATTCCTC TAGCAGTGAA

GACTCCGACG ACTCGTCCAG CGGTGCCAAG AGGAAGAAGC ACAATGAT

GACTCAGACG CCTCGTCCAG CGATGTCAAG AGGAAAAAGC ATAACGAT

GATTCCGATG ACTCATCAAG TGAGGTCAAA AGGAAAAAGC ATAAAGAT

>Ortholog Group 234, Repeat 1

3 198

M00023433 AACGCGGCCA AGCCTGACTT AGATAAGGTC ATCAGTTTGA AGGAAGCTAA

H00284320 AATGCAGCCA AACCAGATTT AGATAAAGTC ATCAGTTTGA AAGAAGCTAA

R00002238 AACGCGGCCA AGCCTGACTT AGATAAGGTC ATCAGTTTGA AGGAAGCTAA

TGTGAAGCTT CGAGCAAATG CCCTTATCAA GAGAGGCACC ATGTGCATGC

TGTGAAGCTT CGAGCAAATG CTCTCATCAA AAGAGGCAGC ATGTACATGC

CGTGAAGCTT CGAGCAAATG CCCTTATCAA AAGAGGCACC ATGTGCATGC

CTATGCTGTC TACTCAGGAC TTCAACATGG CTGCCGAGAT CGATCCTATG

CTTTGCTGTC CACTCAAGAT TTTAACATGG CTGCTGACAT CGATCCTCAG

CTATGCTGTC TACTCAGGAC TTCAACATGG CTGCTGAGAT TGACCCTATG

AACTCAGACG TTTATCACCA CCGAGGACAG CTGAAAATTC TGCTGGAT

AATGCAGATG TTTATCACCA CCGAGGACAG CTGAAAATAC TCCTTGAT

AACTCGGATG TTTATCACCA CCGAGGACAG CTGAAAATCC TGCTGGAT

>Ortholog Group 235, Repeat 1

3 198

R00031772 ---------- ---------- ---------- ---------- ----------

H00409493 ACCCCTTCCC TGGTCACCTA CCTCAAGAAG GCTGGGAGGC CAGGCACCTC

M00020732 ACCCCTTCCC TAGTTACCTA CCTCAAGAAG GCCGGGGGTC CTGGGATCTC

---------- ---------- ---------- ---------- ----------

ACCACTGGCC AGCAAGGTTG GGGCCCCAGC AGCCCCCTCT GTGAAGCCAG

ACCCTTGGCC AGCAAGCATG AGGCCCAGGT GACCACGTCT GTGAAGCCAG

---------- ---------- ---------- ---------- ----------

AGCCACTGGC TGCTGTGCGC CCACCACTGG GAGACCTGAG CACCAAAGAC

AGCCAGTCGT GCCCACGTGC CCACCGCCGG GAGACTTGAG TGCAGCAGAT

---------- ---------- ---------- ---------- --------

CTGGGTGATC CCTCAATGGA CAAGGCAGCT GTGAAGATCC AGGCTGCC

TTGATGGATC CATCCCTGGA CAAGGCTGCT GTGAAGATTC AGGCTGCC

>Ortholog Group 236, Repeat 1

3 198

M00092548 ATGAAGAAAA ACCTAAGCAA GAAACAGGAA GAGATAGATA CAAACAGGAA

R00043154 ATGAAAAAAA AACTAAGCAG GAAGCAGGAA GAGATAGATG CAAGCAGGAA

H00391504 ATGAGAAAAA AACTAGAAAA GAAAAGAGAA GAGATGCATG AAAGCAGGAG

ACTGCTTCTT GACAATATGC ACAATAAAAA CATCATCCGA GCTGTAGAAG

GCGGTTTTTT GATAATATGC GTGATAAAAA CATCATCCGA GCTGTAGAAG

ACGGTTTCTT GAACATATGC AGGATAAACA TATTATCAAA GCTGTAGAAG

AGGAGGAAGA CGAAAAGATT AGAAAGTTTA TCAAAGCCAA AAAACGTCTT

AGGAAGAAGA GGAAAAGACT AGAAAGTTTA TCAAAGCCAA AAAACGTCTT

AGGAAGAAGA TGAAAAGATG AGAAAATTCA TCAAAGCAAA AAAGCGTCTT

ATACAAATGA GGATGGACAA AGATGCTGAA ACACATAGGC TAATGGAA

ATACAAATGA GGAAAGACAA AGAAGCTGAA ACACACAGGC TAATGGAA

ATACAAATGG GGAAAGAAAA AGAGGCTGAA ACACACAGGC TTATGGAG

>Ortholog Group 237, Repeat 1

3 213

H00390475 GGGTCAGGGA CAGGGGTTGG GGCCATGCTT GCTCGGGGCT CTGCTTCGCC

M00025444 GGGTCAGGGA CAGGGGTTGG GGCCATGCTT GCTCGGGGCT CTGCTTCCCC

R00020472 GGGTCAGGGA CAGGGGTTGG GGCCATGCTT GCTCGGGGCT CTGCTTCGCC

CCACAAATCC TCTCCGCAGC CCTTGGTGGC CACACCCAGC CAGCATCAC-

CCACAAATCT TCTCCACAGC CCTTGGTGGC CACACCTAGC CAGCACCAC-

CCACAAATCT TCTCCACAGC CCTTGGTGGC CACACCTAGC CAGCATCACC

---------- ----ATCAAA CGGTCAGCCC GCATGTGTGG TGAGTGTGAG

--CACCAACA GCAGATCAAA CGATCAGCTC GGATGTGTGG TGAGTGCGAG

AGCAACAGCA GCAGATCAAA CGGTCAGCTC GGATGTGTGG TGAGTGTGAG

GCATGTCGGC GCACTGAGGA CTGTGGTCAC TGTGATTTCT GTCGGGACAT

GCCTGCCGAC GCACTGAGGA CTGTGGCCAC TGTGACTTCT GCCGTGACAT

GCATGCCGAC GCACTGAGGA CTGTGGCCAC TGTGACTTCT GTCGTGACAT

GAAGAAGTTC GGG

GAAGAAGTTT GGG

GAAGAAGTTT GGG

>Ortholog Group 238, Repeat 1

3 198

H00285199 CCACATGTCA AGCTGGCGGT GGAGTGGGAT AGCTCTGTCA AGGAGCGCCT

M00021288 CCTCATGTCA AGCTGGCTGT GGAATGGGAC AGCTCTGTCA CAGAGCGCCT

R00060701 CCTCATGTCA AGCTGGCTGT GGAATGGGAC AGCTCTGTCA CAGAGCGCCT

GTTCGGGAGC CTCCAGGAGG AGCGAGCGCA GGATGCCGAC AGTGTGTGGG

GTTTGGGAGC CTCCAGGAGG AACGGGTCCA GGATGCAGAC AGTGTGTGGG

GTTTGGGAGC CTCCAGGAGG AACGGGTCCA GGATGCAGAC AGTGTGTGGG

CGCATCAGCA GCACAGCTGT ACCTTGGATG AATGTTTTCA GTTCTACACC

CACACCAGCA GCCCAGCTGT ACCCTGGACG AATGTTTTCA GTCCTATACC

CACACCAGCA GCCCAGCTGT ACCCTGGACG AATGTTTTCA GTCCTATACC

AAGGAGGAGC AGCTGGCCCA GGATGACGCC TGGAAGTGTC CTCACTGC

AAGGAGGAAC AGCTGGCCCA GGATGATGCA TGGAAGTGCC CTCACTGC

AAGGAGGAGC AGCTGGCCCA GGATGACGCA TGGAAGTGCC CTCACTGC

>Ortholog Group 239, Repeat 1

3 198

M00040936 CCCCTGCCAA CATTAGATGT GTTCGCTGGC TGCCAGATTC CGTACCCCAA

R00000713 CCCCTGCCAA CATTAGATGT GTTCGCTGGC TGCCAGATTC CATACCCCAA

H00357907 CCTTTGCCAA CATTAGATGT ATTTGCCGGC TGCCAGATTC CATACCCCAA

AAGAGAATTC CTTAATGAAG ATGAACCAGA AGAGAAAGGT GACAAGAACA

AAGAGAGTTC CTCAATGAAG ATGAACCGGA AGAGAAAGGT GACAAGAACA

ACGAGAATTC CTTAATGAAG ATGATCCTGA AGAAAAAGGT GACAAGAATA

ACCCACATCA GCAACCTGCA GCCCCCGCAC AGCAGACAGC AGCCCCCCCA

ACCCACATCA GCAGCCTGTA GCCCCCCAAC AGCAGGCAGC AGCCCCCACA

ACCAGCATCA GCAGCCCACA GCCCCTCCAC AGCAGGCAGC AGCCCCTCCA

CAGGCTCCTC CACCACAGCA GAGCAGTGCC CAAACCAATG GAACTGCT

CAGGCTCCTC CACCACAGCA GAGCAGTGCC CAGACCAATG GAACTGCT

CAGGCGCCCC CACCACAGCA GAACAGCACC CAGACCAACG GGACCGCA

>Ortholog Group 23, Repeat 1

3 198

H00251636 GAGTGGAGTG ATGCAGAAAA GAAAAGGGAA GAATTAAATA AAATGGAAAC

R00013854 GAGTGGAGCG ATGAAGAAAA GAAAAAGGAG GAACTGAATA AAATGGAAAC

M00035244 GAGTGGAGTG ATGAAGAGAA GAAAAGGGAA GAACTCAATA AAATGGAAAC

CAATAAACCA CGTGATCTTT TTATTGCCAA ACTTCTGAAT AAACTGAAAC

CAATAAACCT CGGGACCTCT TTATTGCCAA GCTACTGAGT AAGTTGAAGC

CAACAAACCA CGGGATCTTT TTATTGCCAA GCTCTTGAAT AAGTTGAAGC

ATTCTGAAAA TAAGAGAGAA AACTCTGAAG ATCCCGAGGA ATCTTGGGAA

ATCCTGAAAA TAAGACGGAG ACCGCGGAGG ATCCAGAGGA GTCTTGGGAA

GTCCTGAAAG CGAGAAAGGG GGCTCCGAGG ATCCCGAGGA ATCCTGGGAA

AATTTAGTTT CGGATGAGGA TTTTTCTGCA CTGTCCTTGG AATCAGCA

AACTTGGTTT CGGATGAGGA TCTGTCTGCA CTGTCCTTGG AGCCCACA

AACTTAGTTT CCGACGAAGA TCTTGCTGCG CTGTCCTTGG AGCCAACC

>Ortholog Group 240, Repeat 1

3 105

H00384983 CAAAGGACCA GCTCGGGCTC GGACACGGAC ATCTATAGCT TCCATTCGGC

R00004800 ---------- ---------- ---------- ---------- ----------

M00030039 CAAAGGACCA GTTCTGGTTC AGACACGGAC ATCTACAGCT TCCACTCCGC

TACGGAGCAA GAGGATTTGC TTTCAGACAT CCAGCAGGCG ATCCGCCTGC

---------- ---------- ---------- ---------- ----------

TACGGAGCAG GAGGATTTGC TCTCAGACAT CCAGCAGGCA ATTCGTCTGC

TCCAG

-----

TG---

>Ortholog Group 240, Repeat 2

3 105

H00384983 CAGCTCCTCC AGGGCGCCGA GGAGCCTGCA GCGCCCCCCA CTGCCGTCTC

R00004800 ---------- ---------- ---------- ---------- ----------

M00030039 ------CTCC AGGACTCCGA GGAGCCTGCA GCGCCCCCCA CTGCCATCTC

CCCTCAGCCC GGGGCCTTCC TGGGCCTGGA CCGGTTCCTG CTGGGGCCGA

---------- ---------- ---------- ---------- ----------

CCCTCAGCCT GGGGCCTTTC TGGGCCTGGA CCAGTTCTTG CTGGGACCTA

GCGGC

-----

GAAGC

>Ortholog Group 241, Repeat 1

3 99

H00286523 CAACCCTTCC AGCTGGCATT CGGCCACCAG GTGAACCGGC AGGTCTTCCG

R00014158 CAACCCTTCC AGCTGGCATT TGGCCACCAG GTGAACCGCC AGGTCTTCCG

M00105923 CAACCCTTCC AGCTGGCATT TGGCCACCAG GTGAACCGTC AGGTCTTCCG

GCAGGGCCCA CCGCCCCCAA ACCCGGTGGC TGCCTTCCCT CCACAGAAG

GCAGGGCCCT CAGCCCTCTA ACCCCACCAC CTCCTTCCCG CCCCAGAAG

GCAGGGCCCT CAGCCCTCTA ACCCCACCGC CTCCTTCCCA CCTCAGAAG

>Ortholog Group 241, Repeat 2

3 99

H00286523 GCAGCCCTAC CCCAGATGCC GCTCTTTGAG AACTTCTATT CCATGCCGCA

R00014158 GCAGCCCTGC CCCACATGCA GCTATTTGAG AACTACTACC CCATGCATCA

M00105923 GCAGCCCTGC CCCAGATGCA GCTATTTGAG AACTACTACC CCATGCATCA

GCAACCCTCG CAGCAACCCC AGGACTTTGG CCTGCAGCCA GCTGGGCCA

GCCGCCTTCA CAGCAGCACC AGGACTTTAC CCTGGCACCG GGCGGGCCC

ACTGCCTTCG CAACAGCACC AGGACTTTGG CCTGGCACCA GGTGGGCCA

>Ortholog Group 242, Repeat 1

3 198

M00044472 CCAGGGCCAG GCCCGGGAGC ATCAGAAGAC AGAGATGGAA GGCAGCAACA

R00047258 CCAGGCCCAG GCCCAGGAGG ATCAGAAGAC AGAGATGGAA GGCAACAGCA

H00392558 CCAGGGCCAG GCCCAGGGGG TCCTGAAGAC AGAGACGGAA GGCAACAGCC

ACCACAGCAG CAGCCACCGC CACAGCAGCA GCAGCAGCAG CAGCAGCCG-

ACCGCAGCAG CAGCAGCAGC AACAGCAGCA GCAGCAGCAG CAGCAGCAGC

GCCGCAGCAG CCACAGCAGC AGCCACAGCC GCAGGCGCCC CAGCAACCAC

-----CCATC ACAGCAGCCA GCGCCAGCTC AGCAGCAGCC ACCGCAGTTT

CAGCGCCATC CCAGCAGCCA GCGCCAGCTC AGCAGCAGCC ACAGCAGTTT

CACCACCATC ACAACAGCCT CCACCAACAC AGCAGCAGCC ACAGCAGTTT

AGAAATGATA GCAGGCAGCA GTTCAACTCA GGTAGAGACC AAGAAAGG

AGAAATGATA ACAGGCAGCA GTTCAACTCA GGTAGAGACC AAGAAAGG

AGAAATGATA ACAGGCAGCA GTTCAATTCA GGTAGAGACC AAGAAAGG

>Ortholog Group 243, Repeat 1

3 99

H00380376 AATTTAGAAC AGCAGGTGAA TCACAGCCAG CAGGGACATA CAAATGCCAA

R00062305 AACCTAGAGC AGCAGGTGGC ACATGTACAG CAGGGACACC CCAGTGCCAG

M00002291 AGCCTAGAGC AGCAGGTGGC CCGTGGCCAG CAGGGACACC CCAATGCCAG

TGCAGTGCTG TTTAGCCAAG TGAAAGTGAC TCCAGAGACA CACATGCTA

TGCTGTGCTC TTTGGCCAGG CTAAAGGTGC TCCTGAGACA CACACGTTG

TGCTGTGCTG TTTGGCCAGG CTAAAGGTGC TCCCGAGACA CACGTGTTG

>Ortholog Group 243, Repeat 2

3 21

H00380376 CACCCGGTTT TACACCTTCA G

R00062305 CACCCAGCCC TGCACCTCCA G

M00002291 CACCCAGCCC TGCACCTCCA G

>Ortholog Group 243, Repeat 3

3 54

H00380376 CCCCAGCAGA TAATGCAGCT C--------- ATCTCTCAG- --CAACCTTA

R00062305 CCC---CAGA TCATGCAGCT C--------- CAGCAGCAGC CCCAGCCTTA

M00002291 CCC---CAGA TCATGCAGCT CCAGCAGCAG CAGCAGCAGC CCCAGCCCTA

CCCC

CCCC

CCCC

>Ortholog Group 243, Repeat 4

3 99

H00380376 CAGCCGCCGC ATCCATTTTC AGCCCATCCG CATCAGTTTT CACAGCAACA

R00062305 CCGCCATCAC ATCAGTTCCC AGTCCATCAG CATCAGTTCT CTCAGCAGCA

M00002291 CCTCCATCAC ATCAGTTCCC GGTCCATCAG CATCAGTTCT CTCAGCAGCA

GCTACAGTTT CCACAGCAAC AGTTGCATCC TCCACAGCAG CTGCATCGC

GCTCCAGTTT CCACAGCAAC CATTACACCC TCAGCAGCAG TTGCATCGC

GCTCCAGTTT CCACAGCAAC CATTACACCC TCAGCAGCAG TTGCATCGT

>Ortholog Group 243, Repeat 5

3 93

H00380376 CCTCAGCAGC AGCTCCAGCC CTTTCAGCAG CAGCATGCCC TGCAGCAGCA

R00062305 CCTCAACAGC AACTGCAGCC ATTTCAGCAG CAGCATGCCC TGCAGCAGCA

M00002291 CCTCAACAGC AGCTGCAGCC ATTTCAGCAG CAGCATGCCC TGCAGCAGCA

GTTCCATCAG CTGCAGCAGC ACCAGCTCCT TGCCCAGCTC CAG

GCTCCATCAG TTGCAGCAGC AACAGCTCTT AGCACAGCTC CAG

GCTTCATCAG TTGCAGCAGC AGCAGCTCTT AGCACAGCTC ---

>Ortholog Group 243, Repeat 6

3 15

H00380376 CAGCACAGCC TGCTC

R00062305 CAGCAGCAGC AGCAG

M00002291 ------CAGC AACAG

>Ortholog Group 243, Repeat 7

3 6

H00380376 CTCCAG

R00062305 CAGCAG

M00002291 CAGCAG

>Ortholog Group 243, Repeat 8

3 45

H00380376 ATGCACATGC AAAGTCAGAC AGCGCCACAC TTGAGTCAGA CGTCA

R00062305 CTCCAGATGC AGAATCAAGC AGCA---CAC TTGAGCCAAA CGTCC

M00002291 CTCCAAATGC AGAATCAAGC AGCA---CAC TTGAGCCAAG CGTCC

>Ortholog Group 243, Repeat 9

3 138

H00380376 CAGGCGCTGC AGCATCAGGT TCCACCTCAG CAGCCCCCGC CACCACCATC

R00062305 CAGGCACTAC AACACCAGGT TCTACCCCAG CAG---CCCC CACCTCAGCA

M00002291 CAGGCACTAC AGCACCAGGT TCTACCCCAG CAG---CCCC CACCTCAGCA

GCCTCAGCAG CATCAGCTTT TTGGACATGA TCCAGCAGTG GAGATTCCAG

GCAGCAGCAG CAGCAGCTTT TTGGACATGA CCCAGCAGTG GAGATTCCAG

ACAGCAGCAG CAGCAGCTTT TTGGACATGA CCCAGCAGTG GAGATTCCAG

AAGAAGGCTT CTTATTGGGA TGTGTGTTTG CAATTGCG

AAGAAAGCTT CTTGCTGGGC TGTGTGTTTG CCATTGCG

AAGAAAGCTT CTTGCTAGGA TGTGTGTTTG CAATTGCG

>Ortholog Group 244, Repeat 1

3 198

H00287934 CTGCTGGAGG CTCCGCTGCT GCTGGGGGTC CGGGCCCAGG CGGCGGGCCA

R00021979 CTGCTGGAGG CTCCTCTGCT TTTGGGGGTC CGAGCGCAGG CGGCG-----

M00058629 TTGCTGGAGG CTCCTCTGCT TTTGGGGGTC CGAGCGCAGG CGGCG-----

GGGGCCAGGC CAGGGGCCCG GGCCGGGGCA GCAACCGCCG CCGCCGCCTA

-------GGC CAGGTATCCG GGCCGGGCCA GCAAGCTCCG CCGCCGCCCG

-------GGC CAGGTATCCG GGCCGGGCCA GCAAGCCCCG CCGCCGCCCA

GCGGGCAGCA GTACAACGGC GAGCGGGGCA TCTCCGTCCC GGACCACGGC

GCGGGCAGCA GTACAACGGC GAACGGGGCA TCTCCATCCC GGACCACGGC

GCGGGCAGCA GTACAACGGC GAACGGGGCA TCTCCATCCC GGACCACGGC

TATTGCCAGC CCATCTCCAT CCCGCTGTGC ACGGACATCG CGTACAAC

TACTGTCAGC CCATCTCCAT CCCGCTGTGC ACGGACATCG CGTACAAT

TACTGCCAGC CCATCTCCAT CCCGCTGTGC ACGGACATCG CGTACAAC

>Ortholog Group 245, Repeat 1

3 240

H00405400 CACCGCCTCA CCCTGGAAAG ACCAGCACTG CGATATCCTG AACGTTTGAA

M00050465 CAACGCCTCA CCCTGCAAAG ACCATCACTG CGGTATCCTG CTCGTCTGAG

R00035017 CAGCGCCTCA CCCTGCAAAG ACCATCACTG CGGTATCCTG ATTGCCTGAA

GAAATTA--- CATAACCTGA AGACAGAAGG TTACAGGAAA CAGCAGCAGC

GAAGCTTTGC CCCAACCTGA AG---CAAGG TGAGGGCCTT GCTGGGCATG

GAAGCTGCGC CACAACCTGA AG---CGA-- ---------- ----------

GGAAGAAGGT GAAAACACCT ATTAAGAAAC AGGAGGCTAA AAAGAAAGCC

GAGGGAAGGC GAAAACCTTT CCTCCGAAAC AGGAGCCTAA GAAGAAAGCC

-----GAGGA GAAAACCTCT ACTAAGAAAC AGGAGGCTAA AAAGAAAGCC

AAGAGTGATC CAGGGATCCA GAGCACTTCA ATTAAGAAAC AGGAGGCTAA

AAGAGAAATG TCAAGGGCCA GTATGGGGAA CCTCCGAAAC AGGAGCCTAA

AAGCGAGATG TTGAGGAACA GTATGGGGAA ACTAAGAAAC AGGAGGCTAA

AAAGAAAGCC AAGAGTGATC CAGGGATCCA GAGCACTTCA

GAAGAAAGCC AAGAGAAATG TCAAGGGCCA GTATGGGGAA

AAAGAAAGCC AAGCGAGATG TTGAGGAACA GTATGGGGAA

>Ortholog Group 245, Repeat 2

3 210

H00405400 CATAAACATC CAGTTACC-- ---------- ---ACCGTTC ATGACCGTCT

M00050465 GAAACCACGT CGGGCCAT-- ---------- ---TTCTTCC ATGACAGTGT

R00035017 GAAACCATGT CGAGCGATTC CTTCCATGAC CCTTCCTTCC ATGACCATGT

CTATGGTTAC AGAACTCTGC CAGGTCAGAA CAGTGACATG AAG-------

A---GGACTC AGA---ATAT CAGGACAGGA AGATCAACAA ACGGAG---G

A---GGACTC AGA---ATAT CACGACGTGA AATTGAACGA AAGGAGGTGG

--ATGGAAAA AGGAACCACT TCGAAACAGG ATTCCACGGA GAGACCA---

ACATAGAGAA AACATCTGCA GAGACACATG TTTCCTTAGA AGAA------

GAATGGAGAA AACATCCCTA GAGAGACGTG TTTCCGTAGA AGAGCCAATG

------AAGA TGAACTACTA TGACCATGCG GATTTCCACC ACAGTGTAAA

GTGTATGAGT TTGACAAGTA CTACACA--- GAATACTACG CCACTCCGGA

GCATATGAGT TCGGCAGCTA CTACACTGCC AGCTACTACA CCAGTCCTGA

AAGTCCTGAA

GAGCGCTGTA

GAGTGCTCTA

>Ortholog Group 246, Repeat 1

3 234

H00363654 GAGCAGCTGG ACGAGGAACA TCTGGAGTCG CACAAAAAGT ACAAGGAGCG

M00030049 GAGCAGCTGG ACGAGGAACA TCTGGAGTCG CACAGAAAGT ACAAGGAGCG

R00015576 GAACAGCTGG ATGAGGAACA TCTGGAGTCA CACAGAAAGT ACAAGGAGCG

CAAAGAGAGA AGGGCACAGC AGGAACAGTT GCTGCTGCAG ----------

CAAAGAGAAA AGGGCCCAGC AGGAGCAGCT ACAGTTGCAA CAGCAGCAGC

CAAAGAGAAA AGGGCACAGC AGGAACAGCT ACAGTTACAG CAACAGCAGC

---------- ---------- AAGCAGTTA- -----CCCCC ATCGCAGCTC

AGCAACAGCT GCAACAG--- CAACAGCTAC AGCTGTCCAC GTCCCAGCCC

AGCAGCAGCT ACAACAGTTG CAACAGTTGC AGCTGTCCAC CTCTCAGCTC

TGCACAGCCC CTGCCTCTTC TCATGAACGC GCAAGCATGA TTGACAAAGC

TGCACAGCCC CAGCT---GC TCACAAACAC CTAGATGGCA TTGAACACAC

TGCACAGCCC CAGCT---GC TCATGAACAC CTAGATAGCA TTGAACACAC

AAAGGAGGAC ATTGTCACAG AGCAGATAGA TTTC

AAAGGAGGAT GTGGTCACTG AGCAGATTGA CTTC

AAAGGAGGAT GTCGTCACTG AGCAGATTGA TTTC

>Ortholog Group 247, Repeat 1

3 207

R00060639 CGTTTTGAAG ACACCCTGGA GTTTGTAGGG TTTGATACGA AAATGGCTGA

M00110771 CGGTTTGAAG ACACCCTGGA GTTTGTAGGG ATTGATACGA AAATGGCTGA

H00384109 CGTTTTGAAG ACACCCTGGA GTTTGTAGGG TTTGATGCGA AGATGGCTGA

GGAATCTTCC TCCTCATCTT CCTCATCTTC ACCAACTGCT GCAACCTCA-

GGAATCTTCC TCCTCATCTT CCTCATCTTC GCCAACTGCT GCAACCTCAC

GGAATCCTCC TCCTCCTCCT CCTCATCTTC ACCAACTGCT GCAACATCT-

--CAGCAGCT TAAAAATAAG AGTATATTAA TTTCTTCGGT GGCTTCAGTG

AGCAGCAGCT TAAAACTAAG AGTATATTAA TTTCTTCTGT GGCTTCAGTG

--------CT TAAAAATAAG AGTATATTAA TCTCTTCTGT GGCTTCGGTG

CACCATGCAA ACGGCCTGGC TAAATCTTCT ACCACGGTCT CTAGCTTTGC

CACCATGCAA ACGGCCTGGC TAAATCTTCT ACCGCGGTCT CTAGCTTTGC

CATCATGCAA ACGGCCTAGC CAAATCTTCT ACCACCGTCT CTAGCTTTGC

TAACAGC

TAACAGC

TAACAGC

>Ortholog Group 248, Repeat 1

3 198

M00086949 GATACACTTG GGGCCCGAAA GAAGGTCTTT CCTCCTCCTC GCCCTCCTCC

R00003971 GATACTCTTG GGGCCCGAAA GAAGGCCTTT CCTCCCCCTC GCCCTCCTCC

H00289292 GATCTTCTCG GAGCCAGGAA GAAGGCCTTT CCTCCTCCTC GCCCTCCTCC

TCCCAACTGG GAAAAATATA GGCTCTTTCG TGCAGCA--- CAGCTGCAGC

TCCCAATTGG GAAAAATATA GGCTCTTTCG TGCAGCGCAG CAGCAGCAGC

TCCCAACTGG GAGAAGTACA GGCTCTTTCG TGCAGCCCAG CAGCAGAAGA

AACAACAACA ACAACAACAG CGATGTGAAG AGGAAGAAGA GAAGGAGCAA

AGCAGCAGCA GCAGCAGCAG CAGCAGGAAG AAGAGGAGGA GAAGGAGCAA

AGCAACAGGA GGAGGAGGAG GAGGAGGAAG AAGAAGAAGA AGAGGAAGAG

GAGGAGGAA- ---------- -GGAGAGAAA GAGGAGGACC TACCACCC

GAGGAGAAG- ---------- -GGAGAGAAA GAAGAGGACC TACCGCCC

GAAGAGGAGG AGGAGGAGGC AGAGGAGGAG GAAGAGGAGC TGCCACCC

>Ortholog Group 249, Repeat 1

3 171

H00290607 TCCCCTTTGC TTTGGAAGGA AAGGAGAGCG CTTGAAGAGC AATGTGACGA

M00117178 TGCCCTGTGC TCAGGAAGGA GAGGAGAGTG CTGGAAGAGC AGTGTGACAG

R00039716 TGTCCTGTGC TCAGGAAGGA GAGGAGAGTG CTGGAAGAGC AGAGTGACAA

GGACCATCAG ACACCGAGGG ATGGAGAGAC ATCCCACAGG GCCCAGATTA

GGACCAGCAG CCATCAAGAC ATAGCGAGAT CCCTTACAGA GCCCAGCCTT

GGACCAGCAG CCATTAAGAA ATGGCAAGAT TTCCTACAGA GCCCAGACTT

GCTACGTAGA GGATTTGAGG CATCAAATCC TAGCAGAAGA GATTCGAGCT

GCCATGTGGA GGCTCTGAAG CAGCAAGCCA AAGAAGGACA GAGTAGAGTC

GCCATGTGGA GGCTCTGAAG CAGCAAGCCA AAGAAGGACA GAGTAGAGTC

GCGAAGGAAC TGGAATTTGA C

CAGAAGGAGC TAGAACTGGA C

CAGAAGGAGC TAAAACTGGA C

>Ortholog Group 249, Repeat 2

3 174

H00290607 GCTTGGATTA GCCAGCAGAT TAAAGAAAAC CAGCAGTGTC TGCTCAGAGA

M00117178 ACACATATCA GCCAGCAGAT AAAAGACAAC CAGCAGTGGC TACTCACAGA

R00039716 ACACATATCA ACCGAAAGAT AACAGACAAC CAGCAGTGGC TACTCAGAGA

AGAGACCTGG CTGGCCAGCT TGGAAGACCA GGTAGCA--- GAGAAAGAAC

AGAGACCTGG CTAGCCAGCT TAGAAGGCAA CTGTGGAGAA GAGAAAGAAC

AGAGACCTGG CTATCCAACT TAGAAGGTGA CTGTGGAGAA GAGAAAGAGC

TTGAGGCATC TGTGGCACTT GATGCTTGGC TTCAGACAGA TCCTGAGATT

TTGAGGCCTC TGTGGCACCT GATGCTTGGC TTCCAACAGT TCCTCAGACT

TTGAGGCCTT TGTGGCACCT GATGCTTGGC TCCCAACAGT TCCTCAGACT

CAGCCATCCC CATTTGTCCA AAGT

CCACCATCCC CACTGGTCCA AAGC

CCACCATCTC CACTGGTCCA AAGC

>Ortholog Group 24, Repeat 1

3 198

R00055431 GGGAGGGAAG CTAGCCAAGC ATCTCCCTCT AGCTCCTGCG TTCTGCAGGT

M00032719 GGGCGGGAAG CTGGGCAAGC GTCTCCCTCC AGCTCCTGTG TTATGCAGGT

H00381007 GGAAGGGAAG CTGGCCAAGC TTCTCCTTCT GGTTCCTGTA CCATGACAGT

AGCACACAGC AGTGGACAGA GTCCTGGCAA TGGAGCTGTC CATCTTCCTC

CACACACAGC AGTGGACAGA GCCCTGGCAA TGGAGCTGTC CAGCTTCCTC

GGCACAAAGC AGTGGGCAGA GCACAGGAAA TGGTGCTGTC CAACTCCCTC

ATAGCCATCC TAACACTGCC ACGGTGGCTC CATTCATTTA CAGAGCACAT

ATAGCCATCC CAACACAGCC ACGGTGGCTC CGTTCATTTA TAGGGCACAT

ATAGCCACCC GAATACCGCG ACAGTGGCAC CATTCATTTA CAGGGCACAT

TCAGAAAATG AAGGCACCCT TTTACCACCT GCTGACTCCT GTACTAGC

TCAGAAAATG AAGGCACCTC TTTACCACCT GCTGACTCCT GTACCAGT

TCAGAAAATG AAGGTACCGC TTTACCATCG GCTGACTCCT GTACCAGT

>Ortholog Group 250, Repeat 1

3 198

M00069525 ---------- ---------- ---------- ---------- ----------

R00053312 ---------- ---------- ---------- ---------- ----------

H00357794 GAAGAGGAGC CGCAGCGGCA AAGAGAGCTC CAGGAGGAAG AAGAGCAGCT

---------- ---------- ---------- ---------- ----------

---------- ---------- ---------- ---------- ----------

ACGGAAGCTG GAGCGGCAAG AGCTGAGGAG GGAGCGCCAG GAGGAAGAGA

---------- ---------- ---------- ---------- ----------

---------- ---------- ---------- ---------- ----------

GGCTGAGGCG CGAGCAGCAA CTAAGGCGCA AGCAGGAGGA GGAGAGGCGC

---------- ---------- ---------- ---------- --------

---------- ---------- ---------- ---------- --------

GAGCAGCAGG AGGAGAGGCG CGAGCAGCAG GAGAGGCGCG AGCAGCAG

>Ortholog Group 251, Repeat 1

3 180

H00357737 ATGTCTTACT GCAAGCAGCC CTGCCAGCCA CCTCCTGTGT GCCCCACGCC

M00088386 ATGTCTTACT GCAATCAGCC GTGCCGGCCT CCTCCTGTGT GCCCACCCCC

R00053336 ATGTCTTACT GCAAGCAGCC CTGCCAACCT CCTCCTGTAT GCCTACCCCC

AAAGTGCCCA GAGCCATGTC CACCCCCGAA GTGCCCTGAG CCTTACCTGC

GAAGTGCCCT GAGCCTTGTC CTCCCCAAGT GTGGCCTGGG CCTTGTCGTC

GAAGTGTCCT GAGCCTTGTC CCCCTCCAGT GTGTCCTGAG CCTTGTCGCC

CTCCT----- -------CCT GAGCCATGTC CACCCCCGAA GTGCCCTGAG

CTGTCATGTG CTTTGAGCCT GAGCCTTGTC CTCCCCAAGT GTGGCCTGGG

CTCCAGTTTG CCCTGAGCCT GAGCCTTGTC CCCCTCCAGT GTGTCCTGAG

CCTTACCTGC CTCCT----- -------CCT

CCTTGTCGTC CTGTCATGTG CTTTGAGCCT

CCTTGTCGCC CTCCAGTTTG CCCTGAGCCT

>Ortholog Group 252, Repeat 1

3 198

R00021074 CTGGTAGAGA TGACCGGCTT GAGCCCGCGG GTCATCCGCG TGTGGTTTCA

H00290759 CTGGTGGAGA TGACCGGCCT GAGCCCGCGG GTCATCCGCG TCTGGTTCCA

M00034869 CTAGTAGAGA TGACCGGCTT GAGCCCGCGG GTCATCCGCG TGTGGTTTCA

GAACAAGCGT TGCAAGGACA AGAAGAAGTC CATTCTCATG AAGCAGCTAC

GAACAAGCGC TGCAAGGACA AGAAGAAATC CATTCTCATG AAGCAGCTGC

GAACAAGCGT TGCAAGGACA AGAAGAAGTC CATTCTCATG AAGCAGCTAC

ACAGCGACAA GGCGAGTCTC CAGGGACTAA CTGGGACGCC TCTGGTGGCG

ACAGCGACAA GACGAGCCTT CAGGGACTGA CTGGGACGCC CCTGGTGGCG

ACAGTGACAA GGCGAGCCTC CAGGGACTGA CTGGGACGCC TCTGGTGGCA

GGCAGCCCCA TCCGCCATGA GAACGCCGTG CAGGGCAGCG CAGTCGAG

GGCAGTCCCA TCCGCCATGA GAACGCCGTG CAGGGCAGCG CAGTGGAG

GGCAGCCCCA TCCGCCATGA GAACGCGGTG CAGGGCAGCG CAGTCGAG

>Ortholog Group 253, Repeat 1

3 198

M00057275 CACATAGCCC ACCTATTGGG GACTACAGGT GCCACTGCTA CTATGGGTAA

R00057938 CACATAGCCC ACCTATTGGG GACTACAGGT GCCACTACTA CTATGGGTAA

H00377942 CACATAGCCC ACTTCCTAGG GACTGGAGGT GCCGCTACTA CCATGGGTAA

TTCCTGCATC TGCCGAGATG ACAGTGGAGC AGAAGACAAT GTTGACACCG

TTCTTGCATC TGCCGAGATG ACAGTGGAGC AGAAGACAGT GTTGACACCG

TTCCTGTATC TGCCGAGATG ACAGTGGAAC AGATGACAGT GTTGACACCG

CTGAGAACAG TACAGTCCCT ACTGCTGATA GTAGGAGCCA ACCTCGGGAC

CTGAGAACAG TACAGTCCCT ACTGCGGACA GTAGGAGCCA ACTTCGGGAC

CCGAGAACAG TGCAGTACCC ACTGCTGACA CAAGGAGCCA ACCACGGGAC

CCTGTCCGGC CTCCAAGGAG AGGCCGAGGA CCTCATGAGC CAAGGAGA

CCTGTTCGGC CTCCAAGGAG AGGCCGAGGA CCTCATGAGC CAAGGAGA

CCTGTTCGGC CACCAAGGAG GGGCCGAGGA CCTCATGAGC CAAGGAGA

>Ortholog Group 254, Repeat 1

3 198

H00353109 AAAGAGTTGT GGACATGGAT GGAAGACCTT CAGAAGGAGA TGTTGGAGGA

M00074459 AAAGAGCTGT GGACATGGAT GGAGGACCTT CAGAAGGAGG TGCTGGAGGA

R00062745 AAAGAGCTGT GGACATGGAT GGAAGACCTT CAGAAGGAGG TGCTGGAGGA

TGTCTGTGCA GATTCTGTGG ATGCAGTCCA GGAACTGATC AAGCAGTTCA

TGTCTGTGCA GACTCTGTGG ATGCGGTCCA GGAGCTGATC AAGCAGTTCA

TGTCTGTGCA GACTCTGTGG ATGCGGTCCA GGAACTGATC AAGCAGTTCA

CCGCCACTCT AGATGCCACA CTCAATGTCA TCAAGGAAGG CGAAGACCTT

CCGCCACCCT GGATGCCACT CTCAATGTCA TCAAGGAAGG CGAAGACCTT

CCGCTACCCT GGACGCCACT CTCAACGTCA TCAAGGAAGG CGAAGACCTT

ATCCAGCAGC TCAGGTCAGC GCCTCCCTCC CTCGGGGAGC CCAGCGAG

ATCCAGCAGC TCAGGTCAGC GCCTCCCTCC CTGGGGGAGC CCACCGAG

ATCCAGCAGC TCAGGTCAGC GCCTCCCTCC CTGGGGGAGC CCACCGAG

>Ortholog Group 255, Repeat 1

3 201

R00010455 GTTGTGCTAA AAAGCACCAC CAAGATGTCT CTAAATGAGC GCTTTACTAA

H00357683 GTTGTGCTAA AAAGCACCAC CAAGATGTCT CTAAATGAGC GCTTTACTAA

M00061800 GTTGTGCTAA AAAGCACCAC CAAGATGTCT CTAAATGAGC GCTTTACTAA

TATGCTGAAG AACAAACAGC CGATGCCAGT GAATATTTGG GCTTCGATGC

TATGCTGAAG AACAAACAGC CGACGCCAGT GAATATTCGG GCTTCGATGC

TATGCTGAAG AACAAACAGC CGATGCCAGT GAATATTCGG GCTTCGATGC

TAGCCAGTGC CAGAAACAGA AGACTGGCCC AGCAGATGGA GAATAGACCC

TAGCCAGTGC CAGAAACAGA AGACTGGCCC AGCAGATGGA GAATAGACCC

TAGCCAGTGC CAGAAACAGA AGACTGGCCC AGCAGATGGA GAATAGACCC

TCTGTCCAGG CAGCATTAAA ACTTAAGCAG AAGAGCTTAA AGCAGCGCCT

TCTGTCCAGG CAGCATTAAA ACTTAAGCAG ---AGCTTAA AGCAGCGCCT

TCTGTCCAGG CAGCATTAAA ACTTAAGCAG AAGAGCTTAA AGCAGCGCCT

G

G

G

>Ortholog Group 256, Repeat 1

3 198

M00059210 TCGCCATACC TCAGCAGCCA GCAGCAGGCA GCTGTGATGA AGCAGCACCA

H00292599 TCCCCCTATC TCAGCAGCCA GCAACAGGCC GCTGTAATGA AGCAGCATCA

R00004361 TCT---TACC TCAGCAGCCA GCAGCAGGCA GCTGTGATGA AGCAGCACCA

GCTGCTCCTG GACCAGCAGA AGCAGAGGGA GCAGCAGCAG CAGCAGCTGT

GTTGCTTTTG GACCAACAGA AACAAAGGGA GCAGCAGCAA AAGCATTTAT

GCTGCTCCTG GACCAGCAGA AGCAGAGAGA G------CAG CAGCAGCTGT

TCCTGCAGAG G---CAGCAC CTCCTGGCTG AGCAGGAGAA ACAACAGTTT

TCCTTCAGAG GCAACAGCAC CTTCTCGCGG AACAGGAGAA GCAACAGTTT

TCCTGCAGAG G---CAGCAC CTCCTGGCTG AGCAGGAGAA GCAACAGTTT

CAGCGTCATC TGACCCGCCC ACCCCCCCAG TACCAAGACC CAACACAA

CAGCGCCATC TGACCCGCCC ACCACCCCAG TACCAAGACC CGACACAA

CAGCGTCATC TGACCCGCCC ACCCCCCCAG TACCAAGACC CAACACAA

>Ortholog Group 257, Repeat 1

3 132

R00057932 ---------- ---------- ---------- ---------- ----------

H00292672 ATGGCCCGCC TGACGGAGAG CGAGGCGCGC CGGCTCCTGC AGCCGCGGCC

M00113675 ---------- ---------- ---------- ---------- ----------

---------- ---------- ---------- ---------- ----------

CTCGCCCGTG GGCAGCAGCG GGCCCGAGCC CCCCGGGGGG CAGCCCGACG

---------- ---------- ---------- ---------- ----------

--ATGAAGGA CCTGGACGCC ATCAAGCTCT TC

GCATGAAGGA CCTGGACGCC ATCAAACTCT TC

--ATGAAGGA CCTGGACGCC ATCAAGCTCT TC

>Ortholog Group 257, Repeat 2

3 219

R00057932 CATCCTGCCT TCTCTGGAGT CCAGCAGTAC ACAGCCATGT ATCCCACCGC

H00292672 CATCCTGCCT TCTCCGGAGT CCAGCAGTAC ACAGCCATGT ACCCCACCGC

M00113675 CATCCTGCCT TCTCCGGAGT CCAGCAGTAC ACAGCCATGT ATCCCACCGC

GGCCATCGCG CCCGTGGCAC ACAGCGTTCC GCAGCCTCCG CACCTCCTGC

GGCCATCACG CCCATCGCGC ACAGCGTCCC CCAGCCGCCG CCCCTCCTGC

GGCCATCGCG CCCGTGGCAC ACAGCGTTCC GCAGCCTCCG CACCTCCTGC

GTGAAGGAGT TTGGAGACAC GGAGCTGACC CAGATGTTCC TCCCCTTCGG

GAGAAGGT-- ---------- ---------C CCGAGGGCTG TAACCTGTTT

GCGAAGGAGT TTGGAGACAC GGAGCTGACC CAGATGTTCC TCCCCTTCGG

CAATATCATC TCCTCCAA-- ---------- ---------- ----------

ATCTACCACC TCCCCCAGGA GTTTGGAGAC ACGGAGCTGA CGCAGATGTT

CAATATCATC TCCTCCAA-- ---------- ---------- ----------

---------- ---------

CCTACCCTTC GGCAATATC

---------- ---------

>Ortholog Group 258, Repeat 1

3 198

R00010628 GAGGTCTTTG TGGAGGCCTC AGCGGGCACA GAGGACATGG ACAGTCTGGA

M00030274 GAGGTCTTTG TGGAGGCCTC ACCGGGCACA GAGGACATGG ACAGTCTGGA

H00360899 GAGGTCTTCG TGGAGGCCTC GCCAGGCACA GAGGACATGG ACAGTCTAGA

GGATGCTGTC GTTCCCCGGG CTCTGTATGA GGAGCTGCTG CGAAACTACG

GGATGCTGTC GTGCCCCGGG CTCTGTATGA GGAGCTGCTG CGAAACTACG

AGATGCTGTG GTGCCCCGGG CTCTGTATGA GGAGCTGCTG CGCAACTACG

AAGAGATGCG CCACCTCCAG CAGGAGCTGG AGCGGACTCG GAGGCAGCTC

AGGAGATGCG CCACCTCCAG CAGGAGCTGG AGCGGACTCG GAGGCAGCTC

AAGAGATGCG CCACCTCCAG CAGGAGCTGG AGCGGACTCG GAGGCAGCTG

GTACAGCAAG CCAAGAAGCT CAAGGAATAC GGGGCATTGG TGTCTGAA

GTACAGCAGG CCAAGAAGCT CAAGGAGTAC GGGGCATTGG TGTCTGAA

GTACAACAGG CCAAGAAGCT CAAGGAGTAC GGGGCACTTG TGTCTGAA

>Ortholog Group 259, Repeat 1

3 198

R00006163 GTGAGGGAGA CTCTGGCTGC GGAGACAGGG CTGAGTGTCC GTGTGGTTCA

M00107008 GTGAGGGAGA CTCTGGCTGC GGAGACAGGG CTGAGTGTCC GTGTGGTTCA

H00340226 GTGAGAGAGA CTCTGGCTGC AGAGACAGGG CTGAGTGTCC GTGTCGTCCA

GGTGTGGTTC CAGAACCAGC GAGCCAAGAT GAAGAAGCTG GCCCGGCGAG

GGTGTGGTTC CAGAACCAGC GAGCCAAGAT GAAGAAGCTG GCCCGGCGAG

GGTGTGGTTC CAAAACCAGA GAGCGAAGAT GAAGAAGCTG GCCAGGCGAG

ATCAACAGAA CACCCAGAGG CTGAGTTCTG CTCAGACAAA TGGTAGTGGC

ACCAACAGAA CACCCAGAGG CTGACTTCTG CTCAGACAAA TGGTAGTGGG

ATCAGCAGAA CACCCAGAGG CTGAGCTCTG CTCAGACAAA CGGTGGTGGG

AATGCTGGCA TGGAAGGCAT CATGAACCCC TATACAACGT TGCCCACC

AATGCGGGCA TGGAAGGGAT CATGAACCCC TATACAACGT TGCCCACC

AGTGCTGGGA TGGAAGGAAT CATGAACCCC TACACGGCTC TGCCCACC

>Ortholog Group 25, Repeat 1

3 198

H00365624 TTGAGGACTA GCCTGGCTGC CCTGGAGCAG ATCCAAACAG CAAAGACCCA

M00111339 TTGAGGACTA GCTTGGCTGC TCTGGAGCAG ATCCAGACAG CAAAGACACA

R00050139 TTGAGGACTA GCTTGGCCGC TCTGGAGCAG ATCCAGACAG CAAAGACACA

AGAACTGAAT ATGCTCCGGG AACAGACCAC TGGGCTGGCA GCTGAGTTGG

AGAACTGAAT ATGCTTCGGG AACAGACCAG TGAATTGGCA TCTGAGTTAG

GGAACTGAAT ATGCTTCGGG AACAGAACAC TGAACTGGCA GCCGAGTTAG

CTGAGTACGA GGACCTTATG GGACAGAAAG ATGACCTCAA CTCCCAGCTC

CTGAATATGA AGAGCTTATG GGACAGAAAG ATGACCTCAA CTCGCAGCTC

CTGACTATGA AGAGCTTATG GGACAGAAAG ACGACCTCAA CTCCCAGCTC

CAGGAGTCAT TACGGGCCAA TAGTCGACTG CTGGAACAAC TTCAAGAA

CAGGAGTCGT TAAGGGCCAA TAGCCGGCTG CTGGAACAAC TTCAAGAA

CAGGAGTCGT TAAGGGCCAA TAGCCGGCTG CTGGAACAAC TACAAGAA

>Ortholog Group 25, Repeat 2

3 198

H00365624 CTCCAGCAGA CAGTGGAAGA ACTTCAAGCT CAGGTACATT CCATGGATGG

M00111339 CTTCAGCACA CAGTGGAAGA ACTCCAGGCT CAGGTACACT CCATGGATGG

R00050139 CTTCAGCACA CAGTGGAAGA ACTTCAGGCT CAGGTACACT CCATGGATGG

AGCCAAGGGC TGGTTTGAAC GGCGCTTGAA GGAAGCCGAG GAATCCCTGG

AGCCAAGGGC TGGTTTGAAC GGCGCTTGAA GGAAGCTGAG GAATCTTTGG

AGCCAAGGGC TGGTTTGAAC GGCGCCTGAA GGAAGCTGAG GAATCCTTAG

AACAAGAGGA AGCCCTCAAG CAGTGTCGGG AGCAGCACGC TGCCGAGCTG

AACAAGAGGA AACCCTCAAG CTGTGCCGGG AGGAGCACGC CGCTGAGCTG

AACAGGAGGA AACCCTCAAG CAGTGCCGGG AGCAGCACGC TGCCGAGCTG

AAGGGCAAGG AGGAGGAGCT ACAGGATGTA CGGGATCAGC TCGAGCAG

AAGGGCAAGG ATGAAGAGCT TCAGAATGTT CGGGAACAGC TCCAGCAG

AAGGGCAAGG AGGAAGAGCT TCAGAATGTT CGAGATCAGC TCCAGCAG

>Ortholog Group 260, Repeat 1

3 198

H00358064 TCAGATGATA AGCATTTTGG CCAGGCTCCC AGCAAGGGCA CTCCAAGTGA

M00088297 TCAGATGATA AGCATTTTGG CCAGACACCC AACAAGGGTA CTTCAAGTGA

R00033119 TCAGACGATA AGCATTTTGG CCAGACCCCC AACAAGGGTA CTTCAAGTGA

TGGTGTCAGT CTCTCAAACC TCACCCAACC CAGCTTGACC GCCACTGATG

TGGTGTCAGT CTCTCAAACC TCACCCAGCC CAGCTTGCCC ACCACTGACG

TGGTGTCAGT CTGTCAAACC TCACCCAGCC CAGCTTGCCT ACCACTGACG

AAGAGCACTA CCGCATAGAA ACCCGCGTCT CCTCCTCCTG CTTAGACTTG

AAGAACACTA TCGAATAGAA ACTCGTGTTT CCTCCTCCTG TTTAGACTTG

AAGAACACTA TCGAATAGAA ACTCGTGTTT CCTCCTCCTG TTTAGACTTG

CCTGATAGCA CAGAAGAAAA GGGGGCCCCT ATAGAAACCT TGGGTTAT

CCTGACAGCA CAGAAGAAAA GGGGGCCCCT ATAGAAACTC TAGGTTAT

CCTGACAGCA CAGAAGAAAA GGGGGCCCCC ATAGAAACTC TAGGTTAT

>Ortholog Group 261, Repeat 1

3 150

H00295367 ATGAGTTCTT ACCAGCAGAA GCAGACCTTT ACCCCACCAC CTCAGCTTGT

M00056287 ATGAGTTCTT ACCAGCAAAA GCAGCCCTTT GTCCCACCTC CTCAGCCTGT

R00037841 ATGAGTTCTT ACCAGCAAAA GCAGCCCTTC GTTGCACCTC CTCAGCCTGT

GAAACAACCC AGCCAGCCTC CACCTCAGGA AATATTTGTT CCC---ACAA

GAAGCAACCT TGTCAGCCTC CACCACAAGG AAAATTTGTT CCCATAGCCA

GAAGCAACCT TGTCAGCCTC CACCACAAGG AAAATTTGTT CCCATAGCCA

CCAAGGAGCC ATGCCACTCA AAGGTTCCAC AACCTGGAAA CACAAAGATT

CCTCTGAGCC ATGCCACACA GATGTTCCAC AACCAGGAAA CACCAAGATT

CCTCCGAACC ATGCCACACT GGTGTTCCAC AACCAGGAAA CACCAAGATT

>Ortholog Group 262, Repeat 1

3 174

M00095258 ATGAGGAAAC ACCGGCATCT GCCCCTAGTG GCTGTCTTTA GCCTTCTCCT

H00295550 ATGAGGAAAC ATCGGCACTT GCCCTTAGTG GCCGTCTTTT GCCTCTTTCT

R00026707 ATGAGGAAAC ACCGGCATCT GCCCCTAGTG GCTGTCTTTA GCCTTCTCCT

CTCAGGCATT GCCACGACTC ATGCC----- ---------- ----------

CTCAGGCTTT CCTACAACTC ATGCCGCAGA TGTCAAAAAT GGTGCGGCTG

CTCTGGCATC ACCATGACTC ATGCC----- ---------- ----------

---------- ---------- ---------- ---------- ----------

CTGATATAAT ATTTCTAGTG GATTCCTCTT GGACCATTGG AGAGGAACAT

---------- ---------- ---------- ---------- ----------

---------- ---------- ----

TTCCAACTTG TTCGAGAGTT TCTA

---------- ---------- ----

>Ortholog Group 263, Repeat 1

3 198

H00357130 GCAGCTGGGG CTCTTCTAAA GAAGCATGAG GCCTTTCTAT TAGATCTCAA

R00049978 GCAGCTGGGG CCCTTCTCAA AAAGCATGAG GCCTTCCTGG TGGACCTCAA

M00027817 GCAGCTGGGG CCCTTCTCAA GAAGCATGAA GCCTTCCTGG TGGATCTGAA

TTCATTTGGA GACAGTATGA AAGCTCTGCG GAATCAGGCA AACGCCTGCG

CGCATTTGGA AACAGCATAA AGGCTCTGGG GGATCAGGCA GAGGCCTGCG

TGCATTTGAA AACAGCATAA AGGCTCTAAG AGATCAGGCA GAAGTCTGCG

CTGCACCAGT GGAGGGAGTT GCTGGAGAAC AAAGGGTCAT GGCTTTATAT

CAGCACCAGT GGATGAGGCT GGTCGAGAAG CTAGGGTCAT AGCTCTGTAT

CAGCACCAGT GGACGAGGCT GGTCGAGAAG CTAGGGTCAT AGCTTTGTAT

GACTTCCAGG CCCGCAGCCC CCGAGAAGTC ACCATGAAGA AAGGTGAT

GACTTTGAGG CTCGCAGCCG TAGAGAGGTC AGCATGAAGA AGAATGAT

GACTTTGAGG CTCGCAGCCG TAGAGAGGTC AGCATGAAGA AGAATGAT

>Ortholog Group 264, Repeat 1

3 186

M00022257 ATGTCGGAGC GGGCCGCGGA TGACGTCAGG GGGGAGCCGC GCCGC-----

R00010103 ATGTCGGAGC GGGCCGCGGA TGACGTCAGG GGGGAGCCGC GCCGC-----

H00381590 ATGTCGGAGC GGGCCGCGGA TGACGTCAGG GGGGAGCCGC GCCGCGCGGC

----GCGGCG GGCGGAGCAG CGGCCGCC-- -------CCA CAGCCTCTGC

----GCGGCG GGCGGAGCAG CGGCCGCC-- -------CCA CAGCCTCTGC

GGCGGCGGCG GGCGGAGCAG CGGCCGCGGC CGCCCGGCCG CCGCCTCCGC

AGCCCCAGCG GCAGCAC--- ------CCGC CGCTGCGGCG CCCACGGGCG

AGCTCCAGCG GCAGCAC--- ------CCGC CGCCGCGGCG CCCACGGCCG

AGCCCCAGCG GCAGCAGCAC CCGCCACCGC CGCCACGGCG CACACGGCCG

GAGGACGGCG GCACCGGGGA CACCACCACC TCGGCC

GAGGACGGCG GCACCGGGGC CGCCACTACC TCGGCC

GAGGACGGCG GGCCCGGCGC CGCCTCCACC TCGGCC

>Ortholog Group 265, Repeat 1

3 198

M00107727 GTGGGGACAG AATGGAGAAA CCTTGAAACA GCCAAGAAAG CAGAATATGA

H00296302 GTGGGGACAG AATGGAGAAA TCTTGAGACA GCCAAGAAAG CAGAATATGA

R00016581 GTGGGGACAG AATGGAGAAA CCTTGAAACA GCCAAGAAAG CAGAATATGA

AGAGCGGGCA GCTAAAGTTG CTGAGCAGCA GGAGAGAGAG CGAGCAGCAC

AGAGCGGGCA GCTAAAGTTG CTGAGCAGCA GGAGAGAGAG CGAGCAGCAC

AGAGCGGGCA GCTAAAGCTG CTGAGCAGCA GGAGAGAGAG CGAGCAGCAC

CGAGTGCTTC TCCCCGAGCA GGCACCCCTG TGGGGGCTCT CATGGGGGTG

CGAGTGCTTC TCCCCGAGCA GGCACCCCTG TGGGGGCTCT CATGGGGGTG

CGAGTGCTTC TCCCCGAGCA GGCACCCCTG TGGGGGCTCT CATGGGGGTG

GTGCCACCAC CAACACCAAT GGGGATGCTC AATCAGCAGT TGACACCT

GTGCCACCAC CAACACCAAT GGGGATGCTC AATCAGCAGT TGACACCT

GTGCCACCAC CAACACCAAT GGGGATGCTC AATCAGCAGT TGACACCT

>Ortholog Group 266, Repeat 1

3 198

M00038405 GCCATGAACC ACGGGCGCTT CCCCGACGGC ACCAACGGGC TGCACCACCA

H00356623 GCCATGAACC ACGGGCGCTT CCCCGACGGC ACCAATGGGC TGCACCATCA

R00016244 GCCATGAACC ACGGTCGCTT CCCCGACGGC ACCAACGGGC TGCACCACCA

CCCTGCCCAC CGCATGGGCA TGGGGCAGTT CCCGAGCCCG CATCATCACC

CCCTGCCCAC CGCATGGGCA TGGGGCAGTT CCCGAGCCCC CATCACCACC

CCCTGCCCAC CGCATGGGCA TGGGGCAGTT CCCGAGCCCG CATCACCACC

CCCAGCACGC CTTCAACGCC CTCATGGGCG AGCACATACA CTACGGCGCG

CCCAGCACGC CTTCAACGCC CTAATGGGCG AGCACATACA CTACGGCGCG

CCCAGCACGC CTTCAACGCC CTCATGGGCG AGCACATACA CTACGGCGCG

GGCAACATGA ATGCCACGAG CGGCATCAGG CACGCCATGG GGCCGGGG

GGCAACATGA ATGCCACGAG CGGCATCAGG CATGCGATGG GGCCGGGG

GGCAACATGA ATGCCACGAG CGGCATCAGG CACGCCATGG GGCCGGGG

>Ortholog Group 267, Repeat 1

3 165

H00321826 ATGAGGAAAT TCAACATCAG GAAGGTGCTG GACGGCCTGA CCGCCGGCTC

R00049322 ATGAGGAAAT TCAACATCAG GAAGGTGCTG GACGGCCTGA CCGCAGGCTC

M00101195 ATGAGGAAAT TCAACATCAG GAAGGTGCTG GACGGCCTGA CCGCAGGCTC

GTCCTCGGCG TCG---CATC CGCCTGGGAA CCGGGAGCCG GAGATCCAGG

GTCCTCGGCC TCGCAACACC CGCCTGGGAA CCGGGAGCCC GAGATCCAGG

GTCCTCGGCC TCGCAACACC CGCCTGGGAA CCGGGAGCCG GAGATCCAGG

AAACGCTCCA GTCCGAGCAC TTTCAGCTCT GCAAGACTGT TCGCCATGGA

AGACGCTCCA GTCCGAGCAC TTCCAACTCT GCAAGACTGT TCGCCATGGA

AGACGCTCCA GTCCGAACAC TTCCAACTCT GCAAGACTGT TCGCCATGGA

TTTCCCTATC AACCC

TTTCCCTATC AGCCC

TTCCCCTATC AGCCC

>Ortholog Group 268, Repeat 1

3 204

M00039806 AGGAGCAGAC TGTCGTTGCC TCCTATGCCT GGTCAAAAAA ACAGTTCACC

H00410944 GGAAGTAGAC TCTCCTTCCC CCCCGTGCCT GGTCAAAAAA ACAGTTCACC

R00031005 AAGAGCAGGC TGTCGTTGCC TCCTATGCCG GGTCCAAAAA CTGGTTCACC

CACCAACTTT TCCAAACTTA TCAGCAATGG CTACAAGGAC GAGTGGTTAA

TACCAATTTT TCCAAACTCA TTAGCAATGG TTATAAGGAT GAGTGGTTGC

CACAAACTTT TCCAAACTTA TCAGCAATGG CTACAAGGAC GAGTGGTTAA

AGGCTGAAGC TGACAGGAGG ACTCCAAAGA CCTCCAGGGC ATCCGTGTCC

GAGCTGACTC AGACAAGAGG ACCCCGAAGA CCTCCAGGGC ATCAGTGTTA

AGGCTGATTC TGACAGGAGG ACTCCAAAGA CATCCGAGGC ATCCGTGTCC

TCCAAGTCCA CAGAGGAC-- ----TCTAAA AGCAACCAGG ACACAGAAAC

TCTCAGTCCC CACGAGAC-- ----CTGGAA GGTCCCCAGG ATGCAGCCAG

ACCCAGTCCA CAGAGGACTC TAAAAGTAAA AGTAGCCAAG ATACAGAGAC

GCCC

GCTC

GCCT

>Ortholog Group 269, Repeat 1

3 210

H00297338 GTACCAGAAG ACCTTAGAAA AAGGAGGAAA GGAGGAGAGG CAGATAATTT

R00006209 GTACCAGAAG ACCTTAGGAA AAGAAGGAAA GGGGGAGAGG CAGATAATCT

M00022927 GTACCAGAAG ACCTTAGGAA GAGAAGGAAA GGGGGAGAGG CAGATAATCT

GGATGAATTC CTCAAAGAAT TTGAAAATCC AGAGGTTCCT AGAGAGGACC

GGATGAGTTC CTCAAAGAGT TCGAGAACCC AGAGGTCCCC CGAGAGGAGC

GGATGAGTTC CTCAAAGAGT TTGAGAATCC AGAGGTTCCC AGAGAGGAGC

ATCAG----- -------CAG CGTGATGTTA TCGATGAGCC CATTATTGAA

CACAGCAGCA GCAGGCGCAG CGTGACGTCA TTGATGAGCC CATTATAGAA

AGCAGCAGCC ACAGCCGCAG CGAGATGTCA TCGATGAGCC CATTATAGAA

GAGCCAAGCC GCCTCCAGGA GTCAGTGATG GAGGCCAGCA GAACAAACAT

GAGCCAAGCC GCCTTCAGGA CTCAGTGATG GAGGCCAGCA GAACAAACAT

GAGCCAAGCC GCCTCCAGGA CTCAGTGATG GAGGCCAGCA GAACAACCAT

AGATGAGTCA

AGAAGAGTCG

AGAAGAATCA

>Ortholog Group 26, Repeat 1

3 198

M00034766 TGCCTGGCCG TGGGGATGTC TCGAGATGCT GTCAAGTTTG GTCGGATGTC

H00261523 TGCCTTGCCG TAGGGATGTC TCGAGATGCT GTAAAATTTG GCCGAATGTC

R00054314 TGCCTGGCTG TGGGGATGTC TCGAGATGCT GTCAAGTTTG GCCGGATGTC

CAAGAAGCAG AGAGACAGCT TGTACGCCGA GGTGCAGAAG CACCGGATGC

AAAAAAGCAG AGAGACAGCT TGTATGCAGA AGTACAGAAA CACCGGATGC

CAAGAAGCAA AGAGACAGCC TGTACGCCGA GGTGCAGAAG CACCGGATGC

GAGACCACCA GCAGCAGCCT GGGGAGGCGG AGCCGCTGAC GCCCACCTAC

GCGACCACCA GCAGCAGCCT GGAGAGGCTG AGCCGCTGAC GCCCACCTAC

GAGACCACCA GCAGCAACCT GGGGAGGCTG AGCCGCTGAC GCCCACCTAC

AACATCTCAG CCAATGGGCT GACGGAACTG CATGATGACC TCAGCACC

AACATCTCGG CCAACGGGCT GACGGAACTT CACGACGACC TCAGTAAC

AACATCTCAG CCAATGGGCT GACGGAACTG CATGATGACC TCAGCACC

>Ortholog Group 270, Repeat 1

3 198

H00427820 CCAATTCCAA GAAGCAAAAA TGACCAATGG GAAAGTGAAG ATAGTGGCTC

R00060947 CCGGTCCCGC GGAGTAAGAA TGACTCATGG GAAAGTGAAG ACAGCAGCTC

M00117421 CCGGTTCCGC GGAGTAAGAA TGACTCATGG GAAAGTGAAG ACAGCAGCTC

TAGTCCTGCA GGAAGCTTAA AGATGGAGCC TAAGAACAAA GGATTAAAAC

TAGTCCGGCG GGAAGCTTGA AGATGGAGCC CAAGACGAAG GGACTAAAGC

CAGTCCAGCA GGAAGCTTAA AGATGGAGCC CAAGACGAAA GGATTAAAAC

ATAAGAAACT CCTGGCCGCA ATGCTCTCTC AAGATTCTTT TGAGTCCATC

ATAAGAAACT CCTGGCTGCA ATGCTCTCCC AGGATTCCTT CGAGTCCATC

ACAAGAAACT CCTGGCTGCA ATGCTTTCCC AGGACTCCTT CGAGTCCATC

CACAGCCCTA CCCCATCTGT AACAGAAGAA GATATTGATA ATGAAGAT

CACAGCCCCA CGCCATCTGT AATAGAAGAA GACATTGACA ATGAAGAC

CATAGCCCCA CACCATCTGT AATAGAAGAA GACATTGATA ATGAAGAC

>Ortholog Group 271, Repeat 1

3 201

R00005873 ACTTTGTGGG AGAATAAACT AATGCAGTCT AGGGCAGTAG ATGGATTTCA

M00021345 ACTTTGTGGG AGAATAAACT AATGCAGTCT AGGGCAGTAG ATGGATTTCA

H00298173 ACTTTATGGG AAAACAAACT AATGCAGTCC AGGGCAGTAG ATGGATTTCA

TTCAGAAGAA CAGCAGCTTT TGTTGCAAGT TCAGCAGCAG CACCAGCCCC

TTCAGAAGAA CAGCAGCTTT TGTTGCAAGT TCAGCAGCAG CATCAACCCC

TTCAGAAGAG CAGCAGCTTC TACTGCAAGT TCAACAGCAG CATCAACCCC

ATCACCACCA CCACCAC--- CACCAGCAGG CGCAACCTCA GCAAACGGTA

ATCACCACCA CCACCACCAG CACCAGCAAG CGCAACCTCA GCAAACGGTA

ATCACCACCA TCACCAT--- CATCAGCAAG CTCAGCCTCA GCAGACAGTA

CCTCAGCAAG CACAGACCCA GCAGGTTCTT ATTCCTGCGT CACAGCAAGC

CCTCAGCAAG CACAGACCCA GCAGGTCCTC ATTCCTGCAT CCCAGCAAGC

CCTCAGCAAG CGCAGACCCA GCAGGTTCTT ATTCCTGCAT CACAGCAAGC

T

T

C

>Ortholog Group 272, Repeat 1

3 198

H00370408 GCCGAGCTAG CCGCCACGCT GGGGCTCTCT GAGAGGCAGG TTAAAATCTG

M00031650 AGTGAGCTGG CTGCCACACT TGGGCTCTCC GAGAGGCAGG TTAAAATTTG

R00045045 GCTGAGTTGG CCGCCACACT CGGGCTCTCG GAGAGGCAGG TTAAAATTTG

GTTTCAGAAC CGCAGAGCAA AGGAGAGGAA AATCAACAAG AAGAAGTTGC

GTTTCAGAAC CGCAGAGCCA AGGAGAGGAA AATC---AAG AAGAAGCAGC

GTTTCAGAAC CGCAGAGCCA AGGAAAGGAA AATCAACAAA AAGAAGTTGC

CACCACAGCC GCCTCCGCCG CCACCACAGC CTCCCCAGCC TCAGCCAGGT

AACAACAGCC TCCACAGCCG CCGCCACAAC CTTCCCAGCC TCAGCCGGGT

CT------CC CGCACCGCCG CCACCACAGC CTTCCCAGCC TCAGTCCGGT

CCTCTGAGAA GTGTCCCAGA GCCCTTGAGT CCGGTGTCTT CCCTGCAA

GCCCTGCGGA GCGTGCCCGA GCCCTTGAGT CCTGTGACCT CCTTGCAA

GCATTGCGGA GCGTGCCGGA GCCCTTGAGT CCTGTGACCT CCTTGCAA

>Ortholog Group 273, Repeat 1

3 153

H00361209 ATGAATGAGG TGAAAGAATC CCTTCGCAGC ATCGAGCAGA AGTACAAGCT

R00017004 ATGAATGAGG TGAAAGAGTC CCTCCGCAGC ATCGAGCAGA AGTACAAACT

M00099941 ATGAATGAGG TGAAAGAGTC CCTCCGCAGC ATCGAGCAGA AGTATAAGCT

CTTCCTCACC TTCACCGCCG CTCTGGAGCA CTGCAGGGAG AACGCCCACG

CTTTTTCACC TTCATTGCTG CTCTGGAGCA CTGCCGGGAG AATGCCCACG

CTTCTTCACC TTCATTGCGG CTCTGGAGCA CTGCCGGGAG AATGCCCACG

ACAAGATCCG GCCCATCTCC AGCATTGGAC AGGTGCAGAG CTACATGGAA

ACAAAATCCG GCCCATCTCC AGTATCGAGC AGGTGCAGAG CTACATGGAA

ACAAAATCCG GCCCATCTCC AGTATCGAGC AGGTGCAGAG CTACATGGAA

CAC

CAT

CAC

>Ortholog Group 274, Repeat 1

3 237

M00107063 GCCTTAGGTG GACTCCTGGG CAACGTGTTT CTGCACCTGC TGCCGGAGGC

R00016191 GCCTTAGGTG GACTCCTGGG CAATGTGTTT CTGCACTTGC TGCCAGAGGC

H00354689 GCCCTGGGGG GACTCTTGGG CAATGTGTTT CTGCATCTGC TGCCCGAAGC

GTGGGCCTAC ACCTGTAACA TCACCCCCGC CACTGGCTTG GCATGGCCTG

GTGGGCCTAC ACCTGTAACA TCAGCCCC-- ---------- ----------

CTGGGCCTAC ACGTGCAGCG CCAGCCCT-- ---------- ----------

TTAGTGCTCC CCCTGCAGGT GGTGAAGGGC AGAGTCTGCT GGGGCTATGG

---------- -------GGT GTTGAAGGGC AGAGTCTGCT GGGGCTGTGG

---------- -------GGT GGTGAGGGGC AGAGCCTGCT GGGGCTGTGG

GTCATCGCTG GCTTCCTGAC CTTCCTGGCG TTGGAGAAGA TGTTCCTCAA

GTCATCGCTG GCTTCCTGAC CTTCCTGGCA TTGGAGAAGA TGTTCCTCAA

GTCATTGCTG GCATCCTGAC CTTCCTGGCG TTGGAGAAGA TGTTCCTGGA

CAGCAAG--- GAGGACCCCA GCCAGGCCCC CAGCAAA

CTGCAAGGAG GAGGACCCTA GCCAGGCCCC CAGCAAA

CAGCAAGGAG GAGGGGACCA GCCAGGCCCC CAACAAA

>Ortholog Group 275, Repeat 1

3 198

H00320968 GTGATGGAGA AACAGGTCCG GTCCACGCAG CCTCAGCAGA ACAACGAGGA

R00011955 GTGATGGAGA AGCAGGTCCG GTCCACACAG CCTCAACAGA ACAACGAGGA

M00013220 GTGATGGAGA AGCAGGTCCG GTCCACACAG CCTCAGCAGA ACAACGAGGA

ACTGCCCACT TACGAGGAGG CCAAAGCACA GTCGCAGTTC TTCAGGGGGG

GCTGCCCACG TACGAGGAGG CCAAGGCCCA GTCCCAGTTC TTCAGGGGAG

GCTCCCCACG TATGAGGAAG CCAAGGCCCA GTCCCAGTTC TTCAGGGGAG

GGGCGGTGGG CCATGGTTAC TACATGGCAG GGGGCACCAG TCAGAAGTCC

GGCCCCTCGG CCACACTTAC TACATGGCCG GAGGTACCAG TCAGAAGTCC

GGCCCCTTAG CCACACTTAT TACATGGCCG GAGGTACCAG TCAGAAGTCC

CGAACTGAGG GGAGGCCCAC TGTGAACCGT GCCAACAGTG GACAGGCG

CGCACCGAGG GGAGGCCCAC CGTGAACCGG GCCAACAGCG GACAAGCA

CGCACTGAGG GGAGGCCCAC AGTGAACCGG GCCAACAGTG GACAGGCG

>Ortholog Group 276, Repeat 1

3 198

R00038767 ACCCTGCATG ATGCCAGCTC TAGCACGCAC AGCAGAGCCC TAGTCGATAA

H00345216 GCCCTGCATG GTGCCAGCTG TAGCACGCAC AGCAGAGCCC TAGTCGATAA

M00022356 ACCCTGCATG ATGCCAGCTC TAGCACGCAC AGCAGAGCCC TAGTCGATAA

GAAGTTGCAA ATCAGTATTC GAAAAGCTCG AAGCCTGCAA GATCGCATGC

GAAGTTGCAA ATCAGTATTC GAAAAGCACG GAGCCTGCAG GATCGCATGT

GAAGTTGCAA ATCAGTATTC GAAAAGCACG AAGCCTTCAG GATCGCATGT

CATCGCAGCA GCCGGTGCAG CCCTCAGCCT CTCTCCCATC ACAGGGGGGC

CACCACAGCA GCCGTCGCAG CCCTCAGCCT GCCTCCCAAC ACAGGCGGGG

CATCGCAGCA GCCGGTGCAG CCCTCAGCCT CTCTCCCATC ACAGGGGGGC

GGCCTCCCTC AGCCGACCAG TGAACAGTCT GTCCCGCTCC AAGTACTG

ACTCTCTCTC AGCCAACAAG TGAACAGCCT ATCCCGCTCC AAGTATTG

GCCCTCCCTC AGCCGACAAG TGAACAGCCT ATCACGCTCC AAGTATTG

>Ortholog Group 277, Repeat 1

3 207

M00103235 ---------- ---------- ---------- ---------- ----------

H00379396 GCTAAGGGAA TAAACATCCA GGGGCTGTCT GCAGAAGAGA TCAGGAATGG

R00051474 GCTAAGGGGA TCAACACCCA GGGGCTGTCT GCAGAAGAGA TCAGGAATGG

---------- ---------- ---------- ---------- ----------

AAACCTCAAG GCCATTCTAG GCCTCTTCTT CAGCCTCTCC CGATACAAG-

GAACCTCAAG GCCATTCTAG GCCTCTTCTT CAGCCTCTCC CGATACAAGC

---------- ---------- ---------- ---------- ----------

-----CCCCA GAAGCAGCAC CTC---TCCT CACCTCTGCC GCCCGCCGTA

AGCAACCTGA GAAGCAGCAC CTCTCATCAT CCCCTCTGCC ACCTGCCGGG

---------- ---------- ---------- ---------- ----------

TCCCAGGTGG CCGGGGCCCC CTCCCAGTGC CAGGCTGGCA CCCCTCAGCA

TCTCAGGTGG CCGGGGCGCC TTCCCAGTGC CAGTCTGGCA TTCTTCAGCA

-------

GCAGGTG

ACAGGGG

>Ortholog Group 278, Repeat 1

3 174

H00371462 CTGGAGGGCA AGGCCCAGTG GGGGGAGAAT CTTCAGGTGA CTCTGATCCC

M00092223 CTGGAAGGCA AGGCCCAATG GGGGGAGAAC CTTCAGGTGA CTCTGATTCC

R00019215 CTGGAAGGCA AGGCCCAGTG GGGGGAGAAC CTTCAGGTGA CTCTGATTCC

TACTCATGAC ACGGAGGTGA CTCGTGAGTG GTACCAACAA ACTCATGAGC

CACTCATGAT ACGGAGGTGA CTCGTGAGTG GTATCAGCAG ACACACGAGC

CACTCATGAC ACGGAGGTGA CTCGTGAGTG GTATCAGCAG ACACATGAAC

TGAATGTCCT GGTCCTGGCT AGCAGCAGCA CCGTGGTGAT GCAGGATGAG

TGAACGTCCT GGTCCTGGCT AGCAGCAGCA CTGTGGTGAT GCAGGACGAG

TGAATGTCCT GGTCCTGGCG AGCAGCAGCA CTGTGGTGAT GCAGGACGAG

TCCTTCCCTG CCTGCAAGAT TGAG

TCCTTTCCAG CCTGCAAGAT TGAG

TCCTTTCCAG CCTGCAAGAT TGAG

>Ortholog Group 279, Repeat 1

3 102

R00044890 CCAGCCACAC CTTCTTCCAT GGACATGAAC AGCAGACTAC TGTAT-----

H00301067 CCAGCCACGC CTTCCTCTAT GGATGTGAAC AGCCGGCAAC TGGTAGGAGG

M00023741 CCAGCCACAC CTTCTTCCAT GGACATGAAC AGCAGGCAAC TTGTCGGAGG

---------- ---------- -----ATTTT TCCAGGCAAC TTG---TTGA

CTCCCAAGCT TTCTATCAGC GAGCACCCTA TCCTGGGTCC CTGCCCTTAC

CTCCCAGGCC TTCTACCAGC GAACACCCTA TCCTGGGTCC CTGCCCTTAC

TC

TG

AA

>Ortholog Group 279, Repeat 18

3 18

R00044890 ---------- --------

H00301067 ATGGGCCTTT TAAACCAG

M00023741 ATGGGCCTCT TGAACCAG

>Ortholog Group 279, Repeat 19

3 117

R00044890 ---------- ---------- ---------- ---------- ----------

H00301067 CGAACTTTAC TGTCTCCTGT GGCACTTGGC CCTGGCATGC CAGCAAAGCC

M00023741 CGAACTTTAT TATCTCCGGT GACACTTGGC CCTGGCCTGC CAGTCAAGCC

---------- ---------- ---------- ---------- ----------

TCTTCAACAC TTTTCTAGCC CTGGAGCCCT GGGTCCAACC CTCCTCCTGA

TCTTCAACAC TTTTCTAGCT CTGGAGCCCT AGGCCCAACC CTCCTCCTGA

-----AAGGA ACAAAAC

CGGGCAAGGA ACAAAAC

CAGGCAAGGA ACAAAAC

>Ortholog Group 279, Repeat 2

3 102

R00044890 AGCCTGGGTC CCTGCCCTTC CATGAGACTT GCCATGTCTG CTCGATTCCC

H00301067 TGGGCAACAG CAGCAACCTC CATGCGATTT GCCATGTCAG CTCGCTTTCC

M00023741 CAACAACCAG CAGCAACCTC CATGAGACTT GCCATGTCTG CTCGGTTCCC

ATCAACTCCT GGACCTGAAC TTGGCCGCCA AGCACTAGGT TCCCCCTTGG

ATCAACTCCT GGACCTGAAC TTGGCCGCCA AGCCCTAGGT TCCCCGTTGG

GTCAACTCCT GGACCTGAAC TTGGCCGCCA AGCACTAGGT TCCCCCTTGG

CA

CG

CA

>Ortholog Group 279, Repeat 20

3 201

R00044890 CCCTCCATTT CTGGAGACTC CCAGCTCTTG CTTGTC---- --------CA

H00301067 CCCTCACTCT CTGGGGACTC ACAACTCCTG CTTGTCCAAC CCCAGCCCCA

M00023741 CCTTCCATTT CTGGAGACTC ACAGCTCCTC CTTGTCCAAT CCCAGGCCCA

GTCTCAGCCC ACCTCAGTGC AGTTGCAGCC ACCACTAAGG CTCCCAGGA-

GCCTCAGCCC AGCTCTCTGC AGCTGCAGCC ACCTCTGAGG CTTCCAGGAG

ATCTCAGGCC ACCTCAGTGC AGCTGCAGCC CCCGCTCAGG CTCCCAGGAG

---------- ---------- ---------- ---------- ----------

TTAGCCTGCT CCACACAGCA GGTGGAGGAA GCCATGGG-- -CAGCTAGGC

TGAACTTGCT TCACACAGCA GGTGGAGGAA GCCATGGGCA GCAGCTAGGG

---------- ---------- ---------- ---------- ----------

AGTGGATCAT CTTCTGAGGC CTCATCTGTG CCCCACCTGC TGGCTCAGCC

AGTGGATCCT CCTCTGAGTC CCCAGCTGTA CCCCACCTGC TGGCCCAACC

-

C

C

>Ortholog Group 279, Repeat 21

3 198

R00044890 ---------- ---------- ---------- ---------- ----------

H00301067 GGTCTGCCTG GGGTTGGAAT CATGCCTACG GTGGGTCAGC TTCGAGCACA

M00023741 GGTCCTCCTG GAGCTGGAGT CATGCCTACA GTGGGTCAAC TTCGAGCACA

---------- ---------- ---------- ---------- ----------

GCTCCAAGGA GTCCTGGCCA AAAACCCACA GCTGCGGCAC TTAAGTCCTC

GCTCCAAGGA GTCCTGGCCA AAAACCCACA GCTGCGGCAC CTGAGCCCAC

---------- ---------- ---------- ---------- ----------

TACAGGCACT CCTCATGCAG CGGCAGCTGC AGCAGAGTCA GGCAGTACGC

TGCAGGCACT CCTCATGCAG CGGCAGCTAC AGCAGAGTCA GGCAGTACGC

---------- ---------- ---------- ---------- --------

CAGACCCCAC CCTACCAGGA GCCTGGGACC CAGACCTCTC CCCTCCAG

CAGACTCCAC CGTTCCAGGA GCCCGGGACC CAGCCCTCTC CCCTCCAG

>Ortholog Group 279, Repeat 3

3 222

R00044890 ---------- ---------- ---------- ---------- ----------

H00301067 GTAGCCAGCG AGTTACCCCT GCTCATTGAG GACCTGTTGG AGCATGAGAA

M00023741 GTAGCTAGCG AGTTGCCCCT TCTCATTGAG GACCTACTAG AGCACGAGAA

---------- ---------- ---------- ---------- ----------

GAAGGAGCTG CAGAAGAAGC AGCAGCTTTC AGCACAGTTG ----------

GAAGGAGCTA CAGAAGAAGC AACAGCTTTC AGCACAGACT GTGCTGCCTG

---------- ---------- ------AATC TCCTTCCCGT ACCAGGCCCT

---------- ----CAGCCT GCCCATTCCC TACTGTCTGC ACCAGGCCCT

CCCAGCAGCA GCAGCAGCAG CAGCATACCC TCCTTCCCAC ACCAGGCCCT

GCCCAGGCCC TGCCCTTGCC ACATGAGTCT GGGCCACCA- ----------

GCCCAGGCCA TGTCTTTGCC ACATGAGGGC TCTTCTCCCA GTTTGGCTGG

GCCCAGGCAC TGCCTTTGCC ACATGAGCCT GGGCCACCA- ----------

-------CAG CAGCTTGCTC TG

GTCCCAACAG CAGCTTTCCC TG

-------CAG CAGCTTGCTC TA

>Ortholog Group 279, Repeat 4

3 201

R00044890 CTGGTCACAG AGCAGCAGAG CAAGATCCAG AAGCAGCTGG ACCAGGTCCG

H00301067 CTGGTTACAG AGCAGCAGAG CAAGATCCAG AAACAACTGG ATCAGGTCCG

M00023741 CTGGTCACTG AGCAGCAGAG CAAGATCCAG AAGCAGCTGG ATCAGGTCCG

AAAACAGCAG AAAGAGCATA CAAATCTCAT GGCAGAATAT CGGAGC----

GAAACAGCAG AAGGAGCACA CTAATCTCAT GGCAGAATAT CGGAACAAG-

GAAACAGCAG AAAGAGCACA CAAATCTCAT GGCAGAATAT CGGAACAAGC

---------- ---------- ---------- ---------- ----------

--CACTCAGC TGTGCTGGCT CTCAGCCCTT CCCAGAGTCC CCGGCTGCTC

AGCACTCAGC TGTGCTGGCT GTCAGCCCTT CTCAGAATCC TCGGGTACTC

---------- ---------- ---------- ---------- ----------

ACCAAGCTCC CTGGTCAGCT GCTCCCTGGC CATGGGCTGC AGCCACCACA

ACCAAGCTCC CTGGCCAGCT GCTCCCTGCC CATGGGTTAC AGCCACCGCA

-

G

G

>Ortholog Group 279, Repeat 5

3 174

R00044890 ---------- ---------- ---------- ---------- ----------

H00301067 GGGCCTCCGG GTGGGCAAGC CGGAGGTCTT CGCCTGACCC CTGGGGGTAT

M00023741 GCACCTCCGG GTGGGCAAGC TGGTGGTCTT CGTCTGCCTC CAGGGGGTAT

---------- ---------- ---------- ---------- ----------

GGCACTACCT GGACAGCCTG GTGGCCCCTT CCTTAATACA GCTCTGGCCC

GGTACTACCT GGACAGTCTG GTGGTCCATT CCTCAATACT ACCTTGGCCC

---------- ---------- ---------- ---------- ----------

ATTCTGGTGG GGCTGGATCC CTGGCTGGCC CTTCAGGGGG CTTCTTCCCT

ATTCTGGTGT GGCTGGATCC TTGACTGGCC CCCCTGGGAG CTTCTTCCCC

---------- ---------- ----

GGCAACCTTG CTCTTCGAAG CCTC

GGCAATCTTG CTCTCCGAAG CCTT

>Ortholog Group 279, Repeat 6

3 96

R00044890 ---------- ---------- ---------- ---------- ----------

H00301067 GGACCTGATT CAAGGCTTTT ACAGGAAAGG CAGCTGCAGC TGCAGCAGCA

M00023741 GGACCTGACT CGAGGCTTTT ACAGGAAAGG CAACTACAGC TGCAGCAGCA

---------- ---------- ---------- ---------- ------

ACGTATGCAG CTGGCCCAGA AACTG----- ----CACCTT CTAGGA

ACGAATGCAG CTGGCTCAGA AACTACAGCA GCAGCACCTC TTAGGG

>Ortholog Group 279, Repeat 7

3 111

R00044890 ---------- ---------- ---------- ---------- ----------

H00301067 CAGGTGGCAA TCGGTCCTGG AGTACAGACA AACCAAGCTC TGGGTCCCAA

M00023741 CAGGTGGCAA TCGGTCCCGG AGTACAG--- AACCAGGCTC TGGGTCCTAA

---------- ---------- ---------- ---------- ----------

GCCCCAGGGC CTTATGCCTC CCAGCAGCCA CCAAGGCCTC CTGGTCCAGC

GCCTCAGGGC CTTCTGCCTC CCAGCAACCA CCAGGGCCTC CTGGTCCAGC

---------- -

AGCTGTCCCC T

AGTTGTCCCC C

>Ortholog Group 279, Repeat 8

3 153

R00044890 ---------- ---------- ---------- ---------- ----------

H00301067 CCTCACAGAC AGGTGCTTAT GACCCAGTCC CGGGTGCTCA GTTCCCCCCA

M00023741 CCTCACAGAC AGGTGCTTAT GACTCAGTCC AGGGTGCTGA GCTCCCCTCA

---------- ---------- ---------- ---------- ----------

GCTGGCACAG CAGGGTCAGG GCCTTATGGG ACACAGGCTG GTCACAGCCC

GCTGGCACAG CAGGGTCACA GCCTTATGGG ACACCGGCTA CTCACAGCCC

---------- ---------- ---------- ---------- ----------

ACCAACAGCA AGGGTCCATG GCAGGGCTGT CCCATCTTCA GCAGAGTCTG

AACAACAACA GGGATCTATG ACAGGGCTTT CCCAACTTCA GCAAGGAATG

---

ATG

ATG

>Ortholog Group 279, Repeat 9

3 54

R00044890 ---------- ---------- ---------- ---------- ----------

H00301067 CACAGTGGGC AGCCCAAACT GAGCGCTCAG CCCATGGGCT CTTTACAGCA

M00023741 CATGGTGGAC AGCCCAAAAT GAGTGCTCAG GCCTTGGGTT CTTTACAG--

----

GCTT

----

>Ortholog Group 27, Repeat 1

3 198

H00424827 CCTAAAACCA GTGGCTCAGA AGATGATAAT GCAGAACAGG CTGAGGAATT

R00009747 CCTAAA---A ACGGCTCAGA TGATGAAAAT GCAGACCAGG CTGAGGAGTT

M00034740 CCTAAA---A ATGGCTCAGA AGATGAAAAT GCAGACCAGG CTGAGGAGTT

AGAGCCTGGC TGGGTTGTTT TGGACCAACC AGATGCTGCT TGCCATTTGG

AGAGCCTGGC TGGGTTGTTT TGGACCAACC AGATGCTGCC ACCCATTTGG

AGAGCCTGGC TGGGTTGTTT TGGACCAACC AGATGCTGCC ACTCATTTGG

AACCTTCTCC TCTACCTCCA GGGTGGGAAG AGAGGCAGGA TATCCTTGGA

AACCTTCTCC CCTACCTCCA GGGTGGGAAG AGAGGCAGGA TGTCCTTGGA

AACCCTCTCC CCTACCTCCA GGATGGGAAG AGAGGCAGGA TGTCCTTGGA

AGGACCTATT ATGTAAACCA TGAATCTAGA AGAACACAGT GGAAAAGA

AGGACCTACT ACGTAAACCA TGAGTCTAGA ACAACACAAT GGAAAAGA

AGGACCTACT ACGTAAACCA TGAATCTAGA AGAACACAGT GGAAAAGG

>Ortholog Group 280, Repeat 1

3 198

H00252985 TGGGAGTGTC TGCTGGCAGA GATGAAAGTG AGCAGGGGTG AGCGCAGCCA

M00103811 ---------- ---------- ---------- ---------- ----------

R00037384 ---------- ---------- ---------- ---------- ----------

CTGCCCAACG CAAACCGTGA AGAAGCTTCT GGAAGAGCAG AGGCGCCGCC

---------- ---------- ---------- ---------- ----------

---------- ---------- ---------- ---------- ----------

CCGACGCTGG CGGGGTGCAG GGACAATTTC TCCCTCCCCC AGAGCAGCCC

---------- ---------- ---------- ---------- ----------

---------- ---------- ---------- ---------- ----------

CTGACCCCAT CTGTGAATGA GGCTGTGACT GGCCACCCTC CCTTCCCA

---------- ---------- ---------- ---------- --------

---------- ---------- ---------- ---------- --------

>Ortholog Group 281, Repeat 1

3 204

H00388053 ATCAGCAAGT CAGGAGCTGC CGGCGGCTCT GCCAAGTCCA GCAGCAATGG

R00055499 ---------- ---------- ---------- ---------- ----------

M00112428 ATCAGCAAAG CAGGGGCTAC C---AGCAGT TCCAAAGCCA GCAGCAGTGG

GCCTGTGGCC AGTGCACAGT ACGTGTCCCA GGCAGAAGCC TCAGCTTTG-

---------- ---------- ---------- ---------- ----------

TCCTGTGGCC AGTGCACAAT ACGTCTCTCA GGCAGAAGCC TCTGCTTTGC

-----TACTA CCAGTGGTAC CAGCAGTACA ACTATGCCTA CCCCTACAGC

---------- ---------- ---------- ---------- ----------

AGCAGTACTA CCAGTGGTAC CAGCAGTACA ACTATGCCTA TCCGTATAGC

TACTACTATC CCATGAGCAT GTACCAGAGC TATGGCTCCC CTTCCCAGTA

---------- ---------- ---------- ---------- ----------

TACTACTACC CCATGAGCAT GTACCAGAGC TATGGCTCCC CCTCCCAGTA

TGGG

----

TGGG

>Ortholog Group 282, Repeat 1

3 198

H00304689 ACCTACACGG TACGCGTCCC CACCATCTTC CCGCTGCGCG GCGTCAATGA

M00048994 ACCTACACGG TGCGCGTTCC CACCATTTTC CCGCTGCGTG GCGTCAATGA

R00025815 ACCTACACGG TGCGCGTTCC CACCATTTTC CCGCTGCGTG GCGTCAATGA

GCGCAAAGTA GCGCGCAGAC CCGCTGGGGC CGCGGCCGCC CGCCGCAGGT

GCGCAAAGTA GCTCGGAGAC CTGCGGGAGC TGCGGCAGCC CGCCGTAGG-

GCGCAAAGTA GCTCGGAGAC CTGCGGGAGC TGCGGCAGCC CGCCGTAGG-

CCTCACCCTC TGCCTCCACT GCCCAGACTG CCCAGCTGCA GCCGAACCTG

--TCTCCGTC CTCCTCCACT GCCCAGACCA CCCAGTTGCA GCCGAACCTG

--TCTCCGTC CTCCTCCACT GCCCAGACTG CCCAGTTGCA GCCGAATCTG

GTATCTGCTT CCGCGGCCGT GCTTCTCACC CTTCAGGCCA CTGTAGAC

GTGTCTGCCT CTGCAGCTGT GCTTCTTACG CTTCAGGCCG CCGTAGAC

GTGTCTGCCT CTGCGGCCGT GCTTCTTACT CTTCAGGCCG CCGTAGAC

>Ortholog Group 283, Repeat 1

3 198

M00075100 ATCCTGTGCA CCTTCTTCAT TAGCCTGCGA TCCTCTGACC ACCGTCAGGT

R00060952 ATCCTGTGCA CCTTCTTCAT TAGCCTGAGA TCCTCTGACC ACCGTCAGGT

H00362814 CTCCTGTGCA CCCTCTTCAT CAGTCTGCGC TCCTCAGACC ACCGGCAGGT

GAACAGCCTG ATGCAGACAG AGGAGTGTCC GGCAGAGATG ---------G

GAACAGCTTG ATGCAGACAG AGGAGTGTCC AGCAGAAATG ---------G

GAACAGCCTG ATGCAGACCG AGGAGTGCCC ACCTATGCTA GACGCCACAG

TGGCCGTCAG TGATGGCAGA GCCTATGACA ATGAGCAGGA TGGTGTCACC

TGGCCGTCGG TGACGGCCGA ACCTATGACA ATGAGCAGGA CGGTGTCACC

TGGCAGCCTG TGAGGGCCGG GCCTTTGACA ACGAGCAGGA CGGCGTCACC

TACAGCTACT CCTTCTTCCA CTTCTGCCTG GTGCTGGCCT CCCTGCAT

TACAGCTATT CCTTCTTCCA CTTCTGCCTG GTGCTGGCCT CCCTGCAT

TACAGCTACT CCTTCTTCCA CTTCTGCCTG GTGCTGGCCT CACTGCAC

>Ortholog Group 284, Repeat 1

3 111

R00018689 ATGTTAGGG- --CTGTACTC GTCGGCCGCG CTCCTGACCG GAGAGCGGAG

M00079226 ATGTTAGGGC AGCTGTACTC GTCGGCCGCG CTCCTGACCG GAGAGCGGAG

H00307183 ATGTTAGGG- --CTGTACTC GTCGGCCGCG CTCCTGACCG GGGAGCGGAG

CCGGCTGCTC ACCTGCTACG TGCAGGACTA CCTGGAGTGT GTGGAGTCGC

CCGGCTGCTC TCCTGCTACG TGCAGGACTA CCTGGAGTGT GTGGAGTCGC

CCGGCTGCTC ACCTGCTACG TGCAGGACTA CCTTGAGTGC GTGGAGTCGC

TGCCCCACGA C

TGCCCCACGA C

TGCCCCACGA C

>Ortholog Group 285, Repeat 1

3 204

H00425443 GAGACTGAGC CATTTTCAAA CTCACACCTT TTGCAACATA AGCCTCATAA

R00048956 GAGGCTGAAC TATTTTCCAG C------TTT TTACAACATA CCCCTCATAA

M00043977 GAGACTCAAC GGTTATCAGG C------TTT TTACAACATA CTCCTCAGAC

ACAGGCAGCA CAAACACAAC CATCCCAGAG TTCACATCTC CCTCAA----

GCAGGCAGCA CAAACACAAG CATCCCAGAA CTCAAATTTC CCTCAAGTCT

GCAGGCATCA CAAACACCAG CATCCCAGAA CTCAAATTTC CCTCAAATCT

--AACAAATT ACAAATAAAG AATAAAGAGG AAATACTCCA GACTTTTCCT

GC---CAGTT ACAGAGAAAG AATAAAGAGC AGATGCCTCA GACTTTCTCT

GCCAGCAGTT ACAGAGGAAG AATAAAGAGC AAATGCCTCA GACTTTCTCT

CACCCCCAAA GCAACAATGA TCAGCAAAGA GAAGGATCAT TCTTTGGCCA

CACCTCCACG GTACCAACCA TGAGCAAAGT CAGGGCCAGT GTTTTGGCAC

CATCTCCAAG GTAGCAATGA TAAGCAAAGA GAAGGCTCGT GCTTTGGCCA

GACT

GATT

GATT

>Ortholog Group 285, Repeat 2

3 102

H00425443 AGCCAGGCTA AACAGTTGGC AGAACTTTTG CGACTTTCAG GACCAGTCAT

R00048956 AGCCCTATGA TACAGATGAC AGCACAACTT CGGCTTTCGG GATCTGTCAT

M00043977 AGCCATATGA AACAAATGAC AGCACAACCG CAGCTTTCGG GCCCGGTCAT

GCAGCAGTCC CAGCAGCCCC AGCCTCTACA GAAGCAGCCA CCACAGCCCA

C--------- CAGCAGCCAC AGTCACTCCA GAGG------ ---CACCTTA

C--------- CGGCAGCCAC CAACACTCCA GAGG------ ---CACCTTA

GA

GG

GG

>Ortholog Group 285, Repeat 3

3 156

H00425443 CCCCCA---- ---------- ---------- ---------- --CATCACCC

R00048956 CCACCA---- ---------- ---------- ---------- --CCTCAGCC

M00043977 CCACCACCTC AGCCGCAGCC GCAGACGACA CCTCAGCCAC AGCCACAGCC

TCAG------ ---------- --ACAGAGTC TGTCAACTCT TATTCTGCTT

ACAGCACATC TTGCCCAGTA ACTCTCAACC TGTTGGTTCT CATTCT---T

ACAGCATATC ATGCCCGGTA ACTCTCAGTC TGTTGGTTCT CATTGT---T

CTGGATCCAC CAATCCATAC ATGAGACGGC CCAATCCAGT TAGTCCTTAT

CTGGATCCAC CAATGTATAC ATGAGACAGC CTGCTCCACT TAGTCCTTAC

CTGGATCCAC CAGTGTCTAC ACGAGACAGC CTACTCCTCA CAGTCCTTAT

CCAAAC

CCCAGC

CCCAGC

>Ortholog Group 286, Repeat 1

3 210

R00019579 AAGGTAGACA CAGCTGCACA GACCAACAGC CGCCTGGCTC GACCCACAAG

H00304895 AAGGTGGACA CAGCTGCTCA GACCAATAGC CGCCTGGCCC GGCCCACGAG

M00063795 AAGGTAGACA CAGCTGCACA GACCAACAGC CGCCTGGCTC GACCCACAAG

GCTGTCCTTG GGGGATCCCA AGGCAAGCAC TTTACCCCGG GTACGAGAGC

GCTGTCCCTG GGGGATCCCA AGGCCAGCAC CTTACCTCGG GCCCGAGAG-

GCTGTCCTTG GGGGATCCCA AGGCAAGCAC CTTACCCCGG GTTCGAGAG-

AACAGCAGCA GTCTTCCCTG CACCCTCCCG AGCCCAAAAG CCCAGGAGAA

---------- -CCCTTGCTG CACCCTCCAG AGCCCAAGAG CCCGGGGGAA

---------- -TCTTCCCTG CACCCTCCCG AGCCCAAAAG CCCAGGAGAA

TATGTGAATA TTGAATTCGG GAGTGGCCAG CCAGGCTATT TAGCTGGCCC

TATGTCAATA TTGAATTTGG GAGTGATCAG TCTGGCTACT TGTCTGGCCC

TATGTGAATA TTGAATTCGG GAGCGGCCAG CCTGGCTATT TAGCTGGCCC

TGCAACTTCC

GGTGGCTTTC

TGCAACTTCC

>Ortholog Group 287, Repeat 1

3 105

R00009129 AATCTGTTCC AGAGCGTGCG CGAAGCGATC CAGAACCCGG GCCCCAGGCA

H00363822 AATCTGTTCC AGAGCGTGCG CGAAGTGATC CAGAACCCGG GCCCCAGGCA

M00052648 AATCTGTTCC AGAGCGTGCG CGAAGCGATC CAGAACCCGG GCCCCAGGCA

CCCTGAGGCC GCTAGCATAG CACCTCCCGG TGCCTGTTTA ---------G

CCCAGAGGCC GCGAGCGCAG CACCTCCCGG CGCCAGTTTG CTGCTGCTGG

CCCTGAGGCC GCTAACATAG CACCTCCCGG CGCCTGTTTA ---------G

AGACT

AGACT

AGACT

>Ortholog Group 287, Repeat 2

3 108

R00009129 CCCCGGCGGC CTGAGGATGG CTCTCCTCAA GCCCACATCA GAGGCACCAC

H00363822 CCC---AGGG GTGAGGATGG TTCTCCCCAA GCCCATCGTA GAGGCCCCAC

M00052648 CCCCGGCGGA CTGAGGATGG TTCTCCTCAA GCCCACATCA GAGGCCCCAC

AGGCTACCTG GCCCTGGAGG AGGAACAGCA GCCTTCACAG CAGCAGTCAG

AGGCTACCTG GTCCTGGATG AGGAACAGCA ACCTTCACAG CCGCAGTCGG

AGGCTACCTG GCCCTGGAGG AGGAACAGCA GCCTTCACAG CAGCAGGCAG

CCTCCGAG

CCCTGGAG

CCTCCGAG

>Ortholog Group 287, Repeat 3

3 249

R00009129 TCCACGTTGT CCCTACTGGG CCCCACTTTC CCAGGCTTAA GCAGCTGCTC

H00363822 TCCACGTTGT CCCTGCTGGG CCCCACTTTC CCCGGCTTAA GCAGCTGCTC

M00052648 TCCACGTTGT CCCTGCTGGG CCCCACTTTC CCAGGCTTAA GCAGCTGCTC

CGCAGACATT AAAGACATCC TGAGCGAGGC CGGCACCATG CAACTTCTTC

CGCTGACCTT AAAGACATCC TGAGCGAGGC CAGCACCATG CAACTCCTT-

CGCCGACATT AAAGACATTT TGAACGAGGC CGGCACCATG CAACTTCTT-

AGCAGCAGCA GCAACAGCAA CAGCAGCAGC AGCAGCAGCA GCAGCAGCAG

---------- ---------- ---------- ---------- ----------

-----CAGCA GCAGCAACAA CAGCAGCAGC ACCAACAGCA GCACCAACAG

GAGGTAATAT CCGAAGGCAG CAGCAGCGTG AGAGCAAGGG AGGCCACTGG

GAAGCAGTAT CCGAAGGCAG CAGCAGCGGG AGAGCGAGGG AGGCCTCGGG

GAGGTAATCT CCGAAGGCAG CAGCGCA--- AGAGCCAGGG AGGCCACGGG

GGCTCCCTCT TCCTCCAAGG ATAGTTACCT AGGGGGCAAT TCGACCATA

GGCTCCCACT TCCTCCAAGG ACAATTACTT AGGGGGCACT TCGACCATT

GGCTCCCTCT TCCTCCAAGG ATAGTTACCT AGGGGGCAAT TCAACCATA

>Ortholog Group 288, Repeat 1

3 99

M00092034 GTTCCCGGGG GCGCTTTCCC GGGTGCTTCT GCCATGCCTA GAGCCTCAGG

H00304956 GTTCCTGGGG GCGCTTTCCC GGGCGCCTCG GCCATGCCCA GAGCTGCGGG

R00029870 GTTCCCGGGG GCGCTTTCCC GGGTGCTTCA GCCATGCCCA GAGCCTCAGG

CATGGTGGGT TTGTCCAAAA TGCACAGCCA GCCACCC--- ---------

CATGGTGGGC TTGTCCAAAA TGCACGCCCA GCCACCGCAG CAGCAGCCC

CATGGTGGGT TTGTCCAAAA TGCACGGTCA GCCACCG--- ---------

>Ortholog Group 288, Repeat 2

3 99

M00092034 CACGGAGTGT TCTTCGAGAG GTTTGGCGGG GCCCGAAAGA TGCCTGTGGG

H00304956 CATGGTGTGT TCTTTGAGAG GTTCAGTGGG GCCAGAAAGA TGCCTGTGGG

R00029870 CACGGAGTGT TCTTCGAGAG GTTTGGCGGG GCCCGGAAGA TGCCTGTGGG

TCTGGAGCCT GCAGTGGGCT CCAGGCACCC GCTAATGCAG CCTCCCCAG

TCTGGAGCCC TCAGTGGGCT CCAGGCACCC GTTAATGCAG CCTCCCCAG

TCTGGAGCCT GCAGTGGGCT CCAGGCACCC GCTAATGCAG CCTCCCCAG

>Ortholog Group 288, Repeat 3

3 204

M00092034 GCCTACCCAG GCCTCCCAGG CGAGTTCACA CCGCCTGTGC CGGACAGCTT

H00304956 GCCTACCCAG GCCTACCCGG CGAGTTCACA CCGCCTGTGC CCGACAGCTT

R00029870 GCCTACCCAG GCCTCCCAGG CGAGTTCACG CCGCCTGTGC CGGACAGTTT

TTCCTCGGGA CCACCCTTGC AGCACCCGGG GCCGGACCAC CAGTCCCTG-

CCCTTCGGGG CCGCCCCTGC AGCATCCGGC CCCGGACCAC CAGTCCCTG-

TCCTTCAGGA CCGCCCTTGC AGCACCCGGG GCCGGACCAC CAGTCCCTGC

-----CGCCA GAATGCGGCC CTCATGATTA AACAGATGGC GTCCCGGAAC

-----CGCCA AAACGCGGCC CTCATGATTA AGCAGATGGC GTCGCGGAAT

AGCAGCGCCA GAACGCGGCC CTCATGATTA AACAGATGGC GTCCCGGAAC

CAGCAGCAGC GGTTGCGACA GCCCAACCTG GCCCAGCTAG GCCATCCTGG

CAGCAGCAGC GGCTGCGCCA GCCCAACCTG GCTCAGCTAG GCCACCCCGG

CAGCACCAAC GGTTGCGACA GCCCAATCTG GCCCAGCTAG GCCACCCTGG

GGAC

GGAC

GGAC

>Ortholog Group 289, Repeat 1

3 132

R00012811 AGGAAGAAGG CGAGCCAGGG CCGGAGGGAG AATGCCAATA GGCACCCGAC

M00030687 AGGAGGAAGG GGGGCCAGGG CCGGAGGGAG AATGCCAACA GGCATCCGGC

H00383846 AGGAGGAAGG GAGGCCAGGG CCGGCGGGAG AATGCCAACA GGAACCTGGC

CAGGAAGAAC AGCAAGGAGC CAGGCTCCAA CTCTCGGAGA CACAAAGGCG

CAGGAAGAAC AGCAAGGAGC CGGGCTCCAA CTCTCGGAGA CACAAAGGGG

CAGGAAGGAG AGCAAGGAGG CGGGTGCTGG CTCTCGAAGA CGCAAGGGGG

GGACAGCAGG GCCACTCACA TCAGTAGGCC CC

GGACAACAGG GCCACTCACA TCAGTAGGAC CT

GGACAGTGGG GCCACTCACA TCTGCAGGGC CT

>Ortholog Group 28, Repeat 1

3 156

H00384177 ATGAGCATAG AGACGCTACT GGAGGCGGCC CGCTTCCTGG AATGGCAAGC

M00000291 ATGAGCATAG AGACGCTACT GGAGGCGGCC CGCTTCCTGG AATGGCAAGC

R00003933 ATGAGCATAG AGACGCTACT GGAGGCGGCC CGCTTCCTGG AATGGCAAGC

GAGAGCACGT GAGGAGCAGG AGCGGCTTCG CTTG------ GAGCAGGAGC

GAGAGCACGT GAGGAGCAGG AGCGCCTTCG CCTGGAGCGG GAACGTGAGA

GAGAGCACGT GAGGAGCAGG AGCGCCTTCG TCTGGAGCGG GAACGTGAGA

GAGAGCAGGA ACAGAAGAAG GCCAATAGCC TGGCCAGGCT GGCACATACC

GAGAGCAGGA GCAGAAGAGG GCAAGTAACT TGGCCAGGTT GGCCCATGCC

GAGAGCAGGA GCAGAAGAGG GCAAGTAACC TGGCAAGGTT GGCCCATGCC

CTTCCT

CTGCCT

CTGCCT

>Ortholog Group 28, Repeat 2

3 201

H00384177 TCCACCCTGC CACCCCCCAG CACCACCCCT GCGCCTCTGC CTCCACACCC

M00000291 TCCGCCCTGC CAACCCCCAG CACCGCCCCT GCGCCTCTGC CTACCCATCC

R00003933 GCCGCCCTGC CAACCCCCAG CACCGCCCCT GCGCCTCTGC CTGCCCATCC

ACACCCTCAC CCCCACTCCG TGGCCCTACC TCCTGCCCAC CTCCCCGTG-

GCACCCTCAC CCCCACCCAG TGGCCCTGTC TCCTGCCCAC CTTCCTGTGC

GCACCCTCAC CCCCACCCAG TGGCCCTGTC TCCTGCCCAC CTCCCTGTG-

--CCACAGCA GAAGACCCCT CTGCCAGCCC CTCCTCCCCC ACCGGCTGCC

AGCCACAGCA AAAGACCCCT CTGCCAGCCC CTCCTCCCCC ACCTGCCACC

--CCACAGCA AAAGACCCCT CTGCCAGCCC CTCCTCCCCC ACCTGCCACC

CCTGCCCAGA CACTGGTGCC AGCTCCAGCC CATCTGGTGG CGACGGCTGG

CCAACCCAGA CGCTAGTGCC AGCTCCGGCC CATCTGGTGG CCACGGCTGG

CCAACCCAGA CGCTAGTGCC AGCTCCAGCC CATCTGGTGG CCACGGCTGG

G

G

G

>Ortholog Group 290, Repeat 1

3 102

R00042047 GTTGAGACAG CCAAACAGGC CAAAGAGAAA GCAAAGGAGA CCGCACTGGC

H00302114 GTTGAGACAG CCAAGGAAGC CAAGGAGAAG GCAAAGGAGA CGGCACTGGC

M00021942 GTTGAGACAG CCAAAGAGGC CAAAGAGAAA GCAAAGGAGA CCGCACTGGC

AGCTACAGAG AAGGCCAAGG ACATGGCCAA CAAGGCCGCC ACCAAGCCC-

AGCTACAGAG AAGGCCAAGG ACCTCGCCAG CAAGGCGGCC ACCAAGAAGT

AGCTACAGAG AAGGCCAAGG ACCTGGCCAA CAAGGCCGCC ACCAAGCAGC

--

TT

TC

>Ortholog Group 291, Repeat 1

3 258

M00058575 GGAGGAACTG GGTCTGCGCA GAGTCCGTCC TTTGCTCCGT GGAGCTACTG

R00062369 ---------- ---------- ---------- ---------- ----------

H00371940 TCAGGGGAGC GGGTACAGGG GCTGCGCAGA GTTTGTCCC- ----------

CCACCTGCTG CAACATCCAC CGTGCCTGCT GCACCCCCAG ---GCCGCTT

---------- ---------- ---------- ---------- ----------

---------- ---------- ---------- -CGACCGCGT GGAGCTACTG

CCCCTTTGTT TCACATGTCC GCAGGTTCCC GGACAATATT GCAACCGCCG

---------- ---------- ---------- ---------- ----------

CCCCCTGCTC CAGCGACCGT CAAGCCTGTC GGACAATTTT G---------

CTGCCGCTGC AGCAAGAACA GCATCACTGT GCCATTAGCT GCGCTCCCGG

---------- ---------- ---------- ---------- ----------

---------C GGCATCAGGA GGAGGACTGC GCCAACGGCT GCGCTCCCAC

TAAAGGCGTG CGGTTAGGCC AGCACCTGTC CGCCGTTGCG GCGCTGCTGA

---------- ---------- ---------- ---------- ----------

CAAGGGCGCG GTGCTGGGCG GGCACCTGTC GGCCGCGTCG GCGCTGCTGC

GGCAACAG

--------

GGTATCAG

>Ortholog Group 292, Repeat 1

3 198

M00038761 CAATTTAGCT CTTTCCTCAG CCAATACCCA GAGATGCAGT TGCAGCCACT

H00305976 CAATACAGCC CTTTCCTCAG CCAGTACCAA GAGATGCAGC TTCAGCCCCT

R00062018 CGATTTAGCT CTTTCCTCAG CCAATACCCA GATATGCAGT TGCAGCCCCT

GCCCTCTACT CCCGGCCCCC AGGCTCCACC TCCCTTACCC TCACAATTGC

GCCCTCCACT TCCGGTCCCC GGGCTGCTCC TCCTCTGCCC ACGCAGCTAC

GCCCTCTACG CCCAGCCCCC GGGCTCCACC TCCCTTACCC TCACAACTGG

CACCACCCCC ACCACCACCC CCTCCACCAC AGCAGCCAGG AGCTGCTCCA

CGCCACCGCC ACCACCCCCT CCACCACCAC GACAGCCAGG AGCTGCCCCA

CACCACCCCC ACCCCCACCC CCTCCACCAC AGCAGCCAGG AGCTACTCCA

ACCTCCTTAC AGTTCTCCTA TCAGACTTGT GAGCTGCCAA GCACCACT

GCCCCCTTAC AGTTCTCCTA TCAGACTTGT GAGCTGCCAA GCGCTGCT

GCCTCCTTAC AGTTCTCCTA TCAGACTTGT GGGCTGCCAA GCACCACT

>Ortholog Group 293, Repeat 1

3 201

R00022817 GACGATGACT GTGAAAGAGC CAAGGGACCT GCAGGAAGTT TAAAGTCTGT

H00382382 GATGATGACT GTGAAAGAGT CAAAGGACCT GTAGGAAGCC TAAAGTCTGT

M00081757 GACGATGATT GTGAAAGAGT CAAAGGACCT GCAGGGAATT TAAAGTCTGT

GGATGCCATT CTAGAAGAGA GCACTGAAAA ACTCAAAAGC CTGTCACTG-

GGAAGCTATT CTAGAAGAAA GCACTGAAAA ACTCAAAAGC TTGTCACTG-

GGATGCTATT CTAGAAGAGA GCACTGAAAA ACTGAAAAGC TTGTCACTGC

--GATGGGGA CAACGGAGAC AGCAGCAAAA GCACTGAGAC AAGTGACTTT

--GATGGAGA TAATGGGGAC AGCAGCAAAA GTACTGAGAC AAGTGACTTT

AGGATGGGGA CAACGGAGAC AGCAGCAAAA GCACTGAGAC AAGTGACTTT

GAGAATATTG AATCCCCTCT CAATGAGAGG GGCTCTTCCA CCTCTGTGGA

GAAAACATCG AATCACCTCT CAATGAGAGG GACTCTTCAG CATCAGTGGA

GAAAATATTG AATCCCCTCT CAATGAGAGG GGCTCTTCCA CCTCTGTGGA

T

T

T

>Ortholog Group 294, Repeat 1

3 198

H00398214 TCCAGCCTGA TGAGTGAAGA GGCTAAGCGA GGAGCACCCA ACCCTTGGCT

R00031413 TCCAGCCTGA TGGGTGAAGA AGCAAAGCGA GGATCTTCCA ACCCTTGGCT

M00021285 TCCAGCCTGA TGAGCGAAGA AGCAAAGCGA GGAACTCCCA ACCCTTGGCT

CTTTGAGGAG CCAGAGGAGA CCAGAGGCTT GGGTTTTGAT GAAATCCGGA

CTGTGAGGAG CCAGAGGAGA CCAGAGGCTT GGGTTTTGAT GAGGTCCGGA

CTGTGAGGAG CCGGAGGAGA CCAGAGGTTT GGGTTTTGAT GAGATCCGAA

AAATTATCCA AGAACAGGAC GCAGGCCTTG ATGCCCTTTC CTCTATCATA

AAATCATAAA AGAACAGGAC GCGGGTCTTG ATGCCCTTTC CTCTACCATA

AAATTATTCA AGAACAGGAC GCGGGTCTTG ATGCCCTTTC CTCTATCATA

AGTCGCCAAA AACAAATGGG GCAGGAAATT GGGAATGAAT TGGATGAA

AGCCGCCAAA AGCAAATGTG CCAGGAGATT GGGAATGAAC TGGACGAA

AGTCGCCAAA AACAAATGGG CCAGGAAATT GGGAACGAAC TGGATGAA

>Ortholog Group 295, Repeat 1

3 234

H00351686 GTATCTGGGC AGCCGTTGAG TCCCTCAGCC CAGCAGGCTC AGCAGGGGCT

R00010423 GTAGCAGGGC AGCCATTGAG CCCCTCTGCT CAGCCGGTCC AGCAGGGGCT

M00074104 GTAGCTGGGC AGCCATTGAG CCCCTCTGCT CAGCAGGTAC AGCAGGGGCT

CAGCCCCTCC CACATCCAGG GCAGTTCTTC CACACAGGGG CAGGCTCTGA

CAGCCCCTCC CATATCCAAG GCAGTTCTTC CACTCAAGGG CAGGCTCTAA

CAGCCCCTCC CATATCCAAG GCAGTTCTTC CACACAAGGG CAGGCTCTCA

ATTCCTCTGT GCAGCACACG TACCTGCCCA GTGCTTGGAA TTCCTTCCGT

ATTCCTCTGT ACAACACACA TACCTCCCCA ATGCTTGGAA CTCCTTCCGT

ATTCCTCTGT ACAACACACA TACCTCCCCA ATGCTTGGAA CTCCTTCCGT

GGCTATTCA- ---------- ---------- ---------- -----TCTGA

GGTTATTCTG CTGTGTCTGC TGAGGAGACC AGCCAGCCTT CATCGTCAGA

GGTTACTCTG CTGTGTCTGC TGGAGATACC AGCCATGAGT CCGCATCTGA

GATTCAAATG ATGACGCTTC CTCCGGGTCA GTTT

AATTCAAATG ATGACACTGC CTCCAGGTCA GTTT

AATTCAAATG ATGACACTGC CCCCAGGTCA GTTT

>Ortholog Group 296, Repeat 1

3 117

R00004256 ---------- ---------- ---------- ---------- ----------

M00061760 ---------- ---------- ---------- ---------- ----------

H00340595 ATGCGGGGGC GTGGCAGTCC CACACGCCGG CAGGGCCAGA AACTCCCATC

---------- ---------- ---------- ---------- ----------

---------- ---------- ---------- ---------- ----------

TCCCTCACCA GCCGGAAAGT ACGAGTCGGC TCAGCCTGGA GGGACCCAAC

---------- -------

---------- -------

CAGAGCCTGG CCTGGGA

>Ortholog Group 297, Repeat 1

3 204

H00302961 CACAAGTCTG AGGAAAATGA GGAGCCAATG GAAACAGATC AGAATGCAAA

R00023628 CACAAGTCCG AGGAGAGCGA GGAGCCAATG GAGACTGATC AGAATGCAAA

M00020630 CACAAGTCTG AAGAGAGTGA GGAGCCCATG GAGACGGATC AGAATGCAAA

GGAGGAAGAG AAGATGCAAG TGGACCAGGA GGAACCACAT GTTGAAGAG-

GGAAGAAGAG AAGATGCAGG TGGACCAGGA AGAACCACAT ACTGAAGAG-

GGAAGAGGAG AAGATGCAAG TGGACCAGGA AGAACCACAT ACTGAAGAGC

-----ACACC AGCAGAAAAT AAGGCAGAGT CTGAAGAAAT GGAGACCTCT

-----ACACC AGCAGAAAAC AAGGCGGAGT CTGAGGAGAT GGAGACCTCT

AGCAGACACC AGCAGAAAAC AAGGCGGAGT CTGAGGAGAT GGAGACATCT

CAAGCTGGAT CCAAGGATAA AAAGATGGAC CAACCACCCC AAGCCAAGAA

CAAGCTGGAT CAAAAGATAA AAAGACGGAC CAACCACCTC AAGCAAAGAA

CAAGCTGGAT CAAAAGATAA AAAGACGGAC CAACCACCTC AAGCAAAGAA

GGCA

GGCA

GGCA

>Ortholog Group 298, Repeat 1

3 198

R00014862 TTGAGACAGA AAGAACTGAT TGCGTTGGAG CACGAGAGAG AATTAGCTAT

M00124785 ATGAGACAGA GAGAAATGAT TACGTTGGAG CATGAGAGAG AATTAGCCAT

H00402890 CTGAGACAAA AGGAAATGGC CAAGCTGGAG CGTGAAAGAG AATTAGCTCT

GATGGAGCAG GAAATGATGG ATCGACTCAA AGCAGAGGAA CTCTTGTTTC

GATGGAGCAG GAAATGCTGG AGCGACTCAA AGCAGAAGAA CTCTTATTTT

GATGGAGCAG GAAATGATGG AGAGGCTCAA AGCAGAGGAG CTCTTACTTC

TGGCATTCCA GTTGGAGTTG GAAATGCAAG AAAAAGAGAA ACAGAAGACA

TGGCATTCCA GTTGGAGTTG GAAATGCAAC AAAAAGAGAA ACAGAAGTCA

TGGCATTGCA GCTAGAGTTG GAAATGCAAG AAAAGGAGAG GCAGAGAATA

GAAGATTTCA AGACAGAGCA AGAAAAATCC ACTAAGGACA CG---ATG

GAAGATTTCG AGACAGGGCA AGAAAAATCA TTTAAGAGCA TG---ATG

CAAGAACTAC AGAGAGCTCA AGAACAATTA GGCAAGGAGA TGAGAATG

>Ortholog Group 299, Repeat 1

3 183

H00304987 CACGATGCCA AGCACTGGCA TGACATGGAG CTCCAGAACC TGGGCGCTGT

M00116249 CACGACGCCC AGCACTGGCA TGACATGGAA CTGCAGAACC TGGGTGCCGT

R00029527 CACGATGCCA AGCACTGGCA TGATATGGAA CTGCAGAACC TGGGTGCCGT

GGTCGGCCGG CTGGAGGCGG AGCTCAGGGA AATCCGAGCG GAGGCGGAGG

GGTGGGCAGG CTGGAGGCAG AGCTGGCAGA GATCCGCTCA GAGACAGAAG

GGTGGGCAGG CTGGAGGCAG AGCTGGCAGA GATCCACTCA GAGACAGAGG

AGCGCGCGCA TCTGCTGGCC CGCAAGTGCC AGCTGCAGAA GGACGTGGCG

AGCGGGCACA CCTGCTGGCG TGCAAGAGCC AGCTACAGAA GGATGTGGCA

AGCGGGCACA CCTGCTGGCG TGCAAGGGCC AGCTACAGAA GGATGTGGCA

TCCTACCACG CCCTGCTGGA CAGGGAGGAG AGC

TCCTACCACG CCCTGCTGGA CAGAGAGGAG AAC

TCCTACCACG CCCTGCTGGA CAGAGAGGAG AGC

>Ortholog Group 29, Repeat 1

3 198

H00367343 CTGGCCTTCC AGACCCCAGA GGCATACTGC CAGCAGCGAG TGTTCCGCTC

M00030189 CTGGCTTTCC AGACCCCAGA GGCATACTGC CAGCAACAAG TGTTCCGCTC

R00022002 CTGGCTTTCC AGACCCCAGA GGCATACTGC CAGCAGCGAG TGTTCCGCTC

ACTGGCCTAC ATGCGGCCAC TGAGCATATG GGCCATGCAG CTAGCCCTGC

CCTGGCCTAC ATGCGGCCAC TGAGCATCTG GGCCATGCAG CTGGCCCTGC

CCTGGCCTAC ATGCGGCCCC TGAGCATCTG GGCCATGCAG CTGGCCCTTC

ACAAAAAG-- -GCCTCCTGG CCAAAAGTCA AACAGGGCAC AGGACTAAGG

ATAAAAAG-- -AGCCGCAGG CCATCAGTCA CACAAGGCAC GGGACTAAGC

ATAAAAAGAA CAGCAGCCGG CCAGCAGTCA CACAAGGCAC AGCACCCAGC

ACAGGGCCTA TGTTTGGACC AAAGGAAGCC ATGGCAAACC TGAGCCCA

ACACAGCCTG AATGTGGACC AAAGAGATCG CTGGCAAACC TCAATTCA

---CAGCCTG AATGCGGACC AAAGAGATCA CTG------- --------

>Ortholog Group 2, Repeat 1

3 198

R00007079 TCTGCTGGGA AGCCTACCAA CCAGGTGCCA GCTCCCCCAC CCCCTGCCCA

H00262367 TCCACAGGGA AGCCTACCAG CCAGGTGCCG GCCCCCCCAC CCCCGGCCCA

M00023165 TCTGCTGGGA AGCCTACCAA CCAGGTGCCA GCTCCCCCAC CCCCTGCCCA

GCCCCCACCT GCAGCAGTGG AAGCAGCCAG GCAAATTGAA CGTGAGGCCC

GCCCCCTCCT GCAGCGGTGG AAGCGGCTCG GCAGATCGAG CGTGAGGCCC

GCCCCCACCT GCAGCAGTAG AAGCAGCCCG GCAAATTGAA CGTGAGGCCC

ACCTGTACCG AGCAAACATC AACAATGGCA TGCCCCCAGG GCGTGCAGGT

ACCTGTACCG GGTGAACATC AACAACAGCA TGCCCCCAGG ACGCACGGGC

ACCTATACCG AGCAAACATC AACAATGGCA TGCCCCCAGG ACGTGCAGGT

ATGGGGACCC CAGGAAGCCA GATGGCTCCT GTGGGCCTGA ATGTGCCC

ATGGGGACCC CGGGGAGCCA GATGGCCCCC GTGAGCCTGA ATGTGCCC

ATGGGGACCC CAGGAAGCCA AATGACTCCT GTGGGCCTGA ATGTGCCC

>Ortholog Group 2, Repeat 2

3 198

R00007079 GTGGCTGGGC CACGGATGCC CAATGTACAG CCACCAAGGA GCATCTCGCC

H00262367 GTGGCTGGGC CCCGGATGCC CAGCGTGCAG CCACCCAGGA GCATCTCACC

M00023165 GTGGCTGGGC CACGGATGCC CAATGTGCAG CCACCAAGGA GCATCTCGCC

AAGTGCCCTG CAAGACCTGC TTCGGACCCT AAAGTCACCC AGCTCTCCTG

CAGCGCTCTG CAAGACCTGC TGCGGACCCT GAAGTCGCCC AGCTCCCCTG

AAGTGCCCTG CAAGACCTGC TACGGACCCT AAAGTCACCC AGCTCTCCTG

TGCTGAACAT CCTCAAATCA AACCCACAGC TAATGGCAGC TTTCATCAAA

TGCTGAACAT TCTCAAATCA AACCCGCAGC TAATGGCAGC TTTCATCAAA

TGCTGAACAT CCTTAAATCA AACCCACAGC TAATGGCAGC TTTCATCAAA

CAGCGCACAG CCAAGTATGT GGCCAATCAG CCTGGCATGC AGCCCCAG

CAGCGCACAG CCAAGTACGT GGCCAATCAG CCCGGCATGC AGCCCCAG

CAGCGCACAG CCAAGTATGT GGCCAATCAG CCTGGCATGC AGCCCCAG

>Ortholog Group 2, Repeat 3

3 141

R00007079 CCCCAGGGTC AGGCTCTGAA TATCATGAAC CCGGGACACA ACCCCAACAT

H00262367 CCCCAGGGCC AGGCCTTGAA CATCATGAAC CCAGGACACA ACCCCAACAT

M00023165 CCCCAGGGAC AAGCTCTGAA CATCATGAAC CCAGGACACA ACCCCAACAT

GGCGAACATG AATCCGCAGT ACCGAGAAAT GGTGAGAAGA CAGCTGCTAA

GGCGAGTATG AATCCACAGT ACCGAGAAAT GTTACGGAGG CAGCTGCTGG

GACAAACATG AATCCACAGT ACCGAGAAAT GGTGAGGAGA CAGCTGCTAA

GCAGTGCCAG CTTGGCCGGG GGCATGGCAG GACACAGCCA G

GGAGTGCCGG CATGGCTGGG GGCATGGCGG GGCACGGCCA G

ATAGTGCCAG CTTGGCCGGG GGCATGGCGG GACACAGCCA G

>Ortholog Group 2, Repeat 4

3 114

R00007079 TTCCAGCAGC CACAAGGACC TGGAGGCTAC GCCCCAGCCA TGCGCATGCA

H00262367 TTCCAGCAGC CTCAAGGACC CGGAGGCTAC CCACCGGCCA TGCGCATGCA

M00023165 TTCCAGCAGC CACAAGGACC TGGAGGTTAT GCCCCAGCCA TGCGCATGCA

ACAGCACCTC CCCATCCAGG GCAGCTCCAT GGGCCAGATG GCTGCTCCAA

GCAGCATCTC CCCCTCCAGG GCAGCTCCAT GGGCCAGATG GCGGCTCAGA

ACAGCACCTC CCCATCCAGG GCAGCTCCAT GGGCCAGATG GCTGCTCCAA

TGGGACAACT TGGC

TGGGACAGCT TGGC

TGGGACAACT TGGC

>Ortholog Group 2, Repeat 5

3 171

R00007079 CAGATGGGGC AGCCTGGGCT AGGGGCAGAC AGCACCCCTA ATATCCAGCA

H00262367 CAGATGGGGC AGCCGGGGCT GGGGGCAGAC AGCACCCCCA ACATCCAGCA

M00023165 CAGATGGGGC AGCCTGGGCT AGGGGCAGAC AGCACCCCTA ATATCCAGCA

GGCCCTGCAG CAGCGGATTC TGATGAAGCA ACAAATTGGG TCACCAGGCC

AGCCCTGCAG CAGCGGATTC TGATGAAGCA GCAGATTGGG TCCCCAGGCC

GGCCCTGCAG CAACGGATTC TGATGAAGCA ACAAATTGGG TCACCAGGCC

AGCCGAACCC CATGAGCCCC CAGCAGCACA TGCTCTCAGG ACAGCCACAG

AGCCGAACCC CATGAGCCCC CAGCAACACA TGCTCTCAGG ACAGCCACAG

AGCCGAACCC CATGAGCCCC CAGCAGCACA TGCTCTCAGG ACAGCCACAG

GCTTCACATC TCCCTGGCCA G

GCCTCGCATC TCCCTGGCCA G

GCCTCACATC TCCCTGGCCA G

>Ortholog Group 300, Repeat 1

3 204

H00302805 GGACCTCGGG CTCCAGGGCG TGCCCCGAAC CAGCCTCGTG TAGACTTC--

M00053223 GGACTTCGGA CTCCAGGAAG GACTCCAAAC CATCCTCGAG CAGATTTCAC

R00037523 GGACCTCGGC CTCCAGGAAG GGCTCCGAAC CATCCTCGAA CAGATTTCAC

----AGTGGC ATTGCGGTGC CTGGGACGTC CAGCTCCCGT CCACAGCACC

TGGGTCTGGG TCGGCCGTGC CTGGAACATC AAGTTCCCGA CACCCGCATC

TGGGTCTGGG TCGGCTGTGC CTGGAACATC AAGTTCGCGA CACCCACATC

CTGGACGCAC ACCCGCTGCC CAGCGGTCAC AGGGCTTGGC GTCAGGAGAG

AGCATCGTAT ACCATCAACA CAGCAAGCCC ACGGATTAGC CTCTGGAGAG

CGCATCGTAT ACCATCAACA CAGCAAGCCC ACGGATTGGC TTCTGGAGAG

AAGGTGGCCG GCCTGCAAGG TCTGGGCAGC CCCGCCCTGA TCCGCAGCAT

AATATGACCT TTGCTCAGGA ACTCGACAGC CCTGCCCTGA TTCGAAGCAT

AATATGGCTT TTGCTCAGGA TCTCAACAGC CCTGCCCTGA TTCGAAGCAT

GCTG

GCTG

GCTG

>Ortholog Group 301, Repeat 1

3 198

R00035554 GCAGCTGCAG CAATGCAGAT GTTCAAGGAT GAGAACAAGA AAGCCGAGGA

M00026553 GCAGCTGCAG CAATGCAGAT GTTCAAGGAT GAGAACAAGA AAGCTGAGGA

H00341282 GCTGCAGCCA CAGTGCAGCT GTTTCAGGAA GAGCACAGGA AGGCTGAGGA

GCTCCTTGAG GCTGCAGCGC AGCAGCACCA GCAGCTGCAG CAGAGGTGCA

GTTCCTAGAG GCTGCAGCTC AGCAGCACGA GCAGCTGCAG CAGAGGTGCA

GCTCCTAGCA GCTGCTGCCC AGCGCCACCA GCAGCTGCAG CAGAAGTGCA

AGCGGCAGAG GCTGAAAGAA GAGCTGGAAA AGCATGGGGT ACAGATCCTT

AACGGCAGAG GCTGAAGGAA GAACTGGAAA AGCATGGGGT ACAGATCCTT

AGCGGCAGAG GCTGAAGGAA GAGCTGGAAA AGCATGGAAT GCAAGTCCCT

GCTCAAATTC AGAGTACGCA AAAGGAAGAG GACAGCTCGT GGAGGACG

GCT------C ATAGTACGCA AAACGAAGAA GACAGCTCAT GGAGGATG

GCCCAAGCCC AGAGCACACA AGAGGAAGAG GCTGGCCCAG GAGATGTG

>Ortholog Group 302, Repeat 1

3 198

M00056227 AATATCAGCA CTGTTCACGG TCATTATACA CGTGCTCAAG CAAATAGTCC

R00000804 AATATCAGCA CTGTTCACGG TCATTATACG CGTGCTCAAG CAAATAGTCC

H00382204 AACGTCAACG CTGTTCACAG CCATTATACA CGTGCCCAAG CAAATAGTCC

CAGACCAGCA ATGAACTCTC AAGCTGCTGT ACCAAAACAG AACACACACA

CAGACCAGCA ATGAACTCTC AAGCTGCTGT ACCAAAACAG AATACACACA

CAGACCAGCA ATGAACTCCC AAGCTGCTGT ACCAAAACAG AATACACACA

GAAGTATTCG TCCAAATAAG AGGAAGGGTT CAGATAGCAG CATACCCGAT

GAAGTATTCG TCCAAGTAAG AGGAAGGGCT CAGACAGCAG TGCACCTGAT

GAAGTATCCG TCCAAATAAG AGGAAGGGCT CAGATAGCAG TATACCAGAT

GAAGAGAAGA TGAAGGAAGA TAAATATGAT TGTGTATCAC GAGGAGAA

GAAGAGAGGA TGAAGGGAGA TAAATACGAC TATGGATCCC GAGGAGAA

GAAGAGAAGA TGAAGGAGGA AAAATATGAT TATATATCAC GAGGAGAA

>Ortholog Group 302, Repeat 2

3 198

M00056227 ACTGTGTTAC CTGGAGTGCC TACTGCCTCT TTACTCGGTG GCCACCCACG

R00000804 ACTGTGTTAC CTGGAGTGCC TACTGCCTCT TTACTGGGTG GCCACCCGCG

H00382204 ACTGTGTTAC CTGGAGTGCC TACTGCCTCC TTACTTGGTG GCCACCCACG

TCTAGAGAGT GCTCATGCAA GCAGCTTGAG CCATTTAGCA CTAGCACACT

CCTGGAGAGT GCTCACACAG GCAGCTTGAG CCATTTAGCA CTAGCTCACC

ACTAGAGAGT GCTCATGCCA GCAGCTTGAG CCACTTAGCG CTAGCACACT

TGTTACAGCA CCAGTCACCT CATCTTCTTG GACAAGCCCA TCCTTCCGCT

TGCTACAGCA CCAGTCACCT CATCTTCTCG GACAAGCCCA TCCTTCCGCT

TGTTACAGCA CCAGTCACCT CATCTTCTTG GACAAGCCCA TCCTTCTGCT

TCATATAATC AGCTTGGACT TTATCCAATT ATTTGGCAAT ATCCGAAT

TCCTATAACC AGCTTGGACT TTACCCAATT ATTTGGCAAT ATCCGAAT

TCATATAATC AGCTTGGACT TTATCCAATT ATTTGGCAGT ATCCAAAT

>Ortholog Group 303, Repeat 1

3 198

R00052701 CTGGTGACAG GACTTGTAAC CTTGGCAACT ATGTTTTTGC AGGTGTCTAA

H00344563 TTGCTGATTG GGCTGGTGAC ATTGGGGATG ATGTTTTTGC AGATATCTAA

M00032261 CTGGTAACAG GACTGGTGAC CTTGGCAACT ATGTTTTTGC AGGTGTCTAA

TGATATTAAC TCTGATTCAG AGAAGTTGAG TGAACTTCAG AAAATCATCG

TGACATTAAC TCAGATTCAG AGAAATTGAG TCAACTTCAG AAAACCATCG

TGACATTAAC TCTGATTCAG AGAAGTTGAG TCAGCTTCAG AAAAGCATCG

ACAATCTGTC TGAATCACTG AACAACTCCA GAAAG---GG TCTTACAGAG

ATAACTTATC CCAGCAACTG GGCAACTCCA ACAACTTGTC CATGGAGGAG

ACAATCTGTC TGAATCACTG AACAGCTCCA GAAAG---AG TCTGACGGAG

GAATCTCTCC AGTCCCAGAT CTCTGCCCTC CTGGAGAGGC AGGGGCAG

GAATTTCTCA AGTCACAGAT CTCCAGTGTA CTGAAGAGGC AGGAACAA

GAGTCTCTCC AGTCCCAGAT CTCTGCCCTA CTGGAGAGGC AGGAGCAG

>Ortholog Group 304, Repeat 1

3 198

R00017636 GAGTGGCTGT TGATCCGCAC CAGCAGCTTC ACCTTCCAGA ACCCCTACTC

H00307479 GAGTGGATGT TGATCCGCAC CAGCAGCTTC ACATTCCAGA ATCCCTATTC

M00082154 GAGTGGCTGT TGATCCGTAC CAGCAGCTTC ACCTTCCAGA ACCCCTACTC

TGATGAGATC GAGTATGTCA TCTGCACCAA CACCAACGTC AAGCAACTTG

TGATGAGATT GAGTACATCA TCTGCACCAA CACCAACGTC AAGCAACTTG

TGATGAGATC GAGTACGTCA TCTGCACCAA CACCAATGTC AAGCAACTTG

CAGAGCTGGA GGTACACCAG CGAGACGGGC TGTCATCATA TGACTTGTCT

CAGAATTGGA AGTGCACCAG AGAGATGGAT TGTCATCGTA TGACTTATCC

CAGAACTGGA GGTACATCAG CGAGATGGGC TGTCGTCATA TGACTTATCT

CAGGTCCCGG TACCCAACCT ACCCACTGGT GTTCATGAGG CAGGGAAG

CAGGTCCCCG TCCCCAACCT ACCAGCCGGT GTTCATGAGG CCGGGAAG

CAGGTCCCAG TACCCAACCT ACCCGCTGGT GTTCACGAGG CCGGGAAG

>Ortholog Group 305, Repeat 1

3 204

R00053169 ACACAAGCAG AACTGGGTCA TCTTTCAGCT GAAGGCCAAG AGGGTCAGGC

M00110012 ACACAAGCAG AACTGGGTCA CCTTTCAGCT GAAGGCCAAG AGGGTCAGGC

H00359001 ACTCAGGCGG AGCTGGGGCA CCTGTCGGCC GAGGGTCAGG AGGGCCAGGC

TACCACCATA CCCATTGTGC TGACACAGCA GGAGCTTGCA GCTCTTGTAC

TACCACCATA CCCATTGTGT TGACACAGCA GGAGCTTGCA GCCCTGGTGC

CACCACCATA CCCATTGTGC TGACACAGCA GGAGCTGGCT GCCCTGGTG-

AGCTCCAGGA GGCTCAAGCC CAAGCCCAGC AACAGCACCA CCTTCCCACT

AGCTCCAGGA GGCTCAAGCT CAAGCCCAGC AACAGCACCA TCTTCCCACT

--CTGCAGGA GGCCCAGGCC CAG---CAGC AGCATCACCA CCTCCCCACT

GAGGCTCTGG CCCCAGCTGA CAGTCTCAAT GACCCATCCA TCGAGAGCAA

GAGGCTCTGG CCCCAGCTGA CAGTCTCAAT GACCCATCCA TCGAGAGCAA

GAGGCCCTGG CCCCTGCCGA CAGTCTCAAC GACCCAGCCA TTGAGAGCAA

CTGC

CTGC

TTGC

>Ortholog Group 306, Repeat 1

3 198

M00086285 AGCATGGAAG AGAAAATCGC CTATCAGTCA TATGAACGGG CCCGGGATAT

H00327349 AGCATGGAAG AAAAAATCGC GTATCAGTCC TATGAACGGG CCCGGGACAT

R00039858 AGCATGGAAG AGAAAATCGC CTATCAGTCA TATGAACGGG CCCGGGACAT

CCAGGAGGCT CTGGAGGCCT GTCAAACCCG CATTTCCAAG ATGGAGCTGG

CCAGGAGGCC CTGGAGGCAT GCCAGACGCG CATCTCCAAG ATGGAGCTGG

CCAGGAGGCC CTGGAGGCCT GTCAAACCCG CATTTCCAAG ATGGAGCTGG

TGGTGCAACT GGAAGGGCTG GAGAATGCCA CTGCCCGAAA CCTTCTGGGC

TGGTGCAGCT AGAAGGGCTG GAGAATGCCA CTGCCCGGAA CCTTCTGGGC

TGGTGCAACT GGAAGGGCTG GAGAATGCCA CTGCCCGAAA CCTTCTGGGC

AAACTCATCA ATATCCTCCT TGCTGTCATG GCAGTTCTCT TGGTCTTT

AAACTCATCA ACATCCTCCT GGCTGTCATG GCAGTCCTTT TGGTCTTT

AAACTCATCA ATATCCTCCT GGCTGTCATG GCAGTCCTCT TGGTCTTT

>Ortholog Group 307, Repeat 1

3 207

H00342144 ACCAAGTCGG CCTGCACACA GATCCTGGAG TCGCACAGCC GGGAGCTCCA

M00055130 ACCAAGTCAG CCTGTGTGCA GATCCTGGAG TCCCACAGGC CGGAGCTGCA

R00059979 ACCAAGTCAG CCTGCGTGCA GATCCTGGAG TCCCACAGGC CGGAGCTTCA

G--------- GAGTTGGAGA GGAAGCTGGA GGACCAGCTG GTGCAGCAGG

GGAGCTGCAG GAGCTGGAGA GGAAGCTGGA GGACCAGCTG GTGCAGCAGG

GGAGCTGCTG GAGCTGGAGA GGATGCTGGA GGACCGGCTG GTGCAGCAGG

AGGCAGCCGC CCTGGCGAGC TGGCAGCAGT GGGTGGCCGA TGGGCCCGGG

AAGAGGCCGT CCTGGAGAGC TGGCAGCGGT GGGCGGCCGA TGGACCTGGG

AAGAGGCTGT CCTGGAGAGC AGGCAGCGGT GGGCGGCTGA CGGACCTGCG

ATTCTGAACG AACCTGGGGA GGTGGATTCT GAAAGGCAGG TCTCTACTGT

---CTGAGCG AACCTGAGGA GATGGATCCT GAAAGGCAGG TCTCTGCCAT

---CTGGGCG AGCCTGAGGA GATAGATCCC GAGAGGCAGG TCTCTGCCGT

CCTGCAC

CCTGCGG

CCTGCGG

>Ortholog Group 308, Repeat 1

3 201

R00034197 GCGTGGGGAG AAGCCGATTC CCGCGCCAAT GGCTACCCCC ACGCCCCCGG

H00419361 GCGTGGGGCG AGGCCGATTC CCGCGCGAAT GGCTACCCCC ATGCCCCCGG

M00023549 GCTTGGGGAG AAGCCGATTC CCGCGCCAAT GGCTACCCCC ACGCCCCCGG

GGGATCGACC CGCGGCTCCA CCAAGCGATC TGGGGGAGCG GTGACCCCAC

GGGCTCTGCC CGCGGCTCCA CCAAGAAACC CGGGGGGGCG GTGACCCCGC

GGGATCAACC CGCGGCTCCA CCAAGAGATC TGGGGGAGCG GTGACCCCAC

GCCTGGCTAG CCGTTGGCGC GGTGGCGACG ACGATGAAGA CCCTCCACTA

GCCTGGCCAG CCGCTGGCGC ---AGCGACG ACGACGACGA TCCTCCGCTG

GCCTGGCCAG CCGTTGGCGC GGTGGCGATG ACGACGAAGA CCCTCCACTA

AGCGGTGACG ACCCTCTGGT CGGAGGCTTC GGCTTCAGCT TCCGTTCTAA

AGCGGTGACG ACCCCCTGGC CGGGGGCTTC GGCTTCAGCT TCCGCTCCAA

AGCGGTGATG ACCCTCTGGC TGGGGGCTTC GGCTTCAGCT TCCGCTCTAA

G

G

G

>Ortholog Group 309, Repeat 1

3 198

R00012758 GCTTCATATT CTCCAATTCA GCCTCATTCT CTAATCAAAC ATCAACAGAT

H00420294 GCTTCATATT CTCCAATTCA GCCTCATTCT CTAATAAAAC ATCAGCAGAT

M00114916 GCTTCATATT CTCCAATTCA GCCTCATTCT CTAATCAAAC ATCAGCAGAT

TCCTCTCCAT TCACCACCTC CTAAAGTTTC CCATCATCAG CTGATATTAA

TCCTCTTCAT TCACCACCTT CCAAAGTTTC CCATCATCAG CTGATATTAA

TCCTCTTCAT TCACCACCTC CTAAAGTTTC CCATCATCAG CTGCTATTAA

TTCAGCCAAT CACCCTTCAG AGTCCAAGTC AAGATCCACC CCCATCCCAG

TTCAGCCAAT CACACTTCAG AATTCAACTC AAGACCCACC CCCATCCCAG

TTCAGCCAAT CACCCTTCAG AGTCCAAGTC AAGATCCACC CCCATCCCAG

CACTGTATCC CACTCCCCAA CCATGGCCTT TCTCCGGCTT CCAGTAAT

CACTGTATAC CACTCCAGAA CCATGGCCTT CCTCCAGCTC CCAGTAAT

CACTGTATCC CACTCCCAAA CCATGGCCTT TCTCCGGCTC CCAGTAAT

>Ortholog Group 30, Repeat 1

3 198

R00044325 AGCCTGCACA TCAATGGTGC GGGCAGTGCC TCCGAGCAGC GGGAGAAGGT

M00099885 AGCCTGCACA TCAACGGTGC GGGCAGTGCC TCTGAGCAGC GCGAGAAGAT

H00176763 AGCCTCCACA TCAACGGCGG GGGCAGCGCA GCTGAGCAGC GTGAGAAGAT

CAAGCAGTTC TCCCAGCAGG AAGAGAAGAG GCAGAAGGCG GAGAGGCTGA

CAAGCAGTTC TCCCAGCAGG AGGAGAAGAG GCAGAAGGCG GAGAGGCTGA

CAAGCAGTTC TCCCAGCAGG AGGAGAAGAG GCAGAAGTCG GAGCGGCTGA

AACACGAGAA CCAGATGCGA GACATGGTGG CACAGTGCGA GAGCAACATG

AACACGAGAA CCAGATGCGA GACATGGTGG CGCAGTGTGA AAGCAACATG

AACACGAGAA CCAGATGCGG GACATGCTGG CGCAGTGTGA GAGCAACATG

AACGAGCTGC AGCAGCTGCA GAATGAAAAG TGTCATCTGT TAGTGGAG

AGCGAGCTAC AGCAGCTGCA GAATGAAAAG TGTCATCTGT TGGTAGAG

AGCGAGCTGC AGCAGCTGCA GAATGAAAAG TGCCACCTCC TGGTAGAG

>Ortholog Group 310, Repeat 1

3 198

M00114006 CTGGCAGCGA GGAGAGATGA GGAAGCCCAG CGGCTGAGAG AACATCAGGA

H00417354 CTGGCAGTGC AGCAGGTGGA GGAGGCCCAG CAGCTGCGGG AACACCAGGA

R00060365 CTGGCAGCAA GGAGAGATGA GGAAGCCCAG CGGCTGAGAG AACATCAAGA

AGCCTTGCAC CAACAGAGGC TCCATGGGCA GTTGCTAAGA CAACAG---C

AGCTTTGCAC CAGCAGAGGC TGCAGGGGCA CTTACTACGG CAGCAGGAAG

AGCTTTGCAC CAACAGAGGC TCCACGGACA GTTGCTACGA CAACAG---C

TGGCCAGAGA GATGGCTCAG CAGAAGCAGG TAGCCCATGA AGATGGCCAG

TGGCAAGAGA GATGGCCCTG CAGAGGCAGG CTGAGCTTGA GGAGGGCCGG

TGGCCAGAGA GATGGCTCAG CAGAAGCAGG CAGACCATGA GGAAGGCCAG

CAGCAGCACC AGGAGCAGCT AAGGCAGCAA GCTCATTACA ATGCTGTG

CCGCAGCACC AGGAGCAGCT CCGGCAGCAA GCTCATTATG ATGCTATG

CAGCAGTACC AGAAACAAGA CAGGCAGCAA GCTCATTCTG ATGCTGTG

>Ortholog Group 311, Repeat 1

3 201

R00030887 AGAACTACTG AACAGCACGT CCCAGAGATT GAGGTCCAGG TCAAACGTAG

H00402125 TGGACTACAG AACAGCACAT GCCCGAGGTG GAAGTTCAAG TCAAA---TA

M00096003 AGAACTACTG AACAGCACGT GCCCGAGGTC GAGGTCCAGG TCAAACGTCG

AAGGACAGCC TCACTGAGCA ACCAAGAGTG TCACTTGTAC CCACGGCATT

CAGAACAGCT GCACTGAGCA ACCAAGAGTG TCAATTGTAC CTGAGGCATT

AAGGACAGCC TCACTGAGCA ACCAAGAGTG TCACTTGTAC CCACGACGTT

CTATTCCTGT GGTGGATTTC CAGGCAGAAC TGAGACAGGC GTTCTTAGCT

CTGTGCTTGT GGTGGATTTC CAGGCCAAAC TGAGACAGGT ATTCATAACT

CTGTTCCTGT GGTGGATTTC CAGGCAGAAC TAAGACAGGC ATTCTTAGCT

GAGACACCAA GAGGTGGT-- ---------- ---------- ----------

GAGACACCAA GATGTGGTAA AAAGCCGTAC TGGAACAATG AGGAAGCTGA

GAGACACCAA GAGGTGGT-- ---------- ---------- ----------

-

A

-

>Ortholog Group 312, Repeat 1

3 198

H00330659 GGCGGCCGGC TTCTGCTGCA GCCCCCAGGC CCTGAATTAC CTCCGGTGCC

M00040896 GGTGGCCGGC TGCTTCTGCA GCCTCCGGGT CCCGAACTAC CCCCGGTGCC

R00014943 GGTGGCCGGC TGCTGCTGCA GCCTCCGGGC CCCGAACTAC CCCCGGTGCC

CTTCCCGCTG CAGGACTTGG TCCCTCTGGG GCGCCTGAGT AGAGGGGAGC

CTTTCCGCTG CAGGACTTGG TCCCTCCAGG GCGCTTGAGC CGAGGGGAGC

CTTTCCGCTG CAGGACTTGG TCCCTCCAGG GCGCCTGAGC CGAGGGGAGC

CTCCCCCGCC CCCGCCTCCT CCCGGGCCCC TCCGGCCACT CGCGGGTCCT

CTCCCCCACC GCCGCCGCCT CCCGGGCCCC TCCGGCCTCT CGCAGGCCCT

CTCCCCCACC GCCGCCGCCT CCCGGGCCCC TTCGGCCGCT CGCAGGCCCT

TCTCGGAAGG GCTCCTTCAA AATCCGCCTC AGTCGCCTCT TTCGCACC

TCTCGGAAGG GCTCCTTCAA AATCCGCCTC AGTCGCCTGT TTCGCACG

TCTCGAAAGG GCTCCTTCAA AATCCGCCTC AGCCGCCTGT TTCGCACG

>Ortholog Group 313, Repeat 1

3 198

H00219905 AATTCTTCAG GCATCATCTC AGAAAATCAG GCGAATAACT TTGTTGTGCC

R00060519 AATTCCTTAA GTTCGGCTTC AGATAATCAG GTGACTAACT TGGTTGTGCC

M00106401 AATTCCTTGA GTTCGACCTC AGATAATCAG GTGACTAACT TGGTTGTGCC

AACTTTGGAT GAAAATATAT TTCCAAAGCA GATTAGTTTG CGGCAGGCAG

ATCTATAGAT GAAAGTGCAT TTCCAAAGCA GATTAGTTTG CGGCAGGCCG

ATCTGTAGAT GAAAATGCAT TTCCAAAGCA GATTAGTTTG CGGCAGGCCG

GAAGTCGCCC TCCAGGCTTG TCTAAATCTC AGGTGAAGCT AATGGACCTG

GAACTCGCCC TCCAGGCTTG TCTAAATCTC AAGTAAAGCT TATGGACCTG

GAACTCGCCC TCCAGGCTTG TCGAAATCTC AAGTAAAGCT TATGGACCTG

GAAGACTGTG CACTTTGGGA AGGAAAACCA AGGACATACA TCACAGAA

GAAGACTGTG CACTCTGGGA AGGAAAACCC AGGACCTATA TTACTGAA

GAAGACTGTG CACTCTGGGA AGGAAAACCA AGGACCTATA TTACTGAA

>Ortholog Group 314, Repeat 1

3 198

M00089036 AGCTGGCGAG ATCCGCAGGA CGACGTGGCC GGGGGCAACC CCGGCGGCCC

H00325819 AGCTGGCGAG ATCCGCAGGA CGACGTGGCC GGGGGCAACC CCGGCGGCCC

R00020044 AGCTGGCGAG ATCCGCAGGA CGACGTGGCC GGGGGCAACC CCGGCGGCCC

CAACCCCGCA GCGCAGGCAG CCCGCGGCGG CGGCGGCGGC ---------G

CAACCCCGCA GCGCAGGCGG CCCGCGGCGG CGGCGGCGGC GCCGGCGAGG

CAACCCCGCA GCGCAGGCAG CCCGCGGCGG CGGCGGCGGC ---------G

CGGGCTCCGG CGCGCCGCAC ACGCCGCAGA CCCCGGGCCA GCCCGGAGCG

CGGGCTCGGG CGCGCCGCAC ACGCCGCAGA CCCCGGGCCA GCCCGGAGCG

CGGGCTCCGG CGCGCCGCAC ACGCCGCAGA CCCCGGGCCA GCCCGGAGCG

CCCGCCACCC CCGGCACGGC AGGGGACAAG GGCCAGGGCC CGCCCGGT

CCCGCCACCC CCGGCACGGC GGGGGACAAG GGCCAGGGCC CGCCCGGT

CCCGCCACCC CCGGCACGGC GGGGGACAAG GGCCAGGGCC CGCCGGGT

>Ortholog Group 315, Repeat 1

3 198

R00050958 ---------- ---------- ---------- ---------- ----------

H00371975 CACGGCCACC ACAACAGCCA CCACCCCCAG CACCACCACC ACCACCACCA

M00106374 ---------- ---------- ---------- ---------- ----------

---------- ---------- ---------- ---------- ----------

CCATCACCAC CACCCGCCGC CGCCCGCCCC GCAACCGCCG CCGCCGCCGC

---------- ---------- ---------- ---------- ----------

---------- ---------- ---------- ---------- ----------

CGCCGCCGCC GCCGCCCCCG GCACCGCAGC CCCCCCAGAC GCGGGGCGCC

---------- ---------- ---------- ---------- ----------

---------- ---------- ---------- ---------- --------

CCGGCCGCCG ACGACGACAA GGGCCCCCAG CAGCTGCTGC TCCCGCCG

---------- ---------- ---------- ---------- --------

>Ortholog Group 316, Repeat 1

3 198

R00040722 CCGCAGCAAA CCCAGGCAGG CCAACATCTG CAGGCACTGC AGCAGCATGT

H00320794 CAGCAGCAAA CCCAGGCAAG TCAGCATCTA CAGGCCCTGC AGCAGCATGT

M00112620 CAGCAGCAAA CCCAGGCAGG TCAACATCTG CAGGCACTGC AGCAGCATGT

ACCCCCTCAA GGCGTGTCTC ACCTTCACAC CAACCATCTG TACCTCAAGG

TCCAGCTCAA GGTGTATCTC ACCTTCATAG TAACCATCTC TACATAAAGG

ACCACCTCAA GGAGTGTCTC ATCTTCACAC CAACCATCTG TACATCAAGG

CAGGGCAGCT AAGGGAGAGG CATCACTTGT ATCAGCTGCA GCATCATGTG

CAGGGCAGTT AAGAGAGAGG CATCACTTAT ATCAAATGCA GCATCATGTA

CAGGGCAGTT AAGAGAGAGG CATCACTTGT ATCAGCTGCA GCATCATGTG

CCTCATGCAG ATAATGCTGT CCACTCCCAG CCCCATATCG TGCACCAA

CCTCATGCAG AGAGCTCTGT CCACTCTCAG CCCCATAATG TCCACCAA

CCTCATGCAG ACAGTGCTGT CCACTCCCAG CCCCATATTG TGCACCAA

>Ortholog Group 317, Repeat 1

3 141

M00124694 ---------- ---------- ---------- ---------- ----------

R00033445 ---------- ---------- ---------- ---------- ----------

H00357631 ATGCAGGCGG GCGCCAGGTT CCAGCGGCGG CGGCGGCAGC TGCCCCGGCG

---------- ---------- ---------- ---------- ----------

---------- ---------- --ATGAATGC AGAC---CCG CCGCCACCGC

GCGGCAGCCT CTCCTCTGGC CGATGGACGC AGAGCCGCCG CCGCCGCCGC

---------- ---------- ---------- ---------- -

CCTGGGTTTG GATGGTCCCC GGCACCGCCG GGCTGCTCCG G

CCTGGGTCTG GATGGTGCCG GGCTCGGCCG GGCTGCTCCG G

>Ortholog Group 318, Repeat 1

3 198

M00102015 CAGAGACAGT GCCAAGAGGA GGTGGCCTCC CTGCAGGCCA TCCTGAAAGA

H00350934 CAGCGGCAGT GCCAAGAGGA GGTGGCCTCG CTGCAGGCCA TCCTGAAAGA

R00025043 CAGAGACAGT GCCAAGAGGA GGTGGCTTCC CTGCAGGCCA TCCTGAAAGA

CTCCATCAGC AGCTACGAGA CCCAGATCGC AGCCCTGAAA CAGGAGCGGG

CTCCATCAGC AGCTATGAAG CCCAGATCAC CGCCCTGAAG CAGGAGCGAG

CTCCATCAGC AGCTACGAGA CCCAGATCGC AGCCCTGAAA CAGGAGCGGG

ACTTTGAGGA GAAGGATCGA GAGCTGGGCC ACCTCAAACA GCTGCTGGCC

ACTGTGAGGA GAAGGAGCGG GAGCTGGGCC GCCTGAAGCA GCTGCTGTCC

ACTCTGAGGA GAAGGAGCGA GAGCTGGGCC ACCTCAAACA GCTGCTGGCC

CGGGCCCACC CTTTGGACTC CTTGGAGAAG CAAATGGAAA AGGCTCAT

CGGGCCTACC CCCTGGACTC CCTGGAGAAG CAGATGGAAA AGGCCCAC

CGGGCCCACC CTTTGGACTC CTTGGAGAAG CAAATGGAAA AGGCTCAC

>Ortholog Group 319, Repeat 1

3 198

R00011732 GTCTACGCCA ACCTCAGCAA CTTCAACCCG GGTGCGCTGA GCAGCGGCGG

M00102711 GTCTACGCCA ACCTCAGCAA CTTCAACCCG GGTGCGCTGA GCAGCGGCGG

H00360266 GTCTACGCAA ACCTCAGCAA CTTCAACCCA GGCGCGCTGA GCAGCGGCGG

TGGGGCGCCC TCCTATGGCG CGACCGGGCT GGCCTTTCCA TCGCAGCCCC

TGGGGCGCCC TCCTATGGCG CGGCCGGGCT GGCCTTTCCC TCGCAGCCGC

CGGGGCGCCC TCCTACGGCG CGGCCGGCCT GGCCTTTCCC GCGCAACCCC

CGCCGCACCA CTTGCCCCAA CAGATCCCGG TGCAGCACCC GCGGCTGCAG

CGCCGCACCA CTTGCCCCAA CAGATCCCGG TGCAGCACCC GCGGCTGCAA

CGCCGCACCA CCTGCCCCAG CAGATGCCCG TGCAGCACCC GCGGCTGCAG

GCGCTGAAGG AAGAGCCGCA GACGGTGCCG GAGATGCCGG GAGAGACG

GCCCTGAAGG AAGAGCCGCA GACCGTGCCG GAGATGCCGG GAGAGACG

GCCCTGAAGG AGGAGCCTCA GACAGTGCCC GAGATGCCCG GCGAGACA

>Ortholog Group 31, Repeat 1

3 198

H00264956 GCTCATGAGA CCTCCCAGGC GGTCCACCAG AGGATGCTGT CCCAGCAGAA

M00031005 ACCCATGGAG CTTCCCAGGG GGTACAGCAG AGGATGCTTT CGCAGCAGAA

R00010541 ACCCATGGAG CGCCCCAGGC TGTACAGCAG AGGATGACGT CACAGCGGAA

GAGGTTCCTG GCCCAGTTCC CAGTGCACCA GCAGATGCGT CTGCACGCCG

GAGGCTCCTG GACCAGTTCA CGAAGCATCA GCAAGGCCGC CTGAACTCTG

GAAGCTCCTG GACCAGTTCA CAAAGCACCA GCAGGGCCGC CTGAACTCTG

CAGGAGTCAT GGACCTTCTG GAAGCCCAGC TGGAGACCCA GCTACAGGAA

CACAAGAGCT GGACCAGCTG CAAGCCCAGC TTGAGACCCA GCTGCAGGAA

CACAAGAGCT GGACCAGCTG GAAGCCCAGC TTGAGACCCA GCTGCAGGAA

GCTGAACAGA ACTTCATCTC CGAGCTGGCA GCCTTGGCCC GAGTGCCC

GCTGAGCAGA CCCTTATCTC AGAGCTATCG ACATTGGCCC GAGTACCC

GCTGAGCAGA CCCTTATCTC GGAGCTGGCG ACATTGGCCC GAGTGCCC

>Ortholog Group 320, Repeat 1

3 198

R00047019 CAACGCGAAC GCTTCATCGA GCAGGAGAAG GCCAAGCTGG AGCAGCTTTT

H00323856 CAGCGGGAGC GCTTCATCGA GCAGGAGAAG GCCAAGCTGG AGCAGCTCTT

M00073124 CAGCGCGAGC GCTTCATCGA GCAGGAGAAG GCCAAGCTGG AGCAGCTCTT

CCAGGACGAG GTGGCAAAAG CAAAACAGCT GCAGGAGGAG CAGCAGCGGA

CCAGGACGAG GTGGCCAAGG CACAGCAGCT GCGTGAGGAG CAGCAGCGGA

CCAGGACGAG GTGGCTAAAG CAAAGCAGCT GCGTGAGGAG CAGCAGCGGA

TGGAGCAGGA AAAGCAGGAG CTGGTGGCCA GCATGGAGGA GGCCCGGAGG

TGGAGCAGGA ACGGCAGCGG CTGGTGGCCA GCATGGAGGA GGCGCGGCGG

TGGAACAGGA AAAGCAGGAG CTGATGGCCA GCATGGAGGA GGCCAGGAGG

CGGCAGCGTG AGGCAGAGGA GGGTGTGAGG CGCAAGCAAG AGGAACTG

CGGCAGCATG AGGCCGAGGA GGGCGTGCGG CGCAAGCAGG AGGAGCTG

CGGCAGCGCG AGGCTGAGGA GGGCGTGAGG CGCAAGCAAG AGGAACTG

>Ortholog Group 321, Repeat 1

3 201

M00095320 TTATTGGATG AGACCACAAC TTTCAAGCCA GGCCATGCTC GGTCAAGGTC

R00030422 TTATTGGACG AGACCACGAC TTTCAAGCCA GGACATGCTC GGTCAAGGTC

H00417003 TTACTGGATG AGACCTCAAC TTTCAAGCCA GGGCATGCTC GATCACGATC

CGATGTTACC CACGTAGACT GGCGGGTAGT CCTTAGCACC ATGCCTTTGC

CGATGTTACC CATGTTGACT GGCGGGTAGT CCTGAGCACC ATGCCTTTGC

AGATATTACC CAAGTGGACT GGAGGGTAGT CCTCAAAACC ACGCCTTTGC

AGGCGTCCCT TCAAGGCATT CACTTTCCCG GGCCATCTTT TCTGTTGTCT

AGGCGTCCCT TCACGGCCCT CACTTCCCCA GGCCGTCTTT TCTGTTGTCT

CATTG---CT TCAAGGCCCG CATGTCACCA GGCCATCTTT TCTGTTGCCC

TCACCCAGCA AGGTTGAAGA TGCTCAAGGA AATACAGAAC ACAAGCAGAC

TCATCCAATA AGGTTGAAGA TGCTCAAGGA AATACAGAAC ACAAGCAGAC

TCACCAAATA AGATAGAAGA TGCTCAAGGA AATACTGAAC ACAAGCAGAC

A

A

A

>Ortholog Group 322, Repeat 1

3 174

H00312767 AGAGCCCAGG AAAGGGAGAG AGGATCTGAG CTCAGCACCA CTTCCCCATC

M00099313 GGGATCTGTG CCCTG----- ----CCCCCT GACCGAAACA ACCTGTCCCT

R00043063 GGGGTCCAGG CC-------- ----CCGCCT GTTGAACGAA ATATTCCTCT

GGGCACCAGC CTCGCCGACG ACGACCGGCA CAGTCAGATG ATCCGAGACA

GGACAACGGC CTTCTCGATG AGGACAAACA GAACCAGATG ATTCGTGACA

GGACAACGGT CTTCTCGATG AGGACAAACA GAACCAGATG ATCCGTGACA

TCTTACAGCA AAACAGGTTG CACAAGCAGT TTCTTGAAGA AGCCCGGAAA

TCTTAGAGCA AAACAAGTTA CACCAGGAGT TTTTGGAAAA AGCCAGGAAA

TCTTAGAGCA AAACAAGATA CACCAGGAGT TTTTGGAAAA AGCCAGGAAA

TGCTTGCGGG AGTTTCAGAA CATA

CGCTTGCAAG AGTTTCAAAA ATCA

CGCTTGCAAG AGTTTCAGAA AACA

>Ortholog Group 323, Repeat 1

3 123

M00001059 GTGGACTCCA TGCTCAAGGA CATGGCTACC ATTATCCTGA GCACCTTCCT

R00017371 GTGGACTCCA TGCTCAAGGA CATGGCTACT ATTATCCTGA GCACCTTCCT

H00401445 GTGGACTCCA TGCTTAAGGA CATGGCTACC ATCATCCTGA GCACCTTCCT

GCTGGTTGGA TGGGTGGCGT TCATCATCAC TTACCCCCTG AGCGTGCATC

GCTGGTCGGA TGGGTGGCGT TCATCATCAC TTACCCCCTG AGCATGCATC

GCTGATTGGC TGGGTGGCCT TCATCATCAC CTATCCCCTG AGCATGCATC

TCCAGCACCA ACAGTTCCAG AAG

TCCAGCACCA GCAGTTCCAG AAG

TCCAGCACCA GCAGTTCCAG AAG

>Ortholog Group 323, Repeat 2

3 123

M00001059 GAGCTGGAGA AGATTCAGCT CCTGCTGCCC TTCCACCCAC ACGGAGACCT

R00017371 GAACTGGAGA AAATTCAGCT CCTTCTGCCC TTCCACCCAC ACGGAGACCT

H00401445 GAACTGGAGA AGATCCAGCT CCTGCTGCCC TTCCACCCAC CTGGAGACAC

TACCCAGGAC CCTGAGTTCC TGGATTCATC TGGCCCCTTC TCAGAGAGCT

TACCCAGGAC CCTGACTTCC TGGATTCATC TGGCCTCTTC TCGGAGAGCT

GGCTCAGGAC GGCGAGCTCC TGGACACGTC TGGCCCGTAC TCAGAGAGCT

CTGGCACCAG CAGCCCCAGC CCA

CAGGCACCAG CAGCCCCAGC CCA

CGGGCACCAG CAGCCCCAGC ACG

>Ortholog Group 324, Repeat 1

3 198

H00313490 CCCAACAACG TCCTCAAATT CTGTGATAAC AGCAGTGCAA TCCAGGGAAA

R00007087 CCTAACAATG TCCTCAAGTT CTGTGACAAC AGCAGTGCAA TCCAAGGGAA

M00021282 CCTAACAATG TCCTCAAGTT CTGTGACAAC AGCAGTGCAA TCCAGGGGAA

GGAAGTCCGA TTCCTACGGC CTGAGGACCC CACACGGCCA AGCCGCTTCG

GGAAGTCCGA TTCCTGCGGC CTGAGGACTC TACACGACCA AGCTGCTTCG

GAAAGTCAAA TTCCTGCGGC CGGAGGACTC TACCCGCCCA AGCTGCTTCG

GGCTGAGACA TGTTGTCTTC ACAGCAGAGA CTCACAACTT TCCCACAGGA

GGCTTAGACA TGTTGTCTTC ACAGCAGAGA CACATAACTT CCCCACCGGA

GGCTTAGACA TGTTGTCTTC ACAGCAGAGA CACATAACTT CCCCACAGGA

GTATGCCCCT TTAGTGGTGC AACCACTGGC ACAGGGGGCC GGATTCGA

GTGGCGCCCT TCAGCGGTGC AACCACAGGC ACAGGTGGCC GCATCAGA

GTGGCCCCCT TCAGCGGTGC AACCACAGGC ACGGGTGGCC GCATCAGA

>Ortholog Group 325, Repeat 1

3 198

H00401343 ACAGGCAAGT TTGTCAAGAA GCCAGCCTCT TCCAGCAGTG CCCCTCAGAA

M00049146 ACAGGCAAGG TGGTCAAGAA GCCAGCCTCT TCCAGTAGTG CCCCTCAGAG

R00026492 ACAGGCAAGG TGGTCAAGAA GCCAGCCTCT TCCAGTAGTG CCCCTCAGAG

TATCCCTAAG AGGACAGATG TGAAGAGCCA GGATGTTGCC GTTTCCCCGT

CATCCCTAAG AGGACAGATG TGAAGAGCCA GGATGTTGCC ATTTCTCCTT

CATCCCTAAG AGGACAGATA TGAAGAGCCA GGACGTTGCC ATTTCTCCTT

GCTCAAAGAG CTATGTCGAC AGGCACATGG AATCCTTGAG TCAGTCCAAA

GCTCAAAGAG TTATGTGGAC AGGCACATGG AATCTTTGAG TCAGTCCAAA

GCTCAAAGAG TTATGTGGAC AGGCACATGG AGTCTTTGAG TCAGTCCAAG

AGTTTCCGTC GTCGGCACAA CTCCTGGTCA TCTAGTAGCA GGCACCCA

AGCTTCCGTC GTCGGCACAA CTCCTGGTCA TCTAGTAGCA GGCACCCA

AGCTTCCGTC GTCGGCACAA CTCCTGGTCA TCTAGTAGCA GGTATCCC

>Ortholog Group 326, Repeat 1

3 198

M00037783 CTGCGCTTGC AGCAGCAGGC CTTGGAGGCA AGGCGCCAAG CCAGGGAGCA

H00323065 CTGCGGGTGA AGCAGCTGGC CGAAGAGCAG AAGCGTCGGG AGAGGGAGCA

R00004027 CTGCGCGTGC AGCAGCAGGC CGCGGAGGCA AGGCGTCAAG CCAGAGAGCA

GCGTATCGCA GAGTGCATGG CCAAGATGCC ACAAATGATT GAAAACTGGC

GCACATCGCA GAGTGCATGG CCAAGATGCC ACAGATGATT GTGAACTGGC

GCATATTGCA GAGTGCATGG CCAAGATGCC ACAAATGATT GAGAACTGGC

GAGAACGCTG GGAGAAAATT CAGGCTGACA AGGAGCGGAG GGCCCGGTTA

GGGAGAACTG GGAGAAGGCC CAGGCTGACA AGGAGAGGAG GGCCCGACTG

GAGAACGCTG GGAGAAAATT CAGGCTGACA AGGAGCGGAG GGCCCGGTTA

CAGGCTGAGG CCCAGGAACG CCTGGGCTAC CACGTGGACC CAAGGAGT

CAGGCTGAGG CCCAGGAGCT CCTGGGCTAC CAGGTGGACC CAAGGAGT

CAGGCTGAGG CCCAGGAACA ACTGGGCTAT CATGTGGACC CAAGGAGT

>Ortholog Group 327, Repeat 1

3 111

M00004780 ATGGAGCCTC TGAAGCAGCC ACAGCAGCCT CTGCTACAGA TGGATGCCAG

H00378515 ATGGAGCCAC TTAAGCAGCC ACACCTGGCT CCTCTGCAGA TGGATGCCAG

R00062477 ATGGAGCCTC TGAAGCAGCC ACAGCAGACT CCGCTACAGA TGGATGCCAG

AGAGAAGCAG GGACCACAGA CGAGAGAATC ACAGTTCCTG TATGCCTCAA

AGAGAAGCAG GGCCAGCAGA TGAGAGAAGC CCAGTTCTTG TATGCCCAAA

AGAGAAGCAG GGATCACACA CAAGAGAAGC ACAGTTCCTG TATGCCCCAA

AACTAGGCAC G

AGCTGGTCAC A

AACTAGGCAC G

>Ortholog Group 328, Repeat 1

3 198

H00426190 CTTCACGACG AGCGCGACTG CCTCTACGAG TACGTGCCCG ACAACGTGGT

R00060272 CTGCACGACG AGGGTGACTG CCTATACGAA TACGTGCCCG ACAACGTGGT

M00077943 CTGCATGACG AGGGTGATTG CCTGTATGAG TACGTGCCCG ACAACGTGGT

GTCCATGCGG CGCATCGTCA ACACCGTGCC CATCACCGTG CGCCTGCTGG

GTCCATGAGG CGCATCGTCA ACACGGTGCC CATCACCGTG CGCCTGTTGC

GTCCATGCGG CGCATTGTCA ACACGGTGCC CATCACCGTG CGCCTGTTGC

GGGACTTTGG GGGCCCCACG CCGCGCCAGG CGGTGGCGTG GGTGGTGCTC

CGGACCGCGT GGGACCCACG CCGCGCCACG CGGTGGCCTG GGTCGTGCTC

CCGACCGCGT GGGACCCACA CCGCGCCACG CGGTGGCCTG GGTCGTCCTC

GCCAACCAGT GGCCGTGCCG CCTGAGCTGG GCGCTGCAGT GCCTGGAG

GCCAACCAGT GGCCTTGTCG CCTCAGCTGG GTGCTGCAAT GCCTGGAG

GCTAACCAGT GGCCTTGTCG CCTCAGCTGG GTGCTGCAAT GCCTGGAG

>Ortholog Group 329, Repeat 1

3 156

R00020529 AGTGTGGGCA TGAACCTGAC TCGGACAGCC ACACCTGCCC CCAGCCAGAC

H00323846 AATGTGGGCA TGAACCTGAC ACGGACAGCC ACACCTGCGC CCAGCCAGAC

M00125568 AGTGTGGGCA TGAACCTGAC TCGGACAGCC ACACCTGCCC CCAGCCAGAC

GCTTATTAGC TCAGCCACAT ACACACAGAT CCAGCCCCAT TCCCTGATTA

ACTTATTAGC TCAGCCACCT ACACACAGAT CCAGCCCCAT TCACTGATTA

GCTTATTAGC TCAGCCACAT ACACACAGAT CCAGCCCCAT TCACTGATTA

TCCACCTCCA GCAGAAGCAA GTGGTGATCC AGCAGCAGAT TGCCATCCAC

TCCACCTCCA GCAGAAACAG GTGGTGATCC AGCAGCAGAT TGCCATCCAC

TCCACCTCCA GCAGAAGCAA GTGGTGATCC AGCAGCAGAT TGCCATCCAC

CACCAG

CACCAG

CACCAG

>Ortholog Group 329, Repeat 2

3 192

R00020529 CAGTTCCAAC ACCGCCAGTC CCAGCTACTT CACACAGCCA CGCACCTCCA

H00323846 CAGTTCCAGC ACTGGCAGTC CCAGCTCCTT CACACAGCTA CACACCTCCA

M00125568 CAGTTCCAAC ACCGCCAGTC CCAGCTGCTT CACACAGCCA CACACCTCCA

GTTGGCCCAG CAGCAGCAGC AGCAGCAGCA ACAGCAGCAG CAGCAGCAAG

GTTGGCG--- ---------- ---------- ---------- ---CCGCAAG

GTTGGCC--- ---------- --------CA GCAGCAGCAG CAGCAGCAAG

CAACAACTCT CGCTGCCCCT CAGCCACCCC AGGTCCCACC TACTCAGCAG

CCACCACCCT CACTGCCCCT CAGCCACCAC AGGTCCCACC TACTCAGCAG

GAACAACCCT CACTGCCCCT CAGCCACCCC AGGTCCCACC TACTCAGCAG

GTCCCACCTT CCCAGTCGCA ACAGCAAGCC CAGACTCTGG TG

GTCCCACCTT CCCAGTCCCA GCAGCAAGCC CAAACCCTGG TC

GTCCCACCTT CCCAATCACA ACAGCAAGCC CAGACTCTGG TG

>Ortholog Group 32, Repeat 1

3 198

H00264731 CCAGTGAGGG GCCGTGAGAC TTATGAAATG CTGTTGAAGA TCAAAGAGTC

R00033463 CCAGTGAGAG GCCGTGAGAC TTATGAAATG CTGCTCAAGA TCAAGGAGTC

M00110965 CCAGTGAGAG GTCGTGAGAC GTACGAGATG TTGCTGAAGA TCAAAGAGTC

CCTGGAACTC ATGCAGTACC TTCCTCAGCA CACAATTGAA ACGTACAGGC

GCTCGAGCTC ATGCAGTATC TCCCTCAGCA CACGATCGAG ACGTACAGGC

ACTGGAGCTC ATGCAGTACC TCCCTCAGCA CACGATCGAA ACGTACAGGC

ACCAGCACTT ACTTCAGAAA CAGACCTCAA TACAGTCTCC ATCTTCATAT

ACCAACACCT ACTTCAGAAA CAGACCTCGA TGCAGTCTCA GTCTTCATAC

ACCAGCACCT ACTTCAGAAA CAGACCTCGA TGCAGTCTCA GTCTTCATAT

GGTAACAGCT CCCCACCTCT GAACAAAATG AACAGCATGA ACAAGCTG

GGTAACAGCT CACCACCTCT GAACAAAATG AACAGCATGA ACAAGCTG

GGCAACAGTT CCCCACCTCT GAACAAAATG AACAGCATGA ACAAGCTG

>Ortholog Group 330, Repeat 1

3 204

H00385169 GGAAGGATCT CCAGGCCAGG TATGGCACTA GGTGCCCCAG AAGTGTGCAA

R00031193 GGCAGGCTCT CCAGGCCAGG CATGACACTA GGTGCCCCAG AAGTGTGCAA

M00076303 GGCAGGCTCT CCAGGCCAGG CATGGCACTA GGTGCCCCAG AAGTGTGCAA

CCAGGTCACC TCATCCCAGT CTGTCCGGGG GCTTCTCCCT TGTACTGCC-

CCAGGTCACC TCACCCCAGT CTGTCCGGGG CCTTCTTCCT TGTACTGTCC

CCAGGTCACC TCACCCCAGT CTGTCCGGGG CCTTCTTCCT TGTACTGCCC

-----CTTCC TGCTCTCCCA CCCACGCCTC AGCAACAGCC ACCCTTGAAT

AGCAGCTTCC TGCCCTCCCA CCCGTTCCTC AACAACAGCC ACCCTTGAAT

AGCAGCTTCC TGCTCTCCCA CCCACTCCTC AACACCAGCC ACCCTTGAAT

AATCACATGA TCTCACAGCC AGTCCCGGCT CTGCAGCCCT CTCCGCAGCC

AATCACATGA TTTCACAGCC AGTCCCGGCC CTGCAGCCCT CTCCGCAGCC

AATCACATGA TTTCACAGCC AGTCCCGGCC CTGCAGCCCT CTCCACAGCC

TGTT

GGTT

GGTT

>Ortholog Group 331, Repeat 1

3 198

R00053317 CCCCTGACGC CCGAGCAGCA GGAGGCCCTC CAGAAGGCCA AGAAATATGC

M00098096 CCCCTGACGC CCGAGCAGCA GGAGGCCCTC CAGAAGGCCA AGAAATATGC

H00322016 CCCCTGACGC CCGAGCAGCA GGAGGCCCTT CAGAAGGCCA AGAAGTACGC

AATGGAGCAG AGCATCAAGA GTGTGCTGGT GAAGCAGACC ATCGCCCACC

AATGGAGCAG AGCATCAAGA GTGTGCTGGT GAAACAGACC ATCGCGCACC

CATGGAGCAG AGCATCAAGA GTGTGCTGGT GAAGCAGACC ATCGCGCACC

TCACCAACCT GCAGATGGCA GCAGTGACAA TGGGCTTTGG AGATCCTCTC

TCACCAACCT GCAGATGGCA GCAGTGACAA TGGGCTTTGG AGATCCTCTC

TCACCAACCT GCAGATGGCA GCAGTGACAA TGGGCTTTGG AGATCCTCTC

TCACCTTTGC AATCGATGGC AGCTCAGCGG CAGCGGGCAC TGGCTATC

TCACCTTTGC AATCGATGGC AGCTCAACGG CAGCGGGCAC TGGCTATC

TCACCTTTGC AATCGATGGC GGCTCAGCGG CAGCGGGCGC TGGCCATC

>Ortholog Group 332, Repeat 1

3 222

H00326391 AAGAACTACA TCAAATCGCT GACGGCCACC ATCCTGACCA TGTCCAGCAG

M00055493 AAGAACTATA TCAAGTCGCT GACCGCCACC ATACTTACTA TGTCCAGCAG

R00035219 AAGAACTATA TCAAGTCGCT GACAGCCACC ATACTTACTA TGTCCAGCAG

CCGCCTCCCA GGCCTGGAG- ---------- -GGGCCGGGC CCCAAGCTCT

CCGCCTCCCG GGGCTGGAGG CACCAGGTCC TGCGCCAGGC CCTAAATTAT

CCGCCTCCCG GGGCTGGAGG CGCCAGGTCC TGCGCCAGGC CCTAAATTGT

ACCAGCACTA C--------- ---------- --GTGGCTGG GGGTGCGTTG

ACCAGCACTA CCATCACCAG CAGCAGCAAC AGGTAGCTGG GGCCATGCTT

ACCAGCATTA CCATCACCAG CAGCAGCAAC AGGTGGCTGG GGCCGTGCTC

GGGGCCACGG AGGCCCAGCC CCAGGGCCAC CTGCAGAGGT ACTCCACGCA

GGTGTCACTG AGGACCAGCC CCAAGGCCAC CTGCAACGCT ACTCTACACA

GGTGTCACCG AGGACCAGCC TCAAGGCCAC CTGCAACGAT ACTCTACACA

GATCCACAGC TTCCGAGAGG GC

GATCCACAGC TTCAGAGAGG GG

GATCCACAGC TTCAGAGAGG GG

>Ortholog Group 333, Repeat 1

3 201

H00326671 CTGGCCTGTG TCTTCCTGGC AACTGCTGCC TTTGTTGCTT ATACTGCCCG

M00090710 ATAGTCTGTG TCTCCTTCCA AGCCTTCTCC TATTTAATCT ACATCCTGAG

R00054717 ATAGTCTGTG TCTTCTTCCA AGCCCTCTCC TATCTAATCT ACATCCTGAG

GCTGGACTGG AAGCTTGCTG CAGAGGAGGC TAAGAAACAT TCAGGC---C

GACAAACTGG AGCAGAGTCG CAGAGCAGGC ACAAGTTCGA GCTGGGCTAA

GACAAACTGG AACAGAGTCG CAGAGCAGGC ACAGGTTCGA GCTGGGCTAA

GGAGAGCAGA GAGCACTGCA ACCAGACCTG GGCCTGAGAA AGCAGTCCTA

AATTGATACC CACGCCAGCA GATCTGCCCA TCTTGGAAAG AGAAGTTATG

AAATGATTCC CACCCCAGCA GATCTGCCCA TCTTGGAGAG AGAAGTTATC

TCTTCAGTGG CTACAGGCAG TTCCCCTGGC ATTACCTTGA CAACGTATTC

GAT------- ---------- -------GGA GTGATTTTGC CTGATATCAT

GAC------- ---------- -------GGA GTGATTTTGC CTGATATCAT

A

C

C

>Ortholog Group 334, Repeat 1

3 198

H00386759 TCTAAAAAGA AAGTACGAAT TAAAGACCGC AATAAACTTT CTACAGAGGA

R00040274 TCTAAAAAGA AAGTACGAAT TAAAGACCGA AACAAACTTT CTACAGAGGA

M00116313 TCTAAAAAGA AAGTACGAAT TAAAGACCGA AACAAACTTT CTACAGAGGA

ACGCCGGAAG TTGTTTGAGC AAGAGGTGGC TCAACGGGAG GCTCAGAAAA

GCGCAGGAAG TTGTTTGAAC AAGAGGTAGC ACAAAGGGAG GCTCAGAAGA

GCGCAGGAAG TTGTTTGAAC AAGAGGTGGC ACAGAGGGAA GCTCAGAAGA

TGCAGAACCT GGGAATGACA TCACCACTGC CCTATGACTC TCTTGGTTAT

TGCAGAACTT GGGGATGACA TCACCACTAC CCTTTGACTC TCTGGGTTAT

TGCAGAACTT GGGGATGACA TCACCACTCC CCTTTGACTC TCTGGGATAT

AATGCCCCGC ATCATCCCTT TGCTGGTTAC CCACCAGGTT ATCCCATG

AATGCCTCTC ATCACCCCTT TGCTGGGTAC CCACCAGGTT ATCCCATG

AATGCCTCTC ATCACCCCTT TGCTGGGTAC CCACCAGGTT ACCCCATG

>Ortholog Group 335, Repeat 1

3 198

R00034376 CCGAAGCGGC CTGTGACCTG CGAGCAGGGC TGCGGCCTGG AGATGCCCAA

H00342755 CCGAAGCGGC CTGTGACCTG TGAACAGGGC TGTGGCCTGG AGATGCCCAA

M00100869 CCGAAGCGGC CTGTGACCTG TGAGCAGGGC TGTGGCCTGG AGATGCCCAA

AGATGAGCTG CCAAACCACA ATTGCATTAA GCACCTGCGC TCCGTGGTCA

AGATGAGCTG CCCAACCATA ACTGCATTAA GCACCTGCGC TCAGTGGTAA

AGATGAACTG CCAAACCACA ATTGCATTAA GCACCTGCGC TCCGTGGTCT

CGCGCATCGC AGAGCTGGAG AAGACATCTG CTGAACACAA GCACCAGCTG

CACGCATCGC AGAGCTGGAG AAGACGTCAG CTGAACACAA ACACCAGCTG

CGCGCATTGC AGAGCTGGAG AAGACCTCGG CTGAACACAA GCACCAGCTG

GCAGAACAGA AGCGAGACAT TCAGTTGCTG AAGGCGTATA TGCGAGCC

GCGGAGCAGA AGCGAGACAT CCAGCTGCTA AAGGCATACA TGCGTGCA

GCAGAGCAGA AGCGAGACAT TCAGCTGCTG AAGGCGTATA TGCGAGCC

>Ortholog Group 336, Repeat 1

3 198

M00037958 TTCAAAAACA TCATGAACAT CATTGATATC GTCTCCATTT TGCCTTACTT

R00006524 TTCAAAAACA TCATGAACAT CATTGATATC GTCTCCATTT TGCCTTACTT

H00328511 TTCAAAAACA TCATGAACAT CATTGACATT GTCTCCATTT TGCCTTACTT

CATCACTCTG GGCACTGACC TGGCCCAACA GCAGGGGGGT GGCAATGGCG

CATCACTCTG GGCACCGATC TGGCCCAGCA GCAGGGGGGT GGCAACGGCG

CATCACACTG GGCACTGACC TGGCCCAGCA ACAGGGGGGT GGCAATGGTG

CCATGTCCTT TGCCATCCTT AGGATCATTC GTCTGGTCCG AGTATTCCGG

CTATGTCCTT TGCCATCCTC AGGATCATCC GTCTGGTCCG AGTGTTCCGG

CCATGTCCTT TGCCATCCTC AGAATCATTC GTCTGGTCCG AGTATTCCGG

ATCTTCAAGC TCTCCAGACA CTCCAAAGGC CTGCAGATCC TGGGCCAC

ATCTTCAAGC TCTCCAGACA CTCCAAGGGC CTGCAGATCC TGGGCCAC

ATCTTCAAAC TCTCCAGGCA CTCCAAAGGC CTGCAGATCC TGGGCCAC

>Ortholog Group 337, Repeat 1

3 198

M00118839 ATTCAGAGCC TCTCTCCCAG CACCCTTGGG AAAGGAGAGT CTAGAGGCGT

R00017557 CTTCAG---- ---------- ---------- ---------- ----------

H00333367 ATTCAGAGCC CCTCTCCCAC CACCCTTGGG AAAGGAGAGT CTAGAGGCGT

TTTCTTACCA AGCCTGCTGA CCCCTGCACC ATGGCCCCAT GCTGCCCCTC

---------- ---------- ---------- ---------- ---------C

TTTCTTACCA GGCCTGCCGA CCCCTGCACC ATGGCTCGGT GCTGCTCCTG

CGCCGCCACC GCCGCAGCCA CAGCCACAGC CCCAGGCAGG CCCCAGACTC

CGCCACCACC TCCACAGCCA CAGCCACAGC CCCAGGCAGG CCCCAGGCTC

CACCGCCGCC TCCACAGCCA CAGCAGCAGC CACAGACAGG CCCTCGGCTC

CCCCCACGGC AGCCCACCGT GGCCTCCTCC GCGGAGTCCG ATGAGGAA

CCACCCCGGC AGCCCACCGT GGCCTCCTCT GCAGAGTCCG ATGAGGAA

CCCCCACGGC AACCCACGGT GGCCTCTCCA GCAGAGTCAG ATGAGGAA

>Ortholog Group 338, Repeat 1

3 198

R00042533 GAACCCAGCA GCCCAGATCA TGGCTCATCC GCCATTGAGC AAGACCTCGC

M00105743 GAACCCAGCA GCCCTGACCA CGGCTCATCA GCCATTGAGC AAGACCTCGC

H00338185 GAACCCAGCA GCCCTGATCA TGGTTCATCA ACGATTGAGC AAGACCTCGC

GGCCCTGGAT GCAGAAATGA CTCAGAAGTT GATAGACTTG AAAGACAAGC

TGCCCTGGAT GCAGAAATGA CTCAGAAGTT GATAGACTTG AAAGACAAGC

TGCTCTGGAT GCTGAAATGA CCCAAAAGTT AATAGACTTG AAGGACAAAC

TGCTTAATCT TCGGCAAGAG CAGTATTACA GTGAGAAGTA CCAGAAGCGG

TGCTTAATCT TCGGCAAGAG CAGTATTACA GCGAGAAGTA CCAAAAGCGA

TGCTTAATCT TCGGCAAGAA CAGTATTATA GTGAAAAATA CCAGAAGCGA

GAGCACATTA AATTGCTCAT TCAGAAGTTG ACAGATGTTG CTGAAGAG

GAACACATTA AACTGCTTAT TCAGAAGTTG ACAGATGTTG CAGAAGAG

GAACATATTA AACTGCTTAT TCAAAAGTTG ACGGATGTCG CAGAAGAG

>Ortholog Group 339, Repeat 1

3 204

R00002904 ACAGCCCTCA CTGCAGGGGA GAAGGCCATT GCAGGGACAG AGGAACGTAC

H00359485 AAAGCCCTCA CTGATAGAGA GAAGGCAGTA GCA------G TGGATCGGGC

M00072232 ACAGCCCTCA CTGCTGGGCA GAAGGCCATT GCA------G AGAAGCACAC

CCAGAGGGAG GCAGCCGAGA AGGAGCAGGA GCTGCTAAGA CAGAAGCAGA

CAAGAAGGAG GCAGCTGAGA AGGAACAGGA ACTTTTAAAA CAGAAATTAC

CAAGAAGGAG GCAGCTGAGA AGGAGCAGGA TCTGCTAAGA CAGAAGCAGA

GGGAGATGGA GGCTCAAGAG AAAAGTAACA AGGAAAACCT AGAGCAACTG

AGGAGATGGA GGCTCAAGAT AAGAGTCGCA AGGAAAACAT AGCCCAACTG

AGGAGATGGA GGCTCAAGAG AAAAGGAACA AGGAAAACCT AGAGCAACTG

AGAGAGAAGC TGGTGCAGGA GAGAAAGCAG CTCATCGAAG AACATAACAT

AAGGAGAAGC TGCAGATGGA GAGAGAACAC CTACTGAGAG AGCAGATTAT

AGAAGGAAGC TGGAGCAGGA GAGAGAGCAG CTCATCAAAG ACCATAACAT

GATG

GATG

GATG

>Ortholog Group 33, Repeat 1

3 198

R00034294 CCTGACATCC CTGGCTGGGT GGCTGAGGAG ATGGCCAAAT TGGAGTACCA

M00091157 CCTGACATCC CCGGCTGGGT AGCTGAGGAG ATGGCCAAAC TGGAGTACCA

H00159087 CCCGATATCC CGGGCTGGGT GGCCGAGGAA ATGGCCAAGC TGGAGTACCA

GCGACGGGAA GCCTTCAAGC GGCACGAACG GCAGGCGCAG CAGCGCTTT-

GCGACGGGAA GCTTTCAAGC GGCACGAGCG GCAGGCGCAG CAGCGCTTTC

GCGCCGCGAG GCCTTTAAGA GACACGAGCG CCAGGCCCAG CATCGCTACC

---------- ---------- ---------- ---------- ----------

GGCGGCGGCG CGAGGAGGAG GAGCGGCAGC GGCACGCGGA GCAGCAGGCG

GCAGGCGGCG GGAGGAGGAG GAGCGACAGC GCCATGCAGA GCACCATGCC

---------- ---------- ---------- ---------- --------

CGGCGGGAGC GCGACACGGG AGGCCGCGAG GAGGCGCGCG CGGAGGCG

CGGCGGGAGC ATGATTCTGG TGGCCGAGAG GAGGCGAGGG CCGAGGGC

>Ortholog Group 340, Repeat 1

3 201

R00053579 GCAGGAGGTA CTGGACTGCA ACCTCTGGCC AGCCCGAACC ACATCACCAC

M00083845 GCAGGAGGCA CTGGACTTCA ACCTCTGGCC AGCCCAAACC ACATCACCAC

H00333602 GCGGGTGGGG CCGGCCTGCA GCCCCTGGCC AGCCCAAGCC ACATCACCAC

GGCCAGTCTG CCACCCCAGA TCAGCAGCAT TATTCAGGGT CAGTTGATAC

GGCCAGCCTG CCACCCCAGA TCAGCAGCAT TATTCAGGGC CAGTTGATAC

GGCTAACTTG CCACCGCAGA TCAGCAGCAT CATCCAGGGC CAGCTGGTT-

AGGTGCTTCA GGGGCAGCCA ATGAACAGAT CTCTGGGATT TGAGAGGACA

AGGTGCTTCA GGGACAGCCA ATGAACCGAT CTCTGGGATT TGAGAGGACA

--GTGCTGCA GGGGCCGCCG CTGCCCCGGC CCCTGGGCTT CGAGAGGACA

CCTGGCGTGT TGCTCCCTGG CGTAGGAGGA CCTTCAGCAT TTGGGATGAC

CCTGGTGTGC TGCTTCCTGG CGTGGGAGGG CCTTCAGCAT TTGGGATGAC

CCCGGCGTGC TGCTCCCCGG GGCTGGGGGC GCAGCGGGGT TTGGGATGAC

A

A

G

>Ortholog Group 340, Repeat 2

3 201

R00053579 CAGACCCCAA CCCGCTCTTT AGTAACCCAG GTGTCTCAAG CCACAGGGGT

M00083845 CAGACTCCAA CCCGTTCCTT AGTAACCCAG GTGTCTCAAG CCACAGGGGT

H00333602 CAGACCCCGG CACGGTCTTT GGTGCCCCAA GTGTCCCAAG CCACAGGAGT

TCAGCTACCC GGAAAAACCA TCACCCCTGC TGCCCATTTC CAGCTTCTTA

TCAGCTACCT GGAAAAACCA TCACCCCTGC TGCTCATTTC CAGCTTCTTA

TCAGCTCCCT GGAAAAACCA TCACACCT-- -GCACATTTC CAGCTTCTCA

GG-------- -ACTTCCCAG GTGCAAGTTC CACAGCTCCA GGGCCAGGCC

GG-------- -ACTTCCCAG GTGCAAGTTC CACAGCTCCA GAGCCAGGCC

GGACGACGAC GACCTCTCAG GTGCAAGTTC CACAGATCCA GGGCCAGGCC

CAGTCTCCTG CACAGATGAA AGCTGTGGGC AAGCTAGCAC CGGAACACAT

CAATCTCCTG CACAGATCAA AGCTGTGAGC AAGCTAGGAC CGGAACACAT

CAGTCCCCAG CACAGATCAA AGCTGTGGGC AAGCTGACGC CGGAACACCT

C

C

C

>Ortholog Group 341, Repeat 1

3 198

M00071715 GCTGCTGACA CCGTGAAGGA GCTCATGTGC CCTGAAGCCC AGCTGCCCCG

R00029108 GCCGCTGACA CCGTGAAGGA GCTCATGTGC CCTGAAGCCC AGCTGCCCCA

H00354451 GCGGCTGACA CTGTGAAGGA GCTGATGTGC CCTGAGGCCC AGCTGCCTCC

AGTCTACCCC TTTGCCTCTG CTTTTTACCA GCAAGAGCTG GCTCTCCTCG

AGTATACCCC TTTGCCTCTG CTGTGTACCA GCAGGAGCTG GCTCTCCTCG

AGTGTACCCT GTTGCATCGT CTATGTACCA GCTGGAGCTG GCAGTGCTCG

GGGAGCTGGG CCAGGAGGAG CTCTTTGTGG CTGTGGAGAT GCTGTCAGCC

GGGAACTGGA CCAGGAGGAG CTCTTTGTGG CTGTGGAGAT GCTCTCAGCC

GGGAGCTTGG CCAGGAGGAG CTCTTCGTGG CTGTGGAGAT GCTCTCAGCT

GTGGTTCTGA TTAACCGTGC TCTGGAAGCT GGAGATGTCT GTGCCTTT

GTGGTTCTGA TTAACCGTGC TCTGGAAGCT GGAGATGCCT GTACCTTT

GTGGTCCTGA TTAACCGGGC CCTGGAGGCC CGGGATGCCA GTGGCTTC

>Ortholog Group 342, Repeat 1

3 198

R00003565 ATGGGCGTCC AGGGCTTCCA AGAGTTCCTG GAGAAGCGCT GTCCCGGGGC

M00073082 ATGGGCGTCC AGGGCTTCCA AGAGTTCCTG GAGAAGCGCT GTCCCGGGGC

H00364324 ATGGGTGTCC AGGGCTTCCA AGAGTTCCTG GAGAAGCGCT GTCCCGGGGC

TGTGGTGCCT GTGGACCTCC TCAAACTCGC GCGCACCGTC TCGCGCCACC

TGTGGTGCCT GTGGACCTCC TCAAACTCGC GCGCACCGTC TCGCGCCACC

CGTGGTGCCC GTGGACCTCC TAAAACTCGC GCGCACGGTC TCGCGCCACT

TGCACCGCCA GCTGCCGCCA ---GCAGCCC TAGCGCCAGG GGCTCCACGC

TGCACCGCCA GCTGCCGCCA ---GCAGCCC TAGCGCCAGG GGCTCCACGC

TGCACCGCCA GCTGCCGCCG ACTGCAGCCC TAGCGCCCGG GGCTCCACGC

ATCGCCAGGG GCTCTGCTCC TCTG---CCG CCGCCGCTCC CGCCTGCT

ATCACCAGGG GCTCTGCTCC TCTGCCGCCG CCGCCGCTCC CGCCCGCT

GCCGCCAGGG GCTCCGTGCC TCTG---CAA CCGCCGCTTC CGCCCGCT

>Ortholog Group 343, Repeat 1

3 9

H00332163 ATGTCTTCC

M00051559 ATGCCTTCC

R00060066 ATGCCTTCC

>Ortholog Group 343, Repeat 2

3 9

H00332163 TGCCCACCC

M00051559 TGCACACCA

R00060066 TGCACACCA

>Ortholog Group 343, Repeat 3

3 108

H00332163 CAGAGGGCCG TGAAGCAGCC TTGTCAGCCA CCCCCTGTTA AATGTCAAGA

M00051559 CAA---GCCG TGAAGCAGCC CTGTCAGCCA CCTCCTACCA AATGTCAAGA

R00060066 CAA---GCCG TGAAGCAACC CTGTCAGCCA CCTCCTACCA AATGTCAAGA

GACATGTGCA CCCAAAACCA AGGATCCATG TGCTCCCCAG GTCAAGAAGC

GGCATGTGTG CCTAAAACCA AGGATCCATG TGTTCCTCAG GCCAAAAAAC

GGCATGTGTG CCCAAAACCA AGGATCCATG TGTTCCTCAG GCCAAAAAAC

AATGCCCA

AATGCCCA

AATGTCCA

>Ortholog Group 344, Repeat 1

3 204

H00371785 GCGCGCGAGA AGCCGGAGGC GCCG---GCC GAGCCTCCAG GCCCCGGGCC

R00002827 GCGCGTGAGA AGCCTGAAGC TCCGCTCGCT GAGCCTCGGG GACCAGCGCC

M00061408 GCGCGCGAGA AGGCCGAAGC TCCGCTCGCT GAACCTCGGG GACCTGCGCC

CGGGTCAGGC GCGCACCCGG GCGGCAGCGC CCGGCCGGAC GCCAAGGAGG

GGAGTCC--- ---------- --GGGGGCGC CCGGGCCGAC GCCAAAGAGG

AGAGTCC--- ---------- --GGGGGCGC CCGGGCCGAC GCCAAAGAGG

AG---CTGCG GCGCAAGATC AACAGCCGCG AGCGGAAGCG CATGCAGGAC

AGCAGCTGAG GCGCAAGATC AACAGCCGCG AGCGGAAGCG CATGCAGGAC

AACAGCTGCG GCGCAAGATC AACAGCCGCG AGCGGAAGCG CATGCAGGAC

CTGAACCTGG CCATGGACGC CCTGCGCGAG GTCATCCTGC CCTACTCAGC

CTGAACTTGG CCATGGACGC GCTGCGCGAA GTTATCCTAC CCTACTCCGC

CTGAACTTGG CCATGGACGC GCTGCGCGAA GTTATCCTAC CCTACTCGGC

GGCG

GGCG

AGCG

>Ortholog Group 345, Repeat 1

3 105

R00007745 ---------- ---------- ---------- ---------- ----------

H00412394 AACACCAAGC CTTTGTTTCA TTTTAACTCA GATCAAGCGA ACCAGCAGAT

M00124083 AATACCAAGC CTTTGTTCCA TTTTAACTCA GACCAAGCAA ACCAGCAGAT

---------- ---------- ---------- ---------- ----------

GCCTTCTGTT TTGCCTTCCC AGAACAAGCC TTCTCTCCTA CACTACACCA

GCCTTCTCTT TTGCCTTCCC AGAGCAAACC TTCTCTCTTG CACTATACCC

-----

GTTCA

AACAA

>Ortholog Group 345, Repeat 2

3 12

R00007745 ---------- --

H00412394 TCAGCTAGCT CA

M00124083 CAGCAGGGCT CC

>Ortholog Group 345, Repeat 3

3 105

R00007745 ---------- ---------- ---------- ---------- ----------

H00412394 TCAGCCCCAT CTTCTCAGCC TGCCCAATCT CTACCAAGCC AGCCTTTGCT

M00124083 GCAGCTCAAC CATCGCAACC CACCCATGCT TTATCAAGCC AGCCTTTGCT

---------- ---------- ---------- ---------- ----------

AAGGTCACCT TTGCCACTTC AGCAAAAGCT CCTACTTCAG CAAATGCAGA

AAGGTCACCC TTACAACTTC AGCAAAAGAT CATGCTTCAG AAAATGCAAA

-----

ATCAG

CTCAG

>Ortholog Group 346, Repeat 1

3 204

M00063809 AGCCCCCTAG GCCAGCCGGA CATCAAGCCC TCGGTGGTGG TACAGCAGGG

R00009043 AGCCCCCTAG GCCAGCCGGA CATCAAGCCC TCGGTGGTGG TACAGCAGGG

H00329170 AGCCCCCTGG GCCAGCCGGA CATCAAGCCC TCGGTGGTGG TGCAGCAGGG

TGGCCGAGGC GACGAGCTGC ACGGGCCAGG AGCGCTGCAG CAACAGCATC

TGGCCGAGGC GACGAGCTGC ACGGGCCAGG AGCGCTGCAG CAGCAGCACC

CGGCCGCGGA GACGAGCTGC ACGGGCCAGG CGCCCTGCAG CAGCAGCAT-

AACAGCGACC GCCACATCTG GTGCACCACG CTGCCAACCA CCATCCCGGG

AACAGCGACC GCCACATCTG GTGCACCACG CTGCCAACCA CCATCCCGGG

-----CGGCC GCCGCATCTG GTGCACCACG CCGCTAACCA CCACCCGGGA

CCCGGGGCAT GGCGGAGTGC GGCGGCTGCA GCTCACCTCC CTCCCTCCAT

CCCGGGGCAT GGCGGAGTGC GGCGGCTGCA GCTCACCTTC CTCCCTCCAT

CCCGGGGCAT GGCGGAGCGC GGCGGCTGCA GCGCACCTCC CACCCTCCAT

GGGA

GGGA

GGGA

>Ortholog Group 347, Repeat 1

3 126

H00333125 CACCAGACAC TGCAGCAGAC ACCCATGATA AGTACCATGA CTCCAATGAG

R00048013 CACCAGACAC TGCAGCAGAC ACCAATGATG GGTACCATGA CTCCGTTGAG

M00085260 CACCAGACAC TGCAGCAGAC ACCCATGATG GGTACCATGA CTCCGTTGAG

TGCCCAGGGC GTCCAGGCAG GCGTCCGTTC AACAGCCATC CTACCTGAG-

TGCCCAGGGT GTCCAGGCAG GCGTCCGTTC AACTTCCATC CTGCCTGAG-

TGCCCAGGGT GTCCAGGCAG GCGTCCGTTC AACTTCCATC CTGCCTGAGC

---------- ---------- TACCAC

--CAGCAACA ACAGCAACAA TACCAC

AGCAGCAACA ACAACAGCAG TACCAT

>Ortholog Group 347, Repeat 2

3 12

H00333125 ATCCGG---A TC

R00048013 ATCCGACAGA TC

M00085260 ATCCGACAGA TG

>Ortholog Group 347, Repeat 3

3 3

H00333125 CGG

R00048013 AGG

M00085260 CGG

>Ortholog Group 347, Repeat 4

3 33

H00333125 GCGGCTCCTC CCCAACCCCA GCCCCAGTCC CAG

R00048013 GCAGCACCTC CCCAACCTCA ACCCCAGTCC CAG

M00085260 GCAGCTCCTC CCCAACCCCA GCCCCAGTCC CAG

>Ortholog Group 347, Repeat 5

3 111

H00333125 CCCCAGTTCC AGCGCCAGGG GCTTCAGCAG ACCACAGCAG CTTTGGTCCG

R00048013 CCCCAGTTCC AGCGCCAGGG GCTTCAGCAG ACCACAGCAG CTTTGGTCCG

M00085260 CCCCAGTTCC AGCGCCAGGG CCTGCAGCAG ACCACAGCAG CTTTGGTCCG

GCAACTTCAA CAACAGCTCT CTAATACCCA GCCACAGCCC AGTACCAACA

GCAACTTCAA CAACAGCTCT CTAATACCCA GCCACAGCCC AGTACCAACA

GCAACTTCAA CAACAGCTCT CTAATACCCA GCCACAGCCC AGCACCAACA

TATTTGGACG C

TATTTGGACG C

TATTTGGACG C

>Ortholog Group 348, Repeat 1

3 198

M00110534 GAGCAGAAGC GCCTGAGAGA AGAAATCCTG AAGCAGAAGG AACTACGAAG

H00384160 GAACAGAAAC GCCTAAGAGA AGAAATCCTG AAACAGAAGG AGTTACGGCG

R00030808 GAGCAGAAGC GACTGAGAGA AGAAATCCTA AAGCAGAAGG AGCTACGAAG

GCAACAGCAG GCTGGTGCCA GAAAGAAGGA GTTACTGGAA AGGCTTGCGA

GCAGCAGCAG GCTGGTGCCA GGAAGAAGGA GCTGCTGGAG AGACTCGCGC

GCAGCAGCAG GCTGGTGCCA GAAAGAAGGA GTTACTGGAA AGACTTGCAA

TCTATGGCTC GCAGACCTCC ATGGAACAAG AGGAACTGGC AGCTACACCA

TGTACGCTCC CCCACCCCCA GCAGAGCAGG AAGAGCAGGC ACTGTCACCA

TCTATGGCTC TCAGACCCCC ATGGAGCAAG AGGAACTGGC AGCTACACCA

TCACCTACCA ATGGTAACCC ATTGTTGCCC TTTCCAGGGG CACAATGC

TCACCCACCA ACGGTAACCC ACTGTTGCCC TTTCCAGGTG CACAGGTC

TCCCCCACCA ATGGTAACCC ACTGTTGCCC TTTCCAGGGA CACAATGC

>Ortholog Group 349, Repeat 1

3 198

R00044643 TGTTTCCGGG AGGAGCTCAT GGAGGCAGTG CACCTGCACC TGGTAAAGGA

M00021710 TGTTTCCGGG AGGAGCTCAT GGAGACTGTG CACCTGCACC TAGTGAAAGA

H00332326 TGTTTCCGGG AGGAGCTCAT GGAGGCCTTG CACCTGCACC TGGTGAAGGA

ATACATCATC CGGCTCAGCA AACGGCGCCT GGTCCTCAAG ACCGCAGAGC

ATACATCATC AGGCTCTGCA AACGGCGCCT GGTCCTCAAG ACGGCCGAGC

GTACATCATC CAACTCAGCA AGGGGCGCCT GGTCCTCAAG ACGGCCGAGC

TGGCAAAGCA CATCCTCGCC AATGCGGACA CCATTCAGCA CTTCTGCACT

TGGCAAGGCA CATCCTGGCC AACGCGGATG CCATTCAGGG GTTCTGCACT

TGGCTGGGTA CATCCTGGCC AATGCTGACA CCATCCAGCA CTTCTGCACC

GAGAATGGCT CCACTGCAAC CTGGCTGCAC GCTGCCCTCC CTACGATC

GAGAATGGCT CCACTGCAAC CTGGCTGCAC CGCGCCCTCC CTATGATT

CAGCACGGCT CCCCGGCGAC CTGGCTGCAG CCTGCTCTCC CTACGCTG

>Ortholog Group 34, Repeat 1

3 198

R00061740 TTGATCACAG TATCCGGAGA CAGCTGTGTG TTCATCTGGC ACCTGGGCCC

H00384792 TTGATCACAG TATCTGGAGA CAGCTGCGTG TTCATCTGGC ACCTGGGCCC

M00103825 TTGATCACAG TATCTGGAGA CAGCTGTGTG TTCATCTGGC ACCTGGGCCC

CGAGATCACC ACCTGCATGA AGCAGCACTT GATGGAGATC AACCACCAGC

GGAGATCACC AACTGCATGA AGCAGCACTT GCTGGAGATT GACCACCGGC

GGAGATCACC ACCTGCATGA AGCAGCACTT GCTGGAGATC AACCACCAGC

TGCCCAAGGA CCAGAAGTGG AGTGGCCCTC CCAGCCAGGA GACCTCTGCA

ACACAAATGA CAAGAAGCGG AGTGGCCACC CCAGGCAGGA TACGTATGTG

AGCCCAAGGA CCAGAAGTGG AGTGGCCCTC CCAGCCAGGA GACATATGCA

TCCACCCCAA GTGAGATCCG TTCCCTGAGC CCTGGGGAGC AGACAGAG

TCCACACCTA GTGAGATTCA CTCCCTGAGC CCTGGAGAGC AAACAGAG

TCCACACCAA GTGAGATTCG TTCCCTGAGC CCTGGAGAGC AGACAGAG

>Ortholog Group 350, Repeat 1

3 198

R00014152 CACACGCCAC AGACGCCGGG CCAAGGGGGC CCGGCCAGCA CGCCGGCCCA

H00329908 CACACGCCAC AGACGCCCGG CCAAGGGGGC CCAGCCAGCA CGCCAGCCCA

M00032768 CACACGCCAC AGACGCCGGG CCAAGGGGGC CCGGCCAGCA CGCCGGCCCA

GACAGCGGCT GGCAGCCAGG GCGGCCCTGG CGGCCCAGGC AGCGACAAAC

GACGGCGGCC GGTGGCCAGG GCGGCCCTGG CGGCCCGGGT AGCGACAAGC

GACAGCGGCT GGCGGCCAGG GCGGCCCTGG CGGCCCGGGC AGCGACAAGC

ACATCGAGTG CGTGGTGTGC GGGGACAAGT CGAGCGGCAA GCACTACGGC

ACATCGAGTG CGTGGTGTGC GGAGACAAGT CGAGCGGCAA GCACTACGGC

ACATCGAGTG CGTGGTGTGC GGGGACAAGT CGAGCGGCAA GCACTACGGC

CAGTTCACGT GCGAGGGCTG CAAGAGCTTC TTCAAGCGCA GCGTGCGG

CAGTTCACGT GCGAGGGCTG CAAGAGCTTC TTCAAGCGCA GCGTGCGG

CAGTTCACGT GCGAGGGCTG CAAGAGCTTC TTCAAGCGCA GCGTGCGG

>Ortholog Group 351, Repeat 1

3 198

R00019403 CTGCAAGGGT CTGATTCTCT AAACATCCAG CAGAACCAGA CATCAGGAGG

M00001326 CTACAAGGGT CTGATTCTCT GAACATCCAG CAGAACCAGA CATCAGGAGG

H00329357 CTACAGGGGT CTGATGCTCT GAACATCCAG CAAAACCAGA CATCTGGAGG

ATCACTGCAA GGAAGTCAGC AAAAAGAGGG AGAGCAAAGT CAGCAGACAA

CTCGCTGCAA GGAAGTCAGC AGAAAGAGGG AGAGCAAAGT CAGCAGACAA

CTCATTGCAA GCAGGCCAGC AAAAAGAAGG AGAGCAAAAC CAGCAGACAA

TTCTTATTCA GCCTCAGCTA GTTCAAGGTG GACAAGCTCT TCAGGCCCTC

TCCTTATTCA GCCTCAGCTA GTTCAAGGAG GACAAGCTCT TCAGGCCCTT

TTCTTATCCA GCCTCAGCTA GTTCAAGGGG GACAGGCCCT CCAGGCCCTC

CAAGCAGCAC CATTGTCAGG ACAGACCTTC ACAACTCAAG CTATTTCC

CAAGCAGCAC CATTGTCCGG ACAGACCTTC ACAACTCAAG CTATTTCC

CAAGCAGCAC CATTGTCAGG GCAGACCTTT ACAACTCAAG CCATCTCC

>Ortholog Group 352, Repeat 1

3 204

R00002995 GGCAGCCCGA CGTCCCCACC GGCTCTCCCG CCCCACCCGG CTCCCTCCAG

M00055267 GGCAGCCCGA CGTCCCCGCC TGCTCTCCCG CCCCACCCGG CGCCCTCCAG

H00334879 ACAAGCAGGG CTTCCCCGCC TGCTCTCCTG CCCGGCCCCG CTCCCCGCGG

AGAGCCGCTG GAGCTGTTTA CCGCCGGCTT CCCGAACCAT TCAACCCAGC

AGAGCCACTG GAGCTGTTTA CCGCCGGCTC CCCGAACCAT TCAACCCAGC

AGACCGGCCG GAGCTGCTGA CCCCCAGCTC CCTGCATTAT TCGACC----

AGCTGCGCAC TCGAGAGTGG GTAGCCAGAT ACCCGCTGGC GCCTGAGGCC

AGCTGCGCAC TCAAGAGTGG GTAGCCAGAT ACCTGCAGGT GCCTGAGCCC

--CTGCGCAC TCGAGAGTGG GTGGCCAGGC ACCCGCAGGT GCCCGAGGCC

GGGATCCAGG GGCCCATTCG CGCCTGGTCG GTGTTGCCAG ACAACTTCCT

GAGATCCAGG CGCCCACTCG CGCCTGGTCG GTGTTGCCAG ACAACTTCCT

CGTGATCAGG GCCCTATCCG CGCCTGGTCG GTGCTGCCAG ACAACTTCCT

CCAG

CCAG

CCAG

>Ortholog Group 353, Repeat 1

3 213

H00345841 CCACGGGTAA AGAAGTTAAA GTACCACCAA TACATTCCAC CAGATCAGAA

M00009713 CCGCGAGTGA AGAAGCTTAA GTACCACCAG TACATTCCAC CCAACCAGAA

R00059539 ---------- ---------- ---------- ---------- ----------

GGGTGAGAAG AATGAGCCGC AGATGGACTC TAACTACGCC CGCCTGCTCC

GGGGGAGAAG AGCGAGCCAC AGATGGACTC CAACTATGCC CGCCTGCTCC

---------- ---------- ---------- ---------- ----------

TGTTCCTGCA ACTGCAGATC CTGAGT---- ---------- -CAGCAGAAG

TCTTCCTACA GCTGCAGATC CTGAGCCAGC AGCAGCAGCA ACAGCAGCAG

---------- ---------- ---------- ---------- ----------

CAGCACTACA ACTACCAGAC CATCCTGCCT GCACCATTCA AGCCACTCAA

CAGCACTACA ACTACCAGAC CATCCTACCT GCACCCATCA AA------AC

---------- ---------- ---------- ---------- ----------

TGACAAAAAT AGT

TGACAAGAAC AGC

---------- ---

>Ortholog Group 354, Repeat 1

3 198

R00008637 GCAATGGACT CTGTTCCTAC CTTAACCACA GCGTCTGAGG TGTTTTCTGA

M00030691 ACAATGGACT CTGTTCCTAC CTTAACCACC GCCTCTGAGG TGTTTTCTGA

H00334319 ACGATAGACT CTGTTCCCAC TTTAACCACA GCATCCGAAG TGTTTCCTGA

GGTGGTCGGA GAGGAAAGCA CCCTTCTAAT TGTCCCTGAT GAAGACAAAC

GGTGGTTGGA GAGGAAAGCA CTCTTTTGAT TGTCCCTGAT GAAGACAAAC

AGTGGTGGGA GATGAAAGTG CTCTTTTAAT TGTCCCTGAA GAAGATAAAC

TTGACTTGGA GCGAGAGCTC CTGGAACTGG AGAAAATTAA GCAACAGCGC

TTGACTTGGA GCGAGAGCTC CTGGAACTGG AGAAAATTAA GCAACAACGC

TAGACTTGGA GCGTGAGCTC CTGGAACTGG AGAAAATTAA GCAACAGCGC

TTTGCTGAAG AACTGGAGTG GGAACGTCAG GAGATTCAGA GGTTCCGA

TTTGCTGAGG AACTGGAGTG GGAACGTCAG GAGATTCAAA GGTTCCGA

TTTGCTGAGG AATTGGAGTG GGAACGTCAG GAAATTCAAA GGTTCCGA

>Ortholog Group 355, Repeat 1

3 198

H00366070 GTGATGAAGA AGCTGAAGGA GGTGGTGGAC AAACAACGCG ACGAGATCCG

R00001401 GTGATGAAGA GGCTGAAGGA GGTGGTGGAC AAGCAGAGGG ATGAGATCCG

M00050014 GTGATGAAGA GGCTGAAGGA GGTGGTGGAC AAGCAGAGGG ATGAGCTCCG

CGCCAAGGAC AGGGAGCTGG GCCTGAAAAA TGAGGACGTT GAGGCTTTAA

AGCCAAAGAC AGGGAGCTGG TCCTGAAGAA TGAGGACGTC GAGGCTCTGA

AGCCAAAGAC AGGGAGCTGG GCCTGAAGAA TGAGGACGTC GAGGCTTTGA

CACGGCTGAT GAAGATCAAC CATGACCTTC GGCACCGGGT CACGGTGGTG

CGAGGCTAAT GAAGATCAAC CACGACCTTC GGCACAGGGT CACCGTGGTG

CGAGGCTAAT GAAGATCAAC CATGACCTTC GGCACCGGGT CACCGTGGTT

GAGGCCCAGG GGAAAGCCCT GATCGAACAG AAGGTGGAGC TGGAGGCA

GAGGCCCAGG GGAAGGCTCT GATTGAGCAG AAGGTGGAAC TTGAGGCA

GAGGCCCAGG GGAAAGCCCT GATTGAGCAG AAGGTGGAGC TCGAAGCA

>Ortholog Group 356, Repeat 1

3 204

M00100613 GGCGGCATCA AGCTGCACAA GAACCTCCTG GTGTCCTACG TGCTCCGCAA

H00361569 GGCGGCATCA AGCTGCACAA GAACCTCCTG GTGTCCTACG TGCTCCGCAA

R00060846 GGCGGCATCA AGCTGCACAA GAACCTCCTG GTGTCCTACG TGCTCCGCAA

CGCGCGCCAG CTCTACCTGA GCGAGCGTTA CGCCGAGCTC TACCGGCGC-

CGCGCGCCAG CTCTACCTGA GCGAGCGCTA CGCCGAGCTC TACCGGCGC-

CGCGCGCCAG CTCTACCTGA GCGAGCGCTA CGCCGAGCTC TACCGGCGCC

-----CCGCC CCACCACCAG CACCAGCACC TCGCTTACGC GGCGCCCGGA

-----CCGCC CCACCACCAG CACCAGCACC TAGCGTACGC GGCGCCGGGC

AGCAGCCGCC CCACCACCAG CACCAGCACC TCGCTTACGC GGCGCCCGGA

ATGCCGGCCA GCGCGGCCGA CTTCGGCCCG CTCCAACTTG GCGGCGGCGG

ATGCCGGCCA GCGCGGCCGA CTTCGGCCCG CTCCAACTTG GCGGCGGCGG

ATGCCGGCCA GCGCGGCCGA CTTCGGCCCG CTCCAACTTG GTGGCGGCGG

GGAC

GGAC

GGAC

>Ortholog Group 357, Repeat 1

3 312

R00059732 AAGGCAAGAA ATACTGTCCG TCAAAAAGCG ACTGAAGTGG AGGAGTTCCA

H00202677 AAGGCAAGAA ATATTGTTCG CCAAAAAGCA ACTGAAGTGG AGGAGTGTCA

M00043364 ---------- ---------- ---------- ---------- ----------

ACAGGCAGAG AGCACGGCTG CAGCCGACTG TGACTATCTT GTGGTAGGAG

ACAGTCAGAA AATGCACCTG CAGCCGGATC TGGCCATCTC ACAGTGGGAG

---------- ---------- ---------- ---------- ----------

TCCCCCGGAG CAGCAGCACC TCTGACATCA CTGAGCGCTT GTACTCAGAT

TCCTTCGGAG CAGCAGCACC TCCGACATCC CCGAGCCGCT GTGCTCAGAT

---------- ---------- ---------- ---------- ----------

TCTTCTCAAG GTTACAGTCC CATGCTGCCT CATCCAGCTT TTTACATGGC

TCTTCTCAG- ---------- ---------- ---------- ----------

---------- ---------- ---------- ---------- ----------

TTCTGGGGAT CTAAACTCAG GTCCTCATGC CTGCACAACA GGTCCTTTAC

---------- ---------- ---------- ---------- ----------

---------- ---------- ---------- ---------- ----------

CACTGAGCCA TCTCCCCAGC CCAGGTCAAA AGGTAGAAAA TTCACAGAAT

---------- ---------- ---GGTCAAA AGGCAGAAAA CACACAGAAT

---------- ---------- ---------- ---------- ----------

TTGAGCTCTT CA

TCGAGTTCTT CA

---------- --

>Ortholog Group 358, Repeat 1

3 198

H00374221 ATGAGCAAGG TGGCCTCCGA GAAGTACGAG GAGGGGGAGC GGGCATTGCG

M00099320 ATGAGTAAGG TAGCCTCCAA GAAATACGAG GAGGGGGAGC AGGCCTTGCA

R00011560 ATGAGTAAGG TAGCCTCCAA GAAGTATGAG GAGGGTGAGC AGGCCTTGCA

CGAGGCCCAG CAGGTGCAGG CAGAGCAGCA GGCCCGGTTG CAGGCGGTGG

GGAGGCGCAG CAGATGCAGA ATGAGCAGCA GGGCCGGCTG CAGGTGGTCG

GGAGGCCCGG CAGATGCAGA CTGAGCAGCA GGGCCGGCTG CAGGTGGTCG

AGCGGCTGCG GAAGCAGGAG CAGCACATGC ACCAGGAGCA TCTGAGTCTG

AGTGGCTGCG GCAGCAGGAG CAACGTGTGC ACCAGGAGCA CCTGAGCCTG

AGTGGCTGCG GCAGCAGGAG CAGCGTGTGC ATCAGGAGCA CCTGAGCCTG

GCCCAGCAGA GGCTGCAACT GGACCGCGCA CGACAGGACC TGCCCTCT

GCACAGCAGA GGCTGCAGCT GGACCGCGTG CGGCAGGAAG TGCCTGCT

GCCCAGCAGC GGCTGCAGCT GGACCGCGTG CGGCAGGAAG CACCTTCC

>Ortholog Group 359, Repeat 1

3 198

H00381543 ATGAGGAAGA GCCCTCTCGG GGGTGGTGGC GGCTCGGGAG CCTCCAGTCA

R00052197 ATAAGGAAGA GCCCTCTCGG GGGTGGCGGC GGCTCGGGAG CCTCCAGTCA

M00042792 ATAAGGAAGA GCCCTCTCGG GGGTGGCGGC GGCTCGGGAG CCTCCAGTCA

GGCCGCCTGC CTCAAACAGA TCCTTCTGCT GCAATTGGAC CTCATCGAAC

GGCCGCCTGC CTCAAACAGA TCCTTCTGCT GCAATTGGAC CTCATCGAAT

GGCCGCCTGC CTCAAACAGA TCCTTCTGCT GCAATTGGAC CTCATCGAAT

TGCAGGCCAA GGAAAAGGAG ATCGAGGAGC TGAAGTCAGA GAGAGACACG

TGCAAGCCAA GGAAAAGGAG ATCGAGGAGC TGAAGTCGGA GAGAGATACG

TGCAGGCCAA GGAGAAGGAG ATAGAGGAGC TGAAGTCCGA GAGAGATACG

CTCCTTGCTC GGATTGAACG TATGGAAAGG CGGATGCAGC TGGTAAAG

CTCCTTGCTC GGATTGAACG TATGGAAAGG CGGATGCAGC TGGTAAAG

CTCCTTGCTC GGATTGAACG TATGGAAAGG CGGATGCAGC TGGTGAAG

>Ortholog Group 35, Repeat 1

3 198

M00045286 AAGGATGAAT TGCTGTGTCA ACTTACCCAG AGCAACAAGG AGCAGGCTAC

R00015651 AAGGATGAAC TGCTGTGTCA ACTTACCCAG AGCAACAAGG AACAGGCTGC

H00323300 AAGGATGAGT TACTCTGCCA GCTTACCCAG AGCAATGAGG AGCAGGCTGC

TCAATGGCAA AAGGAAGAAA TGGAACTGAA ACACATACAA GCAGAACTGG

TCAGTGGCAA AAGGAAGAAA CGGAACTGAA ACACAGACAA GCAGAACTGG

TCAATGGCAA AAGGAAGAGA TGGCACTAAA ACACATGCAG GCAGAACTGG

CTGTGTTGGC TAAGGAGGTC CAGGACCTGA GGGAGACTGT GGAGTTTATA

CTGTGTTGGC TAAGGAGGTA CAGGACCTGA GGGAGACCAT GGAGTTTGTA

CTGTCCTGGC CAAAGAGGTG CGGGACCTGA AAGAGACCTT GGAGTTTGCA

GATGAAGAAA GTCAAGTTGC TCACCGGGAG CTGGGCCAGA TTGAGAGT

GATGAAGAGA GTCAAGTTGC TCACCTGGAG CTGGGCCAGA TCGAGAGT

GACCAGGAGA ATCAGGTTGC TCACCTGGAG CTGGGTCAGG TTGAGTGT

>Ortholog Group 360, Repeat 1

3 198

M00052204 CCTACTAATA ACCAAGTCCC TACTGCCATG TCATCTTCTT CCACCCTTCA

R00006970 GCTACTAGTA ACCAAGTCCC TGCGGCCATG TCATCTTCTT CCACCCTTCA

H00335044 GCTACTAATA ACCAAGTCCC TACTGCCATG TCGTCGTCCT CTACCCCTCA

GTCACAGGGA CCCCCTCCTA CTGTCAGTCA GATGCTCTCT GTGAAGAGGC

GTCACAGGGA CCCCCTCCTA CTGTCAGTCA GATGCTGTCT GTGAAGAGGC

ATCACAGGGA CCACCTCCTA CTGTCAGTCA AATGTTATCT GTGAAAAGGC

ACTCACCAGC AGCGCCAGCA CAGCAGGTCC AGGTCCAGGT TCAGCAGCCG

ATTCACCAGC GGCGCCAGCA CAGCAGGTCC AGGTCCAGGT G---CAGCCG

ATTCACCAGC ACCCCCACCA CAGCAGGTAC AAGTACAAGT TCAGCAGCCC

CAGCAGGTCC AGGTGCAAGT TCAGCCGCAG CAACCGAGTG CTGGGGTC

CCGCAAGTCC AGGTGCAAGT GCAGCCCCAG CAGGCGGGTG CAGGAGTT

CAACAAGTAC AGATGCAAGT TCAACCTCAA CAGTCGAATG CAGGAGTT

>Ortholog Group 361, Repeat 1

3 192

R00004519 AAAAGAAATG AAGCTGAGGC CCATGCGCCC CCGCCATTCA CACCCTATGT

H00355800 AAAGGAAGTG AGGCAGAGGC CCACATGCCC CCACCGTTCA CACCCTACGT

M00027800 AAAGGAAGCG AAGCCGAGGC CCACGTGCCC CCGCCATTCA CACCCTATGT

GCCTCGGATT CTGAACGGTC TGACCTCAGA GAGGACAGCA CTGTCCCCAA

GCCTCGGATT CTGAACGGCT TGGCCTCGGA GAGGACAGCA CTGTCTCCGA

GCCTCGGATT CTGAACGGTT TGGCCTCAGA GAGGACAGCG TTATCCCCAA

CCTACGGTGC CATCCGTAAC ATCAGTGGGA CTCTGCCTGG ACAGCTCGTG

CCTATGGTGC CATCCACAAC ATCAGCGGGA CTATCCCTGG ACAGTGCTTG

CCTACGGTGC CATCCGTAAC ATCAGTGGGA CTCTTCCTGG GCAGCCCGTG

GCTCAAGATC CTAGTGACAC TGTGGCTGGT GTCTACCAGG AA

GCGCAGAGCG CCACGGGCAG TGTGGCTGCT GCCCCCCAGG AG

GCTCAAGATC CTAGTGGCAC TGCGGCTTAT GCCTACCAGG AA

>Ortholog Group 362, Repeat 1

3 198

M00107033 AAGACGGTCG CTGAGTGTGT CCTCTATTAC TACCTGACCA AGAAGAATGA

H00348551 AAGACAGTGG CTGAGTGCGT CCTCTATTAC TACCTGACTA AGAAGAATGA

R00062850 AAGACAGTAG CCGAGTGTGT CCTCTATTAC TACCTGACCA AGAAGAATGA

AAATTACAAG AGCTTGGTGA GGCGGAGCTA TCGGCGCCGT GGCAAGAGC-

GAACTATAAG AGCCTGGTGA GACGGAGCTA TCGGCGCCGC GGCAAGAGCC

GAACTACAAG AGCTTGGTGA GGCGCAGCTA TCGGCGCCGT GGCAAGAGT-

--ATGGCACG GAGCAGCCAG GAGGAGAAGG AGGAGAAGGA GAAGGAGAAG

CCATGCCCCG CAGCAGCCAG GAGGAGAAAG ATGAGAAGGA GAAGGAAAAG

--ATGGCACG GAGCAGCCAG GAGGAGAAGG AGGAGAAGGA GAAGGAGAAG

GAGGCCGACA AGGAGGAAGA GAAGCAGGAT GCGGAGAACG AGAAGGAA

GAGGCGGAGA AGGAGGAGGA GAAGCCGGAG GTGGAGAACG ACAAGGAA

GAAGCCGACA AGGAGGAGGA GAAGCAGGAT GCGGAGAACG AGAAGGAA

>Ortholog Group 363, Repeat 1

3 111

H00379861 CCAAAGGTGA AGAAGCTCAA GTACCACCAG TACATCCCCC CGGACCAGAA

R00029616 CCAAAAGTGA AGAAGCTCAA GTACCACCAG TACATCCCCC CGGACCAAAA

M00105207 CCAAAGGTGA AGAAGCTCAA GTACCACCAG TACATCCCCC CGGACCAGAA

GCAGGACAGG GGGGCACCCC CCATGGACTC ATCCTACGCC AAGATCCTGC

GCAGGACAAG GGGGCGCCTG CCATGGACTC TTCCTACGCC AAGATCCTGC

GCAGGACAAG GGGGCGCCCG CCATGGACTC CTCCTATGCC AAGATCCTGC

TCTTCCTCCA G

TCTTCTTGCA G

TCTTCCTGCA G

>Ortholog Group 363, Repeat 2

3 123

H00379861 CAGATCCTCA AC-------- ----CACCAC AACTACCAGG CCATCCTGCC

R00029616 CAGATCCTCA ACCAGCAGCA GCAGCATTAC AACTACCAGG CCATCCTGCC

M00105207 CAGATCCTCA ACCAGCAGCA GCAGCACTAC AACTACCAGG CCATCCTGCC

TGCCCCGCCA AAGTCAGCAG GCGAGGCCCT GGGAAGCAGC GGGACCCCCC

CGCCCCTCCC AAGCCCTCGG GTGAGACTCC CGGAAGCAGT GCCCCCACCC

TGCCCCTCCC AAGCCCTCGG CTGAGACTCC TGGAAGCAGT GCCCCTACCC

CAGTACGCAG CCTCTCCACT ACC

CATCACGCAG TCTCTCCACC AGT

CATCACGCAG CCTCTCCACC AGT

>Ortholog Group 364, Repeat 1

3 111

R00041005 AATCCAGTCT CTGCGGCAGT GTCTGGTTCA GGGTCAGGCT CTGTGGCAGT

M00112637 AATCCAGTCT CTGTGGCAGT ATCTGGTTCA GGGTCAGGCT CTGTGGCAGG

H00421180 AATCCGGCAG CAGTGACAGT GGCCGGTTCA GCGTCAGGGC CTGTGGCTGT

GCCCAGCTCT GACATGTCCC CAGCAGAGCA GCTCAAACAG ATGGCTGCCA

GCCCAGCTCT GACATGTCCC CAGCAGAGCA GCTCAAACAG ATGGCTGCCA

GCCCAGCTCT GACATGTCTC CAGCAGAACA GCTCAAACAG ATGGCTGCAA

GGGCGAAGCT C

GGGCAAAACT C

GGGCCAAACT C

>Ortholog Group 364, Repeat 2

3 111

R00041005 ATGCAGCAGA AGCACTCAAA TCAGACTTCA AGTTGGTCTC CCTTAGGGCC

M00112637 ATGCAGCAGA AGCACTCAAA TCAGACTTCA AGTTGGTCTC CCTTAGGGCC

H00421180 ATGCAGCAGA AACACTCAAA TCAGACTTCA AATTGGTCTC CCTTAGGACC

TCCGTCAAGT CCGTATGGAA CAGCTTTTGC TTCAGAGAAA CCAAATAGCC

TCCGTCAAGT CCATATGGAA CAGCTTTTGC TTCAGAAAAA CCAAATAGCC

TCCCTCTAGT CCATATGGAG CAGCTTTTAC TGCAGAAAAA CCAAATAGCC

CAATGATGTA C

CAATGATGTA C

CAATGATGTA C

>Ortholog Group 364, Repeat 3

3 198

R00041005 GGCAACACTA AGCCTCTGAC CCATTTTAAC GCGGACTTGA GTCCGAGAAT

M00112637 GGCAACACTA AGCCTCTGAC CCATTTTAAT GCGGACTTGA GTCCGAGAAT

H00421180 GGCAACACTA AACCCCTGAC CCACTTCAAT GCAGACCTGA GTCAGAGGAT

GACGCCCCCT ATGGCCAACC CCAGTAAAAC CCCCTTGATG CCCTACATCC

GACGCCCCCT ATGGCCAACC CCAACAAAAC CCCCTTGATG CCCTACATCC

GACACCACCA GTGGCCAACC CCAACAAAAA CCCCTTGATG CCGTATATCC

CACCTCCACC GCAGCTGCAG GCTCCCAGGG CGCACCTGAG TGAGGACCAG

CGCCTCCCCC GCAGCTGCAG GCTCCCAGGG CGCACCTGAG CGAGGACCAG

CGCCACCTCC ACAGCTCCAG GCCCCCAGGG CACACCTGAG CGAAGACCAG

AAACGCATGC TTCTCATTAA GCAGAAAGGA GTGATGAACC CACCCATG

AAACGCATGC TTCTCATTAA GCAGAAAGGA GTGATGAACC CACCCATG

AAACGCCTGC TTCTCATGAA GCAGAAAGGA GTGATGAATC AGCCCATG

>Ortholog Group 364, Repeat 4

3 225

R00041005 GGCAGCCAGC CACAGGCAGC CATCATGAAG CAGATGCTAA TGGATCAACG

M00112637 GGCAGCCAGC CACAGGCAGC CATCATGAAG CAGATGCTAA TGGACCAACG

H00421180 GGCAGCCAGC CCCAAGCAGC CATCATGAAG CAGATGCTCA TTGATCAGCG

CGCCCAGCTG ATGGAACATC AGAAGCAACA GTTCCTTCGG GAGCAGAGGC

GGCCCAGCTG ATGGAGCACC AGAAGCAACA GTTCCTTCGG GAGCAGAGGC

GGCCCAGTTG ATAGAGCAGC AGAAGCAACA GTTCCTGCGG GAGCAAAGG-

AGCAGCAGCA GCAGCAACAG CAACAAATCC TGGCTGAGCA GCAATTACAC

AGCAGCAGCA ACAGCAGCAG CAGCAGATCC TGGCTGAGCA GCAGTTACAG

---------- ---------- ------ATTT TGGCGGAACA GCAGTTGCAG

CAGTCACACC TGCCCCGCCA GCACCTCCAG CAACAGCGGA CTCCATACCC

CAGCCGCACC TGCCCCGTCA GCACCTCCAG CAACAGCGGA ACCCATACCC

CAATCACATC TACCCCGGCA GCACCTCCAG CCACAGCGGA ATCCATACCC

AGTGCAGCAG GTCAATCAGT TCCAA

AGTGCAGCAG GTCAACCAGT TTCAA

AGTGCAGCAG GTCAATCAGT TTCAA

>Ortholog Group 365, Repeat 1

3 198

R00039322 TGCCAGTATG AGTCCATTGT GGAGACAAAC CGTAAAGACG TAGAAGAATG

M00103069 TGCCGGTACG AGTCCATCAT GGAGACAAAC CGTAAAGACG TAGAAGAATG

H00347823 TGTCAATATG AGCCCATCAT GGAGACAAAC CGCAAAGATG TGGAACAGTG

GTTCAATACA CAGATGGAGG AACTGAATCA ACAGGTAGTG AGCAGCTCTT

GTTCAATACA CAGATGGAGG AACTGAATCA ACAAGTAGTG AGCAGCTCTT

GTTCAACACG CAGATAGAGG AGCTGAATCA ACAAGTGGTG ACCAGCTCTT

GCTGCCAGAA GGACATCATA GAGCTGAGAC GGACCATAAG TGCCCTCGAG

GCTGCCAGAA GGACATCATA GAGCTGAGAC GGACCATAAG TGCCCTCGAG

GCTGCCAAAA GGAGATCATA GAACTGAGAC GCAGTGTGAA CACTCTGGAG

GTTGAGCTGC AGGCCCAGCA TCGAATGAGA GATTCCCAGG AATGCATC

ATTGAGCTGC AGGCCCAGCA CAGAATGAGA GAGTCCCAGG AATGCATC

GTTGAACTGC AGGCCCAGCA TCGAATGAGA GATTCCCAAG AGTGCATC

>Ortholog Group 366, Repeat 1

3 150

H00347184 ATGGCGACCC TGGAAAAGCT GATGAAGGCC TTCGAGTCCC TCAAGTCCTT

R00054971 ATGGCAACCC TGGAAAAACT GATGAAGGCT TTCGAGTCGC TCAAGTCGTT

M00078945 ATGGCAACCC TGGAAAAGCT GATGAAGGCT TTCGAGTCGC TCAAGTCGTT

CCCGCCACCG CCGCCGCCGC CGCCGCCGCC TCCTCAGCTT CCTCAGCCGC

C---GCGCCG CCACCACCGC CGCCGCCGCC GCCTCAACCC CCTCAGCCGC

T---CCGCCG CCACCGCCGC CGCCGCCTCC GCCTCAACCC CCTCAGCCGC

CGCCGCAGGC ACAGCCGCTG CTGCCTCAGC CGCAGCCGCC CCCGCCGCCG

CGCCTCAGGG GCAG------ ---------- ---------- -CCGCCGCCA

CGCCTCAGGG GCAG------ ---------- --------CC GCCGCCGCCA

>Ortholog Group 367, Repeat 1

3 198

M00104731 AAAGAGGAAG ATCCTGTGAA GAAAGTTCTG GAGAAGAAAT TTCATACTGT

H00351108 AAAGAAGAAG AACCCGTGAA GAAGGTTCTG GAGAAAAAGT TCCATACTGT

R00004885 AAAGAGGAAG ACCCTGTGAA GAAAGTTCTG GAGAAAAAAT TCCATACTGT

CAGTGGAAGC AAGTGTGAAA TCAAGGTTGC CCAGCCCAAA GAGGTGTATT

CAGTGGAAGC AAGTGTGAGA TCAAGGTGGC CCAGCCCAAA GAAGTCTATT

CAGTGGTAGC AAGTGTGAAA TCAAGGTTGC CCAGCCCAAA GAGGTGTACT

ATGGCTCTGG GGGCAGAGGA AATCGCAATC GAGGGAACCG AGGCAGT---

ATGGCTCTGG GGGCCGTGGA AACCGCAACC GAGGGAACCG AGGCAGCGGA

ATGGCTCTGG GGGCCGAGGA AATCGCAATC GAGGGAACCG AGGCAGT---

---------- --GGTGGAGG TCAGAGTCAG AGTTGGAATC AGGGCTAC

GGTGGTGGTG GAGGTGGAGG TCAGAGTCAG AGTTGGAATC AGGGCTAC

---------- --GGTGGAGG TCAGAGTCAG AGTTGGAATC AGGGCTAC

>Ortholog Group 368, Repeat 1

3 198

H00361824 AAGGATGAAG TGGCAGCTCG TATGAATGAG GTGATCAGTT TGTGGAAGAA

M00109348 AAGGAAGAAG TAGCAGCTCG TATGAATGAG GTCATCAGTT TGTGGAAGAA

R00019239 AAGGAAGAAG TAGCAGCTCG GATGAATGAG GTCATCAGTT TGTGGAAGAA

ACTGCTAGAG GCCACTGAAC TGAAAGGAAT AAAGCTTCGT GAAGCCAACT

ACTTCTAGAG GCCACAGAAC TGAAAGGAAT AAAGCTCCGA GAAGCCAACT

ACTTCTAGAG GCCACAGAAC TGAAAGGAGT CAAGCTCCGA GAAGCCAACT

TTAATCGCAA TGTTGAGGAT ATTGAATTGT GGCTATATGA AGTAGAAGGT

TTAATCGAAA TGTTGAAGAC ATTGAATTGT GGCTGTATGA AGTAGAAGGT

TTAATCGAAA TGTTGAGGAC ATTGAGTTGT GGCTGTATGA AGTTGAAGGT

CACTTGGCTT CGGATGATTA CGGCAAAGAT CTTACCAATG TGCAGAAC

CATTTGGCTT CAGATGATTA TGGTAAAGAC CTCACTAATG TCCAGAAC

CACTTGGCTT CAGATGATTA TGGTAAAGAC CTCACTAATG TCCAGAAC

>Ortholog Group 369, Repeat 1

3 204

H00352314 CTCTTCTCCA GCCACCCCCC TCTCGGGGGA GGGGTCGGCG GCCAGGACGG

R00020697 CTCTTCTCCA GCCACCCCCC TCTCGGGGGA GGGGTCGGCG GCCAGGACGG

M00113755 CTCTTCTCCA GCCACCCCCC TCTCGGGGGA GGGGTCGGCG GCCAGGACGG

CCTGGGGGCC CCCAAGGAC- -----CAGCA GCAGCCGCCG CAGCAGCAGC

CCTGGGGGCC CCCAAGGACC AGCAGCAGCA GCAGCAGCCG CAGCAGCAGC

CCTGGGGGCC CCCAAGGACC AGCAGCAGCA GCAGCAGCCT CAGCAGCAGC

CGTCACCGCC GCCGCAGGAG GAGCCCGGGA CTCCTTCTTC CTCCCCCGAC

CTTCACAGCC ACCACAGGAG GAGCCCGGGA CTCCTTCCTC CTCCCCCGAC

CTCCACCGCC GCCACAGGAG GAGCCCGGGA CTCCTTCCTC CTCCCCCGAC

GACAAGCTGC TGACCAGCCC CCGGGCCATC AACAACCTGG TGCTGCAGGG

GACAAGCTGC TGACCAGTCC CCGAGCCATC AACAACCTAG TGCTGCAGGG

GACAAGCTGC TGACCAGCCC CAGAGCCATC AACAACCTAG TGCTGCAGGG

CTGC

CTGC

CTGC

>Ortholog Group 36, Repeat 1

3 198

R00039753 AAACAAAATT CCAATAATGC AAGAGAAAAT TCTAATAAAG TA------GA

H00327077 AAGCAAAATT CAAATAACAC TAGAGGAAAT GCCAATAAAA CACAGAAAGA

M00039056 AAACAAAATT CCAATAATGC AAGAGAAAAT TCTAATAAAA TA------GA

TACTGGGGTA AATGAAAAAA CAAGAGAGAA ATTTTATGAG GCCAAACTAA

TACTGGAGTA AATGAAAAGG CAAGAGAGAA ATTTTATGAG GCTAAACTAA

TACTGGAGTA AATGAAAAAA CAAGAGAGAA ATTTTATGAG GCTAAACTAA

GAGAGCTTAA ACAGTTGCAG GAGGAAAGAA AGAAATTGAT TGAAATTCAA

GAGAGCTAAA ACAATTGCAG GAAGAAAGAA AGAAACTGAT TGACATTCAG

GAGAGCTTAA ACAATTGCAG GAGGAAAGAA AGAAATTGAT TGAAATTCAA

GAGAAAATTC AAGCAGTTCA AAAGGCATGC CCTGACCTTC AGCTATCA

GAGAAAATTC AAGCATTGCA AACGGCATGC CCTGACTTAC AGCTGTCA

GAGAAAATTC AAGCAGTGCA AAAGGCATGC CCTGACCTTC AGCTCTCA

>Ortholog Group 370, Repeat 1

3 198

R00027056 GTAGCAGGCA CGTACCAGAA TACGGCTCAG ACTGTACATA TATGGGACCC

H00350094 GTGGCAGGCA CGTACCAGAA TACGGCTCAA ACTGTCCATA TATGGGACCC

M00006851 GTAGCAGGCA CATACCAGAA TACGGCTCAG ACTGTACATA TATGGGACCC

ACAGCAGCAG CCGCAACAGC AAACTGCACA AGAACAGACA CCACCACAGC

CCAACAGCAG CCGCAGCAGC AAACTCCCCA GGAACAGACA CCACCACCAC

ACAGCAGCAG CCACAACAGC AAACTGCACA AGAGCAGACA CCACCACCAC

TGCAGGTCAC TTGTTCAGCA CAAACTGTTC AGGTCGCTGA AGTTGAGCCA

TCCAAGTTAC TTGTTCAGCT CAAACTGTCC AGGTTGCTGA AGTTGAACCA

TGCAGGTCAC TTGTTCAGCA CAGACTGTTC AGGTTGCTGA AGTTGAGCCA

CAGTCACAAC CGCAGCCTTC ACCAGAGCTT TTGCTTCCAA ATTCACTG

CAGTCACAGC CACAGCCTTC CCCAGAACTT CTGCTTCCAA ATTCTTTG

CAGTCACAAC CACAGCCTTC ACCAGAGCTT TTGCTTCCAA ATTCTTTG

>Ortholog Group 371, Repeat 1

3 132

R00024714 CAACAGAGTC ATCCCTCAGG ATCTTTACCT CCAGCGCATC ACCCAATGCA

H00363929 CAGCAAAGTC ATCCCTCAGG ATCTTTAGCT CCCCCACATC ACCCAATGCA

M00105295 CAACAGAGTC ATCCCTCAGG ATCTTTACCT CCAGCGCATC ACTCAATGCA

ACCTGTTCCT GTGAACAGAC AAATGAACCC AGCTAATTTT CCCCAGCTG-

GCCTGTCTCT GTGAACAGAC AAATGAACCC AGCTAATTTT CCCCAGCTG-

GCCTGTTCCT GTGAACAGAC AAATGAACCC AGCTAATTTT CCCCAGCTGC

---------- ---------- TTGCAGACTA GA

---------- ---------- TTGCAGGCAA GA

AGCAGCAGCA GCAGCAACAG TTGCAGACAA GA

>Ortholog Group 371, Repeat 2

3 111

R00024714 TTACAACAAC ATCCACAGGG GATTCGACCA CAGTTTACTG CTCCAACTCA

H00363929 CCACAGCAAC ATCCACAGGG AATTCGACCC CAGTTTACTG CCCCAACTCA

M00105295 CTACAGCAAC ATCCACAGGG GATTCGACCA CAGTTTACTG CTCCAACTCA

GGTGCCTGTT CCTCCAGGCT GGAACCAGCT GCCTTCTGGA GCCTTACAGC

GGTGCCTGTT CCTCCAGGCT GGAACCAGCT GCCTTCTGGA GCCCTTCAAC

GGTGCCTGTT CCTCCAGGCT GGAACCAGCT GCCTTCTGGA GCCTTACAGC

CTCCACCAGC C

CTCCTCCAGC C

CTCCACCAGC C

>Ortholog Group 371, Repeat 3

3 111

R00024714 GTTGAACAGA GGCCACTGCA GCAGATGCCT CCTCAACTCA TGCAGCATGT

H00363929 GTTGAACAGA GGCCACTTCA GCAGATGCCT CCTCAACTCA TGCAGCATGT

M00105295 GTTGAACAGA GGCCACTACC GCAGATGCCT CCTCAGCTCA TGCAGCATGT

GGCACCCCCA CCACAGCCAC CACAGCAGCA GCCACAACCA CAACTGCCTC

GGCACCCCCA CCACAGCCAC CACAGCAGCA GCCACAGCCA CAACTGCCTC

CGCACCCCCA CCACAGCCAC CACAGCAGCA GCCACAACCA CAACTGCCTC

CACCACCACC T

CACCACCTCC C

CACCACCACC T

>Ortholog Group 371, Repeat 4

3 111

R00024714 CAGCCACAGT CGATGATGAT GATGCTCATG ATGCAGCAAG ATCCCAAATC

H00363929 CAGCCACAGT CTATGATGAT GATGCTCATG ATGCAGCAGG ATCCCAAATC

M00105295 CAGCCACAGT CCATGATGAT GATGCTCATG ATGCAGCAAG ATCCTAAATC

CATTAGGCTT CCGGTCTCCC AAAATGTCCA TCCTCCACGG GGTCCTCTGA

AGTTAGGCTT CCAGTCTCTC AAAATGTCCA TCCTCCAAGG GGCCCCCTGA

CATTAGGCTT CCAGTCTCCC AAAATGTCCA TCCTCCACGG GGTCCTCTGA

ACCCAGACTC C

ACCCCGACTC C

ACCCAGACTC C

>Ortholog Group 372, Repeat 1

3 198

H00355260 CGGGGTGGCG CCGGTGGCAG CCCCGGCTCC AGCAGCGGCT CAGGCTCCTC

R00022046 CGCGGGGGCA CCGGCGGCAG CCGGGAATCC AGCAGCCACT CCGGCTCCTC

M00045110 CGCGGTGGAA CCGGCGGCAG CCCAGGATCC AGCAGCAGCT CCGGCTCCTC

CCGGGAGGAC TCGGCGCCCG TGGCCACGGC GGCCGCTGCA GGGCAGGTTC

AAGGGAGGAT TCGGCGCCGG TGGCCACAGT GGCAGCCGCT GGGCAAGTGC

AAGGGAGGAC TCGGCGCCGG TGACTACGGT GGCCGCCGCC GGGCAAGTGC

GGCGACACCA GCAGGGAAAA GTGACAGTGA AATACGATCG TAAGGAGCTT

GCCGCCACCA GCAGGGAAAA GTGACAGTGA AATATGACCG TAAGGAGCTT

GACGCCACCA GCAGGGAAAA GTGACAGTGA AATACGACCG TAAGGAGCTT

CGGAAGCGGC TGGTGCTGGA GGAATGGATC GTGGAGCAGC TGGGTCAG

CGGAAGCGGT TGGTGCTGGA AGAATGGATC GTGGAGCAAC TGGGTCAG

CGTAAGCGGT TGGTGCTGGA AGAATGGATC GTGGAGCAGC TGGGTCAG

>Ortholog Group 373, Repeat 1

3 198

M00096514 AAATGGCCAT CTGGTACTCC GGGTGGACGC CTACGCTGGG CCAAGCTCCG

R00048545 AAATGGCCAT CTGGTACTCC GGGTGGACGC CTACGCTGGG CCAAGCTCCG

H00354597 AAGTGGCCGT CTGGCACACC AGGTGGGCGT CTGCGCTGGG CCAAGCTCCG

GAATGTTGTC CTGGGGGCTG CTCAGTTCCG CCAGCCCTTA AAGGACCGAC

GAATGTTGTC TGGGGGGCGG CTCAGTTCCG CCAGCCCTTA AAGGACCGAC

GAATGTTGTC CTGGGGGCTG CTCAGTTCCG CCAGCCCTTG AGAGAACGGG

AACAGGTGGC CGAGCAGCAA AAGTCGGGCA GCTCTCAGAC AGAGCCCTAT

AACAGGTGGC CGAGCAGCAA AAGTCGGGCA GCTCTCAGAC AGAGCCCTGT

AGCAGGTGTC AGCACATCAA GAGGCAGGCA GCTCCCAGGC AGAGCCCTAT

CTGGAGCGTC CCTCCCCAAC GCGCCCTCTT CAGCGCCAGA CTACTTGG

CTGGAGCGTC CTTCCCCAAC CCGCCCTCTT CAGCGCCAGA CTACTTGG

TTGGAGCGCC CTTCCCCTAC TCGCCCTCTT CAGCGCCAGA CTACTTGG

>Ortholog Group 374, Repeat 1

3 201

R00038197 CGCATGCTGC TCCCTTCTTC TTCTTCCTCG AAGCCACCGG GCTTGGGCAC

M00125251 CGAATGCTGC TCCCT---TC CTCTTCCTCG AAGCCGCCAG GCTTGGGCAC

H00355133 AGGATGCTGC TCCCT---TC TTCTTCCTCG AAGCCTCCAG GCCTGGGCAC

TGGGACGCCC CTGTCCACTC ACCACCAGAT GCAGCTCCTC CAGCAGCTCC

GGGGACGCCC CTGTCCACTC ACCACCAGAT GCAGCTCCTC CAGCAGCTCC

AGAGACACCG CTGTCCACTC ACCACCAGAT GCAGCTCCTC CAGCAGCTCC

TCACACAAGT GGCTGTGGCT CAGGTTCACT TGCTGAAGGA TCAGTTGGCT

TCACACAAGT GGCTGTGGCC CAGGTACACT TACTGAAGGA TCAGTTGGCT

TCACACAAGT GGCTGTGGCC CAGGTACACT TGCTGAAGGA CCAGTTGGCT

GCTGAGGCTG CGGCACGGCT GGAGGCCCAG GCACGAGTGC ACCAGCTCCT

GCTGAGGCTG CGGCGCGGCT GGAGGCCCAG GCACGAGTGC ACCAGCTCCT

GCTGAGGCTG CGGCGCGGCT GGAGGCCCAG GCTCGCGTGC ATCAGCTTTT

G

G

G

>Ortholog Group 375, Repeat 1

3 198

R00032208 GCCTTATACC AGATGCAACA CCTCCAGTAC CAGCAGTTCT TAATACAACA

H00386765 GCCTTATACC AGATGCAGCA CCTGCAGTAC CAGCAGTTTT TAATACAACA

M00027475 GCCTTATACC AGATGCAGCA CCTCCAGTAC CAGCAGTTCT TAATCCAACA

ACAGTATGCG CAGGTCTTGG CTCAACAGCA GAAAGCAGCT TTGTCATCTC

ACAATATGCA CAGGTTTTGG CCCAACAGCA GAAAGCAGCA CTGTCTTCCT

ACAGTATGCA CAGGTTTTGG CTCAACAGCA GAAAGCAGCT TTGTCATCTT

TGGCTCTTCT TCTGCAACAG TTCCAGGCTC TGAAGATGAG AATGTCTGAT

TGGCACTTCT TCTTCAACAG TTTCAGACCT TGAAGATGAG AATATCTGAT

TGGCTCTTCT TCTGCAGCAG TTCCAGGCTC TGAAGATGAG AATGTCTGAT

CAGAACATCA TTCCCTCAGT AACTAGG--- GTGTCAGTGC CAGATACT

CAGAACATCA TTCCCTCAGT AACTAGGTCT GTGTCCGTGC CAGATACT

CAGAACATCA TTCCCTCAGT AACTAGGTCT GTGTCAGTGC CAGATACT

>Ortholog Group 375, Repeat 2

3 108

R00032208 CGCATGGAGG AGGAGGCAGC CAGACTTAGG CATGAAGAAG AGGAACGAAA

H00386765 CGGATGGAAG AGGAGGCAGC CAGACTCCGG CATGAGGAAG AAGAACGGAA

M00027475 CGCATGGAGG AGGAGGCTGC CAGACTTCGG CATGAAGAAG AAGAAAGAAA

ACGGAAGGAA CTGGAGCTGC AGAGACAGAA GGATCTGATG CGCCAGAGGG

GAGAAAGGAG CTGGAGGTCC AGCGGCAGAA GGAGTTAATG CGCCAGAGGG

ACGGAAGGAA TTGGAGCTGC AGAGGCAGAA GGATCTGATG CGTCAGAGAG

AAGCCCTC

AGGCTCTC

AGGCTCTC

>Ortholog Group 375, Repeat 3

3 96

R00032208 CGAAGACTGC TTGCACAAAT GAAGCTTCCC TCTTCGTCAA CGTGGGGACA

H00386765 CGGAGGTTGC TGGCGCAGAT GAAGCTTCCT TCTTCTTCAA CGTGGGGCCA

M00027475 CGAAGGCTGC TTGCACAAAT GAAGCTTCCC TCTTCGTCAA CGTGGGGTCA

GCAGTCTAAC ACCACAACAT GTCAGTCCCA GGCCACACTG TCATTG

GCAGTCCAAT ACAACAGCAT GTCAGTCCCA GGCCACGCTG TCGTTG

GCAGTCTAAC ACAGCAACAT GTCAGTCCCA GGCCACGCTG TCATTG

>Ortholog Group 375, Repeat 4

3 135

R00032208 GCTGAGATCC AGAAGCTGGA AGAAGAACGA GAACGCCAGC TTCGAGAAGA

H00386765 GCTGAAATCC AAAAACTAGA GGAAGAACGA GAACGGCAGC TTCGAGAAGA

M00027475 GCTGAGATCC AGAAGCTGGA AGAAGAACGA GAACGCCAGC TTCGTGAAGA

GCAAAGGCGT CAGCAGAGGG AATTGATGAA AGCTCTCCAG AAACTCTCAG

GCAAAGGCGC CAGCAGAGGG AGTTGATGAA AGCTCTT--- AAACTCTCAG

GCAAAGACGT CAGCAGAGGG AATTGATGAA AGCTCTCCAG AAACTCTCAG

GTTGGGGGAA TGTCAGCAAA CCTGCAGGTA CCACC

GTTGGGGGAA TGTCAGCAAA CCTTCAGGTA CCACG

GTTGGGGGAA TGTCAGCAAA CCTGCAGGTA CCACC

>Ortholog Group 375, Repeat 5

3 156

R00032208 TCTCTACTAG AGATCCAGCA GGAAGAGGCC AGGCAGATGC AGAAG---CA

H00386765 TCTCTTCTGG AGATCCAGCA GGAAGAGGCC AGGCAAATGC AAAAG-----

M00027475 TCTCTACTAG AGATCCAGCA GGAAGAGGCC AGGCAGATGC AGAAGCAGCA

GCAGCAGCAC CAGCAATCAA ACAGAGCCCG GAACAGCACA CATTCCAACC

-------CAC CAGCAACCAA ACAGAGCTCG TAACAATACG CATTCCAACC

GCAGCAGCAC CAGCAATCAA ACAGAGCCCG GAACAGCACA CATTCCAACC

TGCACACCAG CATTGGGAAT TCTGTATGGG GCTCTATAAA CACAGGTCCT

TGCACACCAG CATTGGGAAT TCTGTTTGGG GCTCTATAAA TACTGGTCCT

TGCATACCAG CCTTGGGAAT TCTGTATGGG GCTCTATAAA CACAGGTCCT

CCTAAC

CCTAAC

TCTAAC

>Ortholog Group 375, Repeat 6

3 102

R00032208 GACTATACCA GGGCCTATTT AGGAGATACT TCTGAGGCTA AGGAGTTTGC

H00386765 GATTATATCA GGGCCTATTT AGGAGATACT TCTGAGGCCA AGGAGTTTGC

M00027475 GACTATACCA GGGCCTATTT AGGAGATACT TCCGAGGCCA AGGAGTTTGC

TAAGCAGTTC CTTGAGCGCC GTGCCAAACA GAAAGCCAAC CAGCAGCGT-

CAAGCAGTTC CTTGAGCGCC GTGCCAAACA GAAAGCCAAC CAGCAGCGTC

CAAGCAGTTC CTTGAACGCC GTGCCAAACA GAAAGTCAAC CAGCAGCGT-

--

TG

--

>Ortholog Group 375, Repeat 7

3 102

R00032208 ---------- ---------- -CAGCAGCAG CAACAGCAGG ACTCTGTCTG

H00386765 CCACCGCCAC AGCAGCCGCC ACAGCAGCCA CAACAGCAGG ACTCTGTGTG

M00027475 ---------- ---------- -CAGCAGCAG CAGCAGCAGG ACTCTGTCTG

GGGGATGAAC CATAGTACAC TCCATTCGGT ATTTCAGACC AATCAAAGCA

GGGGATGAAC CACAGTACAC TCCATTCAGT ATTTCAGACC AATCAAAGCA

GGGGATGAAC CATAGTACAC TCCATTCGGT ATTTCAGACC AATCAAAGCA

AC

AC

AC

>Ortholog Group 376, Repeat 1

3 198

R00001160 GGTTCCGAGA TCACTGCACC CCAAAAGACA GCTGCTCAGC TCAAGAAAGA

H00364815 GGCCCCGAGG CTCCTGCCCT CCCAAAGACA GCTGCTCAGC TCAAGAAAGA

M00084572 GGCTCTGAGG TCATCGCACC CCAAAAAACA CCCGCGCAGC TCAAAAAAGA

AGCAAAGAAA CGGGAGAAAC TAGAGAAATT CCAACAGAAG CAGAAGACGC

GGCAAAGAAA CGGGAGAAGC TAGAGAAATT CCAACAGAAG CAGAAGATCC

GGCAAAGAAA CGGGAGAAAC TAGAGAAATT CCAGCAGAAG CAGAAGACCC

CGGCACACGG GGAGAAGAAA CCAAAACCAG AGAAGAAGGA GAAGCGAGAC

CACCTCCAGG GGAGAAGAAA CCAAAACCAG AGAAGAGGGA GAAACGGGAT

CCCCGCACGG AGAGAAGAAA CCAAAACCAG AGAAGAAGGA GAAACGGGAC

CCCGGGGTCA TTACCTATGA CCTCCCTACC CCACCTGGGG AGAAGAAA

CCTGGGGTCA TTACCTATGA CCTCCCAACC CCACCCGGGG AAAAGAAA

CCTGGGGTCA TTACCTATGA CCTCCCTACC CCACCGGGGG AGAAGAAA

>Ortholog Group 377, Repeat 1

3 198

H00384672 CTACTTGGTG ATATGTCATC AATAAACAAT ACTTTGAGTA ACCATCAACT

M00036847 CTGCTTGGTG ATATATCATC CATAAACAAT TCATTGAATA ACCATCAACT

R00031544 CTGCTTGGCG ATATGTCATC CATAAACAGT TCTCTGAATA ACCATCAACT

GACTCATCTA CAGTCGCTGT TAAACAACAA TCAGATGTTT CCTCCAAATC

GACTCATCTA CAGTCGCTAC TAAACAGCAA TCAGATGTTT CCTCCAAATC

GACTCACCTA CAGTCACTGC TAAACAGCAG TCAGATGTTT CCTCCGAGTC

TTCTCCAGGG GTACCAGAAT CTCCAGGCGT TCCAAGGACA GTCCACAATT

TTCTCCAGGG ACACCAGAAT CTCCAGGCCT TCCAAGGACA ACCCACAGTC

TTCTCCAGGG ACAGCAGAAT CTCCAGGCCT TCCAAGGACA ACCCACAGTC

CCTTGCCCAG CTAACAATAA CCCCATGGCT TGTCTGTTTC AGAACTTT

CCTTGCCCAG CTAACAATAA CCCCATGGCT TGTCTGTTTC AGAACTTC

CTTTGCCCAG CTAACAATAA CCCCATGGCA TGTCTGTTTC AGAACTTC

>Ortholog Group 378, Repeat 1

3 99

H00365080 CTGTCTCTGG CATCTCTCTT GTTTTTCACA GTCCTGCTCT CTGATCACTT

M00106596 CTGTCTCTGG CTTCTCTCTT GTTTTTCACA GTCCTGCTCT CTGATCACTT

R00054167 CTGTCTCTGG CTTCTCTCTT GTTTTTCACA GTCCTGCTCT CTGATCACTT

GTGGTTCTGC GCCGAGGCCA AGCTGACCCG GGCCCGGGAC AAGGAGCAC

GTGGTTCTGC GCCGAGGCCA AGCTGACCCG GACCCGGGAC AAAGAGCAT

GTGGTTCTGC GCCGAGGCCA AGCTGACCCG GACCCGGGAC AAAGAGCAT

>Ortholog Group 378, Repeat 2

3 3

H00365080 AGG

M00106596 CAG

R00054167 CAG

>Ortholog Group 378, Repeat 3

3 135

H00365080 CGGCGG---- ---------- ---------- ---------C GGCAGCAGGA

M00106596 CAGCGACAGC AGCAGCGGCA GCGACAGCAG CAGAGGCAGC GACAGCAGGA

R00054167 CAGCGACAGC AGCAGCGGCA GCGACAGCAG CAGAGGCAGC GACAGCAGGA

GCCCTCCTGG CCCGCGCTCC TGGCGAGCAT GGGGGAGTCC TCGCCCGCCG

GCCCTCCTGG CCCGCGCTCC TGGCCAGCAT GGGGGAGTCC TCGCCCGCCG

GCCCTCCTGG CCCGCGCTCC TGGCCAGCAT GGGGGAGTCC TCGCCCGCCG

CCCAGGCACA CAGACTCCTC TCCGCCTCCT CGTCC

CCCAGGCACA CAGACTCCTC TCCGCCTCCT CGTCC

CCCAGGCACA CAGACTCCTC TCCGCCTCCT CGTCC

>Ortholog Group 379, Repeat 1

3 204

M00025253 TCTACTCGCC AGCCCCCCAG TCAGGGCTTG GGCTACCCCA AATACCAGAA

H00365175 CCCACCCGCC AGCCCCCTAG TCAGGGCTTG GGCTACCCCA AATATCAGAA

R00001133 CCCACTCGCC AGCCCCCCAG TCAGGGCTTG GGCTATCCCA AATACCAGAA

GTCATTGCCT CCTCGCTTCC AGCGGCAGCA ACAGGAGCAG CTCCTGAAGC

GTCGTTGCCT CCTCGTTTCC AGCGGCAGCA GCAGGAGCAG CTCCTGAAGC

GTCATTGCCA CCTCGCTTCC AGCGGCAGCA ACAGGAGCAG CTCCTGAAGC

AACAG---CA GTGGCAGCAG CAGCAACAAG GCACTGCCCC TCCTGCCCCA

AC------CA GTGGCAGCAG CATCAACAGG GCTCTGCCCC TCCTACCCCA

AGCAGTGGCA GCAGCAGCAG CAACAACAAG GCACTGCCCC TCCTGCCCCA

GTGCCCCCAT CACCACCACA ACCTGTGACC CTGGGAGCTG TACCTGCCCC

GTGCCCCCAT CACCACCACA GCCTGTGACC CTGGGGGCTG TGCCAGCTCC

GTGCCCCCAT CACCACCACA GCCTGTGACC CTGGGAGCTG TACCTGCCCC

ACAA

ACAG

ACAG

>Ortholog Group 37, Repeat 1

3 204

M00101269 CACGTGCGAG GCCGGGAGAA CTTTGAGATC TTGATGAAAG TCAAGGAGAG

R00052265 CATGTTCGAG GCCGGGAGAA CTTTGAGATC TTGATGAAAG TCAAGGAGAG

H00367545 CAGGTGCGAG GCCGGGAGAA CTTTGAGATC CTGATGAAGC TGAAAGAGAG

CCTAGAACTG ATGGAGCTTG TGCCCCAGCC TTTGGTTGAC TCCTATCGAC

CCTAGAGCTG ATGGAGCTTG TGCCGCAGCC TTTGGTTGAC TCCTACCGAC

CCTGGAGCTG ATGGAGTTGG TGCCGCAGCC ACTGGTGGAC TCCTATCGG-

AGCAGCTCCT ACAGAGGCCG AGTCACCTGC AGCCTCCATC CTATGGGCCC

AGCAACTCCT ACAGAGGCCG AGTCACCTGC AGCCTCCCTC CTATGGGCCC

-----CTCCT ACAGAGGCCG AGTCACCTAC AGCCCCCGTC CTACGGGCCG

GTGCTCTCCC CAATGAACAA GGTACACGGT GGTGTCAACA AACTGCCCTC

GTGCTGTCCC CAATGAACAA GGTACACGGT GGTGTCAACA AACTGCCCTC

GTCCTCTCGC CCATGAACAA GGTGCACGGG GGCATGAACA AGCTGCCCTC

CGTC

TGTC

CGTC

>Ortholog Group 380, Repeat 1

3 198

M00035452 ATCGGGAGGT TTGAAGATGA TGCCTCCCGG GACACGCAGG AGCCCGAGTC

R00001113 ATCGGAAGGT TTGAAGATGA TGCCTCCCGG GAGACGCAGG AGCCCGAGTC

H00365318 ATGGGGAGGT TTGAAGGTGA TGCCTCCCAT GAAACCCAGG AACCTGAGTC

CTTCTCAGCC TGGTCAGAGC GCCTGGCTCG GGAGCATGCC CAGAAGCAGC

CTTCTCAGCC TGGTCAGAGC GCCTGGCCCG AGAGCATGCC CAGAAGCAGC

CTTCTCAGCC TGGTCAGATC GCCTGGCCCG GGAACATGCC CAGAAGTGCC

TGGAGGCAGA GGGATCCTGC CGACCTCCAA GGGCTGAGGG CTCCAGTCAC

TGGAGGCAGA GGGATCCCGC CGACCCCCAA GGGCTGAGGG CTCCAGTCAC

GAGAAGCAGA GGGATCCCGT CGACCCCCAC GTGCTGAGGG CTCCAGCCAG

AGCTGGCGAC AGCACGAGGA GGAACAGCGG CTTTTCCGAG AGCGAGCC

AGCTGGCGAC AGCAAGAGGA GGAGCAGCGG CTTTTCCGGG AGCGAGCC

AGCTGGCGAC AGCAGGAGGA GGAGCAGCGG CTCTTCAGGG AGCGAGCC

>Ortholog Group 381, Repeat 1

3 204

M00036881 GGAAAAAAGG ACAAGAAGAC CAAAAAGACG TTCTTTGAAG AGCTGGCAGT

H00313603 GGGAAGAAGG ACAAGAAGAT CAAAAAAACG TTCTTTGAAG AGCTGGCAGT

R00001049 GGCAAAAAAG ACAAGAAGAC CAAAAAGACG TTCTTTGAGG AACTGGCAGT

AGAGGACAAA CAAGCTGGGG AAGAGGAAAA ATTGCAGAAG GAGAAGGAGA

AGAAGATAAA CAGGCTGGGG AAGAAGAGAA AGTGCTCAAG GAGAAGGAGA

GGAAGACAAG CAAGCCGGGG AAGAGGAGAA ACTGCAGAAG GAGAAGGAGA

AGAAAAAGCG AGACACCAGG AAAGGCCGTC GGAAAAAGGA TGTG------

AAAAAAAGCG AGATACCCGA AAAGGCAGGC GGAAGAAGGA TGTG------

AGAAAAAGCG AGACACCAGG AAAGGTCGTC GGAAGAAGGA TGTGGATGAT

GACGATGATA GTGATGAGAG AGTGCTCATG GAGCGACTTA AGCAACTGTC

GATGATGATG GAGAAGAGAA AGAGCTCATG GAGCGTCTTA AGAAGCTCTC

GATGATGATG GTGATGAGAG AGTGCTCATG GAGCGCCTTA AGCAGCTGTC

TGTG

AGTG

TGTG

>Ortholog Group 382, Repeat 1

3 198

R00057317 ---------- ---------- ---------- ---------- ----------

H00417711 TTTCCCTGCA TTCCAGAAAG CCAGTTCCTG GAACTGCAGA CAGCAAGTGA

M00103625 ---------- ---------- ---------- ---------- ----------

---------- ---------- ---------- ---------- ----------

CCCGGACCTG CATCACAAAG TTATGCCCTG GATGCCAACG GCCCTTGATG

---------- ---------- ---------- ---------- ----------

---------- ---------- ---------- ---------- ----------

CTTTACCAGG TACTGTCACT GAACACCCTA AGCTGCTCCG AGTCTTGTCT

---------- ---------- ---------- ---------- ----------

---------- ---------- ---------- ---------- --------

GTGGAAGCCA TTGAGAAACT GGAGACAACT TTACGGCACA AGCATCTG

---------- ---------- ---------- ---------- --------

>Ortholog Group 383, Repeat 1

3 201

R00011300 GTGGGGATCC GAGTGCAGAT TCACAGCCAG GATGAGCCCC CTGCCATTGA

H00297512 GTGGGGATCC GAGTGCAGAT CCACAGCCAG GAGGAGCCGC CCATCATCGA

M00039914 GTGGGGATCC GAGTGCAGAT CCACGGCCAG GAGGAACCCC CTGCCATTGA

CCAGCTGGGC TTCGGGGCAG CCCCAGGCCA TCAGACTTTT GTGTCCTGTC

TCAGCTGGGC TTGGGGGTGT CCCCGGGCTA CCAGACCTTT GTTTCTTGCC

CCAGCTGGGC TTCGGTGCTG CCCCAGGCCA CCAGACTTTT GTGTCCTGCC

TGAGTTTCCT GCCACCACCC TGGGGTGACT GCAATACCGC ATCTTTGGAT

TGAGCTTCCT GCCACCGCCC TGGGGCGATT GCAGTTCAGC ATCTCTGAAC

TGAGTTTCCT GCCACCACCC TGGGGTGACT GCAATACCGC ATCTGTGGAT

CCCGACGACT TTGATCCAGA GCCCTCTGAT CCCTTGGGTT CCCCCAGACC

CCC---AACT ATGAGCCAGA GCCCTCTGAT CCCCTAGGCT CCCCCAGCCC

CCC---GACT TTGATCCAGA GCCCTCTGAT CCCCTGGGTT CC------CC

C

C

T

>Ortholog Group 384, Repeat 1

3 162

H00384250 ACTACAGAAC AGCACGTACC AGAGGTGGAA GTCCAAGTCA AACGCAGAAG

R00030887 ACTACTGAAC AGCACGTCCC AGAGATTGAG GTCCAGGTCA AACGTAGAAG

M00096003 ACTACTGAAC AGCACGTGCC CGAGGTCGAG GTCCAGGTCA AACGTCGAAG

GACTGCCTCA CTGAGCAACC AAGAGTGTCA GTTGTACCCG AGGCGTTCTG

GACAGCCTCA CTGAGCAACC AAGAGTGTCA CTTGTACCCA CGGCATTCTA

GACAGCCTCA CTGAGCAACC AAGAGTGTCA CTTGTACCCA CGACGTTCTG

TACCTGTGGT GGATTTCCAG GCTGAACTGA GGCAGGCATT CTTAGCTGAG

TTCCTGTGGT GGATTTCCAG GCAGAACTGA GACAGGCGTT CTTAGCTGAG

TTCCTGTGGT GGATTTCCAG GCAGAACTAA GACAGGCATT CTTAGCTGAG

ACACCAAGAG GT

ACACCAAGAG GT

ACACCAAGAG GT

>Ortholog Group 385, Repeat 1

3 198

M00063284 ---------- ---------- ---------- ---------- ----------

H00387020 GACACTGGGA TCCACTCAGA AGAGTTGCTG CAACCCTACC CTTCTGCTCC

R00062181 GACAGTGGGA TCCACTCAGA AGAAATGCTC CAACCCTACC CTTCCACTCC

---------- ---------- ---------- ---------- ----------

CAGTTCTGGC CCTGCCATCA CACATCTGAT GCCCCCAGTG AAGAAAAGCA

CAGTTCTGCC CCTGCTGTCA CACATCTGAT GGCCCCAGCG AAGAAAGAGA

---------- ---------- ---------- ---------- ----------

GCCTGATGGA GAGCATGCAG CCCGGGAAGC CCAGTGACTG GGAGCTGGAG

GCCTCATGGA GAGCATGCAG CCCGGGAAGC CAGGAGACTG GGAGATGGAG

---------- ---------- ---------- ---------- --------

GGCAGGAAGC ACGAGCGGCC CGAGAGCCTT CTGGCACCGA CGCAGTTC

GGCAGAAAGC ACGAGCGGCC CGAGAGCCTG CTGGCGCCAG CGCAGTTC

>Ortholog Group 386, Repeat 1

3 243

M00028665 CCTTCACCAG ATCCTTTTCT GTTCTACAGC CCACTGACCC CGTGGCCCCC

R00022964 CCCTCACCAG ATCCTTTTCT GTTCTGCAGC CCTCTGACCC CGTGGCCCCC

H00416673 CCCTCACCAG ACCCAACTCT CTTCTGCAGC CTGCTGACCT CGTGGCCCCC

TAAGCTCAGC CTTCCCAGTC ATCTGACCCA GCTCCACCCT CAGCACCAGC

TAAACTCAGA CTTCCCAGTC ATCTGACCCA GCTCCACCCT CAGCATCAGA

TAGGTTC--- ------AGTC ATCTGACCCA GCTCCACCCT CGGCACCAAC

AGATCCTGCA ACGGTGGCGT AGG------A GGCGGAGTCC AACAGCAAGA

AGATCCTACA GCAGTGGAGT CAGACAGCAA GGATGTTTCT CCTGGCTAGA

GAATCTTGCA TAGTCAAACA CCA------- ---------- ----------

AGTGTCCCCG CCCAGAAGCC TTGGTCTCGT GAGCCAGCTG CCTCAGATGC

CGTGTGCCAG GCCAGAAGCC TCGGTCTCGT GAG------- --TCAGACGC

AGTCCCCCAG CCAAGAAGCC TTGGTCTCAG CAG------- --CCAGACCC

CTATGCTAAC CTCATGACCC GAAAAGAGAA GGACTGGGTG ATA

CTATGCTAAC CTCATGACCC GAAAAGAGAA GGACTGGGTG ATA

CTATGCTAAC CTCATGACCA GAAAAGAGAA GGACTGGGTG ATA

>Ortholog Group 387, Repeat 1

3 231

R00048827 AATCAGAAAC CACCTCCACC AGGATTCCCA CCAAGACCAC CTGCT-----

M00073689 AATCAAAGGC CACCTCCTTC AGGGTTCCAA CCAAGACCGC CTGTT-----

H00412436 CGTCAGGGAC CACCTTTGGG AGGACAGCAA TCTCAACCCT CTGCTGGTGA

---------- ---AATGGGA GCCAGCAAGG CCCACCCCCA CAAGGAGGCG

---------- ---AATGGGA GCCAGCAAGG CCCACCACCA CCAGGAGGCG

TGGGAACCAG GATGATGGCC CTCAGCAGGG ACCACCCCAA CAAGGAGGCG

GCCCACCCCC ACCAGGAGGC CCACAGCAGA AACCCCCTCA GCCTGGAAAC

GCCCACCACC ACCAGGAGTA CCACAGCCGA GACCCCCT-- ----------

GTCCACCACC TCCTCAGGGA AAGCCACAAG GACCACCCCA ACAGGGAGGC

CAACAAGGCC CACCCCCACC AGGAGGCCCA CAACAGAAAC CAACTCAGCC

---CAAGGCC CACCACCACC AGGAGGCCCA CAGCAGAGAC CC--------

CAT------C CCCCTCCTCC TCAAGGA--- ---------- ----------

TGAAAAGCCC CAAGGCCCAC CCCCACCAGG A

-------CCT CAAGGCCCAC CACCACCAGG A

----AGGCCA CAAGGACCAC CCCAACAGGG A

>Ortholog Group 388, Repeat 1

3 213

R00057317 ---------- ---------- -------CTT GACATGGAAA CTGGAAGGAA

H00398718 TTTCCCTGCA TTCCAGAAAG CCAGTTCCTG GTACTGCAGA CAGCA---AG

M00103625 ---------- ---------- -------GTT GACATGGAAG CTGGAGGGAA

AAAGCCCAAC CTAGAGAAA- ---------- -------CCC AAGGCAGTGG

TGACCCAGAC CTACATCACA AAGTTATGCC CTGGATGCCA ACGGCCCTTG

AAGGTTCAAC CTAGAGAAA- ---------- -------TCC AAAGTCGTGG

GAGGGGATGC TGGTCTTGGG CTTTACCTGG TCAGTCAAAA ACCACACCGG

ATGCCTTACC AGGT------ ------ACTG TCACTGAACA CCCTAAGCTG

GGGGGGACGC AGGTCTTGGG TTTTCCCTGG TCAGTCAAAA GACACGCCAG

GACAGAGAG- ---------- -------CAG GAACTGGAAA AGAAGACCCT

CTCCGAGTCT TGTCTGTGGA AGCCATTGAG AAACTGGAGA CAACTTTACG

GATGGAGAG- ---------- -------CAG ------GAGA AGAGGCTCCT

GCACAGA--- ---

GCACAAGCAT CTG

GCACAGA--- ---

>Ortholog Group 389, Repeat 1

3 198

R00057220 GCGCTCGACC TGGCGCTGCA GTACGTGCTC GCGCTCTACC ACGGCCAAGT

M00116234 GCGCTCGACC TGGCGCTGCA GTACGTGCTC GCGCTCTACC ACGGCCAAGT

H00395244 GCGCTGGACC TGGCGCTGCA GTACGTGCTG GCGCTCTACC ACTGCCAAGT

GTTCCTGAAG CGCTTCCTGC GTTTGCGGTA CCCACGGCAG CGG------A

GTTCCTGAAG CGCTTCTTGT GTTTGCGGTA CCCGCGGCGG CGC------A

GTTCCTGAAG CGCTTCCTGC GCTTGCGGTA CGGGCGACAG AGGCGGCGGC

CCAGGGACAC AGTACCCGCA GCCCCGGACG CCCAGATCCT TTGGGAGGCT

CCAGGGACAC GCTACCCGCA GCCCGGGACG CCCAGATCCT TTGGGAGGCT

GGAGGGGCGC GCTCCCCGTC CCTCCCGGCG CCCGGGTCCC TACTGCGGCC

GGTGGCCAGC GACGAGGACC CCAAAGTCCA AAGGGCATAG AAAGATCC

GGTGGCCAGC GACGAGGACC GGGTGGCGCA AGGGGCACAG AACGATCT

GGAGCCCGGC GGCGACGACC CCGTGGCCCC AGGGGCGCCG GGGGAGCC

>Ortholog Group 38, Repeat 1

3 198

M00053841 ---------- ---------- ---------- ---------- ----------

R00032812 CGATCCCAGT CCTTTCCAGA TGTGGAGCCT CAGCTGCCCC AAGCTCCAGC

H00171887 CGCTCTCAAT CCTTTTCGGA AGCTGAACCC CAGCTGCCCC CAGCTCCGGT

---------- ---------- ---------- ---------- ----------

CCGTGGGGGC AGCAGCCGAG AGGCTGTGCA GAGGGGCCTG AATTCA---T

CCGAGGGGGA AGCAGCCGGG AGGCTGTGCA AAGGGGACTG AATTCGTGGC

---------- ---------- ---------- ---------- ----------

CCCACCCACC TCCCCGCCAG CAGGAGCGAT CCCCACTGCA GAGTCTAGCC

CTCGCCCACC TCCACGCCAG CAGGAAAGAG CCCACTTGGA GAGTCTTGTA

---------- ---------- ---------- ---------- --------

TGCAGCAAGC CTAGCCCCCA TCTGTCAGCA GAGACCCCCA TACCAGCT

GCCAGCAGGC CCAGCCCTCA GCCATTGGCA GAGACCCCCA TCCCCAGT

>Ortholog Group 390, Repeat 1

3 198

H00377542 TACTACTATG AGCTCTGGTG GTTCTGGCTG CTCTGGACTG TCCTCATCCT

R00011719 TACTACTATG AACTCTGGTG GTTCTGGCTG CTTTGGACTG TCCTCATCCT

M00032111 TACTACTATG AACTCTGGTG GTTCTGGCTG CTTTGGACTG TCCTCATCCT

CTTTAGCTGC TGTTGCGCCT TCCGCCACCG ACGAGCTAAA CTCAGGCTGC

CTTTAGCTGT TGTTGTGCCT TCCGCCACCG AAGAGCTAAA CTCAGGCTGC

CTTTAGCTGC TGTTGTGCCT TCCGCCACCG AAGGGCTAAA CTCAGGCTGC

GGCAGCGTGA AATCAACTTG TTGGCCTATC ATGGGGCATG CCATGGGGCT

GGCAGCGTGA AATCAACTTG TTGGCTTACC ATGGGGCGTG CCACGGGGCT

GGCAGCGTGA AATCAACTTG TTGGCTTACC ATGGGGCATG CCACGGGGCT

GGTCCTTTCC CTACCGGTTC ACTGCTTGAC CTTCGCTTCC TCAGCACC

GGCCCTGTTC CAACTGGTTC ACTGCTTGAC CTTCGCCTCC TCAGCGCC

GGCCCTGTTC CAACCGGTTC ACTGCTTGAC CTTCGCCTCC TCAGCGCC

>Ortholog Group 391, Repeat 1

3 234

M00030049 GAGCAGCTGG ACGAGGAACA TCTGGAGTCG CACAGAAAGT ACAAGGAGCG

H00429249 GAGCAGCTGG ACGAGGAACA TCTGGAGTCG CACAAAAAGT ACAAGGAGCG

R00015576 GAACAGCTGG ATGAGGAACA TCTGGAGTCA CACAGAAAGT ACAAGGAGCG

CAAAGAGAAA AGGGCCCAGC AGGAGCAGCT ACAGTTGCAA CAGCAGCAGC

CAAAGAGAGA AGGGCACAGC AGGAACAGTT GCTGCTGCAG ----------

CAAAGAGAAA AGGGCACAGC AGGAACAGCT ACAGTTACAG CAACAGCAGC

AGCAACAGCT GCAACAG--- CAACAGCTAC AGCTGTCCAC GTCCCAGCCC

---------- ---------- AAGCAGTTA- -----CCCCC ATCGCAGCTC

AGCAGCAGCT ACAACAGTTG CAACAGTTGC AGCTGTCCAC CTCTCAGCTC

TGCACAGCCC CAGCT---GC TCACAAACAC CTAGATGGCA TTGAACACAC

TGCACAGCCC CTGCCTCTTC TCATGAACGC GCAAGCATGA TTGACAAAGC

TGCACAGCCC CAGCT---GC TCATGAACAC CTAGATAGCA TTGAACACAC

AAAGGAGGAT GTGGTCACTG AGCAGATTGA CTTC

AAAGGAGGAC ATTGTCACAG AGCAGATAGA TTTC

AAAGGAGGAT GTCGTCACTG AGCAGATTGA TTTC

>Ortholog Group 392, Repeat 1

3 222

M00071292 ATGGCGATAG TGATGTCTGC GGCCAAGATC TGGAGGCCGA GCCGTGGCCT

R00027995 ATGGCGGTAG CGATGTCCGC GGCCAAGATC TGGAGGCCGA GCCGTGGCCT

H00416000 ATGGCGGTAG CGATCGCTGC AGCGAGGGTC TGGCGGCTAA ACCGTGGTTT

GCGCCAGGCT GCTCTTCTCC TGTTGGGACG ATCTGGGGTT CGGGGCTTGG

GCGCCAGGCT GCCCTCTTGC TGTTGGGGCG ACCTGGGGCT CGGGGCTTGG

GAGG------ ---------- ---------- ---------- ----------

CTAGATCTCA CCCCAGCAGG CAGTTCCCAT CCCTGGACGA CAAGCCCCAG

CTAGATTCCA CCCCAGCAGG CAGTTCCCAT CCCTGGATGA CAAGCCCCAG

---------- ---------- ---TTTTCAT CTCTGGATGA CAAGCCCCAG

TTCCCAGGGG CCTCTGCAGA GTTTGTAGAC AAGCTTGAGT TCATCCAGCC

TTCCCAGGGG CCTCTGCAGA GTTTGTAGAC AAGCTTGAGT TCATCCAGCC

TTCCCAGGGG CCTCGGCGGA GTTTATAGAT AAGTTGGAAT TCATCCAGCC

CAATGTCATC TCCGGCATCC CC

CAATGTCATC TCCGGCATCC CC

CAACGTCATC TCTGGAATCC CC

>Ortholog Group 39, Repeat 1

3 129

R00016740 CGAGCTCAGA TTTTAGCTTA CAAAATGTTG GCCAGGGGCC AGCCCCTCCC

M00025862 CGAGCTCAGA TTTTAGCTTA CAAAATGTTG GCCAGGGGCC AGCCTCTCCC

H00371638 CGAGCTCAGA TTTTAGCTTA TAAAATGCTG GCCCGAGGCC AGCCCCTCCC

TGAAACTCTG CAGCTTGCAG TCCAGGGAAA AAGGACCTTG CCTGGTATG-

TGAAACTCTG CAGCTGGCAG TCCAGGGAAA AAGGACCTTG CCTGGCATGC

CGAAACGCTG CAGCTTGCAG TCCAGGGGAA AAGGACGTTG CCTGGCTTG-

---------- ----CCTCAG CAGCAGACA

AGCAGCAGCA GCAGCCTCAG CAGCAGGCT

---------- ----CCGCAG CAGCAGCCG

>Ortholog Group 39, Repeat 2

3 123

R00016740 CCACAGCAGC AGCAACCGCC AGCTCTTGTT AGCTACAATA GACCATCTGG

M00025862 CCCCAGCAGC AGCAG---CC AGCTCTTGTT AGCTATAATC GACCATCTGG

H00371638 CCGCAACCAC AGACG---CC GGCCCTTGTT AACTACAACA GACCATCTGG

CCCTGGGCAG GAGCTGCTGA TGACTGCTCA GAACACCCAG CAGAAGCTGT

CCCCGGGCAG GAGCTGCTAC TGAGTGGCCA GAGCGCTCCG CAGAAGCTGT

CCCGGGGCCG GAGCTG---- --AGCGGCCC GAGCACCCCG CAGAAGCTGC

CGGCACCCGC ACCCAGCGGC CGA

CAGCACCAGC ACCAAGCGGC CGA

CGGTGCCCGC GCCCGGCGGC CGG

>Ortholog Group 3, Repeat 1

3 186

M00124204 ATGATGACCA TGACTACGAT GGCTGACGGC TTGGAAGGCC AGGACTCGTC

R00014468 ATGATGACCA TGACTACGAT GGCTGACGGC TTGGAAGGCC AGGACTCGTC

H00428480 ATGATGACCA TGACTACGAT GGCTGACGGC TTGGAAGGCC AGGACTCGTC

CAAATCCGCC TTCATGGAGT TCGGGCAACA GCAGCCGCCG CCGCCGCCAC

CAAATCCGCC TTCATGGAGT TCGGGCAGCA ACAGCAGCCG CCGCCGCCGC

CAAATCCGCC TTCATGGAGT TCGGG----- -------CCG CCGCCGCCGC

CGCCGCCGCC GCCGCAGCCG CACTCGCAGC AGACCTCCCC GGCCATGGCA

CACCGCCACC ACCA------ ---------- ---------- ----------

CGCCGCCGCC GCCGCAGCCG CACTCGCAGC AGAGCTCCCC GGCCATGGCA

GGCGCACATT ACCCTCTGCA CTGCTTGCAC TCGGCC

NNNNNNNNNN NNNNNNNNNN NNNNNNNNNN NNNNNN

GGCGCGCACT ACCCTCTGCA CTGCCTGCAC TCGGCG

>Ortholog Group 40, Repeat 1

3 237

M00056530 TATTTTGCAG CCCCTGCTCA GCCAGATCCA GATGATCAGT TTATCATCCC

H00262719 TATTTTGCTG CCCCGGCTCA GCCGGATCCT GATGATCAGT TTATCATACC

R00003840 TATTTTGCAG CCCCTGCTCA ACCAGATCCA GATGATCAGT TTATCATCCC

CCCAGAGCTG GAAGAGGAAG TCAAAGAAAT CATGAAACAT CACCAGGAG-

CCCGGAGCTG GAAGAGGAGG TCAAAGAAAT CATGAAGCAT CACCAGGAG-

CCCAGAGCTG GAAGAGGAAG TCAAAGAAAT CATGAAACAT CACCAGGAGC

---------- ----CAGCAG CAGCTGCCAC CACCACCCCA GCCGCCACAG

---------- ---------- ---------- --CCGCCACC ACCCCCT---

AGCAGCAGCA GCAGCAGCAG CAGCAGCAGC AGCCACCACC TCCACCCCAG

CCACAGCCAC AGCCACAGCC ACAGCCACAG CCACAGCCAC AGCGGCACTT

---------- ---------- -CAGCTCCAG CCGCAGCTGC CGCGGCACTA

CCG---CCAC AGGCACAGGC ACAGGCACAG GCACAGGCAC AGAGGCCCTT

CCAAATGGAT CACCTGCCAG ACTGTTACGA TACACCG

CCAGCTGGAC CAGCTGCCAG ATTATTACGA CACGCCA

CCAGATGGAT CACCTGCCGG ACTGTTACGA CACACCA

>Ortholog Group 41, Repeat 1

3 213

R00037279 AACAGCTGTA GCATGCTGCA GCAAAACAGC GTCCAACCAG CTACCAACTG

M00106324 AACAGCTGCA GCATGCTGCA ACAGAACACT GTCCAACCAG CCGCCAACTG

H00380136 AGCAGCTGCA GCATGATGCA GCAGAGCAGC GTCCAGCCTG CTGCCAACTG

CAACATCAAG TCGCCTCAGA CCTGTGTGGT GGAAAGGCCT CCCAGTAACC

CAATATCAAG TCACCGCAGA CTTGTGTGGT GGAAAGGCCT CCCAGTAACC

CAGCATCAAG TCACCTCAGA GCTGCGTGGT GGAGAGGCCT CCCAGTAACC

CACCACCACC ACCCCCGCCG CCACCACAGC AGCCGCAGCC GCAGCCGCAG

CGCCACCACC CCCGCCGCCA CCACCACAGC AGCCGCAGCC GCCGCCACAG

CGCCACCACC GCCTCCACAG CAGCCACAGC CGCCGCCGCC ACAACCA---

CAGCAGGCAG CCCCACAGCC TCCCCCACCA CAGCCG---- ----------

CAACAGGCGG CCCCACAGCC TCCCCCACCA CAGCCGCAGC AGCAGCAGCA

---CAACCAG CACCACAGCC TCCACCACCC ---------C AGCAGCAGCC

-CAGCAGCAG CCG

GCAACAGCAG CAG

GCAACAGCAG CCG

>Ortholog Group 42, Repeat 1

3 204

R00002810 TGTGAAGAAG GAACCGACAC GGAGGAACGC TTTCTGTTCC CGGAGTACGT

H00264230 TTTGGAGAAG GTGGTGACAT GCAGGAGCGT TTTCTGTTCC CGGAGTACAT

M00045948 TATGCAGAAG GCCCGGACAC GGAGGAACGC TTTCTGTTCC CGGAGTACGT

CCCGGAGCGG ACCCCTGAGG AACAGCTGCG GGAATTGCGG GAGCTGCGGG

CCTGGAT--- ---CCGGAGC CGCAACCCAC CCGCGAAAAG CAGCTGCAGG

CCCGGAGCGG ACCCCGGAGG AACAAGTGCG GGAGTTGCAG GAGCTGCGGG

AGTTGCAGGA GAAAGAACGA CAGAGGTTGC AGCGGCGGGA GGAGCGGCTT

AGCTCGAGGA GGAGGAGCGA CAGAGGCAGC AGCGGCGGGA GGAGCGGCGA

AGTTGCAGGA GAAGGAACGA GAGAGATTGC AGCAGCGGGA GGAGCGGCTT

CAGCAGAAAC TGCGGGCAGG CCTCCGGGCT CTGCCGGTCC CGGAGCTTCC

CAGCAAAACC TACGGGCCAG GTCCCGGGAG CACCCGGTCG TGGGGCACCC

CAGCAGAAGC TGCGGGCGGG CTTCCGGACG CTGCCTGTCC CGGAGTTTCC

AGAC

GGAC

AGAC

>Ortholog Group 43, Repeat 1

3 198

R00034776 AGAGAAGCAA TTTACAACGA TGTATTGACA AAACAACAAA TGTTAATCAG

H00378969 AGAGAAGCAA TCTACAATGA TGTATTGACA AAACAACAGA TGTTAATCAG

M00109203 AGAGAAGCAA TCTACAACGA TGTATTGACA AAGCAACAGA TGTTGATCAA

CTGTGTTCAG CGAATACTCA TGAACAGACG GCTTCAGCAG CAGTATACTT

CTGTGTTCAG CGAATACTTA TGAACAGAAG GCTCCAGCAG CAGTACAATA

CTGTGTTCAG CGAATACTTA TGAATAGACG GCTCCAGCAG CAGTACACTT

TGACTTATCA GCAAGCAACA CTGAGTCACC TCATGATGCC AAAGCCTCCA

TGACTTATCA ACAAGCAACA CTGGGTCACC TCATGATGCC AAAGCCCCCA

TGACTTATCA GCAAGCAACA CTGAGTCACC TCATGATGCC AAAGCCTCCA

AATTTAATCA TGACTCCTTC CAACTACCAG CAGATTGATA TGAGAGGA

AATTTGATCA TGAATCCTTC TAACTACCAG CAGATTGATA TGAGAGGA

AATTTAATCA TGACTCCTTC TAACTACCAG CAGATTGATA TGAGAGGA

>Ortholog Group 44, Repeat 1

3 198

H00198939 AACGAGTACT CCTCAGTGGT CCAGCCGGTG CAGCTGGCCT TCCAGCAGCA

R00017424 AATGAGTACT CATCAGTGGT GCAGCCTGTG CAGCTGGCCT TCCAGCAGCA

M00078469 AATGAGTACT CGTCAGTGGT GCAGCCTGTG CAGCTGGCCT TCCAGCAGCA

GATCCAGACC CTCAAGACGC AGCACGAGGA GTTTGTCACC AGCCTGGCCC

GATCCAGAGC CTCAAGACAC AGCATGAGGA GTTTGTCAGC AGCCTGGCCA

GATCCAGAGC CTCAAGACAC AGCATGAAGA GTTCGTCAGC AGCTTAGCCA

TCCAGATGCC GCAGATGGAG GCTGAAGTCA AGGCCACGCC TCCACCGCCT

TTCAGCTGCC TCCGATGGAA GCCGACGTGA AGGCCACGCC CCCACCGTCA

TTCAGCTGCC TCAGATGGAA GCAGATGTGA AGGCCACACC CCCACCACCA

GCTCCACCCC CGGCCCCAGC ACCTGCCCCT GCCATCCCGC CCACCACC

GCACCACCAC CAGCCTCAGC ACCTGCCCCT ACCATCCCAC CAACTACC

GCACCACCAC CAGCCTCAGC ACCTGCCCCC ACCATCCCAC CAACTACC

>Ortholog Group 45, Repeat 1

3 198

H00202556 AATGTAATAA ATGTACCTGG AGAAAAACGT ACTGAAAATG GGGTTGGGAA

M00062464 AGTGTAGTAA ATGTACCTGG AGAAAAGCGC ACTGAAAATG GGGTTGGGAA

R00016895 AACGTTATGA ATGTTCCTGG AGAGAAGCAC ACTGAAAATG GGGTTGGGAA

TCCACGTGTT GAACTTACCC TCTCAGAGCT CCAAGATATG GCAGCTAGGA

TCCACGTGTT GAACTTACCC TCTCAGAACT TCAAGATATG GCAGCCAGGA

CCCACGTGTT GAACTTACCC TCTCAGAACT TCAAGACATG GCGGCCAGGA

TTGAAAATCA GCAGCAGATG TTGGTTGCCA AGGAACAGCG TTTACATTTT

TTGAAAACCA GCAGCAAATG TTGGTTGCCA AGGAACAGCG TTTACATTTT

TTGAGAGTCA GCAGCAAATG CTGGTTGCCA AGGAACAGCG TTTACATTTC

CTAAAGCAAC AGGAGCGCCG TCAGCAGCAG TCTATTTCTG AAAATGAA

CTAAAGCAAC AGGAGCGCCG CCAGCAGCAG TCCGTTTCTG AAAATGAA

CTAAAGCAAC AGGAGCGCCG CCAGCAGCAG TCCATTTCTG AGAATGAG

>Ortholog Group 46, Repeat 1

3 222

R00053998 CTTCAGCCCA CACCACAAGT GCCCCCGGTG CCACAGGCAC AGCCATCCCA

H00395705 CTTCAGCCTA CACCACAAGT GCCGCAAGTA CAGCAGTCAC AGCCGTCTCA

M00098616 CTTCAGCCTA CACCACAAGT GCCCCAGGGG CCACAGTCCC AACCATCTCA

AGGTTCTGAA GCAGCGCAGC CGCAGCAGAA GGATGCACAG CAGCAGCAGC

AAGCTCCGAA CCATCCCAGC CCCAGCAGAA GGACCCC--- ----------

AGGCTCAGAA GCAGCCCAGC CTCCACAGAA GGACTCG--- ----------

AGCAGCAGCA GCAGCAGCAG CAGCAAGCAC AACAGCCCAA GAAACCATCC

---------- -CAGCAACCA GCCCCAGCCC AACAGCCCAA GAAACCCTCT

--------CA GCAGTCAGCC CAGCAAGCAC AACAGCCCAA GAAACCATCC

CCTCAGCCCA GTCCTCCGCG ACAGGCCAAG CGTGCCGTG- ----------

CCGCAGCCCA GTTCTCCCCG ACAGGTTAAG CGAGCCGTGG TGAGTGGGCT

CCTCAGCCCA GTCCTCCCCG ACAGGCCAAG CGCGCCGTG- ----------

---------- ---------- --

AAAGCAGGCC CTGGGTAGAG CA

---------- ---------- --

>Ortholog Group 47, Repeat 1

3 207

M00003404 CAGGGTCCGG GGACCAACTT TGGGCCCTTC CCAGGGCAAG GTGGAGGCGG

H00205061 CAGGGTCCCG GGGCCAACTT TGTGTCCTTC GTAGGGCAGG CCGGAGGCGG

R00025570 CAGGGTCCGG GGGTCAACTT TGGGCCCTTC GCAGGGCAAG CTGGAGGCGG

CAGCCCGGCC GGCCAACAGC CGCCTCAGCA GCCTCAGTTA TCG------C

CGGCCCGGCG GGTCAGCAGC TGCCCCAGCT GCCTCAGTCA TCGCAGCTTC

CAACCCGGTC GGGCAGCAGC CACCTCAGCT GCCTCAGTTA TCGCAGCAGC

AGCAACAGCA GCAGTCGCTT TTCGCGGCGG GCGGGCTCCC GGCCCGGCGG

CTCAGCCGCC GCAGCCGCCT TTCCCGGCGG GTGGGCCTCC GGCCCGGCGG

CT-------- ---------- TTCCCGGCGG GCGGGCTCCC GGCCCGGCGG

GGCGGAGCGG GGCCCGGTGG GACTGGCGGA GGCTGGAAGC TGGCGGAGGA

GGAGGAGCG- --------GG GGCTGGTGGG GGCTGGAAGC TGGCGGAGGA

GGCGGAGCAG GGCCCGGTGG GACTGGCGGA GGCTGGAAGC TGGCGGAGGA

AGAGTCC

AGAGTCC

AGAGTCC

>Ortholog Group 48, Repeat 1

3 108

M00091595 GTAAGCCAGC CTCAGCCTGC CAACTCTGAT AACGGCACTT CCACAGCAAC

R00059607 GTAAGCCAGC CTCAGCCTGC CAACTCCGAT AACGGCACTT CCACAGCTAC

H00379144 GTAAGCCAGC CTCAGCCTGC CAACTCTAAT AACGGCACTT CCACAGCAAC

CAGCACTAAT AATAATGCCA AGCGAGCTAC AGCCAGCAAT CAGCAGCCGC

CAGCACTAAT AATAATGCCA AGCGAGCTAC AGCCAGCAAT CAGCAGCCGC

CAGCACTAAT AATAATGCCA AGCGAGCTAC AGCCAACAAT CAGCAG----

CGCCACCG

CGCCGCCG

-----CCA

>Ortholog Group 48, Repeat 2

3 12

M00091595 ---------- --

R00059607 CAACAAGAGC AG

H00379144 CCACAGCAGC AG

>Ortholog Group 48, Repeat 3

3 111

M00091595 CAGCAGCAGC CGCAGCAGCA GCAGCCACAA GCCTTGCCTC GGTATCCACG

R00059607 CAGCAGCAGC AGCAGCAGCA GCAGCCACAA GCCTTGCCTC GGTATCCTCG

H00379144 CAGCCGCAGC CGCCACAGCA GCAGCCACAG GCCTTGCCTC GGTATCCTCG

TGAAGTACCT CCACGATTTC GCCACCAGGA ACACAAACAG CTTCTGAAGA

TGAAGTACCT CCACGATTTC GCCACCAGGA ACACAAACAG CTTCTAAAGA

TGAAGTACCT CCACGATTTC GCCACCAGGA ACACAAACAG CTTCTAAAGA

GGGGTCAGCA T

GGGGTCAGCA T

GGGGTCAGCA T

>Ortholog Group 49, Repeat 1

3 198

M00109121 TCCCGAGGCA TCGAGGCCTG CACCAACGCT GCTGACGCCC TGCTGCAGTG

H00370695 TCCAGAGGCA TTGAGGCCTG CACCAATGCT GCTGATGCCC TTCTGCAGTG

R00056565 TCCAGAGGCA TTGAGGCCTG CACCAATGCT GCTGACGCCC TTCTGCAGTG

CATGAACGTT GCTCTTCGCC CAGGCCATGA CATGCTCCTG GCCGTCAAGC

CATGAATGTA GCTCTTCGAC CAGGCCATGA CTTGCTTCTG GCAGTCAAAC

CATGAACGTC GCCCTTCGAC CAGGCCATGA CATGCTCCTG GCCATCAGGC

GCTTCAGTGA CCTGAGGGAA CATTTCGCCC GGAGACTGGC CAGCCATCTC

GATTCAGTGA TTTGCGAGAG CTTTTTGCCC GGAGACTGGC CAGTCACCTC

GCTTCAGCGA GCTGAGGGAG CATTTTGCCC GGAGACTGGC CAGTCATCTC

AACAATGTTT TTGTTCAACA GGGTCATGAT CAGAGTTCAA CTCTTGCT

AACAATGTTT TTGTTCAACA GGGTCATGAT CAGAGTTCGA CTCTTGCC

AACAATGTTT TTGTTCAACA GGGTCACGAT CAGAGCTCAA CCCTTGCT

>Ortholog Group 4, Repeat 1

3 198

H00428039 CTGGTAGAGA TGACGGGCCT CAGTCCCCGT GTGATCCGGG TCTGGTTTCA

M00044879 CTAGTGGAGA TGACGGGCCT CAGTCCCAGA GTCATCCGAG TGTGGTTTCA

R00017305 CTAGTGGAGA TGACCGGCCT CAGTCCCCGA GTCATCCGGG TCTGGTTTCA

AAACAAGCGG TGCAAGGACA AGAAGCGAAG CATCATGATG AAGCAACTCC

AAACAAGCGG TGCAAGGACA AGAAACGCAG CATCATGATG AAGCAGCTCC

AAACAAGAGG TGCAAGGACA AGAAACGCAG CATCATGATG AAGCAGCTCC

CCAATGACAA AACTAATATC CAGGGGATGA CAGGAACTCC CATGGTGGCT

CCAACGACAA AACTAATATC CAGGGGATGA CAGGAACTCC CATGGTGGCT

CCAACGACAA AACTAATATC CAGGGGATGA CAGGAACTCC CATGGTGGCT

GCCAGTCCAG AGAGACACGA CGGTGGCTTA CAGGCTAACC CAGTGGAA

GCTAGTCCGG AGAGACATGA TGGTGGTTTA CAGGCTAACC CAGTAGAG

GCTAGTCCGG AGAGACATGA TGGTGGTTTA CAGGCTAACC CAGTTGAG

>Ortholog Group 50, Repeat 1

3 198

M00111212 AGCACAGAAG CAGTTTTGAA CAATCGGTTC ATTCGAGTCC TGTGGCATAG

R00035970 AGCACAGAAG CTGTTTTGAA CAATCGGTTC ATTCGAGTCC TATGGCACAG

H00265271 AGCACAGAAG CAGTTCTAAA CAACCGATTC ATTCGAGTCT TGTGGCATAG

AGAGAACAAT GAGCAACCAG CACTCCAGTC CTCAGCACAG ATTCTCCTGA

AGAGAACAAT GAGCAACCAG CACTCCAGTC CTCAGCACAG ATTCTCCTGA

GGAAAATAAT GAGCAACCGA CACTACAGTC CTCAGCACAG CTGCTCCTGA

CCCTGAGTCA CCTTTCACAG CAACACCATA GCCTCCCACA GCATCTTCAT

CCCTGAGTCA CCTTTCACAG CAGCACCACA ACCTACCACA GCATCTTCAT

CACTTAGTCA CCTCTCACAG CAGCACCATC ACCTGCCACA GCATCTACAT

CCGCAGCAGG TGATGGTAAC CCAGTCTTCC CCCTCATCGG TCCATGGA

CCGCAGCAGG TGATGGTAAC CCAGTCTTCC CCCTCTTCAG TCCACGGA

CAGCAGCAGG TGCTAGTGGC CCAGTCTGCT CCTTCAACAG TGCACGGA

>Ortholog Group 51, Repeat 1

3 147

M00031090 ---------- ---------- ---------- ---------- ----------

H00382767 ATGCAGCGGC GCGGCGCGGG GCTCGGGTGG CCGCGG---- -----CCCCC

R00006610 ATGCAGTGGC GCGGCGCGGG GCTTTGGTGG CCACGGCGGC GACAGCCGCC

---------- ---------- ---------- ---------- ----------

GCCGCTCGCG GTCGGCCCCC GGGCCGCAGC CATGGTC--- CCGAGTGGCG

GCCGCCGGCT TTCGGTCCCC GGGCCGCAGC CATGGTCCCC CCGAGCGGCG

---------- ---------- ---------- ---------- -------

GCGTCCCCCA GGGCCTCGGC GGCCGCTCTG CCTGCGCGCT GCTCCTG

GTGTCCCCCT GAGCCTCGGC GGCCGCCCTG CCAGCGCGCT GCTCTTT

>Ortholog Group 52, Repeat 1

3 105

R00011641 ATTCAAACGC AACATCACGT TGGCCAAACT CAGCTCCAAC TTCTACAACA

M00026284 ATTCAAACAC AACATCATGT TGGCCAAACT CAGCTCCAAT TTTTA---CA

H00430497 ATTCAAACCC AACATCACGT TGGTCAAACT CAACTCCAGA TACTACAGCA

ACAAGCACAG CAGTACCAAG CTGCCCAGCC TCAGCTGCAG CCT---CCAC

ACAAGCACAA CAGTACCAAG CTGTACAGCC CCAGCTGCAG CCT---CAAC

ACAAGCACAA CAATACCAAG CCACACAGCC CCAGCTGCAG CCTCAAAAAC

CCCCA

CCCTA

CACCA

>Ortholog Group 52, Repeat 2

3 105

R00011641 CCGCCACCAA GCAAACTGTT GAAACAAGAG CAAGGTAGCC TGGCGAATAC

M00026284 CCACAACCAA GCAAACTGTT GAAACAAGAG CAAGGTAGCC TGGCAAGTAC

H00430497 CCTCCAGCAA GCAAATTATT GAAACAAGAG CAAAGTAACA TAGTGAGTGC

CGACTGCCAG CTCATGAAGG ATGCGCCATC TTACAAGGAG GCAGAAGAGA

AGACTGCCAA CTCATGAAGG ACATGCCATC TTACAAGGAG GCAGAAGAGG

AGACTGCCAA ATCATGAAGG ATGTGCCATC TTATAAGGAG GCAGAAGATA

TGACT

TGACT

TTTCT

>Ortholog Group 53, Repeat 1

3 198

R00006137 CAGATTGACC CAAAGAAGTT GAAAAGGAAG CAAAGTGTGA ATGTTTCTCT

H00308533 CAAATTGACC CAAAGAAGTT GAAAAGGAAG CAAAGTGTGA ATATTTCTCT

M00021379 CAGATTGACC CAAAGAAGTT GAAAAGGAAG CAAAGTGTGA ACATTTCTCT

TTCGGGATGC CACCCTGCCC CTGAAGGTTA TTCCCCAACA CTGCAGTGGG

TTCAGGATGC CAACCCGCCC CTGAAGGTTA TTCCCCAACA CTTCAATGGG

TTCCGGATGC CAGCCTGCGC CTGAAGGTTA CTCTCCAACA CTTCAGTGGG

TGATACAGTT TTCGAGTGTT CGACAGAGTG TGCACAAGCA TAGAAATCAC

TGGCACAGTT TTCAACTGTT CGACAGAATG TGAACAAACA TAGAAGTCAC

TGGCACATTT TTCAACTGTT CGACAGAGTG TACACAAGCA TAGAAATCAC

TGGAAATCTC AACAGTTGGA TAGTAATGTG ACAATGCCAA AATCTGAA

TGGAAATCAC AACAGTTGGA TAGTAATGTG ACAATGCCAA AATCTGAA

TGGAAATCAC AACAGTTGGA CAGTAATGTG GCAATGCCAA AATCTGAA

>Ortholog Group 54, Repeat 1

3 99

H00263205 CAGCCGCCTC CTGGGACCTC GGGGATGGCC CCTCACAGCA TGGCTGTCGT

M00012259 CAACCACCCC CTGGAACCTC TGGAATGGCC CCTCATGGCA TGGCTGTGGT

R00002568 CAGCCACCCC CTGGAACCTC TGGAATGGCC CCTCATGGCA TGGCTGTGGT

GTCTACGGCA ACTCCACAGA CCCAGCTGCA GCTCCAGCAG GTGGCGCTG

GTCTACAGCA ACTCCACAGA CTCAGCTGCA GCTCCAGCAA GTGGCATTG

GTCTACAGCA ACTCCACAGA CTCAGCTGCA GCTCCAGCAA GTGGCATTG

>Ortholog Group 54, Repeat 2

3 3

H00263205 GCG

M00012259 GCA

R00002568 GCA

>Ortholog Group 54, Repeat 3

3 39

H00263205 CTA------- -----TTCCA GGCTCAGCAG AGTGCCATG

M00012259 CTGCAGCAAC AGCAGTTCCA GGCACAACAG AATGCCATG

R00002568 TTGCAGCAGC AGCAATTCCA GGCACAACAG AATGCCATG

>Ortholog Group 54, Repeat 4

3 24

H00263205 CAGCAGCAGT TCCAAGCAGT AGTG

M00012259 CAGCAACAGT TCCAAGCAGT AGTG

R00002568 CAGCAGCAGT TCCAAGCAGT AGTG

>Ortholog Group 54, Repeat 5

3 18

H00263205 CATCTAATTA AATTGCAT

M00012259 CACCTGATTA AGTTGCAT

R00002568 CACCTGATTA AGTTGCAT

>Ortholog Group 54, Repeat 6

3 30

H00263205 CAAAATCAGC AACAGATACT GCAGCGAATA

M00012259 CAAAGCCAGC AACAACAGCT GCAGAGGATG

R00002568 CAAAGCCAGC AACAACAGCT GCAGAGGATG

>Ortholog Group 54, Repeat 7

3 111

H00263205 CAGCTGCAGC TCGCTTTGCA GGCCCAGCCA CCAATTCAGC AGCCACCGAT

M00012259 CAGTTGCAGC TGGCTTTGCA GGCCCAGCCA CCAATGCAGC AGCCATCAAT

R00002568 CAGTTGCAGC TGGCTTTGCA GGCCCAGCCA CCAATGCAAC AGCCACCAAT

GCAGCAGCCA CAGCCTCCGC CCTCCCAGGC TCTGCCCCAG CAGCTGCAGC

GCAGCAGCCA CAGCCTCCCC CTTCTCAGGC CCTACCCCAG CAGCTGTCAC

GCAGCAGCCA CAGCCTCCTC CTTCTCAGGC CCTACCCCAG CAGCTGTCAC

AGATGCATCA C

AGCTGCATCA T

AGATGCATCA T

>Ortholog Group 54, Repeat 8

3 201

H00263205 GCGCAAGCTC TCCCTGGACA AATGTTGTAT ---ACCCAAC CACCACTGAA

M00012259 GCACAAGCCC TTCCTGGACC GATGCTGTAT GCTGCCCAAC AGCAGCTGAA

R00002568 GCACAAGCCC TGTCTGGACC AATGCTCTAT GCTGCCCAAC AGCCACTGAA

ATTTGTCCGA GCTCCGATGG TGGTGCAGCA GCCCCCAGTG CAGCCCCAGG

ATTTGTCCGT GCTCCGATGG TGGTCCAGCA GCCGCAAGTG CAGCCCCAGG

ATTTGTCCGT CCTCCGATGG TGGTCCAGCA GCCGCAGGTG CAGCCCCAGG

TGACAGCAGT ACAGACAGCT CAGGCTGCCC AGATGGTGGC TCCCGGAGTC

TGGCAGCAGT GCAGGCAGCA CAGTCTGCCC AGATGGTAGC TCCCGGCGTC

TGGCAGCAGT GCAGACAGCA CAGTCTGCCC AGATGGTAGC TCCCGGCGTT

CAGATGATCA CGGAAGCCTT GGCCCAAGGT GGGATGCACA TAAGAGCCCG

CAGATGATTG CTGAAGCCTT GGCCCAAGGC GGGATGCACG TAAGAGCCCG

CAGATGATTG CTGAAGCCTT GGCCCAAGGC GGGATGCACG TAAGAGCCCG

G

G

G

>Ortholog Group 55, Repeat 1

3 198

H00215793 AACCCCAATG ACCCTTACCA TGCCTACTAC CGCCACAAGG TCAGCGAGTT

R00008355 AATCCCAATG ACCCTTACCA TGCCTACTAC CGCCACAAGG TCAGCGAGTT

M00002198 AATCCCAATG ACCCTTACCA TGCCTACTAC CGCCACAAGG TCAGCGAGTT

CAAGGAAGGG AAGGCTCAGG AGCCGTCCGC CGCCATCCCC AAGGTCATGA

CAAGGAGGGG AAGGCTCAGG AACCCTCAGC TGCCATCCCC AAGGTTATGG

CAAGGAGGGG AAGGCTCAGG AGCCCTCAGC TGCCATCCCC AAGGTTATGG

CCACCCAGCA GCAGCTGCCC CAGAAGGTCC AAGCCCAAGT AATCCAAGAG

CCACACAACA ACAGCTGCCC CAGAAGGTCC AAGCCCAGGT GATCCAAGAG

CTACGCAACA GCAGCTGCCC CAGAAGGTCC AAGCCCAGGT GATCCAAGAG

ACCATCGTGC CCAAAGAGCC TCCTCCTGAG TTTGAGTTCA TTGCTGAT

ACCATAGTAC CGAAGGAGCC CCCTCCTGAG TTCGAGTTCA TCGCTGAC

ACCATAGTAC CGAAGGAGCC CCCTCCTGAG TTTGAGTTCA TCGCTGAT

>Ortholog Group 56, Repeat 1

3 255

M00124930 ---------- ---------- ---------- ---------- ----------

R00012699 GCACAGCACT CTGCCCTTGG TGGCGTGTCT CATTATCAGC AGGATTACAC

H00352463 GCACAGCACT CTGGCCTTGG CGGTGTGTCA CATTATCAGC AGGATTACAC

---------- ---------- ---------- ---------- ----------

AGGGCCTTTC TCTCCTGGGA GTGCTCAGTA TCAACAGCAG GCCTCTAGCC

TGGGCCTTTC TCTCCAGGGA GTGCTCAGTA CCAACAGCAG GCTTCCAGC-

---------- ---------- ---------- ---------- ----------

AACAACAGCA GCAGCAGCAG CAGCAGCAAC AGCAACAGCA GCAACAGCAA

---------- ---------- ---------- ---------- ----------

---------- ---------- ---------- ---------- ----------

CAGCAGGTAC AGCAGTTGAG ACAACAGCTT TACCAATCAC ATCAGCCTCT

------GTCC AGCAGTTGAG ACAACAGCTT TACCAGTCCC ATCAGCCCCT

---------- ---------- ---------- ---------- ----------

GCCACAAGCC ACTGGACAGC CAGCCTCTGG CTCATCCCAT CTACAACCAA

GCCACAGGCC ACTGGCCAAC CAGCATCCAG CTCATCCCAT CTACAGCCAA

-----

TGCAG

TGCAG

>Ortholog Group 56, Repeat 2

3 99

M00124930 ---------- ---------- ---------- ---------- ----------

R00012699 AATGCACAGG CTTATGGGAC ACAGTCAAAT TATAGCTATC AACCTCAGTC

H00352463 AATGCACAGG CTTATGGAAC ACAATCCAAT TACAGCTATC AGCCTCAATC

---------- ---------- ---------- ---------- ---------

TATGAAAAAT TTTGAACAGG CAAAGATTCC ACCAGGATCC CAGCAGGGG

TATGAAGAAT TTTGAACAGG CAAAGATTCC ACAAGGGACC CAACAGGGG

>Ortholog Group 56, Repeat 3

3 99

M00124930 ---------- ---------- ---------- ---------- ----------

R00012699 CATCCCCCCC AGCATGTGAT GCAGTACACA AATGCTGCCA CCAAGCTGCC

H00352463 CACCCTTCTC AGCATGTGAT GCAGTATACT AACGCTGCCA CCAAGCTGCC

---------- ---------- ---------- ---------- ---------

TCTGCAAAGC CAGGTGGGGC AGTACAACCA GCCTGAGGTT CCTGTAAGG

CCTGCAAAGC CAAGTGGGGC AGTACAACCA GCCTGAGGTT CCTGTGAGG

>Ortholog Group 56, Repeat 4

3 198

M00124930 CCACCTCCTA AGAGGTCCAC AGAAATGCAG AGCAAAGTCA AGGTTCGGCA

R00012699 CCACCTCCTA AGAGGTCCAC AGAAATGCAG AACAAGGTCA AGGTTCGGCA

H00352463 CCACCTCCTA AGAGGGCCAC AGAAATGCAG AGCAAAGTTA AGGTACGGCA

CAAAAGTGCT TCTAAGGGTT CTAAAACTGA CACTGAGGAG GAGGAGCAAA

CAAAAGCGCT TCTAATGGTT CTAAAACTGA CACCGAGGAG GAGGAGGAGA

CAAAAGTGCT TCTAATGGCT CCAAGACGGA CACTGAGGAG GAGGAAGAGA

AGGAGCAGAG GAGCCTGGCT GCTCATCCTA GGTTCAATCT GCGCCAGCGC

AGGAGCAGAG GAGCCTAGCT GCTCATCCTA GGTTCAAGCG GCGCCACCGC

AGGAGCAGAG AAGCCTGGCC GCACACCCCA GGTTTAAGCG GCGCCACCGC

TCCAAAGACT CTGCTGGAGG TCCTCGGTCC CTGTCCAGGG GTCGCCCT

TCCGAAGACT GTGGTGGAGG TCCTCGGTCC CTGTCCAGGG GGCTCCCT

TCGGAAGACT GTGGTGGAGG CCCTCGGTCC CTGTCCAGGG GGCTCCCT

>Ortholog Group 57, Repeat 1

3 183

H00216034 ATGTACAGGT TTTCCAGGGC AGCCTTGTGG ATTGGGACCA CTTCCTTTAT

M00023062 ATGTACAGAT TTTCCAGGGC AGCTTTGTGG ATTGGGACCA CTTCCTTCAT

R00019323 ATGTACAGAT TTTCCAGGGC AGCTTTGTGG ATTGGGACCA CTTCCTTCAT

GATCCTGGTT CTTCCCGTTG TCTTTGAGAC GGAGAAGTTG CAAATGGAGC

GATCCTGGTT CTTCCCGTTG TCTTTGAGAC AGAAAAGTTG CAGATGGAGC

GATCCTGGTT CTTCCTGTTG TCTTTGAGAC AGAAAAGTTG CAAATGGAGC

TGCAGCAGCG GCAGATACTT CTAGGACCTA ACACAGGGCT CTCAGGAGGA

TGCAGCAACG GCAGATACTT TTAGGGCCTA ACACAGGGCT GTCAGGAGGA

TGCAGCAAAG GCAGATACTT TTAGGGCCTA ACACAGGACT GTCAGGAGGA

ATGCCAGGGG CTCTACCCTC ACTTCCTGGA AAG

ATGCCAGGGG CTCTACCTCC ACTTCCTGGA AAG

ATGCCAGGGG CTCTACCTCC ACTTCCTGGA AAG

>Ortholog Group 58, Repeat 1

3 198

M00086835 TTCCTGTGTG TGGTGTCTAA GGAGCTCCAC AGCTCCCCAC ACGGCCTGAG

H00385096 TTCCTGTGTG TGGTGTCCAA GGAGCTCCAC AGCACCCCAA ACGGGCTGAG

R00000247 TTCCTGTGTG TGGTGTCTAA GGAACTCCAC AGCTCCCCCC ACGGCCTGAG

CTCCGAGTCC ACTCGCAAGG CCAAGAGCAC GGACGAGCAG CTGGAGGAG-

CTCAGAGTCC AGCCGCAAAA CCAAGAGCAC GGAGGAGCAG CTGGAGGAGG

TTCGGAGTCC ACTCGCAAGA CCAAGAGCAC GGACGAGCAG CTGGAGGAG-

-----GAGGT GGAGGAGGTG GAGGTGGCCC AGGTGCAGGT GGAGGCGGAT

AGGAGGAGGT GGAGGAGGTG GAGGTGGAAC AGGTGCAGGT GGAGGCAGAT

-----GAGGT GGAGGAGGTG GAGGTGGCCC AGGTGCAGGT GGAGGCGGAT

GCCCAGGAGA AAGCTATTAC AAGCCAGAGG ---------- --------

GCACAGGAGA AAGCCCAGTC ATCTCAGGAT CCCGCTAACC TTTTCTCC

GCCCAGGAGA AAGCTATTAC AAGCCAGAGG ---------- --------

>Ortholog Group 59, Repeat 1

3 198

H00263253 TTGGGCCAGG TAGGTATCAG CCCACTCAAA CCAGGCACTG TGTCTCAACA

M00066789 TTGGGCCAAG TGGGTGTGAG CCCTCTCAAG CCAGGCACTG TGTCTCAACA

R00000206 TTGGGCCAAG TAGGTGTGAG CCCTCTCAAG CCAGGCACTG TGTCTCAACA

AGCCTTACAA AACCTTTTGC GGACTCTCAG GTCTCCCAGC TCTCCCCTGG

AGCCTTACAA AACCTTTTGC GGACTCTCAG GTCTCCCAGT TCTCCCTTAG

AGCCTTACAA AACCTTTTGC GGACTCTCAG GTCTCCCAGT TCTCCCTTAG

TGCTTAGTAT CCTTCACGCC AACCCCCAGC TGTTGGCTGC ATTCATCAAG

TGCTTAGTAT CCTTCATGCC AACCCCCAAC TGTTGGCTGC ATTCATCAAG

TGCTTAGTAT CCTTCATGCC AACCCCCAAC TGTTGGCTGC ATTCATCAAG

CAGCGGGCTG CCAAGTATGC CAACTCTAAT CCACAACCCA TCCCTGGG

CAGCGGGCTG CCAAGTATGC CAACCCTAAT CCACAGCCTC TCCCTGGA

CAGCGGGCTG CCAAGTATGC CAACTCTAAT CCACAGCCTC TCCCTGGA

>Ortholog Group 59, Repeat 2

3 138

H00263253 CCCATGGGAG GGATGAGCCC CCAGGCTCAG CAGATGAACA TGAACCACAA

M00066789 CCCATGGGAG CAATGAGTCC CCAAGCTCAG CAAATGAACA TGAATCACAA

R00000206 CCCATGGGAG GGATGAGTCC CCAAGCTCAG CAAATGAACA TGAATCACAA

CACCATGCCT TCACAATTCC GAGACATCTT GAGACGACAG CAAATGATGG

TACCATGCCT TCACAGTTCA GAGACATCTT AAGACGGCAG ATGATG---G

CACCATGCCT TCACAGTTCA GAGACATCTT AAGACGTCAG ATGATG---G

GAGCAGGGCC AGGAATAGGC CCTGGAATGG CCAACCAT

GAGCAGGGCC AGGAATCGGC CCTGGAATGG CC------

GAGCAGGGCC AGGAATTGGC CCTGGAATGG CCAACCAC

>Ortholog Group 59, Repeat 3

3 153

H00263253 AACCAGTTCC AGCAACCCCA AGGAGTTGGC TACCCACCA- ----------

M00066789 AACCAGTTCC AGCAGCCCCA AGGAATTGGC TATCCACCG- --------CA

R00000206 AACCAGTTCC AGCAGCCCCA AGGAATTGGC TATCCACCCC AACAGCAGCA

----CGGATG CAGCATCACA TGCAACAGAT GCAACAAGGA AATATGGGAC

GCAGCGAATG CAGCATCACA TGCAGCAAAT GCAGCAAGGA AATATGGGAC

GCAGCGAATG CAGCATCACA TGCAGCAAAT GCAGCAAGGA AATATGGGAC

AGATAGGCCA GCTTCCCCAG GCCTTGGGAG CAGAGGCAGG TGCCAGTCTA

AAATGGGTCA GCTTCCCCAG GCTCTGGGGG CTGAGGCAGG AGCCAGTCTA

AGATGGGTCA GCTTCCCCAG GCTTTGGGGG CTGAGGCGGG AGCCAGTCTA

CAG

CAG

CAG

>Ortholog Group 5, Repeat 1

3 198

M00113040 CACCAGCAAG TCGTGGCC-- -ACTAGCCTG AGTCCACAGA ACCACCCGAC

R00022507 CACCAGCAAG TCGTGGCC-- -ACTAGCCTG AGTCCACAGA ACCACCCGGC

H00419465 CACCAGCAGC AGATGGCCCC CAGTACCCTG AGCCAGCAGA ACCACCCCAC

TCAGAACCAA CCCACAGGGC TCATGAGTGT GCCCAATGCA CTGACCACTA

TCAGAACCCA CCCACGGGGC TCATGAGTGT GCCCAATGCA CTCACCACGA

TCAGAACCCA CCCGCAGGGC TCATGAGTAT GCCCAATGCG CTGACCACTA

AACTGCGGCT TCAGAGGATC CAGATGGAGA GAGAGAGGAT TAGGATGCGT

AACTGCGGCT TCAGAGGATC CAGATGGAGA GAGAGAGGAT CAGGATGCGT

AACTGCGGCT TCAGAGAATC CAGATGGAGA GAGAAAGGAT TCGAATGCGC

CAAGAGGAGC TCATGAGGCA GGAAGCTGCC CTCTGCCGAC AGCTCCCC

CAAGAGGAGC TCATGAGGCA GGAAGCTGCT CTCTGCCGAC AGCTCCCC

CAAGAGGAGC TCATGAGGCA GGAAGCTGCC CTCTGTCGAC AGCTCCCC

>Ortholog Group 60, Repeat 1

3 198

M00118956 GCCAGCGTTC AGAGTAAGCA AGAGAAAGTG CACTGTCATG ACCACGACAA

H00346359 ACAAGCATTC AGAGGAAACA AGAGAAATTA CATTGTCATG ATCACGAAAA

R00011056 GCAAGCATTC AGCACAAACA GGAGAAATTA CACTGCCATG ATCACGAGAA

GCAGATGAAC GCGTTCATGG AGCAGCATAT CAGGCACCTT GAAAAACTAA

GCAAATGAAT GTGTTTATGG AGCAGCACAT AAGGCATCTT GAAAAGTTAA

ACAGATGAAC GTGTTTATGG AGCAGCACAT CAGGCACCTT GAAAAATTAA

TAGACATTCA GACCCATTTT ATTGATGCTG CACTCAAGGC TAGCAGTCTT

TAGATATTCA GACTCATTTT ATTAGTGCTG CACTCAAGAC TAGTAGTTTT

TGGATATTCA GACCCATTTC ATTGATGCTG CACTCAAGGC TAGTAGTCTT

CAA---CTTG GCATGTCCAC GTCCAGAGCA GTGGGGAAGT ATTCTGGA

CAGCCTGTTA GTATGCCCTC CTCCAGAGCA GTGGAAAAGT ATTCCGTA

CAA---TTTG GCTTGTCCTC TTCCAGAGCA GAGGACAAGT ATTCTGGA

>Ortholog Group 61, Repeat 1

3 198

R00047580 AAGTTAACAT ACGGTTCTTC TCGCCCTTCT ATAGAAATTT ATCGACCACC

H00251038 ACACTTACAT ATGGTTCTTC TCGCCCTTCT ATTGAAATTT ATCGACCACC

M00105732 ACACTTACAT ACGGTTCTTC TCGCCCTTCT ATTGAAATTT ATCGACCACC

TGCAAGTAGA AATGCAGACA CTGGTACTCA TTTAAACAGG CCGCAACTTA

TGCAAGTAGA AATGCAGATA GTGGTGTTCA TTTAAACAGG TTGCAATTTA

TGCAAGTAGA AATGCAGACA CTGGTACTCA CTTAAACAGG CTGCAACTTA

GCAGTACCCA TACTGCCAAG CAGCTGGATG GACAGAGCAG CCAGGTGTAT

ATAGTATTCA TGCTGCCAAG CAGCTTGATA TGCAGAGTAG TTGGGTATAT

GCAGTGCTCA CGCTGCCAAG CAGCTGGATG TACAAAGCAG CCAGGTATCC

GAAGCAGGAC GGTTGTGTGA ACCAGAAGTA CTTGGCAGCG TAGAAGAT

GAAACAGGAC GTTTGTGTGA ACCAGAGGTG CTTAACAGCT TAGAAGAA

GAAGCAGGAC GGTTGTGTGA GCCACCAGTG CTTAGCAGCG TAGAAGAC

>Ortholog Group 62, Repeat 1

3 99

M00104892 CCGAAGGAGA CTGTCCCAGC TGTGCAGCGG GTCGTGTGGA ACGCATCAAG

H00420095 CCGAAGGAGA CTGCCCCGGC CGTGCAGCGG GTCGTGTGGA ACTCATCAAG

R00025932 CCGAAGGAGA CTGTCCCAGC CGTGCAGCGG GTCGTGTGGA ACGCATCAAG

TAAGTTTCAA ACGTCCTCCC AAAAGTGGCA CATGCAGAAG ATACAGCGC

TAAGTTTCAA ACGTCCTCCC AAAAGTGGCA CATGCAGAAG ATGCAGCGT

TAAGTTTCAA ACGTCCTCCC AAAAGTGGCA CATGCAGAAG ATACAGCGC

>Ortholog Group 62, Repeat 2

3 99

M00104892 CCTCAGTCTT CCCAGGGGAC GAGATATCAG ACCAGACAGG CTGTGAAAGC

H00420095 CCTCAGTCTT CCCAGGGGAC GAGATATCAG ACCAGACAGG CTGTGAAAGC

R00025932 CCTCAGTCTT CCCAGGGGAC AAGATATCAG ACCAGACAGG CCGTGAAAGC

TGTCCAGCAG AAGGAGGTCA CCCAGAGCCC ATCCACGTCC ACCATCACG

TGTCCAGCAG AAGGAGATCA CACAGAGCCC ATCCACGTCC ACCATCACC

TGTCCAGCAG AAGGAGGTCA CCCAGAGCCC GTCCACATCC ACCATCACG

>Ortholog Group 63, Repeat 1

3 198

M00089474 ---------- ---------- ---------- ---------- ----------

R00023145 ---------- ---------- ---------- ---------- ----------

H00427550 TTCAGACCTT CCACGTCACC ACTGAGTCAT TCTTCTCCTA GTGAAATTTC

---------- ---------- ---------- ---------- ----------

---------- ---------- ---------- ---------- ----------

TGGAACGAGT TCATCAGGGT GTGCGTTAGA GTCCTTTGGT TCAGCAGCTC

---------- ---------- ---------- ---------- ----------

---------- ---------- ---------- ---------- ----------

CTCCCTGTGA GCAGGAGTTG TCTCCCTTGG TGTGCTCGCC TGCTGGGGTG

---------- ---------- ---------- ---------- --------

---------- ---------- ---------- ---------- --------

AGCAGGCTGA CGTATGTGTC TGAACCAGAG AGCTCCTATC CTACCACA

>Ortholog Group 64, Repeat 1

3 198

M00029315 ---------- ---------- ---------- ---------- ----------

H00417510 TCTCAAGCAG GTGTTCCATT TGGTTTAAAA AATACTTCAA GTCTCAGGCC

R00054835 ---------- ---------- ---------- ---------- ----------

---------- ---------- ---------- ---------- ----------

CTTAAATCTA CTCCAGCTTC CAGGTGGTTC ACTTATTTTT AACACTCTGC

---------- ---------- ---------- ---------- ----------

---------- ---------- ---------- ---------- ----------

TCTCCCAGTT TACACCACAA CAACCTCAGC AGCCCACAAC TTGTAGTCCT

---------- ---------- ---------- ---------- ----------

---------- ---------- ---------- ---------- --------

CAACAGCCAG GGGAGCAGGG TTCTGAGCAA GGTTCAACCA GTCAAGAA

---------- ---------- ---------- ---------- --------

>Ortholog Group 65, Repeat 1

3 213

R00055007 GTCTTGAGTG GCATGGGCAA CGGTACCATT TCTTCT---- ----------

M00044517 ATCTTGAGTG GCATGGGCAA TGGTACCGTT TCTTCCTCTC CTGTTGCTAA

H00317053 ATCTTGAGTG GCATGGGCAA TGGTACTATT TCTTCCTCTG CTGCTGTTAG

-CCTGTCCTT AACGCAGCTG CAGGTATCAC GGTGGGAGTG GTTTCAAGTC

CAGTGTCCTT AATGCAGCTG CAGGTATCAC TGTGGGAGTG GTTTCCAGTC

CAGTGTTCCT AATGCAGCTG CAGGGATGAC TGGGGGATCG GTTTCAAGT-

CGCAGCAGCC GCCGCCAACA GTTAACACAT CAAGGTTCAG AGTTGTGAAG

AGCAGCAGCA GCAACCAACA GTTAACACAT CGAGGTTCAG GGTTGTGAAG

---------- ----CCAACA GTTAACACTT CGAGGTTCAG AGTTGTGAAG

TTAGACTCTA CTTCTGAACC CTTTAAAAAA GGCAGATGGA CTTGCACTGA

TTAGACTCTA CTTCTGAACC CTTTAAAAAA GGTCGATGGA CTTGCACAGA

TTAGATTCTA GTTCTGAGCC CTTTAAAAAA GGTAGATGGA CTTGCACTGA

ATTCTATGAG AGA

ATTCTATGAG AAG

GTTCTATGAA AAA

>Ortholog Group 65, Repeat 2

3 201

R00055007 AGCACTAGTG GGAGTTCCGT GAGCAGTAGT GTCAGCACAC TGAGTCACTA

M00044517 AGCACTAGTG GGAGTTCTGT GAGCAGTAGT GTGAGCACAC TGAGTCACTA

H00317053 AGCACTAGTG GGAGTTCAGT GAGCAGTAGT GTCAGCACAC TGAGTCACTA

CACAGAGAGT GTGGGAAGCG GAGAG---AT GGGAGCCCCT ACTGTGGTGG

CACGGAGAGT GTGGGAAGTG GAGAGATGAT GGGAGCCCCG GCTGTGGTGG

TACAGAGAGT GTGGGAAGTG GAGAG---AT GGGAGCCCCT ACTGTGGTGG

TGCCAGGTCT TCAAGGCGTG GCCCTCCAAC AGCTAGATTT CAGTAGCCCT

CGCCAGGTCT TCAAGGTGTG GCTCTCCAAC AGCTAGAGTT CAGTAGCCCT

TGCCAGCTCT CCAAGGTGTG ACCCTCCAAC AGATGGATTT TGGTAGCACT

GGT------- ---------- ---------- CCACAGAGTA TTTCTCAGTC

GCTCCACAGA GTATTGCGGC GGTTAGTATG CCACAGAGTA TTTCTCAGTC

GGTCCACAGA GTATTCCAGC AGTTAGTATA CCACAGAGTA TTTCTCAGTC

A

A

A

>Ortholog Group 66, Repeat 1

3 198

H00396538 CAGCAGGCTA CACAGTTTCA GACAAGAGAA ACTCAGTCTA GAGAGATATT

R00017005 CAGCAGGCGG CACAGTTTCA GACAAGAGAC GCTCAGTCCA GAGACACAAT

M00119370 CAGCAGGCAA CACAGTTTCA GACAAGAGAA GCTCAGTCCA GAGACACAAT

ACAGTCAGAT GGTACAGTGG TTAATTTGTC ACAACTGACT GAGGCATCAT

GCAGTCAGAC ---TCAGTGG TTAACTTGTC ACAGTTGACT GAGGCACCAT

ACAGTCAGAT ---ACAGTGG TTAACTTGTC ACAGTTAACT GAAGCATCAT

CACCACTACA AGAACAAGCA CAGACTTTAC AGCAGCAGAT TTCATCAAAT

CCCCACTGCA AGAACAAGCA ---------- -----CAGAT ACCATCAAAT

CCCCACTACA AGAACAAGCA CAAACGTTAC AGCAGCAGAT ACCATCAAAT

ATTTTTCCAT CACCAAATAG TGTGAGTCAG CTTCAGAATA CTATTCAG

ATTTTTCCGT CGCCAAATAG TGTGAGCCAG CTCCAGAGTA CTATTCAG

ATTTTTCCAT CGCCAAGCAG TGTCAGCCAG CTCCAGAGTA CTATTCAG

>Ortholog Group 66, Repeat 2

3 213

H00396538 TTTTCCTCAA CAGAGCCAAC AGTCCATACC AGACCAGATA ATTTATTACC

R00017005 TTTTCCTCAA CAGAGTCTGC AGTTCACACC AGACCAGATA ACTTACTACC

M00119370 TTTTCTTCAG CGGAGTCTGC AGTTCACACT AGACCAGATA ACTTACTACC

TGGAAGAGCT GAAAGTGTTC ATCCACAGTC TGAAAACACG TTATCTAAT-

TGGGAGGGCC GACAGCGTCC ATCAGCAGAC TGAAAACACA CTGTCTAGTC

TGGGAGGGCT GACAGCGTCC ATCAACAGAC TGAAAATACA CTGTCTAAT-

---------- ----GTGATG GAATCTTCAG CCGCAATGGT GATGGAGATG

AGCAGCAGCA ACAGGTGATA GAGTCTTCAG CTGCCATGGT GATGGAGATG

---------- ----GTGATG GAGTCATCAG CTGCAATGGT GATGGAGATG

CAACAGAGTA TCTGCCAGGC AGCTGCCCAG ATTCAGTCAG AGTTATTCCC

CAGCAGAGCA TTTGCCAAGC AGCAGCCCAG ATTCAGTCAG AACTGTTTCC

CAGCAGAGCA TTTGCCAAGC AGCTGCCCAG ATCCAGTCAG AGCTGTTTCC

TTCAACTGCT TCA

TTCAGCTGCT TCA

TTCAGCTGCT TCA

>Ortholog Group 66, Repeat 3

3 207

H00396538 TCCCTTCCAC CTAATCCAAT GCCTCAAAGC CAACAAGGAA CCATGTTCCA

R00017005 TCCCTTCCTG CTAATCCAAT GCCTCAGAAC CAGCAAGGCC CAATTTTCCA

M00119370 TCCCTGCCTC CTAATCCAAT GCCTCAAAAC CAGCAAGGTC CAATTTTCCA

GTCACAGCAC TCAATAGTTG CCATGCAGAG TAACTCTCCA TCCCAGGAA-

GACACAGCGC CCAATAGTTG GCATGCAGAG TAACTCTCCA TCCCAGGAG-

AACACAGCGC CCAATAGTTG GCATGCAGAG TAACTCTCCA TCCCAGGAGC

--------AG CATTTTATTC AGTAATCAGA ATACCATGGC TACAATGGCG

-----CAGAG CATTTTATTT AGTAATCAGA ATGCCATGGC TACAATGGCC

AGCAGCAGAG CATTTTATTC AGTAATCAGA ATGCCATGGC TACAATGGCC

TCTCCAAAGC AACCACCACC AAACATGATA TTCAACCCAA ATCAAAATCC

TCCCAGAAGC AGCCACCACC AAACATGATA TTTAGCCCAA ACCAGAACCC

TCCCAGAAGC AGCCACCACC AAACATGATG TTTAGCCCAA ACCAGAACCC

AATGGCT

GATGGCT

AATGGCT

>Ortholog Group 67, Repeat 1

3 201

M00034090 AAGGTAGCGG TGGCCCAGTT CTCCCAGGAG GCGAGGTGTG GGGGGGCCTC

R00018628 AAGGTGGCGG TGGCCCAGTT CTCCCAGGAA GCGAGGTGTG GGGGGGCCTC

H00251020 AAGGTGGCGG TGGCCCAGTT CTCCCAGGAA GCGAGGTGCG GCGGGGCCTC

CGGAGGCAAG CTGCTCATCT CAACCCTCAT GGAACAACTC CTGGCTCTGA

TGGAGGCAAG CTGCTCATCT CGGCCCTCAT GGAGCAGCTC CTAGCTCTGA

TGGGGGCAAG CTGGCCGTCC CAGCCCTCAT GGAACAACTC CTAGCTCTGA

TTCACCAGCT GCAGCTCATA GAGCAAATAC GTCACCAAAT ACTGCTGCTG

TCCACCAGCT GCAGCTCATA GAACAGATTC GTCACCAAAT ACTGCTGCTG

TCCACCAGCT GCAATTGATC GAACAGATTC GTCACCAAAT ATTGCTGTTG

GCCTCTCAGA GCGCAGACCT GCCGGCAGCA CCCTCTATCC CGTCTCAAGG

GCTTCTCAGA ACGCAGATCT G---TCAGCC TCTTCTGTTC CTTCTCAAGG

GCTTCTCAGA ATGCAGACTT GCCAACATCT TCTAGT---C CTTCTCAAGG

T

T

T

>Ortholog Group 68, Repeat 1

3 111

H00385705 GCTCCACTGA TAAGCTCCAT GGGAACGACC ATGGTTGGCT CAGCACCCTC

M00105250 GCTCCACTGA TAAGTTCCAT GGGGACGACC ATGGTTGGTT CAGCAACCTC

R00034957 GCTCCACTTA TCAGCTCCAT GGGAACGACC ATGGTTGGCT CAGTATCCTC

CACCCAAGTG AGTCCTTCGG TGCAAACCCA GCAGCATCAG ATGCAATTG-

CACCCAGGTG AGCCCTTCGG TGCAAACCCA GCAGCATCAG ATGCAGTTG-

CACGCAGGTG AGCCCTTCGG TACAAACCCA GCAACATCAG CTGCAGCTGC

-----ATGCA A

-----ATGCA G

AGCAGATGCA G

>Ortholog Group 68, Repeat 2

3 45

H00385705 CAGATGCTCC AGCAGCACCA AATGCATCAG CAAATCCAGC AGCAG

M00105250 CAGATGTTAC AGCAGCACCA AATGCATCAG CAGATCCAGC AGCAG

R00034957 CAGATGCTGC AGCAGCACCA GATGCATCAG CAGATTCAGC AGCAG

>Ortholog Group 68, Repeat 3

3 144

H00385705 CAGCAGCAGC ATTTCCAGCA CCACATGCAG CAGCACCTG- --CATCTCCA

M00105250 CAGCAGCAGC ATTTTCAGCA TCACATGCAG CAGCACCTGC AGCATCTCCA

R00034957 CAGCAACAGC ATTTCCAGCA CCACATGCAA CAGCACCTGC AGCACCTCCA

GCAGCAAATT AATCAACAGC AGCTGCAGCA GCAGCTGCAG CAGCGCCTCC

GCAGCAGCTC AGCCAACAGC AGCTGCAGCA GCAGTTGCAG CAGCATCTCC

GCAGCAGATC AGCCAACAGC AGCTGCAGCA GCAGCTGCAG CAGCATCTCC

AGCTG---CA GCAGCTGCAA CACATGCAGC ACCAGTCTCA GCCT

AGCTG---CA GCAACTGCAG CACATGCAGC ACCAGTCTCA GCCT

AGCTGCAGCA GCAGCTGCAG CACATGCAGC ACCAGTCTCA GCCT

>Ortholog Group 69, Repeat 1

3 198

R00014988 TCTCATAAAC TTCGTAAAAA TAATCACCAT CTTTTTACAA CCACAATTAA

H00281537 TCTCATAAAC TTCGTAAAAA TAATCACCAT CTTTTTACAA CTACAATTAA

M00099652 TCTCATAAGC TTCGTAAGAA CAATCACCAT CTCTTCACAA CTACAATTAA

CTTAAACTCA ATGAATGATG GTTGGTATGG TGCCCTGAAA GAAGCGATTA

CTTAAATTCA ATGAATGATG GTTGGTATGG TGCGCTGAAA GAAGCAATTA

CTTAAACTCA ATGAATGATG GTTGGTACGG TGCCCTGAAA GAAGCGATTA

ACCAGCTGGT GTGGGTCTCT GAGGGGAAGG CGGATGGTGC TACAAGTGAT

ACCAGCTGGT ATGGGTTTCC GAGGGAAAGG CGGATGGTGC TACAAGTGAT

ACCAGCTGGT GTGGGTCTCT GAGGGGAAGG CGGATGGTGC TACAAGTGAT

GACCTTGATT TGCATGATGA TCGTCTGTCC TACCTGTCAG CCCCAGGT

GACCTTGATT TGCATGATGA TCGTCTGTCC TACCTGTCAG CTCCAGGT

GACCTTGATT TGCATGACGA TCGTCTGTCC TACCTGTCAG CCCCAGGT

>Ortholog Group 6, Repeat 1

3 198

M00079913 CAAATAAAAC AAGAAACTAT TGAGTCCAGG CTTTCAGAAT TAAAAAGTGA

H00357440 CAGATAAAAC AGGAAACTAT TGAGTCCAGG CTTTCTGAAT TAAAAAGTGA

R00001057 CAAATAAAAC AAGAAACTAT TGAGTCCAGG CTTTCAGAAT TAAAAAGTGA

GAATGAATCC CTTTGGAAGG AGGTGTCAGA ACTAAGAGCA AAGCATGCCG

GAATGAGTCC CTTTGGAAGG AGGTGTCAGA ATTACGAGCA AAGCATGCAG

GAATGAATCC CTTTGGAAGG AGGTGTCAGA GTTAAGAGCG AAGCATGCAG

TTATTCGGAA GATTGTCCAG TTTATTGTTA CATTGGTTCA GAATAATCAA

TTATTCGAAA GATTGTCCAG TTTATTGTTA CATTGGTTCA AAATAACCAA

TTATTCGAAA GATTGTCCAG TTTATTGTTA CATTGGTTCA GAATAATCAA

CTTGTGAGTT TAAAACGTAA AAGGCCTCTA CTTCTAAACA CAAATGGA

CTTGTGAGTT TAAAACGTAA AAGGCCTCTA CTTCTAAACA CTAATGGA

CTTGTGAGTT TAAAACGTAA AAGGCCTCTA CTTCTAAACA CAAATGGA

>Ortholog Group 70, Repeat 1

3 198

R00012377 AATGCAGCAT CAAATACACT GGTTAGGGGA AAGGCCCCGA ACTATCAGCT

M00029879 AACGTAGCAT CAGATACGCT GGTTAGAGGA AAGACCCCGA GCTATCAGCT

H00265433 AGTATGGTAT CAAATACTTT GGCTAAGATG AGAATCCCAA ACTATCAGCT

TTCACCAATG AAATGTCCTG CTGCAAGTAA AAATAAAGAT TGGTCTTCT-

TTCTCCAATG AAATTTCCTG TTGCAAATAA AAATAAGGAT TGGACTTCT-

TTCACCAACT AAATTGCCAA GTATAAATAA AAGTAAAGAT AGGGCTTCTA

--AACTCCAT CAAAAACTAC TTCCAGCCAT GCTCCAGAAA AAGGGAAAGG

--AACTCCAT CAAAAACTAC TTCCAGCCAT GCACCAGAAA AAGGGAAAGG

CCAACTCCAT CAGAAACTAC TTTCAGCCGT CTACCAAAAA AAGGGAAAGG

GATGAAGAAA ATCCAGAACA GTCCTCATGC AAATCGTCAA GAGTGGAA

GATGAAGACA ACCCAGAGCT GTCCTCGTGC AAATCATCCA GGATGGAG

GATGAAGAAA ATCAAGAAAT GTCTTCATGC AAATCAGCAA GAATAGAA

>Ortholog Group 71, Repeat 1

3 198

M00033096 CTCAGCCAGG AGACAGAGGC CCTGGGGCGC TCCCAGGACC GCCTGGAGGC

H00385211 CTCAGCCAGG AGACAGAGGC TCTAGGGCGG TCCCAGGGCC GCCTGGAGGC

R00028390 CTCAGCCAGG AGACAGAGGC CCTGGGGCGC TCCCAGGACC GCCTAGAGGC

ACAGAAGAGA GAGCTGCAGC AGGCTGTTCT GCAGATGGAG CAGAGGAAG-

CCAGAAGAGA GAGCTGCAGC AGGCTGTGCT GCACATGGAG CAGCGGAAGG

TCAGAAGAGA GAGCTGCAGC AGGCCGTTCT GCAGATGGAG CAAAGGAAG-

-----AGCGC TCCGCCTTCC AAACCTGACG GGCAGCTGCA GTTCCGTGCA

GCCACAAGGC CCCGGCTGCC CACCCTGAGG GGCAGCTCAA GTTCCACCCA

-----AGCGC TCCGCCTTCC CAACCTGATG GGCAGCTGCA GTTCCGTGCA

GACACAGATG ACGCTCCTGT CCCAGCTCCA GCAGGTGACC AGAAAGAT

GACACAGACG ATGTACCTGT CCCAGCTCCA GCCGGTGACC AGAAGGAG

GACACAGGGG ATGCTCCTGT CCCAGCTCCA GCAGGTGACC AGAAAGAC

>Ortholog Group 72, Repeat 1

3 198

M00046059 CAGCTGGAGG AGGAGCGGGA CCTGAAAGTC ACCGACATCA TAGTGTCTTT

H00406273 CAGCTGGAAG AGGAGCGAGA CCTGAAGGTC ACCGACATCA TCGTCTCCTT

R00036792 CAACTGGAGG AGGAGCGAGA TCTCAAGGTC ACCGACATCA TAGTATCTTT

CCAGGCAGCG GCACGGGGCT ACCTGGCCCG TAGGGCTTTC CAGAGACGGA

CCAGGCAGCT GCCCGGGGAT ACCTGGCTCG CAGGGCCTTC CAGAAGCGCA

TCAGGCAGCG GCACGGGGCT ACCTGGCCCG TAGGGCTTTC CAGAGGAGGA

GTGCTCTGAG GGTGATGCAG AGAAACTGTG CTGCCTACCT CAAGCTCAGG

GCGCCCTGAG GGTGATGCAG CGGAACTGCG CGGCCTACCT CAAGCTGAGA

GTGCTCTGAG GGTGATGCAG AGAAACTGTG CCGCCTACCT CAAGCTGAGG

AACTGGCAGT GGTGGAGGCT GTTCATCAAG GTGAAGCCCC TGCTGCAG

CACTGGCAGT GGTGGCGGCT GTTTACCAAG GTGAAGCCAC TGCTGCAG

AACTGGCAGT GGTGGAGACT GTTCATCAAG GTGAAGCCCC TGCTGCAG

>Ortholog Group 73, Repeat 1

3 198

H00221996 GAGGAGGTGG CTCTGAAGAT CAATCTGCCT GAGTCCAGGG TTCAGGTTTG

M00043436 GAGGAGGTTG CTCTTAAGAT CAATCTGCCT GAGTCCAGGG TCCAGGTCTG

R00018858 GAGGAGGTTG CTCTCAAGAT CAACCTGCCT GAGTCCAGGG TCCAGGTCTG

GTTCAAGAAC CGGAGGGCTA AATGCAGGCA GCAGCGACAG CAGCAGAAAC

GTTCAAGAAT CGTAGGGCGA AATGCAGACA GCAGCGACAG CAGCAGAAAC

GTTCAAGAAT CGCAGGGCGA AATGCAGACA GCAGAGGCAG CAGCAGAAAC

CCCCAGGGGG CCAGGCCAAG GCCCGGCCTG CCAAGAGGAA GGCGGGCACG

CCCCGGGGGC ACAGACCAAG GCTCGTCCTG CGAAGAGGAA GGCAGGGACA

CCCCAGGGGT ACAAGCCAAG GCTCGTCCTG CGAAGAGGAA GGCAGGCACA

TCCCCAAGAC CCTCCACAGA TGTGTGTCCA GACCCTCTGG GCATCTCA

TCCCCGAGAC CCTCTACAGA TGTTTGTACA GATCCTTTGG GCATCTCA

TCCCCGAGAC CCTCTACAGA TGTTTGTACA GATCCTTTGG GCATCTCA

>Ortholog Group 74, Repeat 1

3 198

M00006470 GCCCGTGTTG CAAGTCTGGG TTCCCTGCCA CTATCTGGGG TGGAGGAGAA

R00041928 ACCCGTGTTG CAAGTCTGGG TCCCCTGCCA CTATCTGGGG TGGAGGAAAA

H00222270 GCCCGGATTG CGGGCGTGGG TTCCTTGCCG CTGTCTGGGG TAGAGGAGAA

AATGTTTAGC CTTCTTAAGA GAGCTAAGGT GCAGTTATTC AAGATCGATA

AATGTTTAGC CTTCTTAAGA GAGCTAAGGT GCAGTTATTC AAGATTGATA

GATGTTCAGC CTCCTCAAGA GAGCCAAAGT GCAGCTATTC AAGATCGATA

AAGTGGCGGC TTCAATGCCG CTAAGCCCTG CAGTACAAAC CGAGGAGGCT

AAGTGGCAGC TTCAATGCCG CCAAGCCCTG CAGTACAAAC CGAGGAGGCT

AGGTGGCAGC TTCCATGCCG CTGAGCCCTG GAGGGCAGAT GGAGGAGGTG

GTGGGGACTG TCAAACAGAC CCCGGACAGA GGCTGTGTCA GGTCTGAA

GTGGGGACTG TCAAACAGAC CCCAGACAGA GGCTGTGTCA GGTCTGAA

GCCGGGGCTG TCAAGCAGAT CTCCGACAGA GGCCCTGTCC GGTCTGAA

>Ortholog Group 75, Repeat 1

3 204

H00378812 TTGGGTCACG CCATTACTGG GGGCTTCAGT GGAGGAAGTA ATGCTGAGCC

M00091835 CTGGGTCACG CCATCACTGG GGGCTTCAGC GGAGGTGGCA GTGCTGAGCC

R00045765 CTGGATCACG CCATCACTGG GGGCTGCAGC AGGGGTGCTA ATGCTGAGGC

TGCGAGGCCT GACATCACTT ACCAGGAGCC TCAGGGAACC CAGCCAGCA-

CGCAAAGCCC GACATCACTT ACCAGGAGCC TCAGGGAGCC CAGCTGCAGT

TGTGAAGCCT GACATCACTA ACCATGAGCC TCAGGGAGTC CAGCTGCAGC

-----CCTTG CCTCTATGAG ATCAAACAGT TTCTGGAGTG TGCCCAGAAC

TTGGACCTTG CTCTCTAGAG ATCAAGCAGT TTCTGGAGTG TGCTCAGAAC

GTGGACCTTA CTCTCTCCAG ATCAAGCAGT ATCTGGAGTG TGCTCAGAAC

CAGGGTGACA TCAAGCTCTG TGAGGGTTTC AATGAGGTGC TGAAACAGTG

CAGAGCGATG TCAAGCTCTG TGAGGGCTTC AACGAGGTGC TGCGGCAGTG

AAGAGTGATG TCATGGTCCG TGAGGGCTTC AGTGAGGTGC TGCGGCAATG

CCGA

CAGG

CTGG

>Ortholog Group 76, Repeat 1

3 153

H00223145 CTGACCGTTG AGCAGCTCTC ATCCCGGGTT TCCTTTACGT CTCTTTCTGA

M00058581 CTGACCGTAG AGCAGCTTTC CTCCCGGGTT TCCTTCACAT CTCTCTCTGA

R00011395 CTAACCGTAG AGCAGCTCTC CTCCCGGGTC TCCTTCACGT CCCTTTCTGA

TGACACCAGC ACAGCGGGCT CCATGGAGGC CTCTGTCCAG CAGCCATCCC

TGACACCAGC ACAGCCGACT CCCTGGAGCC CTCTGCCCAG CAGCCATCTC

TGACACCAGC ACCGCAGACT CCCTGGAGCC CTCTGTCCAG CAGCCATCTC

TCCTGCAGGA ACTGCAGGGT GAGGACCACA TCTCTGCTCA GAACTATGTG

TCCTACAGGA CTTGCAGGTG GAGGAACACG TCTCCACTCA GAACTATGTG

TCCTGCAGGA TTTGCAGGCA GAGGAACACA TCTCCACTCA GAACTATGTG

ATC

ATG

ATG

>Ortholog Group 77, Repeat 1

3 231

M00114173 GCAGCAGCTG CAGCTGGAGG CATTATGACT CTGCCATTGC AAAATCTACA

R00017713 GCGGCAGCTG CAGCTGGAGG CATTATGACT CTGCCATTGC AAAATTTACA

H00384004 GCAGCAGCTG CAGCCGGAGG CATTATGACT CTGCCACTGC AAAATCTACA

AGCTACCTCA TCCCTGAACT CCCAGCTGCA ACAGCTCCAG CAACTCCAGC

AGCTACCTCA TCCCTGAACT CCCAGCTGCA ACAGCTCCAG CAACTCCAGC

AGCTACCTCA TCCCTGAACT CCCAGCTCCA GCAGCTC--- CAGCTCCAGC

TGCAGCAACA ACAACAGCAG CAACAACAAC CACCTCCTCC ACCAACCAGC

TC-------- ---------- ---------C CACCTCCTCC ACCAACCAGC

TC-------- ---------- ---------- --CCTCCCCC GTCAACCAAC

CAGCACCCAC AGCCAGCCTC ACAGGCACCC CCACAGTCC- ----------

CAGCACCCAC AGCCAGCCTC ACAGGCGCCC GCACAGTCTC AGCAGCCGCA

CAGCACCCGC AACCAGCCCC ACAGGCGCCC TCGCAGTCCC AGCAGCAGCC

----CAGCCC ACACCGCCTC ACCAGCCACC A

GTCCCAGCCC ACCCCGCCTC ACCAGCCACC A

GCTGCAGCCC ACCCCACCCC AGCAGCCACC A

>Ortholog Group 78, Repeat 1

3 123

M00112137 GCTGGTGAGG TTGACTTCAA AGACATCGAC ATAACGGACG AAATCCTGAC

R00006618 TCCGGTGAGG TTGACTTCAA AGACATCGAC ATAACAGACG AAATCCTGAC

H00242057 TCTGGTGAGG TTGACTTCAG AGACATTGAC TTAACGGATG AAATCCTGAC

CTACGTGCAG GATTCCCTGA ACAATTCAAC TTTGCTGAAC TCG---GCTC

GTACGTGCAG GATTCTCTGA ACAATTCAAC TCTGCTGAAT TCA---GCTC

GTATGTCCAA GATTCTTTAA GTAAGTCTCC CTTCATACCT TCAGATTATT

CTGTGACTCA GCACCTAAGC TGT

CTGTGAGCCA GCACCTAAGC TGC

CCTTGGCTCT GAACTCAAGC TGT

>Ortholog Group 78, Repeat 2

3 159

M00112137 CTGCAGGAGC GCCTGCAACT AGAGCTTCAG CAGCCC---C CGCCGCAGGC

R00006618 CTGCAGGAGC GCCTGCAGCT GGAGCTTCAG CAGCAGCACC CCACTCAGAC

H00242057 GTACAGGAAC ACCTACATCT AGAACATCAC CAAAAG---- --CAAGTAGT

TCTGGAGCCC CAGCAGCAGC TGTGTCAGAT GGTGTGCCCC CAGCAAGATC

ACTGGAGCCC CAGCGCCAGT TGTGTCAGGT GGAGGTCCCC CAGCACGAGC

AGTGGAGCCA CAGCAACAGC TGTGTCAG-- ---------- ----------

TGGGT----- -CCGAAGCAC ACGCAAATCA ACGGCACGTT TGCAAGTTGG

TGGGTCAGAA AACGAAGCAC ATGCAAGTCA ATGGCATGTT CGCCAGTTGG

--------AA GATGAAGCAC ATGCAAGTTA ATGGCATGTT TGAAAATTGG

AACCCCACC

AACCCTGCC

AACTCTAAC

>Ortholog Group 79, Repeat 1

3 48

H00265997 ATGCAGGATG ATTTACTGAT GGACAAAAGC AAAACCCAGC CCCAGCCC

M00078690 ATGCAGGATG ATTTACTGAT GGACAAAAGC AAAACCCAGC CCCAGTCT

R00040393 ATGCAGGATG ATTTACTGAT GGACAAAAGC AAAACCCAGC CCCAGTCT

>Ortholog Group 79, Repeat 2

3 111

H00265997 CCC------- -----CAACC TGAGTCCAGC GTATCCGAAG CCCCGTCCAC

M00078690 CAGCAGCAAC AGCTCCAGCC CGAGCCCGGC GCAGCTGAAG CCCCGTCCAC

R00040393 CAGCAGCAAC AGCTCCAGCC CGAGCCCGGC GCAGCCGAAG CCCCGTCCAC

GCCCCTCTCC TCAGAGACCC CCAAGCCGGA GGAAAACAGC GCAGTGCCGG

GCCCCTCTCC TCAGAGATCC CCAAGCCCGA AGACAGTAGC GCAGTGCCGG

GCCCCTCTCC TCAGAGACCC CCAAGCCTGA AGACAGTAGC GCAGTGCCGG

CCCTCAGCCC A

CCCTCAGCCC C

CCCTCAGCCC C

>Ortholog Group 7, Repeat 1

3 201

M00069080 CCCTCCTCCT CCATCAGCAG CAAAAGCAAC AGCGAAGACC GGTTCCCACA

R00003092 CCCTCCTCCT CCATCAGCAG CAAAAGCAAC AGCGAAGACC GGTTCCCGCA

H00420194 CCCTCCTCCT CCATCAGCAG CAAGAGCAAC AGCGAAGACC CATTCCCACA

GCCGGAGAGG CAGAAGCAAC AGCAACCACT GGCCCTGACC CAGCAAGAAC

GCCAGAGAGG CAGAAGCAAC AGCAACCGCT GTCCCTGACC CAGCAAGAAC

GCCCGAGAGG CAGAAGCAGC AGCAGCCGCT GGCCCTAACC CAGCAAGAGC

CCCTGACTCT CCAGCCGCAG CAACAGCAAC AGCCACAGCA GCCGAGATGC

CCCTGACCCT CCACCCACAG CAACAGCAGC AGCCACAGCA GCCGAGATGC

CCCTGACCCT C---CCACAG CAGCAACGAT CTCAGCAGCA GCCCAGATGC

AAACAGAAGG TCATCTTCGG CAGTGGCACA GTCACCTTCT CTCTGAGCTT

AAACAGAAGG TCATCTTCGG CAGTGGTACG GTCACCTTCT CTCTGAGTTT

AAGCAGAAGG TCATCTTTGG CAGCGGCACG GTCACCTTCT CACTGAGCTT

T

T

T

>Ortholog Group 80, Repeat 1

3 198

M00124863 ATGGCCAATG CCAACAACCC TATGAATCCA GGTGGCAACC CCATGGCATC

H00334474 ATGGCCAATG CCAACAACCC CATGAATCCA GGCGGCAACC CCATGGCGTC

R00014003 ATGGCCAATG CCAGCAACCC TATGAATCCA GGTGGCAACC CCATGGCATC

AGGCATGAGC ACCAGCAACC CCGGCATCAA CTCCCCACAG TTCGCAGGGT

GGGCATGACC ACCAGCAACC CAGGCCTCAA CTCCCCACAG TTTGCGGGGT

GGGCATGAGC ACCAGCAACC CCGGCCTCAA CTCCCCACAA TTTGCAGGGT

TCTCCACCAA AGCTGGCCCT GCACAGCCCT ATATCCAGCC CAACATGTAC

TCTCAGCCAA GGCTGGCCCC GCTCAGCCCT ACATCCAGCA GAGCATGTAT

TCTCCACCAA AGCTGGCCCT GCACAGCCCT ATATCCAGCC CAACATGTAC

GGCCGACCTG GCTACCCTGG TAGCGGGGGC TTTGGGGCCA GTTACCCT

GGCCGGCCCA ACTACCCCGG CAGCGGGGGC TTTGGGGCCA GTTACCCT

GGCCGGCCCG GCTACCCTGG TAGCGGGGGC TTCGGAGCCA GCTACCCT

>Ortholog Group 81, Repeat 1

3 171

R00031472 ATGGAGGAGA AGCCCGGCCA GCCACAGCCT CAGCACCATC ACAGCCACCA

M00049412 ATGGAGGAGA AGCCCGGCCA GCCACAGCCT CAGCACCATC ACAGCCACCA

H00225388 ATGGAGGAGA AGCCCGGCCA GCCACAGCCT CAGCACCATC ACAGCCACCA

CCATCCGCAC CATCACCCC- --TCGCACCA CCACCACCAT TATTATTTCT

CCATCCGCAC CATCATCCCC AGTCGCACCA CCACCACCAT TATTATTTCT

CCATCCGCAC CATCACCCT- --CCGCACCA CCACCACCAT TATTATTTCT

ACAACCACAG CCACAACCAC CACCACCACC ACCATCACCA GCAGGCTCAC

ACAACCACAG CCACAACCAC CACCACCACC ACCATCACCA GCAGCCTCAC

ACAACCACAG CCACAACCAC CACCACCACC ATCATCACCA GCAGCCTCAC

---TACCTGC AGCATGGAGC C

CAATACCTGC AGCATGGAGC C

CAATACCTGC AGCATGGAGC C

>Ortholog Group 82, Repeat 1

3 198

M00044268 TTTGTTGAAG CCACACAAAG AACAAGCTGC AGTTGTTTGA GGCATAAAAG

H00380888 TTTGTTGAAG CCACACAAAG AACAAATTGC AGTTGTTTGA GGCACAAAAA

R00032864 TTTGTTGAAG CCACACAAAG AACAAGCTGT AGTTGTTTGA GGCATAAAAG

TCTCAAGACA AGAAATACTG GGCAACAAGG ACAGGCACCA TCTTTAGGGG

TCTCAAGTCA AGAAATGCTG GACAACAAGG ACAGGCACCA TCTTTAGGTA

TCTTAAAACA AGAAATACTG GGCAACAAGG ACAGGCACCA TCTTTAGGGA

TACTTCCTAA GCACAAGACC AATGAGAAGC AAGACAAGAG TGAGAAGCCA

TACTTCCTAA GCACAAGACC AATGAGAAGC AAGAAAAGAG TGAAAAGCCA

TACTTCCTAA GCACAAGACC AATGAGAAGC AAGAAAAGAG TGAGAAGCCA

CAGAAGCGCC CCTTGACTCC CTTTCACCAT CGTGTATCAG TTAGTGAT

CAGAAACGCC CCTTGACTCC TTTTCACCAT CGTGTGTCTG TTAGTGAT

CAAAAGCGCC CCTTGACCCC TTTTCACCAC CGTGTATCAA TTAGTGAT

>Ortholog Group 83, Repeat 1

3 198

H00379122 CAGCCCCATG ACAGGCCGCT GACTGCCAGC TCCAGCCTGG CCCCGGGGCA

R00030733 CAGCCTCATG ACAGGCCGAT GAGTGCCAAT GCGAGCCTGG CTCCAGGGCA

M00099749 CAGCCTCATG ATAGGCCGAT GAGTGCCAAT GCGAACCTGG CTCCAGGGCA

GCGGGTCCAG AATCTTCATG CCTACCAGTC GGGCCGCCTC AGCTATGACG

ACGGGTCCAG AATCTTCACG CTTACCAGCC GGGCCGCCTT GGCTAT---G

ACGGGTCCAG AATCTTCACG CTTACCAGCC TGGCCGCCTT GGCTAC---G

CCCTTCAGAG CCGGCACCAT GCCCAGGAAA CCCTCCATTA CCAAAACCTC

CACTTCAAGG CCGGCACCAC ACCCAGGAAA CACTCCACTA CCAGAACCTC

CACTTCAAGG CCGTCACCAC ACCCAGGAAA CACTCCACTA CCAGAACCTC

GCCAAGTATC AGCACTACGG GCAGCAAGGC CAGGGCTACT GCCAGCCG

GCCAAGTACC AACACTATGG ACAGCAAGGC CAGGGCTACT GTCCAGCG

GCCAAGTACC AACACTATGG ACAGCAAGGC CAGGGCTACT GTCCACCG

>Ortholog Group 84, Repeat 1

3 114

M00021157 GTGAATGCGC AGTGTGCGAA GTTCATCGAC GAGCAGCAGA TTCTGCACTG

R00019919 GTGAATGCTC AGTGTGCGAA GTTCATTGAC GAACAGCAGA TTCTGCACTG

H00225728 GTGAATGCTC AGTGTGCGAA ATTTATTGAT GAACAGCAGA TTCTACATTG

GCAGCACTAC TCTCGGAAGC GGGTGCGTCT TCAGCAAGCC CTGGCCGAGA

GCAGCACTAT TCGAGGAAGC GGATGCGTCT TCAGCAAGCC CTAGCAGAGA

GCAGCACTAT TCCCGGAAGC GGATGCGCCT TCAGCAAGCC TTGGCAGAGA

ATAACACAGC AGGG

ATACCACAGC AGGA

ATAACACATC GGGA

>Ortholog Group 85, Repeat 1

3 198

M00017974 GAAAGGATCT TACTGAAGCA GTTTGGGAGT CCTGGCCACA CTCGGGGTAT

H00251642 GAAAAGATCC TGCAAAGGCA GTTCAGTAGC TCTAACAGCC CTCGGGGTAT

R00024718 GAAGGGATCC TACTGAAGCA GTTTGGGAGT CCTGACCACA CTCGGGGTAT

CATCTTCACC AGAACCCGTC AGACTGCTTC CTCCCTCCTG CTCTGGCTTT

CATCTTCACC CGCACCCGCC AAAGCGCACA CTCCCTCCTG CTCTGGCTCG

CATCTTCACC AGAACCCGTC AGACTGCTTC TTCCCTCCTG CTCTGGCTTT

GCCTACAGAC TGTGGGCATC AAGCCGCAGA TGCTGATCGG AGCAGGGAAC

GCCTGCAGAC TGTGGACATC CGGGCCCAGC TACTGATTGG GGCTGGGAAC

GCCTACAGAC TGTGAACATC AAGCCCCAGA TGCTGATTGG AGCAGGGAAC

ACAAGCCAGA GCACACACAT GACCCAGAAA GACCAGCAGG AAGTGATC

AGCAGCCAGA GCACCCACAT GACCCAGAGG GACCAGCAAG AAGTGATC

ACAGGCCAGA GCACACATAT GACCCAGAAA GACCAGCAAG AGGTGATC

>Ortholog Group 86, Repeat 1

3 198

R00052059 ---------- ---------- ---------- ---------- ----------

M00008999 GGTAGCCCCA GCCCCGTGGA GCTTCGGGGG GCTCTGGCGG GCCCCATGGA

H00225983 GGCAGCCCCA GCCCTGTGGA GCTACGGGGG GCTCTGGTGG GCTCTGTGGA

---------- ---------- ---------- ---------- ----------

CCCTGCGCTA CGGGAGCAGC AACTGCAGCA GGAGCTCCTG GTCCTCAAGC

CCCCACACTG CGGGAGCAGC AACTGCAGCA GGAGCTCCTG GCGCTCAAGC

---------- ---------- ---------- ---------- ----------

TCCAGAAGCA GCTCCTGTTC GCCGAGTTCC AGAAGCAGCA CGACCACTTG

TGCAGAAGCA GCTCCTGTTC GCTGAGTTCC AGAAACAGCA TGACCACCTG

---------- ---------- ---------- ---------- --------

ACGCGGCAGC ACGAGGTCCA GCTGCAGAAG CACCTC---- --------

ACAAGGCAGC ATGAGGTCCA GCTGCAGAAG CACCTCAAGC AGCAGCAG

>Ortholog Group 87, Repeat 1

3 198

M00090177 TATTTCGTTG ACCATAACAA CAGAACAACA CAATTTACAG ATCCCCGGCT

H00262435 TATTTCGTTG ACCATAACAA CAGAACAACA CAATTTACAG ATCCTCGGCT

R00060126 TATTTCGTGG ACCATAACAA CAGAACAACA CAGTTTACAG ATCCTCGTCT

CTCTGCTAAC TTGCATTTAG TTTTAAATCG TCAGAACCAG TTGAAAGACG

GTCTGCTAAC TTGCATTTAG TTTTAAATCG GCAGAACCAA TTGAAAGACG

CTCTGCTAAC TTGCATTTAG TTTTAAATCG TCAGAACCAG TTGAAAGACG

TGGTGCCATT GTGTCCCGAT GACACTGAGT GTCTGACAGT GCCAAGATAC

TGGTATCGTT ATGTCCTGAT GACACAGAAT GCCTGACAGT CCCAAGGTAC

TGGTGTCGTT GTGTCCCGAT GACACTGAGT GTCTGACAGT GCCAAGGTAC

AAGCGAGATT TGGTTCAAAA ACTAAAAATC TTGCGGCAAG AACTTTCC

AAGCGAGACC TGGTTCAGAA ACTAAAAATT TTGCGGCAAG AACTTTCC

AAGCGAGACT TGGTTCAAAA ACTAAAGATC TTGCGGCAAG AACTTTCC

>Ortholog Group 88, Repeat 1

3 198

H00318136 GAGCTTGTTG ATCAACTTGC AAAAGAAGCA GAAAATGAAA AGATGAAGGC

M00118015 GAGCTTGTTG ATCAGCTTGC CAAAGAAGCA GAGAACGAGA AGATGAAGGC

R00011795 GAGCTTGTTG ATCAGCTTGC CAAAGAAGCA GAGAACGAGA AAATGAAGGC

CATCGGTGCT CGGAACTTGC TCAAATCTAT AGCAAAGCAG AGAGAAGCTC

CATTGGTGCT CGGAACTTGC TGAAGTCCAT AGCGAAGCAG AGAGAAGCCC

CATTGGTGCT CGAAACTTGC TGAAATCCAT AGCGAAGCAG AGAGAAGCCC

TTCAAGCCCT AATAGCAGAA AAGAAAATGC AGCTAGAAAG GTATCGGGTT

TGCAGGCCCT GATAGCAGAA AAGAAGACGC AGCTAGAAAG GTATCGGGTT

TGCAGGCACT GATAGCAGAA AAGAAGATGC AGCTAGAAAG GTATCGGGTT

GAATATGAAG CTTTGTGTAA AGTAGAAGCA GAACAAAATG AATTTATT

GAATATGAAG CTTTGTGTAA AGTAGAAGCA GAACAAAATG AATTTATT

GAATATGAAG CTTTGTGTAA AGTAGAAGCA GAACAAAATG AATTTATT

>Ortholog Group 89, Repeat 1

3 201

H00379401 AGCAATGAGT TACTTCTTAA TCTTAATAAT GGTCAACTTT TGCCACTACA

R00035991 AGCCATGAGT TACTCCTGAA TCTTAATAAT GGTCAACTTT TGCCACTGCA

M00117898 AGTCATGAGC TACTCCTGAA TCTTAATAAT GGTCAACTTT TGCCACTGCA

ACTTCAGGGC CCACTTAATT CATGGATTCC ACCTTTCTCT GGAATTTTA-

ATTTCAGAGC GCCTTCAACT CCTGGATTCC TCCCTTCCCT GGGCTTCTAC

GTTCCAGGGT GCCTTCAACT CCTGGATTCC TCCCTTCCCT GGGTTTCTG-

--GCTCAAAT TCCAGGACTC TCCCAGTTCT CTTTATCAGC TCTAGACCAG

AGGCTCAGGT CTCAGGACGC CCACAGTTTC CTCTCTCAAC ACTAGAGAGC

--GCTCAGGT CTCAGGACGC CCACAGTTTA CTCTCTCAAC ACTAGAGAGC

TTTGCTGGAC TGCTCCCAAA TCAGATACCC TTAACAGGAG AGGCCAGTTT

TTTGCTGGGC TATTCCCAAA TCAGATACCT TTCTCAAGAC AGGTTGGGTT

TTTGCTGGAC TATTCCCAAA TCAGATACCT TTATCAAGAC AGGTTGGACT

T

T

T

>Ortholog Group 8, Repeat 1

3 129

M00105321 ATGGGGCGGC GGGCGCGGGG CCGGCGGTTC CAGCCTGAGG GCGAGGAAGA

H00264670 ATGGGGCGGC GGTCGCGGGG TCGGCGGCTC CGGCCGGAGG ACGCGGAGGA

R00023641 ATGGGGCGGC GGGCGCGGGG CCGGCGGTTC CAGCCTGAGG GCGAGGAAGA

CGCCAGCGAC GGCGGCAGAA AGCGAGGCCA GGCGGGCTGG GAAGGTGGCT

TGGCGCCGAG GGTGGTGGAA AGCGCGGCGA GGCGGGCTGG GAAGGAGGCT

CGGCAGCGAC GGCAGCAGAA AGCGCGGCCA GGAGGGCTGG GAAGGTGGAT

ATCCCGAGAT CGTAAAGGAG AACAAGCTC

ACCCCGAGAT CGTCAAGGAG AACAAGCTG

ATCCCGAGAT CATAAAGGAG AACAAGCTC

>Ortholog Group 90, Repeat 1

3 198

H00336741 ATTGATGAAG CCTGTAAGAG AATAAAGCGT GAAGTTGATG ATTTGGGCCC

M00031061 ATTGATGAGG CCTGTAAGAG AATAAAGCGT GAAGTTGATG ATTTGGGCCC

R00005126 ATTGATGAGG CCTGTAAGAG AATAAAGCGT GAAGTTGATG ATTTGGGTCC

TGAAGTTGGT GACATTAAAA TCATTCCATT GTATTCTACA CTTCCACCTC

TGAAGTTGGT GATATTAAAA TCATTCCATT ATATTCTACA CTTCCACCCC

TGAAGTTGGT GATATTAAAA TCATTCCATT ATATTCTACA CTTCCACCCC

GCATTTTTGA GCCTCCACCT CCCAAAAAAC AGAATGGAGC AATTGGAAGA

GCATTTTTGA GCCACCACCT CCAAAAAAAC AGAATGGAGC AATTGGAAGA

GCATTTTTGA GCCACCACCT CCAAAAAAAC AGAATGGAGC AATTGGAAGA

AAGGTAGTTG TGTCAACTAA CATAGCAGAG ACGTCTTTGA CAATAGAT

AAGGTGGTGG TGTCAACTAA TATTGCAGAG ACCTCTTTGA CAATAGAC

AAGGTGGTTG TGTCAACTAA TATTGCAGAG ACCTCTTTGA CAATAGAT

>Ortholog Group 91, Repeat 1

3 231

H00377528 GTG------- ---------- ---------- ------GGCA GCAAAAGACG

R00015280 AGCGTTCTGG TTCTGAGCTG CGTCTGCTGG TGCTGGGGA- ----------

M00103306 GTG------- ---------- ---------- ------GGCA GCAAAAGACG

ACGAACTGGA GGCTCTCTGA GAGGTAACCC TTCCTCAAGC CAGGTAGATG

---------- ---------- ---------- ---------- ----------

ACGAACTGGA GGCTCTCTGA GAGGGAATGC TTCCTCAAGC CAGGTTGATG

AAGAACAGAT GAATCGTGTG GTAGAGGAGG AACTCAGACA ACAAGAGGAG

---------- ---------- ---------- ---------- ----------

AGGGACAGAT GAATCGCGTG GTTGAGGAGG ATGCGAGACA TCAAGAGGAG

GAGCACACTG CAAGGAATGG TGAAGTTGTT GGAGTAGAAC CTAGACCTGG

---------- ---------- ----GTTTTG CTGCCGGTTC CGCTGCCT--

GAGCACACTG CGCGGAATGG TGAACTTGTG GGTGCAAACC CTAGGCCTGG

AGGCCAAAAT GATTCCCAGC AAGGACAGTT G

---------- ---------- ---------- -

AGACCAGAAC GATACCCAGC AAGGACAAGT G

>Ortholog Group 92, Repeat 1

3 198

H00263674 CCACCGACGC CACCCCCTCG GACATGCTTC CCCCTGGCGG GTCTGCGTTC

R00026047 CCTCCGACGC CACCTCCTCG GACGTGCTTT CCTCTGGCTG GCCTGCGTTC

M00102647 CCTCCGACGC CACCTCCTCG AACGTGCTTT CCTCTGGCTG GCCTGCGTTC

GGCGCGGCCC CTGACCGGGC CGGAGACCGA AGGGAGGCTG CGCCGGCCGG

TGCGCGGCCT CTGTCCGGGT CCGGGATCGA GGGGAGGCGA CGTCGGCAGG

TGCGCGGCCG CTGTCCGGGC CCGGGATCGA GGGGAGGCGA CGTCGGCAGG

AGCGGGCGCA GCGTCCAGCG GATGGTTTAC ATTCTTGGCA TATCTTCTCC

AGCGGGCGCA GCGTCCGGCG GATGGTTTAC ATTCTTGGCA TAGCTTCTCC

AGCGGGCGCA GCGTCCGGCG GATGGTTTAC ATTCTTGGCA TAGCTTCTCC

CAACCGCAGG CCGGGGCCCG GGCCTCCTGC TCCTCCTCCT CCATCGCC

CAACCGCAGG CCGGGGCCCG GGCCTCC--- TCCTCCTCCT CCATTGCC

CAACCGCAGG CCGGGGCCCG GGCCTCC--- TCCTCCTCCT CCATTGCC

>Ortholog Group 93, Repeat 1

3 198

H00324948 GCAGCGCATG ATGAACAGAA AAAACTGGCA GCGTCACAAA TTGAGAAACA

M00072583 GCAGCCCATG ATGAACAGAA AAAGCTGGCA GCATCGCAGA TCGAGAAACA

R00027816 GCAGCGCACG ATGAACAGAA AAAGCTGGCA GCCTCGCAGA TCGAGAAACA

ACGGCAGCAA ATGGACCTTG CTCGCCAACA GCAAGAACAG ATTGCGAGAC

GCGGCAGCAA ATGGACCTTG CTCGCCAACA GCAAGAACAG ATCGCAAGAC

GCGGCAGCAA ATGGACCTTG CTCGCCAACA GCAAGAACAG ATCGCAAGAC

TTCTGCAACA GCAGCACAAA ATTAATCTCC TGCAGCAACA GATCCAGGTT

TTCTACAGCA GCAGCACAAG ATTAATCTCC TGCAGCAACA GATCCAGGTT

TTCTACAGCA GCAGCACAAG ATTAATCTCC TGCAGCAACA GATCCAGGTT

CAGGGTCACA TGCCTCCGCT CATGATCCCA ATTTTTCCAC ATGACCAG

CAGGGGCACA TGCCTCCGCT CATGATCCCA ATTTTTCCAC ATGACCAG

CAGGGGCACA TGCCTCCGCT CATGATCCCA ATTTTTCCAC ATGACCAG

>Ortholog Group 93, Repeat 2

3 198

H00324948 CTATCTAGTC TCAACTCCCC TGCCCTTTTT GGGGATCAGG ATACAGTGAT

M00072583 CTATCCAGTC TCAACTCTCC TGCCCTGTTT GGGGACCAGG ACACAGTGAT

R00027816 CTATCCAGTC TCAACTCTCC TGCCCTGTTT GGGGACCAGG ACACAGTGAT

GAAAGCCATT CAGGAGGCGC GGAAGATGCG AGAGCAGATC CAGCGGGAGC

GAAAGCCATT CAGGAGGCTC GGAAGATGCG AGAACAGATC CAGCGGGAGC

GAAAGCTATC CAGGAGGCTC GGAAGATGCG AGAACAGATC CAGCGGGAGC

CACATGGTGT TGACGGGAAA CTGTCCTCCA TAAATAATAT GGGGCTGAAC

CACACGGAGT TGATGGGAAA CTGTCCTCCA TGAACAACAT GGGGCTGAGC

CGCACGGAGT TGATGGGAAG CTGTCCTCCA TGAACAGCAT GGGGCTGAGC

AGCTGCAGGA ATGAAAAGGA AAGAACGCGC TTTGAGAATT TGGGGCCC

AACTGCAGGA CTGAGAAGGA AAGAACACGC TTTGAGAACC TGGGTCCC

AACTGCAGGA ATGAGAAGGA AAGAACACGC TTTGAGAACC TGGGTCCC

>Ortholog Group 94, Repeat 1

3 102

H00261203 GCTGCTAAAG ACAGCGTCAA GCTGGTGGTG CGATACACCC CAAAAGTTCT

M00020057 GCTGCTAAGG ACAGTGTGAA GCTGGTGGTC AGATACACCC CAAAAGTCCT

R00006083 GCTGCTAAGG ACAGTGTGAA GCTGGTCGTC AGATACACCC CAAAAGTCCT

GGAAGAAATG GAGGCTCGCT TTGAAAAGCT ACGAACAGCC AGGCGTCGGT

GGAAGAGATG GAGGCTCGTT TCGAGAAGCT GCGGACAGCT CGGCGTCGAT

GGAGGAGATG GAGGCTCGTT TTGAAAAGCT GAGGACAGCT CGGCGTCGGT

TG

TG

TG

>Ortholog Group 94, Repeat 2

3 21

H00261203 ATTACACAAC AAAACCACAT G

M00020057 ATTCCACAAC AAAACCACAT G

R00006083 ATTCCACAAC AAAACCACAT G

>Ortholog Group 95, Repeat 1

3 198

H00229088 CGAAGTGAAT ACCATGCAGC TTTTAACAGT ATGATGATGG AACGCATGAC

R00024679 CGAAGTGAGT ACCACGCAGC GTTCAACAGC ATGATGACAG AGCGGATGAC

M00070488 CGGAGTGAGT ACCATGCAGC GTTCAACAGT ATGATGATGG AGCGGATGAC

CACAGATATC AATGCACTGA AGCGGCAGTA CTCTCGAATT AAAAAGAAGG

CACGGACATC AACGCCCTGA AGAGGCAGTA CTCGCGCATT AAGAAGAAGC

CACGGACATC AACGCCCTGA AGCGGCAGTA CTCCCGAATT AAGAAGAAGC

TTCATCAGGT GTACATCAGG GCAGACAAAG GGCCAGTGAC CAGCATTCTC

TCCACCAGGT GTACATAAGA GCAGACAAAG GGCCAGTGAC CAGCATCCTC

TCCATCAGGT GTACATAAGG GCAGACAAAG GGCCGGTGAC CAGCATCCTC

CCGTCTCAGG TAAACAGTTC TCCAGTTATA AACCACCTTC TTTTAGGA

CCATCTCAGG CAAATAGTTC TCCGGTGATC AACCATCTTC TTCTAGGG

CCATCGCAGG CAAATAGTTC TCCCGTGATC AACCACCTTC TTCTGGGG

>Ortholog Group 96, Repeat 1

3 198

R00019865 CTCTTGCCCA ACAACAACAC TCATCCGGGC CCTTTCCCTC CT---ACCGG

H00379915 CTCTTAGCCA ACAGCAATGC CCATCCAGGC CCCTTCCCTC CCTCTACTGG

M00115407 CTCTTGGCCA ACAACAACAC CCATCCAGGC CCTTTCCCTC CT---ACTGG

GGGTCAATCC ACAGCCCACC CGCCAGCCCC TGCACATCAC CATCAC---C

GGCCCAGTCC ACCGCCCACC CACCAGTCTC AACACATCAC CATCACCACC

GGGTCAATCT ACAGCCCACC CAGCAGCCCC TACACATCAC CATCAC---C

ATCATGGAAA CTCTGGGCCC CCTCCACCTG GGGCATATCC TCACCCCCTA

ATCACGGAAA CTCTGGGCCC CCTCCTCCTG GAGCATTTCC CCACCCACTG

ATCATGGAAA CTCTGGGCCC CCTCCACCCG GAGCGTATCC TCACCCTCTA

GAGAGCAGTA ACTCCCACCA TGCACACCCT TACAACATGT CACCCTCC

GAGGGCGGTA GCTCCCACCA CGCACACCCT TACGCCATGT CTCCCTCC

GAGAGCAGTA ACTCCCATCA TGCACACCCT TACAACATGT CACCCTCC

>Ortholog Group 97, Repeat 1

3 156

R00020529 AGTGTGGGCA TGAACCTGAC TCGGACAGCC ACACCTGCCC CCAGCCAGAC

H00399194 AATGTGGGCA TGAACCTGAC ACGGACAGCC ACACCTGCGC CCAGCCAGAC

M00125568 AGTGTGGGCA TGAACCTGAC TCGGACAGCC ACACCTGCCC CCAGCCAGAC

GCTTATTAGC TCAGCCACAT ACACACAGAT CCAGCCCCAT TCCCTGATTA

ACTTATTAGC TCAGCCACCT ACACACAGAT CCAGCCCCAT TCACTGATTA

GCTTATTAGC TCAGCCACAT ACACACAGAT CCAGCCCCAT TCACTGATTA

TCCACCTCCA GCAGAAGCAA GTGGTGATCC AGCAGCAGAT TGCCATCCAC

TCCACCTCCA GCAGAAACAG GTGGTGATCC AGCAGCAGAT TGCCATCCAC

TCCACCTCCA GCAGAAGCAA GTGGTGATCC AGCAGCAGAT TGCCATCCAC

CACCAG

CACCAG

CACCAG

>Ortholog Group 97, Repeat 2

3 192

R00020529 CAGTTCCAAC ACCGCCAGTC CCAGCTACTT CACACAGCCA CGCACCTCCA

H00399194 CAGTTCCAGC ACCGGCAGTC CCAGCTCCTT CACACAGCTA CACACCTCCA

M00125568 CAGTTCCAAC ACCGCCAGTC CCAGCTGCTT CACACAGCCA CACACCTCCA

GTTGGCCCAG CAGCAGCAGC AGCAGCAGCA ACAGCAGCAG CAGCAGCAAG

GTTGGCG--- ---------- ---------- ---------- ---CCGCAAG

GTTGGCC--- ---------- --------CA GCAGCAGCAG CAGCAGCAAG

CAACAACTCT CGCTGCCCCT CAGCCACCCC AGGTCCCACC TACTCAGCAG

CCACCACCCT CACTGCCCCT CAGCCACCAC AGGTCCCACC TACTCAGCAG

GAACAACCCT CACTGCCCCT CAGCCACCCC AGGTCCCACC TACTCAGCAG

GTCCCACCTT CCCAGTCGCA ACAGCAAGCC CAGACTCTGG TG

GTCCCACCTT CCCAGTCCCA GCAGCAAGCC CAAACCCTGG TC

GTCCCACCTT CCCAATCACA ACAGCAAGCC CAGACTCTGG TG

>Ortholog Group 98, Repeat 1

3 198

H00352567 CGGAACACAG CTAGCTTTAG GAAGACTGCT GAACGCCTGC TGAGATTTTC

M00040074 CAGAACACAG CTAGCTTTAG AAAGACTGCC GAGCGCCTAC TGAGGTTTTC

R00001093 CAGAACACAG CTAGCTTTCG GAAGACTGCT GAGCGCCTAC TGAGGTTTTC

AGATAAGAGA CAGACTGAGG AGGCCATTGA CAGGATTTTT GCCATATCAA

AGATAAGAGA CAGACAGAGG AAGCCATTGA CAGAATTTTT GCTATATCCG

GGATAAGAGA CAGACAGAGG AGGCCATTGA CAGGATCTTT GCTATATCCG

GCAAGTCAAA GAAAAACCGA AGGGCAGGCA AACGCTATAA ATTTGTGGAT

GCAAGTCGAA GAAAAGCCGA AAGGCGGGTA AGCATTACAA GTTTGTGGAT

GCAAGTCAAA GAAAAGCCGA AGGACCGGCA AGCATTACAA GTTTGTGGAT

GCTGTCCCTG ATATTTTTGC ACAGATTGAA GTCAATGAGA AAAAGATT

GCGGTTCCTG ATATTTTCGC ACAGATTGAA GTTAATGAGA AGAAGATT

GCCGTCCCTG ATATTTTTGC ACAGATTGAA GTCAATGAGA AGAAGATT

>Ortholog Group 99, Repeat 1

3 201

M00093356 CAACCTTCTG TGAGGACTCA GATGTGGCTG ACAGAGCAGT TACGGACAAA

H00357477 CAGCCTTCCG TAAGGACTCA GATGTGGCTT ACAGAGCAGC TGCGGACAAA

R00058939 CAACCTTCTG TGAGGACTCA GATGTGGCTT ACAGAGCAGT TACGGACAAA

CCCATTGGAA GGCAGAACCA CAGACGATTC TTACAGTTTA GCTCCTTGGA

TCCTTTGGAA GGTAGAAATA CAGAGGATTC TTACAGTTTA GCTCCTTGGA

CCCATTGGAA GGCAGAACCA CAGAGGATTC TTACAGTTTA GCTCCTTGGA

CTGAAGAGTT TCAGCAAGGA AGTGAGACGC CAATGCAGGT TTTGACTGGA

TTGAAGACTT TCGACAAGGA AGTGAAACAC CAATGCAGGT TTTGACTGGA

CTGAAGAGTT TCAGCAAGGA AGTGAGACAC CAATGCAGGT TTTGACTGGA

TCATCTCGTC AGAGTTATTC ACCTCCTGGC TTCCAGGATT TCAGTAAATG

TCATCTCGTC AAAGTTATTC A---CCTGGC TATCAGGATT TCAGTAAGTG

TCATCTCGTC AGACTTATTC ACCTCCTGGT TTCCAGGATT TCAGTAAATG

G

G

G

>Ortholog Group 9, Repeat 1

3 198

H00339299 GAGCTGTGGA CGTGGCTGGA GGAGCTGCAG AAGGAGCTGC TGGACGACGT

M00087714 GAGCTCTGGA CATGGCTGGA GGAGTTACAG AAGGAACTGC TGGACGACGT

R00016784 GAGCTCTGGA CATGGCTGGA GGAGCTGCAG AAGGAACTGC TGGACGACGT

GTATGCCGAG TCGGTGGAGG CCGTGCAGGA CCTCATCAAG CGCTTTGGCA

GTACGCCGAG TCGGTGGAGG CCGTGCAGGA CCTCATCAAG CGATTCGGCA

GTACGCCGAG TCGGTGGAGG CCGTCCAGGA TCTCATCAAG AGATTCGGCA

CCACCCTGCA GGTGACTGTC AACGTGATCA AGGAAGGGGA GGACCTCATC

CCACCCTGCA GGTGACTGTC AACGTGATCA AGGAAGGAGA AGACCTCATC

CCACCCTGCA GGTGACAGTC AACGTGATCA AGGAAGGGGA GGACCTCATC

CAGCAGCTCA GGGACTCTGC CATCTCCAGT AACAAGACCC CCCACAAC

CAGCAGCTGA GGGACTCCGC CATCTCCAGT AACAAAACTC CGCACAAC

CAGCAGCTGA GGGACTCTGC CATCTCCAGT AACAAGACGC CGCACAAC

>Ortholog Group 100, Repeat 1

3 198

H00343948 GCGTCACGCA TAGGGTTGCG CATGCAGCTC ATGCGGGAGC AGGCGCAGCA

M00024786 GCGTCACGCA TCGGGCTGCG CATGCAGCTC ATGCGGGAGC AGGCCCAGCA

R00037517 GCGTCGCGCA TCGGGCTGCG CATGCAGCTC ATGCGGGAGC AGGCGCAGCA

GGAGGAGCAG CGGGAGCGCA TGCAGCAACA GGCTGTCATG CATTACATGC

GGAGGAGCAG CGAGAGCGCA TGCAGCAGCA GGCTGTCATG CATTATATGC

GGAGGAGCAG CGAGAGCGCA TGCAGCAGCA GGCCGTCATG CATTACATGC

TCGGAGGGCC GCCCACCCCG GCCATCAATA CCCCCGTCCA CTTCCAGTCG

TGGGTGGGCC CCCCACCCCA GCCATCAACA CCCCTGTCCA CTTCCAGTCG

TGGGAGGCCC TCCCACCCCA GCCATCAACA CCCCAGTTCA CTTCCAGTCG

CCACCACCTG TGCCTGGGGA GGTGTTGAAG GTGCAGTCCT ACCTGGAG

CCCCCGCCTG TGCCCGGGGA GGTGCTGAAG GTGCAGTCCT ACCTGGAG

CCACCACCAG TGCCTGGGGA GGTGCTGAAG GTGCAGTCCT ACCTGGAG

>Ortholog Group 101, Repeat 1

3 198

M00124317 ATTCCCATCT TTAGTCCAAT GATGCCTTAC GGCACAGGAC TTACTCCACA

R00002038 ATTCCCATCT TCAGTCCAAT GATGCCTTAC GGCACAGGGC TTACTCCACA

H00230354 ATCCCTATCT TTAGTCCAAT GATGCCTTAT GGCACTGGAC TGACCCCACA

GCCTATTCAG AACACCAACA GTCTCTCTAT TTTGGAAGAG CAACAAAGAG

GCCTGTTCAG AACACCAATA GTTTATCTAT TTTGGAAGAG CAACAAAGAG

GCCTATTCAG AACACCAATA GTCTGTCTAT TTTGGAAGAG CAACAAAGGG

CAACTGCAGC AGCCTCAGTA CAGCAATCAA CATCTCAGCA ACCCACACAG

CAACTGCAGC AGCCTCAGTA CAGCAATCAA CATCTCAGCA GCCCACACAG

CAGTGGCAGC TGCAGCCGTT CAGCAGTCAA CGTCCCAGCA GGCAACACAG

GGTGCCTCAG GCCAGACCCC ACAACTCTTC CATTCTCAAA CTCTGACC

GGTGCCTCAG GCCAGACCCC ACAACTCTTC CATTCTCAAA CTCTAACC

GGAACCTCAG GCCAGGCACC ACAGCTCTTC CACTCACAGA CTCTCACA

>Ortholog Group 102, Repeat 1

3 198

M00064839 TTGGATGATT ACATGAAATG TTTTAAGACT CCAGTTGTAA AGAATGACTT

R00047739 TTGGATGATT ACATGAAATG TTTTAAGACT TCAGTTGTAA AGAATGAATT

H00358813 TTGGATGATT ACATGAGCTG TTTTAGAACT CCAGTTGTAA AGAATGACTT

TCCACCTGCC TGTCCATCAT CAACACCTTA CAGCCAGCTT GCCCGCCTCC

CCCACCTGCC TGTCCATCAT CAACACCTTA CAGCCAACTT GCCCGCCACC

TCCACCTGCT TGTCAGTTGT CAACACCTTA TGGCCAACCT GCCTGTTTCC

AGCAGGGACT CAGCACTCCT CTTCAAAGCT TGCAGATTTC AGGTTCTTCA

AGCAAGGGCT CAGCACTCCT CTTCAAAACT TACAGATTTC AGGATCCTCA

ATCAAATACT TGCCACTCCA CTTCAAAATT TACAGGTTTT AGCATCTTCT

TCAATAAATG AATGCATTTC AGTTAACGGA AGAATTTATT CCATATTA

TCAATGAGTG AATGCATTTC AGTTAACGGA AGAATTTATT CCATATTA

TCAGCAAATG AATGCATTTC GGTTAAAGGA AGAATTTATT CCATATTA

>Ortholog Group 103, Repeat 1

3 198

R00020780 CAAGAGTTCA CCGCAGCGCG GGACGGTTCA GCACTTGAGA AGGAAGGAGT

M00037788 CAAGAGTTCA CAGCAACTCG GGACAGTTCA GCACTTGAAA AGGAAGGAAT

H00264935 GAAGAATTCA CAGCAGCTCA GGACAGCTCT GCGATGGTGG GTGAAGATGT

TGGGTACCTG TCTCTGGAGA ACAAGGCTCT GCAAAGCCGC CTTACGGAGT

TGGTTACCTA TCTCTTGAAA ATAAGACTCT GCAAAGCCGC CTTGCTGAGT

CGGCTCCCTG GCTCTGGAGA GTAAGTCCCT GCAAAGCCGC CTTGCTGAGC

ATGCTGCTGC GGTGACCAAG GTGACGACAG AGCTCAACAA CACAAAGAGG

ATACTGCTAC AGTGACCAAG ATGACAGCAG AGCTCAATAA TACAAAGAGG

ACGCCCGGGA GATGAGCGAG GTGACGGCGG AGCTGCACCA CACACACAAG

GAGCTGGACA CATTGAGACA ACATTTGGAC AACTCATTGG AAGAGAAT

GAGCTGGACA CATTGAGACA ACATTTGGAC AAATCATTGG AAGAGAAT

GAGCTGGATG ATTTGAGACA ACATTTAGAT AAATCTTTGG AAGAGAAC

>Ortholog Group 104, Repeat 1

3 198

H00231524 GCAGCCTGCC TCCACTGTGA AAAGACTTTG CAGCAGGATG ATTGTAGAGT

R00016647 ACAGCCTGTC TCCATTGT-- -AAGACTTTG CAGCAGGATG ATTGCAGAGT

M00022225 ACAGCCTGTC TCCACTGTGA AAAAACTCTG CAGCAGGATG ATTGCAGAGT

TGTCTTGGCA AAACAGGAAA TTACAAGGTT ACTGGAAACA TTGCAGAAAT

TGTCTTGGCA AAACAAGAAA TCACAAGATT ATTAGAAACA CTGCAGAAAT

TGTCTTGGCA AAACAAGAAA TCACAAGATT ACTAGAAACA CTGCAGAAAT

TTACAGAAGT TGCAGATCAC ATTCAGTTGG ATGCCAGCAT CCCTGTCACT

TTACAGAAGT TGCAGATCAT ATTCAGTTGG ATGCCAGTAT TCCAGTCACT

TTACAGAGGT TGCAGATCAT ATTCAGTTGG ATGCCAGTAT TCCAGTCACT

TTTACAAAGG ATAATCGAGT TCACATTGGA CCAAAAATGG AAATTCGG

TTTACAAAGG ACAACAGAGT TCATATTGGA CCCAAAATGG AAATCCGA

TTTACAAAGG ACAATAGAGT TCATATTGGA CCCAAAATGG AAATCCGA

>Ortholog Group 105, Repeat 1

3 207

H00231656 AAATCAGAGC TGGCTGCCAA TCTGGGGCTC ACTGAACGGC AGGTGAAGAT

M00025521 AAGTCCGAGC TGGCTGCTAA CCTGGGGCTC ACAGAGCGGC AGGTAAAGAT

R00025141 AAGTCAGAGC TGGCTGCTAA CTTGGGTCTC ACAGAGCGGC AGGTAAAGAT

CTGGTTCCAA AACCGGCGGG CAAAGGAGCG CAAAGTGAAC AAGAAGAAAC

CTGGTTCCAG AACCGCCGGG CCAAGGAGCG CAAAGTAAAC AAGAAGAAAC

CTGGTTCCAG AACCGTCGGG CCAAGGAGCG TAAAGTAAAC AAGAAGAAAC

CCCCACAGCC G--------- CCGATGGCCC ACGACATCAC GGCCACCCCA

CCCTGCCTCC CACACAGCTG CCCCTGCCCC TGGATGGCAC TCCCACACCA

CCATGCCTCC CACACAGTTG CCCCTGCCCC TGGATGGCAC CCCCACACCA

GCCGGGCCAT CCCTGGGGGG CCTGTGTCCC AGCAACACCA GCCTCCTGGC

TCAGGGCCAC CCCTAGGAAG TCTATGCCCT ACTAATGCTG GCCTTCTGGG

TCGGGGCCAC CCCTGGGGAG TCTATGCCCC ACCAATGCTG GTCTTCTGGG

CACCTCC

CACCCCC

CACCCCC

>Ortholog Group 106, Repeat 1

3 198

H00424765 ACTGCTGTCA CTGACCCACG GTACCGTGCC CGCGGCAGCC CGCACTTCCA

R00058797 GCGGCTGTCA ATGACCCAAG ATACCGTCCG AGAAGCAGCC CACACTTCCA

M00121113 GCGGCTGTCA CTGACCCAAG ATACCGACCC CGAAGCAGCC CACACTTCCA

GCATGCTGAA GTCAGGATCC TGCAGGCCCA GGTGCCTCCT GTGTTCCTCT

GCATGCTGAA GTCAGGATCC TGCAGGCACA GGTACCACCG GTTTTCCTCT

GCATGCCGAA GTCAGGATCC TGCAGGCCCA GGTACCACCG GTGTTCCTCT

ACCAGTACCT GCAGCAATCT CAGGAGCACC CCCCTCCCCC ACATCCAGCT

ACCAGTACCT GCAGCAACCC CAGGAGCACT CCCCACCCCT CCATCCAGCC

ACCAGTACCT GCCACAGCCC CAGGAGCACT CTCCACCCCT CCACCCGGCA

GCTCTCGGCC ATGGCCCCCT GAGCTCCCTC AGTCCACCTG CTGTGGAG

GCTCTAGGCC ATGGTCCCCC AAGTTCCTTC AGTCCACCTG CCCTGGAG

GCTCTGGGCC ATGGACCCCC AAGCTCCTTT GGTCCACCTG CAGTGGAG

>Ortholog Group 107, Repeat 1

3 177

M00108954 CAGCAGCAGC AGCAACAGCA GCAACAGCAG ---------- ----------

H00420736 TACAGCAAGA AGACGGAAAT CCAAAGGCAG ACAGTACGGG CTCCCTTCGC

R00013271 CAGCAGCAGC AGCAACAGCA GCAGCAGCAG ---------- ----------

---------- ---------- -----CAGCA ACAACAGCAG CAGCAGCAAG

CAAACTCTTC ATTTTCTCTG CACTTCAGGT GGCAAGACAG CTCCTTCTTG

---------- ---------- -----CAGCA GCAGCAGCAG CAACAGCAGG

TTAGTGGATT AAAGTCTCCC AAGAGGAATG ACAAGCAACC AGCTCTTCAG

TTAGTGGATT AAAATCTCCC AAGAGGAATG ACAAACAACC AGCTCTTCAG

TTAGTGGATT AAAGTCTCCG AAGAGGAATG ACAAGCAACC AGCTCTTCAG

GTTCCCGTGT CAGTGGCTAT GATGACA

GTTCCCGTGT CAGTGGCTAT GATGACA

GTTCCCGTGT CAGTGGCTAT GATGACA

>Ortholog Group 107, Repeat 2

3 84

M00108954 CAGGTTATCA CTCCTCAACA AATGCAGCAG ATCCTCCAGC AGCAGGTGCT

H00420736 CAAGTTATCA CTCCCCAGCA AATGCAGCAG ATCCTCCAGC AACAAGTGCT

R00013271 CAGGTTATCA CTCCTCAACA AATGCAGCAG ATCCTCCAGC AGCAAGTGCT

GAGCCCCCAG CAGCTCCAGG TTCTCCTCGC CCTC

GAGCCCTCAG CAGCTCCAGG TTCTCCTCGC CCTC

GAGCCCACAG CAGCTCCAGG TTCTCCTCGC CCTC

>Ortholog Group 107, Repeat 3

3 111

M00108954 ATGCTTCTTC AAGAATTTTA TAAAAAACAA CAGGAACAGT TGCAGCTTCA

H00420736 ATGCTTCTTC AAGAGTTTTA TAAAAAACAA CAGGAACAGT TGCAGCTTCA

R00013271 ATGCTTCTTC AAGAATTTTA CAAGAAACAA CAGGAACAGT TGCAGCTTCA

ACTTCTCCAA CAGCAACATG CTGGAAAACA GCCGAAAGAG ------CAGC

ACTTTTACAA CAACAACATG CTGGAAAACA GCCTAAAGAG ------CAAC

ACTCCTCCAA CAGCAACATG CTGGAAAACA GCCGAAAGAG CAGCAGCAGC

AGCAGGTGGC T

AGCAGGTGGC T

AGCAGGTGGC T

>Ortholog Group 108, Repeat 1

3 198

R00011668 TACCCAGACA TCTTCATGCG CGAGGAGGTG GCTCTCAAGA TCAACCTGCC

H00282549 TACCCTGACA TCTTCATGCG GGAGGAGGTG GCGCTCAAGA TCAACCTGCC

M00006071 TACCCAGACA TCTTCATGCG CGAGGAGGTG GCACTCAAGA TCAACCTGCC

CGAGTCCAGA GTCCAGGTTT GGTTCAAGAA CCGCCGAGCC AAGTGCCGCA

GGAGTCTAGA GTCCAGGTCT GGTTCAAGAA CCGCCGCGCC AAATGCCGCA

AGAGTCCAGA GTCCAGGTTT GGTTCAAGAA CCGCCGCGCC AAGTGCCGCA

GCGGGAATGG AACGAAAAGC CGGCCGGTCA AGAAGAAGTC GTCTCCGGTG

GCGGGAGCGG AACCAAGAGC CGCCCAGCCA AGAAGAAGTC CTCTCCAGTG

GCGGGAATGG AACGAAAACC CGGCCGGTCA AGAAGAAGTC GTCTCCAGTA

CGCGAGAGCT CGGGTTCCGA GAGCAGCGGC CAGTTCACGC CGCCCGCC

CGGGAGAGCT CGGGCTCCGA AAGCAGTGGC CAATTCACGC CGCCAGCT

CGCGAGAGCT CGGGTTCAGA GAGCAGCGGC CAGTTCACGC CGCCCGCC

>Ortholog Group 109, Repeat 1

3 132

H00386456 GCTCCTCCCA CTCCACAGCA GACGCCTTCT ACTCAGGCCC AGGGTCTGCC

M00086948 GCTCCACCCA CCCCACAGCA GACACCTGCT ACCCAGACAC AAGGTTTGCC

R00044665 GCTCCACCCA CCCCACAGCA GACGCCTGCT CCCCAGACCC AGGGTCTGCC

CGCTCAGGCC CAGGCCACAC CCCAGCACCA GCAGCAACTC TTCCTCAAG-

CACCCAGGCC CAGGCCACTC CCCAGCACCA GCAGCAACAC CTCCTCAAG-

CACCCAGGCC CAGGCTACTC CTCAGCACCA GCAGCAGCTC CTCCTCAAGC

---------- ----CCACCG CCAGCACAGC AG

---------- ----CAGCCT ACAGCACCAC CA

AGCAGCAGCA GCAGCAGCCA ACAGCACCAC CA

>Ortholog Group 109, Repeat 2

3 93

H00386456 CCGGCAGGCA CGTTTTAC-- ---------- ------GCCC AGACTCAGCA

M00086948 CCTGCAGGCA CCTTTTACCA GCAGCAGCAG CAACAGGCTC AGACTCAGCA

R00044665 CCATCAGGCA CCTTTTAC-- -CAGCAGCAG CAGCCCGCTC AGACTCAGCA

GTTTCAGGCA GTACATCCAG CAACCCAGAA ACCAGCAATT GCT

GTTTCAGGCA GTACATCCAG CAGCCCAGCA ACCAGTCACT GCT

GTTTCAGGCA GTGCATCCAG CAGCCCAGCA ATCAGTCACT GCT

>Ortholog Group 109, Repeat 3

3 156

H00386456 CAGTTCCCTG TGGTGTCCCA AGGAGGCTCT CAACAGCAGC TAATGCAGAA

M00086948 CAGTTCCCTG TGGGGTCCCA GGGAGGTGCT CAGCAACAGC TGATGCAGAA

R00044665 CAGTTCCCTG TGGTGTCCCA GGGAGGCTCT CAGCAACAGC TGATGCAGAA

TTTCTACCTG GCCACAGCCC TGCATCAACA ACAGCTGATG ACTCAGCAGG

CTTCTAC--- ---------- -----CAGCA GCAGCTGATG GCTCAGCAGG

CTTCTAC--- ---------- -----CAGCA ACAGCTGATG GCTCAGCAGG

CTGCCTTGCA GCAAAAGCCC ACTATGGCAG CAGGACAGCA GCCCCAGCCA

CTGCCCTGCA GCAGAAG--- ACTGCTGTGG TAGTACCACA GTCTCAGGCA

CCGCCCTGCA GCAGAAG--- ACTGCTGTGG TAGTACCACA GCCTCAGGCA

CAGCCA

CAGCCC

CAGCCA

>Ortholog Group 10, Repeat 1

3 207

R00022939 ---------- ---------- ---------- ---------- ----------

H00356116 CACCAGCAGC ACCACCACCA CCACCATGCC CACCACCACC ACCACCATGC

M00090398 CATCAGCAGC ACCACCACCA CCATGCCCAC CACCACCACC ACCACCATGC

---------- ---------- ---------- ---------- ----------

C--------- CACCACCTCC ACCACCACCA CGCACTACAG CAGCAGCTAA

CCACCACCTC CACCACCTCC ACCACCACCA CGCACTACAG CAGCAGCTAA

---------- ---------- ---------- ---------- ----------

ACCAGTTCCA TCCCATTTCC AACAACAACA GCTTGGGCGG CGCGGGCGGC

ACCAGTTCCA TCCCACTGCC ---AACAACA GCCTGGGCGG TGCGGGCGGC

---------- ---------- ---------- ---------- ----------

GGCGCGCCTC AGCCCGGCCC CGACATGGAG CAGCCGCAAC ATGGAGGCGC

GGCGCGCCTC AGCCCGGCCC GGACATGGAG CAGCCGCAAC ATGGAGGCGC

-------

CAAGGAC

CAAGGAC

>Ortholog Group 110, Repeat 1

3 207

R00017064 CACACGAGCC AG-------- ---------- ---------- --------GG

H00234389 CACACCAGCC AGAAAATCCA CCGCGCCCTC AACACGGAGC CACCAGAGGG

M00048576 CACACGAGCC AGAAGATCCA CCGAGCCCTC AACACAGGGC CACCAGAGGG

G---CAACAG GAGAGGGCAG AGCACGAGCG CAGCGGCCCC ---------G

GTCGAAGGAG GAGACGGCAG AGGCGGAGCC CAGCGGCCCC GAGGTGGAGG

G---CAACAG GAGAGGGCAG AGCAGGAGTG CAGCGGCCCC ---------G

AGCTGCCTGC CACCGATGGT GCAGGGCGCT GGAGGCGGGT GCGCCGGGCT

ACCAGCCAAC GGCTCCGGAG GGC------T GGAAACGGGC GCGCCGGGCC

AGCAACCTGC AGCCGACGGT GCGGGGCGCT GGAGGCGGGT GCGCCGGGCC

---GTGGAAC GGGAGCGACG CGTGCGTTTC CTGCTGGAAC CTGGG-----

---GTGGACA AGGAGCGCCG CGTGCGCTTC CTGCTGGAGC CCGCCGTGGT

GTGGTGGAAC GGGAACGGCG CGTGCGTTTC CTGCTGGAAC CTGGG-----

-------

TGTGGCA

-------

>Ortholog Group 111, Repeat 1

3 198

H00365007 ATTCAAATCA GTAAGGCTTA CGAGATTCTT TCAAATGAAG AAAAGAGATC

M00048714 ATTCAGATCA GCAAGGCATA CGAGATCCTG TCCAATGAGG AAAAGAGGAC

R00017247 ATTCAGATCA GCAAGGCTTA TGAGATTCTG TCCAATGAGG AGAAGAGGAC

AAATTATGAT CAATATGGAG ACGCTGGAGA GAACCAGGGC TACCAGAAGC

AAACTACGAC CACTATGGTG ATGCCGGTGA GAACCAAGGC TACCAGAAGC

AAACTATGAC CACTACGGTG ATGCGGGTGA GAACCAAGGC TACCAG---C

GAGAGTATCG CTTCCGCCAT TTCCATGAAA ATTTTTATTT TGATGAATCC

GCGAACACCG CTTCCGCCAT TTCCACGAGA ACTTCTATTT TGATGAGTCC

GCGAGTACCG CTTCCGCCAT TTCCACGAGA ACTTCTATTT TGATGAATCC

TTTTTTCACT TCCCTTTTAA TTCTGAACGG CGGGACTCAA TTGACGAA

TTTTTCCACT TCCCCTTCAA TGCGGAGCGG CGGGACTCGG GTGACGAG

TTTTTTCACT TCCCTTTCAA TTCCGAGCGG CGGGACTCAA TCGATGAG

>Ortholog Group 112, Repeat 1

3 132

H00349748 CCGCCGCCCG GCATGGGCCT CAATCAGAAT CGCGGCCCCA TGGGTCCTGG

R00018049 ---------- ---------- ---------- ---------- ----------

M00030623 CCGCCGCCGG GCATGGGCCT CAACCAGAAC CGCGGCCCCA TGGGCCCGGG

CCCGGGCCAG AGCGGCCCTA AGCCTCCGAT CCCGCCACCG CCTCCACACC

---------- ---------- ---------- ---------- ----------

CCCTGGC--- ---GGCCCGA AGCCGCCGCT CCCGCCTCCA CCTCCTCACC

CACCACCGCA GCAGCCACCG CCGCAGCAGC CG

---------- ---------- ---------- --

CGCCGCCGCA GCAGCCTCCG CCGCAGCAGC CG

>Ortholog Group 112, Repeat 2

3 135

H00349748 CCGCAT---C AGCCGCCGCC GCATCCACAG CCGCATCCGC CGCCACCGCC

R00018049 ---------- ---------- ---------- ------CCCC CGCCTCCGCC

M00030623 CCGCACCAGC AGCCGCCGCC GCACCAGCCG CCCCATCCCC CGCCTCCGCC

GCAGGACTCT TCCAAGCCCG TCGTTGCTCA GGGACCCGGC CCCGCTCCCG

GCAGGAA--- TCCAAGCCCG TCGTCCCGCA AGGCCCCGGC TCGGCGCCCG

GCAGGAA--- TCCAAGCCCG TCGTCCCCCA AGGCCCCGGC TCGGCGCCGG

GAGTAGGCAG CGCACCACCA GCCTCCAGCT CGGCC

GAGTGAGCAC TGCGCCGCCT CCGGGGGTCT CGGCC

GGGTGAGCAG TGCGCCTCCG CCGGCGGTCT CGGCT

>Ortholog Group 113, Repeat 1

3 198

M00041241 CCTCAACAGT CACAGCCACC TTCCCAGCCA CCCCTTACAT CTTTACCAGC

R00048900 CCTCAACAAT CACAGCCACC TTCCCAGCCA CCCCTTACAT CTTTACCAGC

H00425133 CCTCAACAAT CACAGCCACC TTCCCAGCAA CCCCTTACAT CTTTACCAGC

TCAGCCAACA GCACAGTCTA CAAGCCAATT GCAGGTTCAA GCTCTAGCTT

TCAGCCAACA GCACAGTCTA CAAGCCAGTT GCAGGTTCAA GCTCTAGCTT

TCAGCCAACA GCACAGTCTA CAAGCCAGCT GCAGGTTCAA GCTCTAACTT

CCCCTACAAA AGTCATACCA GCTTTGGGGA AAAGCCCGCC TCACCACTCT

CCCCTACAAA AGTCATACCA GCCTTGGGGA AAAGCCCGCC TCACCACTCT

CCCCTACAAA AGCTGTGCCG GCTTTGGGGA AAAGCCCGCC TCACCACTCT

GGATTCCAGC AGTATCAACA GGCAGATGCC TCCAAACAGC TGTGGAAT

GGATTCCAGC AGTATCAACA GGCAGATGCC TCCAAACAGC TGTGGAAT

GGATTCCAGC AGTATCAACA GGCAGATGCC TCCAAACAGC TGTGGAAT

>Ortholog Group 114, Repeat 1

3 207

H00375928 CCTCGGCCGG CTGTATTATC TGGCTATTTC AAACAGTTTC AGAAGTCTTT

R00039138 ---------- ---------- ---------- ---------- ----------

M00028016 CCTCGACCT- ---------- -GGCTATTTC AAACAGTTTC AGAAATCATT

ACCTCCACGA TTCCAGCGGC AGCAGGAACA GATGAAACAG CAGCAGTGG-

---------- ---------- ---------- ---------- ----------

GCCACCACGA TTTCAGCGTC AGCAGGAGCA GATGAAACAG CAACAGTGGC

--------GG TGTACTTCCA CAGACTGTTC CTTCACAACC GTCCAGTAGT

---------- ---------- ---------- ---------- ----------

AGCAGCAGGG TGTGCTTCCA CAGACTGTGC CCTCACAGCC GTCTAATGGT

ACTGTCCCTC CTCCACCACA CAGACCTCTT TATCAGCCTA TGCAGCCTCA

---------- ---------- ---------- ---------- ----------

TCAGTCCCTC CTCCACCACA CAGACCCCTT TACCAGCCAA TGCAACCCCA

TCCTCAG

-------

CCCTCAA

>Ortholog Group 114, Repeat 2

3 198

H00375928 CCAAATATTT GTAAAGTGAA ACCTCAGCAG TTACAGACAA GCAGCCTGCC

R00039138 ---------- ---------- ---------- ---------- ----------

M00028016 CCGAATATTT GTAAAGTAAA ACCCCAACAA TTACAGACCA GCAGCCTACC

TTCTGCAAGT CATTTTTCAC AGTTAAGCTG TATGCCTTCC CTTATTGCCA

---------- ---------- ---------- ---------- ----------

TTCTGCAAGT CACTTTTCAC AGTTGAGCTG TATGCCTTCC CTCATTGCAA

ATCCGCAAGT TTATGTGTCT CAGTCTGCAG CAGCTCAAAT CCCAGCCTTC

---------- ---------- ---------- ---------- ----------

GCCCGCAGGT TTATGTGTCT CAGTCTGCAG CAGCTCAAAT CCCAGCCTTC

TATATGGACA CAAGTCATTT ATTCAATACC CAACATGCAC GATTGGCT

---------- ---------- ---------- ---------- --------

TATATGGATA CAAGTCATTT ATTCAATACG CAGCATGCGC GATTGGCT

>Ortholog Group 115, Repeat 1

3 198

M00010319 GTGGCATTAA GGGGCAATGA AAACGAAAGA GAGATGGCCC CGCAGTCTGT

R00004905 GTGGCATTAA GGGGCAATGA AAACGAAAGA GAGATGGCCC CGCAGTCTGT

H00400694 GTGGCATTAA GGGGCAATGA AAATGAAAGA GAGATGGCCC CGCAGTCTGT

GAGTCCCCGA GAAAGTTACA GAGAAAACAA ACGCAAGCAG AAGCTGCCCA

GAGTCCCCGA GAAAGTTACA GAGAAAACAA ACGCAAGCAG AAGCTGCCCA

GAGTCCCCGA GAAAGTTACA GAGAAAACAA ACGCAAGCAA AAGCTTCCCA

GTTTCCAGCA GCTGGTTTCA GCCCGAAAAG AACAGAAGCG AGAGGAGCGC

GTTTCCAGCA GCTGGTTTCA GCCCGAAAAG AACAGAAGCG AGAGGAGCGC

GTTTCCAGCA GCTGGTTTCA GCCCGAAAAG AACAGAAGCG AGAGGAGCGC

CGACAGCTGA AACAGCAGCT GGAAGACATG CAGAAGCAGC TGCGCCAG

CGACAGCTGA AACAGCAGCT GGAAGACATG CAGAAGCAGC TGCGCCAG

CGACAGCTGA AACAGCAGCT GGAGGACATG CAGAAACAGC TGCGCCAG

>Ortholog Group 116, Repeat 1

3 102

R00029706 AGGGTACAAA ACACCATTCC CCAC------ ---------- ----------

M00044653 ---------- ---------- ---------- ---------- ----------

H00394875 CGGATGCCAG AGACAGTGCC CCAAGAGGAG ATGCCAGGGC CGCCACTGAA

---------- ---------- ---------- ---------- ----------

---------- ---------- ---------- ---------- ----------

TTCAGAGTCT GGGGAGGAGG CTCCCACAGG CCGGGACAAG AAGCGGAAGC

--

--

CT

>Ortholog Group 117, Repeat 1

3 198

R00030918 TGGCAGCTGC CCCCTCCTCC TCCCCAGAAT GGCTTCATGA ATGGCACCAT

H00353734 TGGCAGCTGC CCCCTCCTGC TACCCAAAAT ACCTTTGGGA ATAGCACTCT

M00112847 TGGCAGCTCC CCCCTCCTAC TCCCCAGAAT GGCTTTGTGA ATAGCACCAT

CCCTGTGGGG GCCGGGGAGC CGCTGCCCCA TAGGATAACT TGCCTGGCAG

TGCCCTGGGG CCTGGGGAAT CTTTGCCCCA CAGGTTAAGC TGTCTGGGGG

CCCTGTGGGG CCTGGGGAGC CACTGCCCCA TAGGATAACC TGTCTGGCGG

AAGCCCAAAT AGCAATGAGC GCTGTGAATA TGGGCCAGCC CCCGCTACCC

AACCCCCACT TGCCATGGGC ACTGTGAGCC TGGGCCAGCT CCCCCTGCCC

AAGCCCAAAT AGCAGTGAGT GCTGTGAATC TGGGCCAGCC CCCCCTACCC

CCGACCCCTC ATGTTTTCAC AGCTGGCACC AACACCGCTA TCCTGCCC

CCCATCCCTC ATGTGTTCTC AGCTGGCACT GGCTCTGCCA TCCTGCCT

CCAACTCCCC ATATTTTCAC AGCTGGCTCC AACACTGCTA TCCTGCCC

>Ortholog Group 118, Repeat 1

3 198

R00006691 CAGCTACAGC AGATCCTACA GCAGTACCAG CAAGTCATCC AGCACTCGCC

M00021670 CAGTTACAGC AGATCCTGCA GCAGTACCAG CAAGTTATCC AGCACTCACC

H00324463 CAGTTACAGC AGATTCTACA ACAGTATCAG CAGATTATAC AGCCCCCACC

ACATATACAG ACCATGTCTC TCGATGTGCA GCTGCGACAC TATGAGATGT

ACACATACAG GTA------- ---------- ---------- ----------

ACATATACAG ACCATGTCTG TAGATATGCA GCTGCGGCAT TATGAGATGT

TTCAACGTCT CTATCAGGAG TGGGAGCGAG AGTTCCAGCT GTGGGAGGAG

---------- ---------- ---------- ---------- ----------

TTCAACATCT TTACCAAGAA TGGGAGCGAG AGTTTCAGCT ATGGGAGGAA

CAGCTCCATT CCTACCCCCA TAAAGATCAG CTTGAGGAGT ATGAGAAG

---------- ---------- ---------- ---------- --------

CAACTCCATT CCTATCCTCA TAAAGATCAG CTTCAGGAGT ATGAGAAG

>Ortholog Group 119, Repeat 1

3 201

R00019968 AGTAGCAGTG GCTCGACACA CTGTGCATTT ACAGCCGAGC AGTACCAGCA

M00028100 AGTAGCAGTG GCTCAGCACA CTGTGCATTC ACAGCCGAAC AGTACCAGCA

H00263062 AGTAGCAGTG GTTCAGCACA CTTTGCATTT ACAGCCGAAC AATACCAGCA

GCACCAGCAG CAACTGGCAC TCATGCAGCA GCAGCAGCTT GCACAAACT-

GCACCAGCAG CAGCTGGCAC TCATGCAGCA GCAGCAGCTT GCGCAGACTC

ACATCAACAG CAACTGGCAC TCATGCAGAA ACAGCAGCTT GCACAAATT-

--GCAAATAG CAGTTCCTCC ACCGCCGCTC CA-------- ----------

AGGCAAATAG TAGTTCCTCT GCCGCCGCGC AA-------- ----------

--GCAAATAG TAATTCCTCC ACCAACACAT CACAGAACCT TGCATCTAAC

---------- ---------- ---------- ---------- ----------

---------- ---------- ---------- ---------- ----------

CAGCAGAAAA GTGGCTTTCG CCTGAATATA CAGGGTTTAG AAAGAACACT

-

-

A

>Ortholog Group 11, Repeat 1

3 201

R00037001 CCCGAGGGCA CTTTCTGGGC CAACAACCAT GGCCGCAACT ACACTGTCCT

H00055335 CCTGAGGGCA CTTTCTGGGC CAACAACCAC GGCCGCAACT ACACAGTCCT

M00122903 CCCGAGGGCA CTTTCTGGGC CAACAACCAT GGCCGCAACT ACACTGTCCT

GCTCCGGATC GCACCCGCTC CCACACCCAC TGATGCTGAA GGGCTGCCCC

GCTCCGGATC GCACCCGCTC CCACACCCAC TGATGCCGAA GGGCTGCCCC

GCTCCGGATC GCACCCGCTC CCACACCCAC TGATGCCGAA GGGCTGCCCC

TGCAGCAGCT GGAGCCACAG CCTGAGTGCC AGGGTCCTGT GGAGGCTGAG

TGCCGCAGCT GGAGCCACAG CCCGAGTGCC AGGGTCCCGT GGAGGCTGAG

TGCAGCAGCT GGAGCCACAG CCTGAGTGCC AGGGTCCTGT GGAGGCTGAG

GCCAGGCAGC TGAAGAGCTG CATGAAGCCG GTGAGGCGCA GGACAAGCCT

GCCAGGCAGC TGAAGAGCTG CATGAAGCCG GTGAGGCGCA GG---CCTGC

GCCAGGCAGC TGAAGAGCTG CATGAAGCCG GTGAGGCGCA GG---CCTTT

C

C

T

>Ortholog Group 120, Repeat 1

3 198

M00036507 AAATCTGAAG AGGTATATTG TTTGCAGAAA GAGCTAAAGA TAAAGACTCA

H00359299 AAATCTGAAG AGGTATATTG TTTACAGAAA GAGCTAAAGA TAAAAAATCA

R00035046 AAATCTGAAG AGGTATATTG TTTGCAGAAA GAGCTAAAGA TAAAGACTCA

TAATCTTGAA GAGACGAGTG AACAAAATGC CATCCTACAG CACACTCTTA

CAGTCTTCAA GAGACTTCTG AGCAAAACGT TATTCTACAG CATACTCTTA

TAACCTTGAA GAGACGAGTG AACAAAATGC TATTCTGCAG CATACGCTTA

TGTTACAGCA AGAGACCATG AGAAATGGAG AGCTAGAAGA TACTCAGAGT

TGTTACAACA AGAGACAATT AGAAATGGAG AGCTAGAAGA TACTCAAACT

TGTTACAGCA GGAGACCCTC AGAAGTGGAG AGCTAGAAGA TATCCAGAGT

AAACTTGAAA AACAGGTATC AAAGCAAGAA CAAGAGCTTC AGAAACAA

AAACTTGAAA AACAGGTGTC AAAACTGGAA CAAGAACTTC AAAAACAA

AAACTGGAAA AACAGGTATC AAAGCAAGAA CAGGAGCTGC AGAAACAA

>Ortholog Group 121, Repeat 1

3 198

R00017848 GTTGCCATCT TCACATTGAT GGTGGAGATA AACAAGAAAG GGAAAGCCCT

H00340507 GTTGCTATAT TTACACTGAT GGTAGAAATA AATAAAAAAG GAAAAGCTCT

M00031859 GTTGCCATCT TCACATTGAT GGTGGAGATA AACAAAAAAG GGAAAGCTCT

GCTGCACCAG CTTGAGAGTC TTGCAAAAGA CCATCGAATG AAACTTATGG

ACTGCATCAG TTAGAGAGCC TTGCAAAGGA CCATCGCATG AAACTTATGG

GCTGCACCAG CTTGAGAGTC TTGCAAAGGA CCATCGAATG AAACTCATGG

AAGTGGCTGG GCTTTCTAAG CAGTTAGAGC ATGTCATGCA TTTTTCTAAA

AAGTGGCTGG ACTCTCTAAA CAATTGGAGC ATGTCATGCA TTTTTCTAAA

AAGTGGCTGG GCTTTCTAAG CAGTTAGAGC ACGTCATGCA TTTTTCTAAA

TGGGCTGTTT CCAGTGGCAG CAGCACAGCC TTGCTGTACA GCAAGCGG

TGGGCAGTTT CCAGTGGCAG CAGTACAGCA TTACTTTATA GCAAACGA

TGGGCTGTTT CCAGTGGCAG CAGCACAGCC TTGCTGTACA GCAAGCGG

>Ortholog Group 122, Repeat 1

3 204

M00021606 TGTGAAAATA GTCCACAGAC ATCAAGTCCA GATCTCTCTT CAGAAGAGCT

H00369996 TCGCAAGATC TTCCAAAGAC ATCATGTGTA ACTCCTGCTT CAGAACAGCC

R00007505 TGTGAAGATA GTCCGCAGAC ATCAAGCACA GATCTTTCTT CAGAAGAGCT

GCGGAGGAGA CGAGAAGCCT ACTTTGAAAA G--------- ---CAACAGG

GAAGAAAATA AAAGAAGACT ATTTTGAAAA GCATCAGCAG GAACAGAAGT

GCGGAAGAGG AGAGAAGCCT ACTTTGAAAA G--------- ---CAACAGG

TAGACCGACC TGGACCCCTT TCATATCCAC GTGAAAGACC GACCACAAGT

CAGATCTGCC GGGCCACAGT TCATACCTAC ACGAAAGGCC AACAACAAGT

CAGACCGACC TGGATACCTT TCATACCCAT GTGAAAGACC CACCACAAGT

TCAGGAGGAC GTAGGAGCGA CCAAGGAGGC GACGCTGTGA GTGAAGAGGA

TCGAGAGCAA TTGAGAGTGA TCTCAGTGAT GACATC---- --AGTGAAGG

TCAGGAGGAC TCCGGAGCAA CCAAGCAGGC AATGCTATGA GTGAAGAGGA

CATG

CACA

CGTG

>Ortholog Group 123, Repeat 1

3 267

H00361066 GGAGATTATA ATACTTCTTT ACCCAGACCT GCACTGGGTG GCTCTATTCC

R00007768 GGAGACTACA GCGCAGCTTT ACCAAGACCC GCCTTGGGGA GCTCCGGGCC

M00085416 GCCGATTACA GTGCCACTTT ACCCAGACCT GCCATGGGGG GCTCTGTGCC

CACATTGCCT CTTCGGTCTA ATAGCATACC AGGTGCGAGA CCAGTATTG-

TACCTTGCCA CTTCGTTCTA ATAGACTGCC AGGCGCAAGA CCAACGTTGC

TACCTTGCCA CTTCGTTCTA ATCGACTGCC AGGTGCAAGA CCATCGTTGC

---------- ---------- ---------- ---------- -------ATG

AGCCTCAGCC GCAGCCGCAG CAGCAACAGC AGCAGCAACA GCAGCAAATG

AGCAACAGCA GCAGCAACAG CAGCAACAGC AACAACAACA GCAGCAACAG

---------- ---------- -CTTCAAATG AGGCCTGGTG AAATCCCCAT

CTTCAAATGA TCTTGATTGA CCTGGAGCTC AGAGCTGGTG AGGTTCCCAT

CAGCAGCAAC AGCAGCAGAT GCTTCAAATG AGAACTGGTG AGATTCCCAT

GGGAATGGGG GCTAATCCCT ATGGCCAAGC AGCAGCATCT AACCAACTGG

GGGAATGGGC GTCAGTCCCT ATAGCCCAGC AGTGCCATCT AACCAACCCG

GGGAATGGGA GTCAATCCCT ATAGCCCAGC AGTGCCGTCT AACCAACCAG

GTTCCTGGCC CGATGGC

GATCGTGGCC AGAGGGC

GTTCCTGGCC AGAGGGC

>Ortholog Group 123, Repeat 2

3 198

H00361066 AGCTCCCAGC AGGGTTTTCT TAATGCTCAA ATGGTCGCCC AACGCAGCAG

R00007768 ------TCCC AGGCTTTCTT TAATGCCCAA ATGGCAGCCC AGCAGAAACG

M00085416 ------CAGC AGGCTTTCTT TAATGCCCAA ATGGCTGCCC AGCAGAAACG

AGAGCTGCTA AGTCATCACT TCCGACAACA GAGGGTGGCT ATGATGATGA

AGAGCTGATG AACCATCACC TGCAGCAGCA GAGGATGGCG ATGATGATGC

AGAGCTGATG AGCCATCACC TGCAGCAGCA GAGGATGGCG ATGATGATGC

CCCAGGCCTT CAGCCCACCT CCTAATGTGA CTGCTTCCCC CAGCATGGAT

CTCAGGCCTT CAGCCCACCT CCCAACGTCA CCGCTTCCCC CAGCATGGAC

CTCAGGCCTT CAGCCCACCT CCCAACGTCA CCGCCTCCCC CAGCATGGAC

GGGCTTTTGG CAGGACCCAC AATGCCACAA GCTCCTCCGC AACAGTTT

GGGGTCTTGG CAGGCTCAGC AATGCCACAA GCCCCTCCAC AACAGTTT

GGGGTTTTGG CAGGTTCAGC AATGCCGCAA GCCCCTCCAC AACAGTTT

>Ortholog Group 124, Repeat 1

3 198

H00362238 GAAAATGACA AGATGAGACT TGAGAAAGAT TTATCATTCA AAGACACTCA

R00006415 GAAAATGACA AGATAAGACT TGAAAAGGAC TTGGCATTCA AGGAAAATCA

M00047655 GAAAATGACA AGATAAGACT TGAAAAGGAC TTGGCATTCA AAGAAAATCA

ATTAAAAGAG TACGAAGAAC TCTTGGCATC AGTGAGAGCA AATAATCACG

AATAAAAGAG TATGAAGAAC TCTTGGCATC AGTGAGAGCA AATAATCGCG

AATGAAAGAG TATGAAGAAC TCTTGGCATC AGTGAGAGCA AATAATCGCG

GACTTCAAGA CTCAAGTTCA AAATGCCAGG CATTGGAAGA AAACAATCTC

GACTTCAAGA CTCAAGCTCA AAATGTCAGA CATTGGAAGA AAATAACCTT

GACTTCAAGA CTCAAGCGCA AAATGTCAGT CATTGGAAGA AAATAACCTT

TCTCTTCGAC ATACACTATC AGACATGGAA TACAGACTAA AAGAACTG

TCTCTTCGAC ATACACTGTC GGACATGGAA TACAGACTAA AAGAACTT

TCTCTTCGAC ATACACTGTC AGACTTGGAA TACAGACTGA AAGAACTT

>Ortholog Group 125, Repeat 1

3 150

H00362013 CTGGCTCAGG TGCCAGTGCA GGGAGGACAG CCACTGCAGG CCCCACAGAT

M00077103 CTGGCCCAGA TGTCCATGCA AGGAGGACAG CCACTGCAAG CCCCTCAGGT

R00061868 CTGGCCCAGA TGTCCGTGCA AGGCGGACAG CCACTGCAAG CCCCTCAGGT

GCTGTCACAG CACATGCAAC AGATGCAGCA GCACCAGTAT TAC------C

GCTGTCCGGC CATATGCAAC AATTGCAGCA GCACCAGTAT TACCCACAGC

GCTGTCCAGC CATATGCAGC CATTGCAGCA GCACCAGTAT TACCCACAGC

CACCGGCCGG GCAACAGCGT ATCTCCATGC AAGAAATA-- ----CAGACG

CGCCGGCCGG ACTGCAGCGG ATCTCTGTGC AGGAGATGCA GCAGCAGCAG

CGCCGGCCGG ACTGCAGCGG CTCTCCGTGC AGGAGATGCA GCAGCAGCAG

>Ortholog Group 125, Repeat 2

3 84

H00362013 CCGCAACAAA TTCGCCCATC ACAGCCACAG CCGCCGCCAC CGCAGCAGCT

M00077103 CCGCAGCAAA TTCGCCCCTC A--------- ---CCACCT- --CAGCAGCT

R00061868 ---CAGCAGA TTCGCCCCTC A--------- ---CCGCCT- --CAGCAGCT

ACAGCTGCAG CAGCGGCAGG GTTCAATGCA GATA

CCAGCTGCAG CAGCGGCAGA GTTCACTGCA GATA

CCAACTGCAG CAACGGCAGA GTTCACTGCA GATA

>Ortholog Group 125, Repeat 3

3 144

H00362013 CCTCAGTATT ATCAGCCCCA ACCCATGATG CAGCACTTGC AAGAGATGCA

M00077103 CCTCAGTATT ATCAGCCCCA ACCCATGATG CAACACTTGC AAGAGATGCA

R00061868 CCTCAGTATT ATCAGCCCCA ACCCATGATG CAACACTTGC AAGAGATGCA

CCTGCAGCCT CCTTCTTATC ACAGGGACCC TCACCAGTAT ACCCCAGAGC

CCTGCAGCCA CCCTCATACC ACAGGGACCC TCATCAGTAT ACCCCGGAGC

CCTGCAGCCC CCTTCGTACC ACAGGGACCC TCACCAGTAT ACCCCGGAGC

AGGCACACAC TGTCCAGCTG ATTCCCCTGG GCTCCATGTC CCAG

AGGCACACGC TGTCCAGCTG ATCCAGCTGG GCTCTATGCC CCAG

AGGCACATGC GGTCCAGCTG ATCCAGCTGG GCTCCATGCC CCAG

>Ortholog Group 126, Repeat 1

3 102

H00244769 GGGGCCACCA CTCCATCCCA GCGCTCCCAG CTGGAGGCCT ATTCCACTCT

R00023140 GGGGCCACCA CTCCATCACA GCGCTCCCAG CTGGAGGCAT ATTCCACCCT

M00089217 GGGGCCACCA CTCCATCACA GCGCTCCCAG CTGGAGGCTT ATTCCACCCT

GCTGGCCAAC ATGGGCAGTC TGAGCCAGAC GCCGGGACAC AAGGCTGAGC

GCTGGCCAAC ATGGGCAGTC TGAGCCAGGC ACCAGGACAC AAGGTTGAG-

GCTGGCCAAC ATGGGCAGTC TGAGCCAGGC ACCAGGACAT AAGGTTGAG-

AT

--

--

>Ortholog Group 126, Repeat 2

3 105

H00244769 CATCACCTCA GCAGGGCTCC GGGGCTCATC ACCCCGGGGT CC---CCCCC

R00023140 ---CACCTCG GCAGGGCTGC GGGATTAGTC AACCCGGGGT CC---CCTCC

M00089217 ---CACCTCA GCAGGGCTGC AGGATTAGTC AACCCGGGGT CCCCTCCTCC

ACCAGCCCAG CAGAACCAGT ACGTCCACAT TTCCAGTTCT CCGCAGAACA

ACCTACCCAG CAGAACCAGT ACATTCACAT TTCCAGCTCT CCACAGAGCT

ACCCACCCAG CAGAACCAGT ACATCCATAT TTCCAGCTCT CCACAGAGCT

CCGGC

CCGGG

CCGGG

>Ortholog Group 127, Repeat 1

3 222

H00352514 AACTTCTTTT GGGATCCGAG CACCAGCCGG CGCTTCAGCC CCCCCTCCAG

R00057733 ---------- --GATCCGAG CACCAGCCGG CGCTTCAGCC CCCCCTCCAG

M00123743 AGCTTCTTTT GGGATCCGAG CACCAGCCGG CGCTTCAGCC CCCCCTCCAG

CAGCCTGCAG CCCGGCAAAA TGAGCGACGT GAGCCCGGTG GTGGCTGCG-

CAGCCTGCAG CCCGGCAAGA TGAGCGACGT GAGCCCGGTA GTGGCTGCTC

CAGCCTGCAG CCCGGCAAGA TGAGCGACGT GAGCCCGGTG GTGGCTGCG-

--GAG----- ---------- ------GCGG CGGCGGCGGC TGCGGCGGCG

AGCAGCAGCA GCAGCAGCAG CAGGAGGCGG CCGCAGCAGC AGCAGCGGCA

--CAGCAGCA GCAGCAGCAG GAGGCGGCCG CAGCAGCAGC GGCGGCAGCG

GCGGCGGCTG CGGCGGCGGC AGCTGCAGTG CCCCGGTTGC GGCCGCCCCA

GCGGCGGCGG CAGCAGCGGC GGCAGCCGTG CCCCGGTTGA GGCCGCCGCA

GCGGCGGCAG CAGCGGCGGC GGCCGCAGTG CCCCGATTGA GGCCGCCGCA

CGACAACCGC ACCATGGTGG AG

CGACAACCGC ACCATGGTGG AG

CGACAACCGC ACCATGGTGG AG

>Ortholog Group 128, Repeat 1

3 198

H00313500 TGGATTGGCA ATCAGAGAAG AGTGACAGTG ACAGAAGTGC TCAGAGAGAC

M00097961 AGCTTGAGTA GTCAGCAAAG AATGACGGGA GCAAAGGTGT TCCAAGAGTC

R00028383 AGCTCAGGCA GTCAGCAAAG AATGACAGGA ACAAAAGTGT CCCAGGAGTC

AGCAAGACCT CAGTCCTCAG CCTTACACCC CCTACTCACC TTTGAGAGCG

GTCAGGCCCC CAGCCCTCAG CCGCACACTC TGGATATATT TTTGAGAGCA

ATCAGGACGT CAGTCCTCGG CCGCACACTC TGGATTTATC TTTGAGAGCA

TTGGTGGCTT TGAGGGGCCT GAACAAGACG AATTTGATAA AGTCCTGGCA

TTGGTGACTT TGAAGCACCT GATCAAGATG ACCTTGACAA GGCCCTGGCA

TTGGTGACTT CGAAACACTT GATCAAGATG AGTTTGACAA GGCCCTGGCG

AGCATGGAGT TGGAGGAGCC TGGCATGGAG CTGGAATGTG GAGTCAGC

AGCATGGAGT TCGAGGGAGC TGGCTTGGAG CTAGAA---- --GCCGAC

AGCATGGAGT TCGAGGGAGC TGGCTTGGAG CCAGAA---- --GTTAAC

>Ortholog Group 129, Repeat 1

3 198

H00245564 GACAGAGCAT GCCACACAAG CCAGCTCACC CCAGGGACAC CTCCACCCTC

R00027579 GACAGAGCTA GCCACACCAG TAAGCTCAAC CCAGGGACAC CTTTGCCCTC

M00103118 GACAGAGCTA GCCACACCAG TAAGCTCAAC CCAGGGACAC CTCTGCCCTC

TGCCCTTCAT GCATGTACCA CTGGGGAAGA AATCTTGGCT CAGTATTTAC

CACTCTCCAT GCGTGTGCCT CTGGAGAAGA AGTCTTGGCC CAGTACTTAC

TGCTCTCCAC GCATGCGCCT CTGGAGAAGA AGTCTTGGCC CAGTACTTAC

CTGGAGTCAT GAGTTCTTCC CATCTGCTGC TGACTCCCTG CAGGGTGGCT

CTAGGGTCTT GAGTTCGTCA TATCTGCTGC TGACACCCTG CAAAATGGCT

CTAGAGTCTT GAGTTCGTCA CATCTGCTGC TGACTCCCTG CAAAGTGGCT

CCTCCTTACC CCCACCTCTT CTCAAGCTGC AGTCCACCGG GTATGGTT

CCTCCGTACC CACACTTCTT CTCAAGCTTC AGTCAGAAAG GGTTGGCC

CCTCCATACC CACACTTCTT CTCAAGCTTC AGTCAGAAAG GGCTGGCC

>Ortholog Group 12, Repeat 1

3 198

M00047962 GCACCGCTGG AGAGCAGCTC CGGGGCGCGG GGATCCTTTA ACAAGTTCCA

R00000009 GCACCGCTGG AAAGCAACGC TGGGGCGCGG GGATCCTTTA ACAAGTTCCA

H00332744 ACACCCCTGG AGAGTGGAGC TGGGGCGCGG GGCTCCTTTA ACAAGTTCCA

GCCGCAGCCG CCCACCCCAC AGCCGCCGCC CGCTCCA--- ----------

GCCGCAGCCG CCCACCCCGC AGCCGCCGCC CGCTCCG--- ----------

GCCCCAGCCG TCGACCCCGC AGCCCCAGCC GCCGCCGCAG CCGCAGCCGC

-----CCCGC GCCGCCTGCG ------CATC TCTACTTGCA GAGGGGCGCC

-----CCCGC GCCGCCTGCG ------CATC TCTACTTGCA GAGGGGCGCC

CGCAGCCCCA GCCGCCCGCG CAACCGCATC TTTACTTGCA GCGAGGCGCC

TGCAAAACGC CCCCGGACGG TAGCCTCAAG CTCCAGGAGG GCAGCGGC

TGCAAAACGC CCCCGGACGG CAGCCTCAAG CTCCAGGAGG GCAGCGGC

TGCAAGACGC CCCCGGACGG CAGCCTCAAA CTCCAGGAAG GCAGCAGC

>Ortholog Group 130, Repeat 1

3 198

M00045918 GCTTCTCCGT GTCAGGAGCA CAAGCGAGGT GGCCTAAGCG AGCTAAAGGG

R00017742 GCTTCTCCGT GTCAGGAGCA CAAGCGAGGT GGCCTGAGCG AGCTGAAGGG

H00315955 GCCTCCCCGT GCCAGGAGCA CAAGCGAGGG GGCCTGGGAG AGCTGAAGGG

AGCACCTGCC TCTGCGCTGA GTCCTCCCGA GCCGGCGCCC TCGCCTGGGG

AACACCTGCC TCTGCGCTGA GTCCTCCGGA GCCGGCGCCC TCGCCTGGGG

GACGCCGGCT GCGGCGCTGA GCCCCCCAGA GCCGGCGCCC TCTCCCGGGG

CTGCAGCCCA CCTGCTGGGC CCACCTCACC ACCCAGGCCT GCCACCAGAG

CTGCAGCCCA CCTGCTGGTC CCACCTCACC ATCCTGGCCT GCCACCAGAG

CCGCGGCCCA CCTGCTGGGC CCGCCCCACC ACCCGGGCCT GCCGCCTGAG

GCCCACCTGA AGCCCGAGCA CCATTACGCC TTCAACCACC CCTTCTCT

GCCCACCTGA AGCCCGAGCA CCATTACGCC TTCAACCACC CCTTCTCT

GCCCACCTGA AGCCGGAACA CCACTACGCC TTCAACCACC CGTTCTCC

>Ortholog Group 131, Repeat 1

3 198

R00009140 GAGGCCTACC AGATCCCTGC CTCGTACCAG CCTGATGAGG AGGAGCGAGC

H00348632 GAGGCCTACC AGGTCCCCGC CTCATACCAG CCCGACGAGG AGGAGCGAGC

M00105473 GAGACCTACC AGATACCTGC TTCATACCAG CCTGACGAGG AAGAGCGAGC

ACGCCTGGCT GGTGAGGAGG AGGCGCTGCG CCAGTATGAG CAGCGGAAAG

GCGCCTGGCG GGCGAGGAGG AGGCGCTGCG TCAGTACCAG CAGCGGAAGG

ACGCCTGGCC GGCGAGGAGG AGGCGCTGCG CCAGTACCAG CAGCGGAAAG

AGGGGAACTA CCTGCAGCAC GTGCAGCTGG AGCAGAGGAG CCTGGTGCTG

AGGGGAACTA CCTGCAGCAC GTCCAGCTGG ACCAGAGGAG CCTGGTGCTG

AGGGGAACTA CCTGCAGCAC GTGCAGCTGG AGCAGAGGAG CCTGGTGCTG

AACACCGAAC CTGCTGAGTG CCCTGTGTGC TACTCAGTGC TGGCACCC

AACACGGAGC CCGCCGAGTG CCCCGTGTGC TACTCGGTGC TGGCGCCC

AACACCGAAC CTACTGAGTG CCCTGTGTGC TACTCAGTGC TGGCACCC

>Ortholog Group 132, Repeat 1

3 198

R00007888 GAGGCTCTGC AGAAGCGCCT GGAGGAGGTC ACCCGGGAGC TATGCCGGGC

H00246043 GAGGCCCTGC AGAAGCGCCT GGACGAGGTC AGCCGGGAGC TGTGCCACAC

M00016072 GAGGCTCTGC AGAAGCGCTT GGAGGAGGTC ACCCGGGAAC TCTGCCGGGC

ACAGACGAGC CATGCCAACC TCCGAGCGGA TGCTGAGAAG GCTCAGGAAG

GCAGAGCAGC CACGCCAGCC TCCGGGCGGA TGCCGAGAAG GCCCAGGAGA

ACAAACGAGC CATGCCAACC TCCGAGCGGA TGCTGAAAAG GCTCAGGAAG

TGGCAGAGTT GCACAGCAAA TTACAGTCCT CGGAGGTGGA GGTAAAAAGC

TGGCCGAGCT GCACAGCAAG TTACAGTCCT CCGAGGCGGA GGTGCGCAGC

TGGCAGAGTT GCACAGCAAA TTACAGTCAT CTGAGGTGGA GGTAAAAAGC

AAGTGTGAGG AGCTGAGTGA TCTCCATGGG CAGCTCAAGG AGGCCAGG

AAATGCGAGG AGCTGAGTGG CCTCCACGGG CAGCTCCAGG AGGCCAGG

AAGTGTGAAG AGCTGAGTAG TCTCCATGGG CAGCTCAAGG AGGCCAGG

>Ortholog Group 133, Repeat 1

3 108

M00116189 GGTGACCACT CTGCTCACCT GTCAAGGCAT CAGCAGCATC TTCTTTCGAG

R00040549 GGAGACCACT CTGCTCACCT GCCGAGGCAT CAGCAGCATC TTCTTCCGAA

H00429013 GGAGACCATT CAGCTCACCT GCCTAGGCCG CAGCAGCATT TCCTTCCTAA

TCAGAGTCAT CAGGGGGACC ACTACCGTCA CGCCCAGGCC AGCCTGACT-

TCAGAGTCAT CAGGGGGACC ACTACCGGCA CCCCCAGCCC AGCCTGACT-

TCAGGCTCAC CAGGGGGATC ATTACCGTCT CTCCCAACCT GGCCTGAGTC

--------

--------

ACCATCAT

>Ortholog Group 133, Repeat 2

3 108

M00116189 ---------G CGCAGCAGCA GCCAGGAGAA GCCTACTCAG CTATGCCTCG

R00040549 ---------G CCCAGCAGCA ACCAGGAGAA GCCTACTCAG CTATGCCTCG

H00429013 CATCACCACC CACAGCAGCA GCCAGGAGAA GCCTATTCAG CTATGCCTCG

GGCTCAG--- CAGTCTGCTT CTTATCAGCC CATGCCAGCT GACCCTTTTG

GGCTCAG--- CAGGCAGCTG CTTATCAGCC CATGCCAGCT GACCCTTTTG

GGCTCAGCCA TCCTCTGCTT CTTATCAGCC AGTGCCAGCA GACCCTTTTG

CTATGGTC

CCATCGTC

CCATTGTT

>Ortholog Group 134, Repeat 1

3 219

H00380019 GCAGCAGCGG CAGCAGTG-- ---------- GCCCAGGCCC AGGCTCAAGC

R00052839 GCAGCGGCAG CGGCAGTG-- ---------- GCCCAGGCTC AGGCCCAGGC

M00108046 GCAGCAGCAG CAGCGGTGGC CCAGGCCCAG GCCCAGGCTC AGGCCCAAGC

TCAAGCCCAG GCTCAGGCTC AGGCTCAAGC CCAGGCCCAG GCCCAGGCCT

TCAGGCTCAG GCCCAGGCCC AGGCTCAGGC CCAGGCCCAG GCCCAGGCTC

TCAAGCCCAG GCCCAAGCTC AAGCCCAGGC CCAAGCTCAA GCCCAGGCCC

CCCAGGCATC ACCA------ ---CCACCAC ACTTCCAGTC TCCTGGGGCA

AGGCTCAGGC CCCACCTCCA CAACCACCAC ACTTCCAGTC TCCTGGGGCA

AGGCCCAGGC CCCACCGCCA CAACCACCAC ACTTCCAGTC CCCTGGGGCA

GCCCCCCAGG GTGGGGGTGG TGGGGACAGC AATCCCAACC CTCCACCCCA

GCTCCCCAGG GAGGGGGTGG TGGGGACAGC AACCAGAACC CTCCACCCCA

GCTCCCCAGG GAGGGGGTGG TGGGGACAGC AACCCGAACC CTCCACCCCA

GTGTTCCTTT GACCTGACC

GTGTTCCTTT GACCTGACC

GTGTTCCTTT GACTTGACC

>Ortholog Group 135, Repeat 1

3 198

M00111134 ATGAGCACTT TAAGCAGCCA ATTAGATGCT GGCAGCAGGG ATGGGAGATC

H00386200 ATGAGCACTC TAAGCAGCCA ATTAGATGCT GGCAGCAGAG ATGGAAGATC

R00007759 ---------- ---------- ---------- ---------- ----------

AAGTGGTGAC ACCAGCTCTG AAGTAAGCAC AGTGGAGCTG CTGCATCTGG

AAGTGGTGAC ACCAGCTCTG AAGTAAGCAC AGTAGAACTG CTGCATCTGG

---------- ---------- ---------- ---------- ----------

CTCTCCAGGC AGCAAGACAA CTTTTGTTAC AGCAGCAAAC CAGTGGATTG

CTCTCCAGGC AGCAAGACAA CTTCTTTTAC AGCAGCAAAC AAGTGGATTG

---------- ---------- ---------- ---------- ----------

AAATCTCCTA AGAGCAGTGA GAAGCAGAGG CCACTGCAGG AATTGCTC

AAATCTCCTA AGAGCAGTGA TAAACAGAGA CCACTGCAGG AATTGCTT

---------- ---------- ---------- CCATGCAAG- --------

>Ortholog Group 135, Repeat 2

3 105

M00111134 TCGGTGGCCA TGATGACTCC CCAAGTGATC ACCCCTCAGC AGATGCAGCA

H00386200 TCAGTGGCCA TGATGACTCC CCAGGTGATC ACCCCTCAGC AAATGCAGCA

R00007759 TCGGTGGCCA TGATGACTCC CCAAGTGATC ACCCCTCAGC AGATGCAGCA

GATTCTTCAG CAGCAGGTCC TGTCTCCTCA GCAGCTCCAG GCCCTCCTCG

GATCCTTCAG CAACAAGTCC TGTCTCCTCA GCAGCTACAA GCCCTTCTCG

GATCCTTCAG CAGCAGGTCC TGTCTCCTCA GCAGCTCCAG GCCCTCCTCG

CTGTG

CTGTC

CTGTG

>Ortholog Group 135, Repeat 3

3 30

M00111134 ATGTTGCTAC AAGAGTTTTA CAAGAAACAG

H00386200 ATGCTGCTAC AAGAGTTTTA CAAGAAACAG

R00007759 ATGCTGCTAC AAGAGTTTTA CAAGAAACAG

>Ortholog Group 135, Repeat 4

3 36

M00111134 GAACAGTTAC ATCTTCAGCT TTTGCATCCT GGAAAG

H00386200 GAGCAGTTAC ATCTTCAGCT TTTGCATCCT GGAAAG

R00007759 GAGCAGTTAC ATCTTCAGCT TTTGCATCCT GGAAAG

>Ortholog Group 135, Repeat 5

3 39

M00111134 CAAGCAAAAG AGTTGGCGGC GCAGCAGCTC GTCTTCCAG

H00386200 CAAGCGAAAG AGTTGGCAGC CCAGCAGCTT GTCTTCCAG

R00007759 CAAGCAAAAG AGTTGGCCGC TCAACAGCTT GTCTTCCAG

>Ortholog Group 135, Repeat 6

3 126

M00111134 CAGCAGCTTC TCCAAATGCA GCAGCTACAT CTGCTCAGCC TTCAGCGCCA

H00386200 CAGCAGCTTC TCCAGATGCA ACAACTCCAT CTGCTCAGCC TTCAGCGTCA

R00007759 CAACAGCTTC TCCAGATGCA GCAACTACAT CTGCTCAGCC TTCAGTGTCA

GGGCCTCATC TCCATCCCAC CCGGCCAAGC AGCCCTTCCT GTCCAGTCGC

GGGACTCATC TCCATTCCAC CTGGCCAGGC AGCACTTCCT GTCCAATCGC

GGGCCTCATC TCCATACCAC CCGGCCAAGC AGCACTTCCC GTCCAGTCGC

TGCCTCAAGC TGGCTTAAGT CCTGCT

TGCCTCAAGC TGGCTTAAGT CCTGCT

TGCCTCAAGC TGGCTTAAGT CCTGCC

>Ortholog Group 136, Repeat 1

3 198

H00249499 GCGACTGGGA CGGGCGGCTC GTCGGAGCCC TCAGCTTGCA GCGACCACCC

R00059218 AAACCACAGT GC------AG TTCTGAACCA CTAAATTGGG AGATCCACNC

M00058490 GGGCCAGGGG CGGTAGGCTC GTCCGAGCCC TCAGCTTGCA GCGATCACCC

GATCCCAGGC TGTTCGCTGA AGGAGGAGGA GAAGCAGCAT TCGCAGCCGC

GAGCCCGGGC TGCCCGCTGA AGGAGGAGGA GAAGCAGCCG CCGCAGCCGC

GAGCCCGGGC TGCTCGCTGA AGGAGGAGGA GAAGCAGCCG CCGCAGCCGC

TTGACCCAAA CAACCCCGCC GCGAACTGGA TCCACGCTCG CTCCACCCGG

TTGACCCAAA CAACCCTGCA GCGAACTGGA TCCACGCTCG CTCCACCCGG

TTGACCCAAA CAACCCTGCA GCGAACTGGA TCCACGCTCG CTCCACCCGG

AAAAAGCGCT GTCCCTACAC CAAATACCAG ACGCTTGAGC TGGAGAAA

AAAAAGCGCT GTCCCTACAC CAAATACCAG ACGCTAGAGC TGGAGAAG

AAAAAGCGCT GTCCCTACAC CAAATACCAG ACGCTAGAGC TGGAGAAG

>Ortholog Group 137, Repeat 1

3 276

H00349856 GTGGCACAGA GCAACAGCAG T--------- ---------- -----GAGGA

R00013663 GTGGCTCAGA GCAACAACAG CAGCAGCAGC AGCAGTAGCA GCATCGAGGA

M00064892 ---------- ---------- ---------- ---------- ----------

AGAGGCCAGA GAGGCTGGGT CCCCTGCACA GGAGTTC--- AAGTATCAGA

GGAAGTCCGG GAGGCTGGGT CCCCTGCACA GGAGTTCAGC AAGTACCAGA

---------- ---------- ---------- ---------- ----------

AGTCCCTTCC TCCCCGATTC CAGCGC---- ---------- ----------

AGTCCCTTCC TCCCCGTTTC CAGCGCCAGC AACAGCAGCA GCAGCAGCAG

---------- ---------- ---------- ---------- ----------

---------- ---------- -------GAG CAGCTGTACA AGATGCAGCA

CAACAACAAC AGCAGCAGCA GCAGCAAGAG CAGCTGTACA AGATGCAACA

---------- ---------- ---------- ---------- --ATGCAGCA

CTGGCAGCCG GTGTACCCCC CGCCGTCCCA CCCCCAGCGC ACCTTTTACC

CTGGCAGCCG GTGTACCCCC CACCTTCTCA CCCCCAGCGT ACCTTCTACC

CTGGCAGCCG GTCTACCCCC CACCTTCTCA CCCCCAGCGC ACCTTCTACC

CACACCACCC CCAGATGTTG GGCTTC

CACACCACCC CCAGATGCTG GGCTTT

CACACCACCC CCAGATGCTG GGCTTT

>Ortholog Group 138, Repeat 1

3 198

M00034700 TCCCGAGAGC ACGTGGCTGT GGTTGTGGGT TCAGGCATTG GCTATGGAGC

H00377144 CCACAAGGGC ACGTGGCTGT GGCCGTGGGC TCAGGTGGCA GCTATGGAGC

R00012138 TCACAAGAGC ATGTGGCTGT GGTTGTGGGT TCAGGCATTG GCTATGGAGC

TGAAGTAGGG ---GAGGAAG AGGATGACAA GACATCACTT CTACAGGAAC

CGAGGATGAG GTGGAGGAGG AGAGTGACAA GGCCGCGCTC CTGCAGGAGC

CGAA---GGA ---GAGGAAG AGGATGACAA AACATCACTT CTACAGGATC

CCAGATTCTG GACATTTGAC TACTATCAGA GCTTTTTTGA TGTGGATACC

CGGGATTCTG GACCTTCAGC TACTATCAGA GCTTCTTTGA CGTGGACACC

CAAGATTCTG GACGTTTGAC TACTATCAGA GCTTTTTTGA TGTAGACACC

TCCCAGGTCT TGGACAGGAT CAAAGGCTCC CTGCTGCCCC ATCCTGGC

TCACAGGTCC TGGACCGGAT CAAAGGCTCA CTGCTGCCCC GGCCTGGC

TCCCAGGTCT TGGACAGGAT CAAAGGTTCC CTGCTGCCCC ATCCTGGC

>Ortholog Group 139, Repeat 1

3 198

M00028984 GAAGGACGGA TCCAGAGCAT GCGCCTTCTG GACCGTCTGA ACGTCTCAGG

R00018512 GAAGGACGAA TCCAGAGCAT GCGTCTTCTG GACCGTCTGA ACGTCTCAGG

H00364603 GAAAGCCGAA TTCAGAACAT CCACTTTGGG GACAGACTGA ATGCCTCAGC

GACAGTGGCT CCAGGGATGG TGGGCTGGCT GATTGGTGGC ATGAACTTCG

GACAGTGGCC CCAGGGATGG TGAGCTGGCT TATTAGTGGC ATGAACTTCG

ACAAGTGGCC CCAGGGCTGG TGGGCTGGCT AATCAGCGGC AGGAAACACG

AAATCAGCAT CAACATTACC AATGTGCAGC TGGACTGTGG TGGGATCCAG

AAATCAGTAT CAACATTACC AATGTGCAGC TGGACTGTGG CGGGATCCAG

AGAGCAGCAT CAACATCACC AACATTCAGC TGGACTGTGG TGGGATCCAG

ATGGCTTTCC CCAAAGAGTG GTTCTCTGCA AACATCACAC TTGAATTT

ATGGCTTTCC CCAAAGAGTG GTTCTCCGCA AACATCACAC TTGAATTT

ATATCATTCC ATAAGGAGTG GTTCTCGGCA AATATCTCAC TTGAATTT

>Ortholog Group 13, Repeat 1

3 198

R00055547 ---------- ---------- ---------- ---------- ----------

M00119120 GTGGAGCAGC TGGCTCACAG GAAGTGTTTC CAGCAAGCCA CAGAACACAG

H00350719 GTGGAACAGC AGATTCAAAA GAAGTATTCT CAGCAGGTAG TGGAATATGA

---------- ---------- ---------- ---------- ----------

CGAACTCACC TGCCTCCTGG AGAAGCTCCA GGACCTTAAG GTTTCTCTGC

TGAATTTACA ACCCTCATGA ATAAGGTACA GGACACTGAG ATTTCTCTGC

---------- ---------- ---------- ---------- ----------

GCCTAACGCT AAGTCTGAAC TCTCCAGGAC AACAGGCGGC GATCGTGGAC

ATCTACAGTT AAGGCTGAAG TCTCCAGAAG AACGGGCAGG GAACCAAAGC

---------- ---------- ---------- ---------- --------

ATGGTCACTC CAGCCGCTGA GCTCCAGGCA ATCAAGTGTG AGTTTTCT

ATGATTGCCT TGACCACTGA CCTCCAGGCT ACCAAGCATG GATTTTCT

>Ortholog Group 13, Repeat 2

3 198

R00055547 ---------- ---------- ---------- ---------- ----------

M00119120 TCAGAGCCC- -----CACGA CGTCGACCAG CCAGCTGGCC TTTCTGAGCT

H00350719 TCAGAGCCAG AGCATCAAGA AGCTCTCCAA CCAGTTAACC TTTCTGAATT

---------- ---------- ---------- ---------- ----------

TGACTCTGTC CTAACTGAAA GGCCACAGTT CAGCAGACAG AAGGATGCTA

GGAATCCATT GTAACTGAAA GGCCACAATT CAGCAGACAA AAAGATTTCG

---------- ---------- ---------- ---------- ----------

TTCTGGAGTT AAAACCATCA GAGCAGAAAG ATTTAATCAA GTTCACAGAA

TTCTGGAGTT AAAACCAATG GAACAGAAAG ATTTCATCAA ATTCATAGAA

---------- ---------- ---------- ---------- --------

CTGAATGCCA AGAAAACATG GCTGCAGGGT CACCAAGAGA ATGAAGAT

TTTAATGCTA AGAAAATGTG GCCCCAGTAT TGCCAACATG ATAACGAT

>Ortholog Group 140, Repeat 1

3 270

M00024717 GGCAGTATGG GTGGCCCTGT ACCCGGAAAC AACAGCAGCA GTTCCTTCTC

H00402935 GGCAGTGCAG GTGGCCCTGT ACCTGGAGGC AACAGCAGTA GCTCCTCCTC

R00014171 GGCAGTATGG GTGGCCCTGT GCCTGGGAGC AACAGCAGCA GCTCTTTCTC

TGCTGCCATC CCCACCAGGA CCTCCACAGA AGCCCCAAGG CATCATTTGC

TGTTGTAATT CCTACCAGGA CCTCAGCAGA GGCCCCAAGC CATCACTTG-

TGCTGCCGTC CCCTTCAGGA CCTCCGCAGA AGCCCCGAGG CATCATTCTC

TGCAGCAGCA ACAGCAGCAG CAGCACCAGC AGCAGCAGCA GCAGCAACCA

---------- ---------- ---------- ---------- ----------

AGCAGCAGCG GCAGCAGCAG CAGCAGCAGC AGCAGCAGCA GCAGCAG---

CAGCAGCAGC AGCAGCAACA CAGACTGATG AAATCTGAAA GCATGCCGGT

---------- ---------- -AGGCTGATG AAATCAGAAA GCATGCCTGT

---------- ---------- -CGGTTGATG AAGTCTGAAA GCATGCCGGT

ACAGTTGAAC AAAGGCGACG TAGTTACAGG AAGCGATGCT CAGGTTTCTG

GCAATTGAAC AAAGGCGATG TAGTTACAGG AAGCGATGCT CAGGTTTCTG

ACAGTTGAAC AAA------- ---------- ---------- ----------

TTCCTGTCCA GGCTCTAACT

TTCCTGTCCA GACTCTAACT

---------- ----------

>Ortholog Group 141, Repeat 1

3 204

M00103539 TGCGTCTGGT CGTTCCGCGG GCGCCAGGGG ACCGGCAAGC AGCAGCCTCA

R00027062 TGCGTCTGGT CGTTCAGCGG GCGCCAGGGG ACCCGCAAGC AGCATTCTCA

H00303282 TGCGTCTCGT CCTTCCGCGG GCGCCAGGGG GCCAGCAAGC AG------CA

GCCGGTGCCA ACGCCGCAGC CGCCTGAGTC CTCACCGCCG CCTCTGCCGC

GCCGGCGCCA ACGCCGCAGC CGCCTGAGTC CTCACCGCCG CCTCTGCTAC

GCCGGCGCCA CCGCCGCAGC CGCCCGAGTC CCCGCCGCCG CCACCGCTGC

CGCCGTGCTC TCAGCCCGGC ACTGCCGCCT CCCCGGCGGG TGCCCCGCTT

CGCCGTGCGC TCAGCCCGGC ACCGCCGCCT CCCCGGCGGG TGCCCCGCTT

CGCCGCCTGC GCAGCCCGGC CCCGCCGCGT CCCCGGCGGG CCCCCCGGCA

TCCTGCGGGC CTGGGGGCCG GCGTGCCGAG CCATGCCCCG GGCTGCCGGC

TCCTGTGGGC CTGGGGGCCG GCGCGCCGAG CCATGCTCCG GGCTGCCGGC

CCCCGCGGGC CCGGGGACCG GCGCGCCGAG CCATGCCCCG GGCTGCCGGC

GGTG

GGTG

GGCG

>Ortholog Group 142, Repeat 1

3 108

H00419704 ATGGAGCGGC TGCGAAACCT GCGTGACTTC CTGTTGGTCT ACAATCGGAT

M00102398 ---------C TGAGAAACTT GCGAGACTTC CTGTTGGTCT ACAATCGGAT

R00000351 CTGGGCGTGC TGAGAAACTT GCGAGACTTT CTGTTGGTCT ACAATCGGAT

GACAGAACTC TGCTTCCAGC GCTGTGTGCC CAGCTTGCAC CACCGAGCTC

GACAGAACTG TGTTTCCAGC GCTGTGTGCC CAGCCTGCAC CACCGAGCTC

GACAGAACTG TGCTTCCAGC TCTGTGTGCC CAGCCTGAAC CACCGAGCTC

TGGACGCT

TGGACGCT

TGGACGCT

>Ortholog Group 143, Repeat 1

3 198

M00073616 GTCCGCATGG TGAGACAATT CCATTTCCAT GGCTGGCCTG AGGTTGGCAT

R00021359 GTCCGGATGG TGAGACAATT CCATTTCCAT GGCTGGCCTG AGGTTGGAAT

H00254667 GTCCGAGTAG TGCGCCAGTT TCACTTCCAC GGCTGGCCTG AGATCGGGAT

CCCCGCTGAA GGCAAAGGCA TGATTGACCT GATTGCAGCA GTGCAGAAGA

CCCCACTGAG GGTAAAGGCA TGATTGACCT GATCGCGGCA GTGCAGAAGA

TCCCGCCGAG GGCAAAGGCA TGATTGACCT CATCGCAGCC GTGCAGAAGA

CGGGCAACCA CCCCATCACC GTGCACTGCA GCGCGGGAGC AGGGCGGACA

CAGGCAACCA CCCCATCACC GTGCACTGCA GTGCGGGAGC AGGGCGGACA

CAGGCAACCA CCCCATCACC GTGCACTGCA GTGCCGGAGC TGGGCGAACA

GGTACATTCA TAGCACTCAG TAACATTTTG GAACGAGTGA AAGCCGAG

GGTACATTCA TAGCACTCAG TAACATTTTG GAACGAGTGA AAGCCGAG

GGTACATTCA TAGCCCTCAG CAACATTTTG GAGCGAGTAA AAGCCGAG

>Ortholog Group 144, Repeat 1

3 198

H00351416 TTGGAGGAAC TACAGAAAGT AGAACGAGAG TTACAACTGA AAACTCAGCA

M00014421 CTGGAGGAGC TGCAGAAAGT AGAGCGAGAG CTACAACTGA AAACTCAGCA

R00032591 CTGGAGGAAC TGCAGAAAGT AGAGCGAGAG CTACAACTGA AAACTCAGCA

GCAGCTAAAA AAGCAGTATC TAGAGGTTAA AGCTCAAAGA ATTCAACTTT

GCAGCTTAAA AAACAGTATC TAGAAGTTAA AGCTCAACGA ATTCAGCTTT

GCAGCTTAAA AAACAGTATC TAGAAGTTAA AGCCCAGCGG ATTCAGCTGT

CTTGCCAACA CCTGGGATTA CTAACTCCTG TTGGAGTTGG AGAGCAGCTT

CTTGCCAGCA TCTGGGACTA TTTACTTCTG TTGGGGTTGG AGAGCAGCTT

CTTGCCAGCA TCTGGGACTC CTTACTTCTG TTGGGGTTGG AGAGCCGCTT

TCTGAGGGAG ACTATGCACG GTTACAGCAA GTGGATCCTG TTTTACTT

TCCGAGGGAG ACTATGCACG GTTACAGCAA GTGGATCCTG TTTTGCTT

CCTGAGGGAG ACTATGCACG GTTACAGCAA GTGGATCCTG TTTTGCTT

>Ortholog Group 145, Repeat 1

3 198

R00028825 AGTCGGCAGA TCCGGCAGTT CCACTTCCAC GGCTGGCCTG AGGTGGGCAT

M00076533 AGTCGGCAAA TCCGGCAGTT CCACTTCCAC GGCTGGCCTG AGGTGGGCAT

H00351559 AGCCGGCAGA TCCGGCAGTT CCACTTCCAT GGCTGGCCTG AAGTGGGCAT

CCCCAGCGAT GGGAAGGGTA TGATCAACAT CATTGCAGCA GTGCAGAAGT

CCCCAGCGAC GGCAAGGGCA TGATCAACAT CATTGCAGCA GTGCAGAAGT

CCCCAGTGAC GGAAAGGGCA TGATCAGCAT CATCGCCGCC GTGCAGAAGT

CAGGGAACCA TCCCATCACT GTGCACTGCA GTGCCGGGGC AGGACGGACA

CGGGGAACCA TCCCATCACT GTGCACTGCA GTGCCGGGGC AGGACGGACA

CAGGGAACCA CCCCATCACC GTGCACTGCA GCGCCGGGGC AGGAAGGACG

GGGACCTTTT GTGCCTTGAG CACAGTCCTG GAGCGCGTGA AAGCAGAA

GGAACCTTCT GTGCCTTGAG CACAGTCCTG GAACGTGTGA AAGCAGAA

GGGACCTTCT GTGCCCTGAG CACCGTCCTG GAGCGTGTGA AAGCAGAG

>Ortholog Group 146, Repeat 1

3 198

M00031640 CTTCCCACGT CAGATGTTTT CGCCGGTTGT CAGATCCCAT ATCCCAAACG

R00057821 ---------- ---------- ---------- ---------- ----------

H00370938 CTTCCTACAT CAGACGTTTT TGCCGGTTGT CAAATCCCTT ACCCAAAACG

AGAATTTTTA ACAGAAGAAG AGCCTGATGA GAAAGGAGAC AAAAAGACCG

---------- ---------- ---------- ---------- ----------

AGAATTTTTA ACGGAAGAAG AACCTGATGA CAAAGGAGAC AAAAAGAACG

GCAACAACCA CACTAACGGA ACTGGCCATC CGGGGAACCA GGACAGCGGC

---------- ---------- ---------- ---------- ----------

GCAATAACCA CACTAATGGA ACTGGCCACC CAGGGAATCA AGACAGCAGT

CACGCACAGG GGCCCCCCTT GAAAAAAGTG AGAGTTGTCC CTCCTACC

---------- ---------- ---------- ---------- --------

CACACACAGG GACCCCCGTT GAAGAAAGTG AGAGTTGTTC CTCCTACC

>Ortholog Group 147, Repeat 1

3 126

M00038369 ATGAAGAGGT GCAAATCGGA CGAGCTGGGC GAGGAGGATG GGGCTGGGAT

R00000036 ATGAAGAGGT GCAAATCGGA TGAGCTGGGC GAGGAGGATG GGGCTGGGAT

H00350718 ATGAAGAGGT GCAGATCGGA CGAGCTGGGC GAGGAGGATG GAGCTGGGCT

GGAAGACGCT GCTTGCCTTC TGCCAGGCGC GGACCTCCGG CATGGGGAGG

GGAAGACGCC GCTTGCCTTC TGCCAGGCGC GGACCTCCGG CATGGGGAGG

GGAAGATGCC GCTTCCCACC TGCCGGGCGC GGACCTCCGG CCTGGGGAGA

CCTCGAGTGC TAACTCCGCT GGCGGG

CCTCGAGTGC TAACTCCGCT GGCGGG

CCACGGGTGC TAACTCTGCT GGCGGG

>Ortholog Group 147, Repeat 2

3 198

M00038369 GAGGGCTCCA TGTTTGGCCA CGGCCTGAAG CACCTGTTTC ACAGCCGCCG

R00000036 GAGGGCTCCA TGTTTGGCCA CGGCCTGAAG CACCTGTTTC ACAGCCGCCG

H00350718 GAGGGCTCCA TGTTTGGCCA CGGTCTGAAG CACCTGTTCC ACAGCCGCCG

CAGGTCACGG GAGAGGGAGC ACCAGGCGTC TCAGGAGGCC CAGCAGCAGG

CAGGTCACGG GAGAGGGAGC ACCAGGCGTC TCAGGAGGCC CAGCAACAGG

TCGGTCTCGG GAAAGGGAGC ACCAGACGTC TCAGGATTCC CAGCAGCATG

GCCTATCCGA TCAGGACTCC CCAGATGAGA AGGAACGCTC CCCGGAGATG

GCCTATCGGA TCAGGACTCC CCAGATGAGA AGGAGCGCTC CCCGGAGATG

GTATGTCCGA CCATGACTCC CCAGATGAGA AGGAGCGCTC TCCGGAGATG

CACCGCGTCT CCTATGCTGT GTCCCTGCAC GACCTGCCTG CCCGACCT

CACCGCGTCT CCTATGCTGT GTCCCTGCAC GACCTGCCCG CGCGACCT

CATCGCGTCT CCTACGCCAT GTCCCTGCAC GACCTGCCCG CCCGGCCC

>Ortholog Group 147, Repeat 3

3 198

M00038369 AGCATGGAGG AGAAGGTGGC CTACCAGTCC TACGAGAGGG CCCGGGATAT

R00000036 AGCATGGAGG AGAAGGTGGC CTACCAGTCC TACGAGAGGG CCCGGGATAT

H00350718 AGCATGGAGG AGAAGGTGGC CTACCAGTCC TATGAGAGGG CACGGGACAT

CCAGGAGGCC GTGGAGTCCT GCCTGACCCG AGTCACCAAG CTGGAACTGG

TCAGGAGGCC GTGGAGTCTT GCCTGACCCG TGTCACCAAG CTGGAGCTGG

CCAGGAGGCC GTGGAGTCCT GCCTGACCCG GGTCACCAAG CTGGAGCTGG

TGGTGCAGTT AGAAGGTGTG GAGAACGCCA ACGCGCGCGC CCTGCTGGGC

TGGTGCAGTT AGAAGGTGTA GAGAACGCCA ACGCGCGCGC TCTGCTGGGC

TGGTACAGCT GGAGGGCGTG GAGAATGCCA ACGCGCGGGC GCTGCTGGGC

AAGTTCATCA ACGTGATCCT GGCACTCATG GCCGTGCTGC TGGTGTTT

AAGTTCATCA ACGTGATCCT GGCACTCATG GCAGTGCTGC TGGTGTTT

AAGTTCATCA ACGTGATCCT GGCGCTCATG GCCGTGCTGC TGGTGTTC

>Ortholog Group 148, Repeat 1

3 132

M00024123 ATGAACTTCC AGGCAGGCGG GGGCCAGAGT CCTAGCCTGG CGGCTCCGGG

H00380413 ATGAACTTCC AGGCGGGCGG GGGGCAGAGC CCGAGCCTGG CGGCTCCGGG

R00017827 ---------- ---------- ---------- ---------- ----------

GACCGGCGGC GGCGGCGGCG GCGGCGCGGG GGGCGGCGGG CAGTTCGGCG

GGGCGGCGGC GCTGCCGCGC AGCAGCTCGT CTGCGGCGGG CAGTTCGGCG

---------- NNNGGCGGCG GCGGCGCGGG GGGCGGCGGG CAGTTCGGCG

GCGCGGGGCC CGGAGCC--- GGGGGTGGCG GC

GCGCGGGGCC CGGGGCCGGG GGCGGCGGCG GC

GCGCGGGGCC CGGAGCC--- GGGGGTGGCG GC

>Ortholog Group 149, Repeat 1

3 198

H00256495 CCCGAACATC TCAAACTTAC AACTTTGGGT CACTTGGAAA AAGCAGTGGT

R00009564 CCCGAACATC TCAAACTTAC TACTTTGGGT CACTTGGAGA AAGCAGTGGT

M00032194 CCCGAACATC TCAAACTTAC TACTTTGGGT CACTTGGAAA AAGCAGTGGT

TCTTGAACTT ACCTTGAAGC ATGTGAAAGC ACTAACAAAC CTAATTGATA

TCTCGAGCTG ACGCTGAAGC ACGTGAAAGC ATTGACAAAC CTAATTGATA

TCTGGAGCTT ACGTTGAAGC ACGTGAAAGC ATTGACAAAT CTAATTGATA

AAATCATTGC CCTGCAGAGT GGTTTACAAG CTGGTGAGCT GTCAGGGAGA

AAATCATGGC CCTGCAGAGC GGTTTACAAG CTGGTGATCT GTCGGGAAGA

AAATCATTGC CCTGCAGAGC GGTTTACAAG CTGGTGATTT GTCGGGAAGA

AATGTCGAAA CAGGTCAAGA GATGTTCTGC TCAGGTTTCC AGACATGT

AATATTGAGG CAGGACAAGA AATGTTCTGC TCCGGTTTCC AGACCTGT

AATCTCGAGG CAGGGCAAGA AATGTTCTGC TCAGGTTTCC AGACTTGT

>Ortholog Group 14, Repeat 1

3 183

M00043874 AGCTCTCAGG AAAGAGCACC ATATGTGCAA AAAGCCAGAG ATAACAGGGC

R00010349 ---------- ---------- ---------- ---------- ----------

H00347325 AGCTCACAAG AAAGAGCACC ATATGTGCAA AAAGCCAGAG ATAACAGAGC

TGCTTTACGC ATAAATAAAG TTCAGATGTC AAACGATTCT ATGAAGAGGG

---------- ---------- ---------- ---------- ----------

TGCTTTACGC ATTAATAAAG TACAGATGTC AAATGATTCC ATGAAAAGGG

ACAGCATCGA TCCCAGCTCA CGCATCGATT CGGATCTTTT TAAAGATCCT

---------- ---------- ---------- ---------- ----------

ATAGCATTGA TCCCAGCTCT CGTATTGATT CGGAGCTTTT TAAAGATCCT

TTAAAGCAGA GAGAATCAGA GCATGAACAG GAA

---------- ---------- ---------- ---

TTAAAGCAAA GAGAATCAGA ACATGAACAG GAA

>Ortholog Group 14, Repeat 2

3 204

M00043874 AAGTTTAGAC AGCAAATGCG TCAGAAAAGT AAGCAACAAG CTAAAATTGA

R00010349 ---------- ---------- ---------- ---------- ----------

H00347325 AAATTTAGAC AGCAAATGCG TCAGAAAAGT AAGCAGCAAG CTAAAATTGA

AGCCACACAG AAGCTGGAAC AAGTGAAGAA TGAGCAGCAG CAGCAGCAGC

---------- ---------- ---------- ---------- ----------

AGCCACACAG AAACTTGAAC AGGTGAAAAA TGAG------ ----------

AGCAGCTTGC TTCTCAGCAC CTTCTGGTAG CACCTGGTTC AGATACTCCA

---------- ---------- ---------- ---------- ----------

-----TTTGG TTCTCAGCAT CTTCTGGTGC AGTCTGGTTC AGATACACCA

AGTAGTGGAG CACAGAGTCC CTTGACACCT CAGGCTGGCA ATGGGAATGT

---------- ---------- ---------- ---------- ----------

AGTAGTGGGA TACAGAGTCC CTTGACACCT CAGCCTGGCA ATGGAAATAT

GTCT

----

GTCT

>Ortholog Group 14, Repeat 3

3 198

M00043874 CCAACCTCAG GAGGAACTGA TACACAGAAC ACTGTAAACA TGTCTCAAGC

R00010349 ---------- ---------- ---------- ---------- ----------

H00347325 CCAACTTCAG GAGTAACTGA TACACAGAAT ACTGTAAATA TGGCCCAAGC

TGACACAGAG AAACTGAGAC AGCGGCAGAA ACTGCGTGAA ATCATTCTCA

---------- ---------- ---------- ---------- ----------

AGATACAGAG AAATTGAGAC AGCGGCAGAA GTTACGTGAA ATCATTCTCA

AGAAGATTGC TAGTCGCCAG GAGAAGGGGC CTCAGGATAC AGCAGTAGTA

---------- ---------- ---------- ---------- ----------

AGAAGATTGC AGGTCGACAG GAGAAGGGGT CACAGGACTC ACCCGCAGTG

CCTCACCCAG TGCCCCTTCC ACACTGGCAG CCAGAGAGCA TCAACCAG

---------- ---------- ---------- ---------- --------

CCTCATCCAG GGCCTCTTCA ACACTGGCAA CCAGAGAATG TTAACCAG

>Ortholog Group 14, Repeat 4

3 198

M00043874 ATTCCTCAAA CATTAGCCCA GCAGAACAGA GAGAGGCCCC TCCTTCTAGA

R00010349 ------CAGC AAATGCGTCA GAAAAGTAAG CAGCAAGCTA AAATTGAAGC

H00347325 ATTCCTCAAA CATTAGCACA GCAGAATAGA GAGAGGCCCC TTCTTCTAGA

GGAACAGCCT CTGCTTCTAC AAGATCTTTT GGATCAAGAG AGGCAGGAGA

CACACAGAAG CTGGAACAAG TGAAGAATGA ACAGCAGCAG CAGCAGCAGC

AGAACAGCCT CTACTTCTAC AGGATCTTTT GGATCAAGAA AGGCAAGAAA

GACAAATGCA AGCCATGATT CGTCAGCGGT CAGAACCATT CTTCCCTAAC

AACAACAACA ACAACTTGCT TCTCAGCACC TT-------- ----------

GACAGATGCA AGCCATGATT CGTCAGCGAT CAGAACCGTT CTTCCCTAAT

ATTGATTTTG ATGCTATTAC AGATCCTATA ATGAAAGCGA AAATGGTA

CTAGATTTCG ATGCAATAAC AGATCCTATA ATGAAAGCGA AAATGGTA

ATTGATTTTG ATGCAATTAC AGATCCTATA ATGAAAGCCA AAATGGTG

>Ortholog Group 14, Repeat 5

3 207

M00043874 CATGTTACTG AGCAGCAGAG CATGGTTCAG AAACAGCTTG AGCAGATTCG

R00010349 CATGTTACTG AGCAGCAGAG CATGGTTCAG AAACAACTTG AGCAGATTCG

H00347325 CATGTTACTG AACAGCAAAG CATGGTTCAG AAACAGCTAG AACAGATTCG

GAAACAACAG AAAGAGCATG CTGAGCTGAT TGAAGATTAT CGGATCAAAC

TAAACAACAG AAAGAGCATG CTGAGCTGAT TGAAGATTAC CGGATCAAGC

TAAACAACAG AAAGAACATG CTGAATTGAT TGAAGATTAT CGGATCAAA-

AGCAGCAGTG TGCCCTAGCC CCTCCCATCC TCATGCCAGG GGTTCAGCCC

AGCAGCAGTG CGCCCTAGCC CCTCCCATCC TCATGCCAGG GGTTCAGCCC

--------TG TGCAATGGCC CCACCTACCA TGATGCCCAG TGTCCAGCCC

CAGCCACCTC TAGTTCCAGG TGCCACTTCA CTTACCATGA GCCAACCCAA

CAGCCACCTC TAGTTCCAGG TGCCAGCCCA CTCACCATGA GCCAACCCAA

CAGCCACCCC TAATTCCAGG TGCCACTCCA CCCACCATGA GCCAACCCAC

CTTTCCC

CTTCCCC

CTTTCCC

>Ortholog Group 150, Repeat 1

3 171

R00027738 ATGGCTAACA ACAGCCCCGC GCTGACCGGC AACTCGCAAC CGCAGCACCA

M00029480 ATGGCCAACA ACAGCCCCGC GCTGACCGGC AACTCGCAAC CGCAGCACCA

H00359042 ATGGCTAACA ACAGCCCCGC GCTGACAGGC AACTCGCAGC CGCAGCACCA

GGCGGCTGCG GCCGTGGTCT GT---GGCGG CGGCGGCGCC ACCAAGCCGG

GGCGGCCGCG GCCGTGACCT GTGGCGGCGG CGGCGGCGCC ACCAAGCCGG

GGCGGCTGCA GCTGCGGCTT GC---GGCGG CGGCGGCGCT ACCAAGCCGG

CGGTGTCGGG CAAGCAGGGC AATGTGCTGC CGCTGTGGGG CAATGAGAAG

CGGTGTCGGG CAAGCAGGGC AATGTGCTGC CGCTGTGGGG CAACGAGAAG

CGGTCTCCGG CAAGCAGGGC AATGTGCTCC CGCTCTGGGG CAACGAGAAG

ACCATGAACC TCAACCCCAT G

ACCATGAACC TCAACCCCAT G

ACCATGAACC TCAACCCCAT G

>Ortholog Group 151, Repeat 1

3 198

R00020729 ---------- ---------- ---------- ---------- ----------

H00308927 GCTGCCCACG ATGAGCAGAA GAAACTAGCT GCCTCTCAGA TTGAGAAACA

M00047567 GCTGCCCACG ATGAGCAGAA GAAGTTGGCT GCATCTCAGA TAGAGAAACA

---------- ---------- ---------- ---------- ----------

GCGTCAGCAA ATGGAGCTGG CCAAGCAGCA ACAAGAACAA ATTGCAAGAC

GCGTCAGCAG ATGGAGCTGG CCAAGCAGCA ACAGGAGCAG ATCGCGAGGC

---------- ---------- ---------- ---------- ----------

TTCTACAGCA ACAACACAAA ATCAATTTGC TCCAGCAACA GATCCAGGTT

TTCTGCAGCA ACAACACAAA ATCAACTTGC TTCAGCAACA GATCCAGGTT

---------- ---------- ---------- ---------- --------

CAAGGTCAGC TGCCGCCATT AATGATTCCC GTATTCCCTC CTGATCAA

CAAGGTCAGC TGCCGCCATT GATGATTCCC GTGTTCCCTC CTGACCAG

>Ortholog Group 152, Repeat 1

3 231

H00256972 CGTCAGGGAC CACCTTTGGG AGGACAGCAA TCTCAACCCT CTGCTGGTGA

R00048827 AATCAGAAAC CACCTCCACC AGGATTCCCA CCAAGACCAC CTGCT-----

M00073689 AATCAAAGGC CACCTCCTTC AGGGTTCCAA CCAAGACCGC CTGTT-----

TGGGAACCAG AATGATGGCC CTCAGCAGGG ACCACCCCAA CAAGGAGGCG

---------- ---AATGGGA GCCAGCAAGG CCCACCCCCA CAAGGAGGCG

---------- ---AATGGGA GCCAGCAAGG CCCACCACCA CCAGGAGGCG

GTCCACCACC TCCTCAGGGA AAGCCACAAG GACCACCCCA ACAGGGAGGC

GCCCACCCCC ACCAGGAGGC CCACAGCAGA AACCCCCTCA GCCTGGAAAC

GCCCACCACC ACCAGGAGTA CCACAGCCGA GACCCCCT-- ----------

CAT------C CCCCTCCTCC TCAAGGA--- ---------- ----------

CAACAAGGCC CACCCCCACC AGGAGGCCCA CAACAGAAAC CAACTCAGCC

---CAAGGCC CACCACCACC AGGAGGCCCA CAGCAGAGAC CC--------

----AGGCCA CAAGGACCAC CCCAACAGGG A

TGAAAAGCCC CAAGGCCCAC CCCCACCAGG A

-------CCT CAAGGCCCAC CACCACCAGG A

>Ortholog Group 153, Repeat 1

3 198

R00008550 GCACCAGCTT ACACTCAACT GCAGCCACAC CAACTCCTCC CACAGCCGTC

M00101690 GCACCAGCTT ATGCTCACCT GCAGTCACAC CAGCTCCTCC CACAGCCGCC

H00391440 GCACCAGCCT ATGCTCAGCT GCAGCCACAC CAGCTCCTCC CACAGCCATC

ATCAAAGCAT CCACAGCCCC AGTTTGTGGC CCAGCAGCAA CCACAGCCA-

AGCGAAGCAC CCACAGCCCC AGTTTGTGGC CCAACAGCAA CCACAGCCA-

CTCAAAGCAC CTGCAGCCCC AATTTGTGAT CCAGCAGCAG CCACAGCCAC

---------- ----CCACGG CCTACACCCC AAGTCCAGTC CCAACCCCAG
[truncated: 539,310 more chars]
